# Tandem dinucleophilic cyclization of cyclohexane-1,3-diones with pyridinium salts

**DOI:** 10.3762/bjoc.9.124

**Published:** 2013-06-10

**Authors:** Mostafa Kiamehr, Firouz Matloubi Moghaddam, Satenik Mkrtchyan, Volodymyr Semeniuchenko, Linda Supe, Alexander Villinger, Peter Langer, Viktor O Iaroshenko

**Affiliations:** 1 Institut für Chemie der Universität Rostock, Albert-Einstein-Straße 3a, D-18059 Rostock, Germany, Fax: (+49-381-498-6411); 2 Laboratory of Organic Synthesis and Natural Products, Department of Chemistry, Sharif University of Technology, P. O. Box 11155-9516 Tehran, Iran; 3 Department of Chemistry, Faculty of Science, University of Qom, Qom, Iran; 4 National Taras Shevchenko University, Volodymyrska st 62, Kyiv-33, 01033, Ukraine; 5 Leibniz Institut für Katalyse e.V. an der Universität Rostock, Albert-Einstein-Straße 29a, D-18059 Rostock, Germany

**Keywords:** cyclization, density functional calculations, heterocycles, nucleophilic addition, pyridinium salt

## Abstract

The cyclization of cyclohexane-1,3-diones with various substituted pyridinium salts afforded functionalized 8-oxa-10-aza-tricyclo[7.3.1.0^2,7^]trideca-2(7),11-dienes. The reaction proceeds by regioselective attack of the central carbon atom of the 1,3-dicarbonyl unit to 4-position of the pyridinium salt and subsequent cyclization by base-assisted proton migration and nucleophilic addition of the oxygen atom to the 2-position, as was elucidated by DFT computations. Fairly extensive screening of bases and additives revealed that the presence of potassium cations is essential for formation of the product.

## Introduction

The nucleophilic addition to pyridinium salts has recently been proven to be a powerful method for the construction of various dihydropyridines [1–4], which are not only valuable intermediates in organic synthesis [5–12], but also interesting compounds in medicinal [13] and bioorganic chemistry [14–16]. The tetrahydropyridine ring is an essential building block for numerous natural products, synthetic pharmaceuticals, and various biologically active compounds [17]. The tetrahydropyridine scaffold is inherent for pharmacologically relevant representatives [18–24]. At the same time, heterocyclic systems containing an annulated pyridinium core, such as quinolinium and isoquinolinium salts, are of considerable importance as building blocks for the synthesis of various alkaloid frameworks [10,25–35].

During the past decade the reaction of dinucleophiles with dielectrophiles has been a major research subject in both our laboratories. For example, we have studied reactions of quinolinium [36–37], isoquinolinium [38–40], quinazolinium [41–42] and quinoxalinium [43] salts with 1,3-bis(silyl enol ethers), i.e., masked 1,3-dicarbonyl compounds, which provided facile access to a number of bicyclic systems. Some of these reactions, such as the cyclization with quinazolinium salts, proceeded as one-pot cyclizations. Other cyclizations, such as the reaction with isoquinolinium salts, had to be carried out in two steps (formation of open-chained condensation products, which are subsequently cyclized by the addition of acid). In some cases the cyclization step failed, e.g., the reaction of 1,3-bis(silyl enol ethers) with pyridine in the presence of methyl chloroformate resulted in the formation of 1,4-dihydropyridines, which however, could not be cyclized by the addition of acid due to decomposition [36]. Additionally, we have shown a broad application of the quinolinium [44–48] and isoquinolinium [49] salts for the synthesis of a wide variety of alkaloid-like frameworks.

## Results and Discussion

### Reaction optimization

**Scheme 1:**
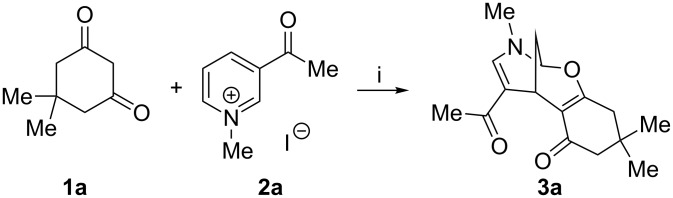
Reagents and conditions: (i) CH_3_CN, K_2_CO_3_, rt, 24 h.

During the course of the above-mentioned study we discovered an interesting reaction that represents an external dinucleophilic addition of the dimedone molecule to the pyridinium salt **2a**, taken as a random example, delivering 8-oxa-10-aza-tricyclo[7.3.1.0^2,7^]trideca-2(7),11-diene (**3a**) in modest yield (Scheme 1). Previously, we have communicated a similar cycloaddition reaction using quinolinium salts [45], and recently Li and Yan [50] reported this reaction with phenanthrolinium salts.

This efficient cycloaddition encouraged us to investigate this process, to improve the reaction conditions, and to extend the reaction with other pyridinium salts and diketones. Herein we report the results of that study. First, we studied the reaction of pyridinium salt **2a** with dimedone (**1a**) using various bases in different solvents (Table 1). At the same time, different ratios of **1a** and **2a** were examined. To reach the optimal yields, dimedone and pyridinium salt **2a** should be used in a ratio of two to one. In addition, the optimization study showed the essential role of a non-nucleophilic base as well as that of the potassium cation.

**Table 1:** Optimization of the reaction conditions.

Entry	Conditions^a^	Yield **3a** (%)

1	**1a** (1 equiv)/CH_3_CN/K_2_CO_3_/rt/24 h	68
2	**1a** (2 equiv)/CH_3_CN/K_2_CO_3_/rt/24 h	**85**
3	**1a** (2 equiv)/CH_3_CN/K_2_CO_3_/rt/12 h	72
4	**1a** (2 equiv)/CH_3_CN/KHCO_3_/rt/4 h	74
5	**1a** (2 equiv)/CH_3_CN/KF/rt/24 h	0
6	**1a** (2 equiv)/CH_3_CN/K_3_PO_4_/rt/24 h	0
7	**1a** (2 equiv)/AcOH/AcOK/rt/12 h	0
8	**1a** (2 equiv)/AcOH/AcOK/100 °C/3 h	0
9	**1a** (2 equiv)/CH_3_CN/NaHCO_3_/rt/4 d	12
10	**1a** (2 equiv)/CH_3_CN/Na_2_CO_3_/rt/24 h	17
11	**1a** (2 equiv)/CH_3_CN/Na_2_CO_3_/KI (0.5 equiv)	32
12	**1a** (2 equiv)/CH_3_CN/Na_2_CO_3_/KI (1 equiv)	42
13	**1a** (2 equiv)/CH_3_CN/Na_2_CO_3_/KI (2 equiv)	68
14	**1a** (2 equiv)/CH_3_CN/Na_2_CO_3_/LiI (2 equiv)	0
15	**1a** (2 equiv)/CH_3_CN/Cs_2_CO_3_/rt/12 h	32
16	**1a** (2 equiv)/CH_3_CN/Cs_2_CO_3_/KI (0.5 equiv)	34
17	**1a** (2 equiv)/CH_3_CN/Cs_2_CO_3_/KI (1 equiv)	44
18	**1a** (2 equiv)/CH_3_CN/Cs_2_CO_3_/KI (2 equiv)	68
19	**1a** (2 equiv)/CH_3_CN/Cs_2_CO_3_/NaI (0.5 equiv)	8
20	**1a** (2 equiv)/CH_3_CN/Cs_2_CO_3_/NaI (1 equiv)	10
21	**1a** (2 equiv)/CH_3_CN/Cs_2_CO_3_/NaI (2 equiv)	12
22	**1a** (2 equiv)/CH_3_CN/Cs_2_CO_3_/LiI (2 equiv)	0
23	**1a** (2 equiv)/CH_3_CN/Li_2_CO_3_/rt/24 h	0
24	**1a** (2 equiv)/CH_3_CN/Li_2_CO_3_/KI (0.5 equiv)	35
25	**1a** (2 equiv)/CH_3_CN/Li_2_CO_3_/KI (1 equiv)	40
26	**1a** (2 equiv)/CH_3_CN/Li_2_CO_3_/KI (2 equiv)	71
27	**1a** (2 equiv)/CH_3_CN/Li_2_CO_3_/NaI (0.5 equiv)	7
28	**1a** (2 equiv)/CH_3_CN/Li_2_CO_3_/NaI (1 equiv)	10
29	**1a** (2 equiv)/CH_3_CN/Li_2_CO_3_/NaI (2 equiv)	10
30	**1a** (2 equiv)/CH_3_CN/organic base^b^/rt/24 h	0
31	**1a** (2 equiv)/CH_3_CN/organic base^b^ (except DIPEA)/KI, KBr, NaI or LiI/rt/24 h	0
32	**1a** (2 equiv)/CH_3_CN/DIPEA/KI (0.5 equiv)	45
33	**1a** (2 equiv)/CH_3_CN/DIPEA/KI (1 equiv)	58
34	**1a** (2 equiv)/CH_3_CN/DIPEA/KI (2 equiv)	75
35	**1a** (2 equiv)/CH_3_CN/DIPEA/KBr (2 equiv)	68
36	**1a** (2 equiv)/CH_3_CN/DIPEA/NaI (0.5 equiv)	22
37	**1a** (2 equiv)/CH_3_CN/DIPEA/NaI (1 equiv)	28
38	**1a** (2 equiv)/CH_3_CN/DIPEA/NaI (2 equiv)	34
39	**1a** (2 equiv)/CH_3_CN/DIPEA/LiI (0.5–2 equiv)	0

^a^For screening we used 2 equiv of base; ^b^as organic bases we tested pyridine, 2,4,6-trimethylpyridine, NEt_3_, DABCO and DIPEA; in all cases 2 equiv of base were added.

This optimization study afforded a general procedure and optimal reaction conditions: 2.0 mmol of diketone, 1.0 mmol of the appropriate pyridinium salt and 1 mmol of K_2_CO_3_ in 7 mL of CH_3_CN are stirred at room temperature for 24 hours.

### Reaction scope

**Scheme 2:**
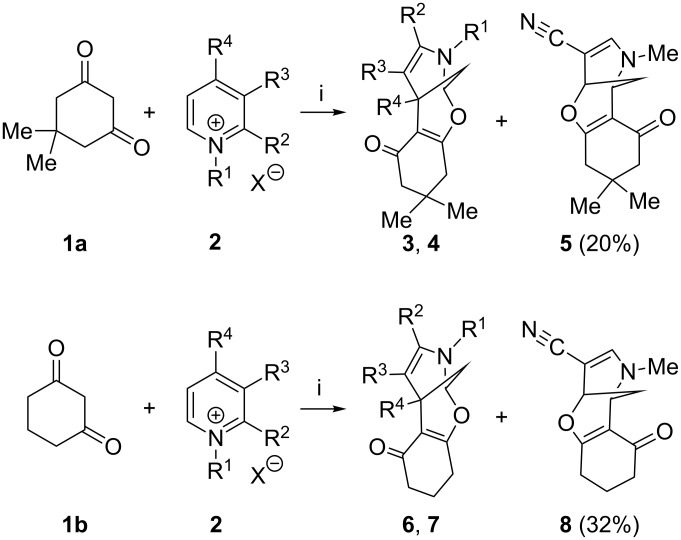
Synthesis of compounds **3**–**8**. Reagents and conditions: (i): CH_3_CN, K_2_CO_3_, rt, 24 h. In the case of 3-cyanopyridinium salts, NaHCO_3_ (2 equiv) was used as a base and the reaction time was 4 days.

**Figure 1:**
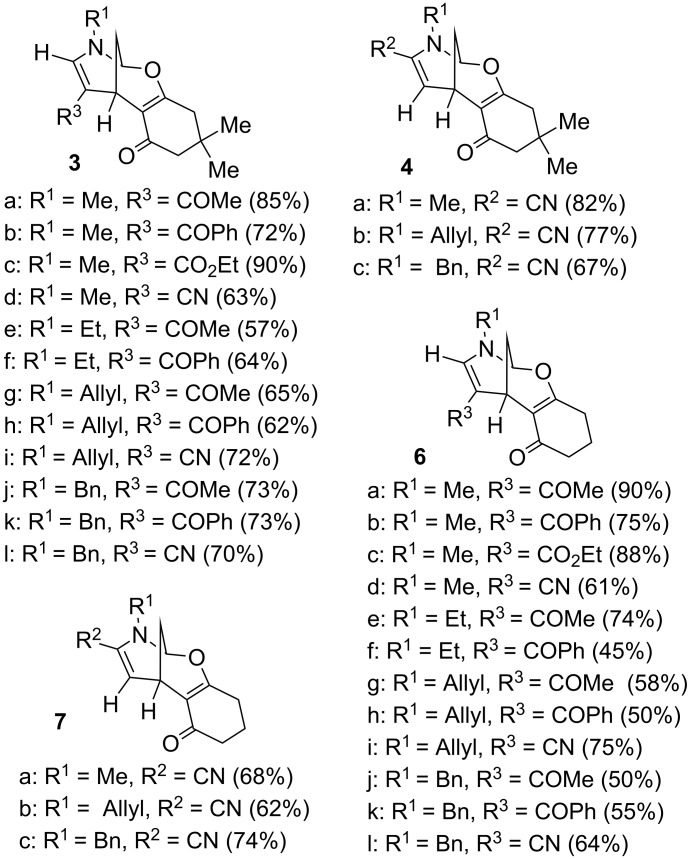
Synthesized compounds **3**, **4**, **6**, **7**.

Having found the optimal reaction conditions, we concentrated on studying the scope and limitations of the discovered cycloaddition. A variety of pyridinium salts with different substituents in positions 1, 2, 3 and 4 were prepared by alkylation of the parental commercially available pyridines (see Supporting Information File 1, Table S1). The results of this cyclization reaction (Scheme 2) are summarized in Figure 1. Generally the reaction was complete in 24 hours and afforded the desired products in good to excellent yields. It is worth mentioning that only cyclohexanediones were prone to reaction with the tested pyridinium salts with formation of the corresponding tetracyclic products **3**, **4**, **6** and **7**. In the frame of the current project a variety of 1,3-diketones and CF_3_-containing 1,3-diketones were investigated, but these failed for all mentioned cases. For 3-cyanopyridinium salts a departure from the general trend was observed in terms of reaction conditions regarding the base used; here the highest efficiency was observed for NaHCO_3_, and the reaction took 4 days to reach completion. In general, the reaction proceeded with high regio- and diastereoselectivity.

Additionally, the reaction was monitored by HPLC, and for most pyridinium salts investigated no byproducts were detected. However, in the case of 3-cyanopyridinium salts the formation of additional products, namely the constitutive isomers of type **5** and **8**, was observed. These compounds were isolated and identified by using standard 2D NMR methods and X-ray analysis [51].

At the same time, to our great disappointment 4-substituted pyridinium salts appeared to be unreactive, and testing different reaction conditions as well as bases with various combinations of time and temperature did not deliver any positive results.

During the course of this work we encountered the low stability of 8-oxa-10-aza-tricyclo[7.3.1.0^2,7^]trideca-2(7),11-dienes; decomposition was observed in polar solvents, i.e., in DMSO. In some cases this had an influence on the quality of the ^1^H NMR spectra. Heating also led to decomposition, which was observed after 2–3 hours at 60–70 °C.

The constitution of products **3**, **4**, **6**, **7** was proven by 1D and 2D NMR experiments; that of compounds **6d**, **7c** and **8** was unambiguously corroborated by X-ray [51] diffraction analysis (Supporting Information File 1, Figures S2, S3, S4)

### Reaction mechanism

In order to shed light on the reaction mechanism we used DFT computations at the B3LYP/6-31G(d,p) [52–54] level, as implemented in the Gaussian 09 program [55], and acetonitrile was included implicitly within the IEFPCM method (more details on inclusion of the solvent can be found in Supporting Information File 2). For simplicity 1,3-cyclohexanedione rather than dimedone was included in the computations.

Our ability to calculate the mechanism as well as to make any further predictions about reaction products is limited by the obscure structure of the real reaction product. In fact the reaction of pyridinium salt **2a** with cyclic 1,3-diketones in the presence of either K_2_CO_3_ or DIPEA/KI yields an orange precipitate in acetonitrile. Redissolved in DMSO-*d*_6_, this precipitate gave rise to broad peaks in the ^1^H NMR spectra; thus its structure was not defined and is supposed to be of polymeric nature. Potassium with a counter-ion (HCO_3_^−^ or I^−^) is also included in the precipitate since that cannot be burned completely and yields an unburned material. On the other hand the orange precipitate is quickly decomposed by silica gel liberating the desired product **3a** or **6a**, which was finally eluted from silica. The formation of a precipitate must be a driving force of the reaction promoted by DIPEA/KI (see Supporting Information File 2, thermodynamic analysis).

The reaction starts with 1,3-diketone deprotonation and nucleophilic attack of potassium 3-oxocyclohex-1-enolate on pyridinium cation, which yields an intermediate with a new C–C bond, and this is a key stage in the reaction. The C–O bond formation can be excluded in the initial stage (see Supporting Information File 2 for the explanation). Bearing this in mind, we computed the local softness of 3-acetyl-1-methylpyridinium (**2a**^+^), 2-cyano-1-methylpyridinium (**2b**^+^) and 3-cyano-1-methylpyridinium (**2e**^+^) cations towards nucleophilic attack. According to the local softness indexes (see Supporting Information File 2) with **2b**^+^ cation we should observe a regioselective formation of product **4a** or **7a,** while for cations **2a**^+^ and **2e**^+^ a regioisomeric mixture should be expected (but is really only observed for **2e**^+^). In reality, regioisomeric mixtures of compounds **3**/**5** or **6**/**8** are produced by cation **2e**^+^, while for cations **2a**^+^ and **2b**^+^ regioselective reaction is inherent. Intrigued by this discrepancy, we computed the full reaction mechanism for cation **2a**^+^ with potassium 3-oxocyclohex-1-enolate in the presence of potassium cation and hydrogencarbonate anion as base. We included HCO_3_^−^ in our computation, both because it has only five atoms and because this base is presumed to operate; it is formed from neutral carbonate when it deprotonates the cyclic diketone. However, the mechanism involving any other base should not differ significantly.

**Figure 2:**
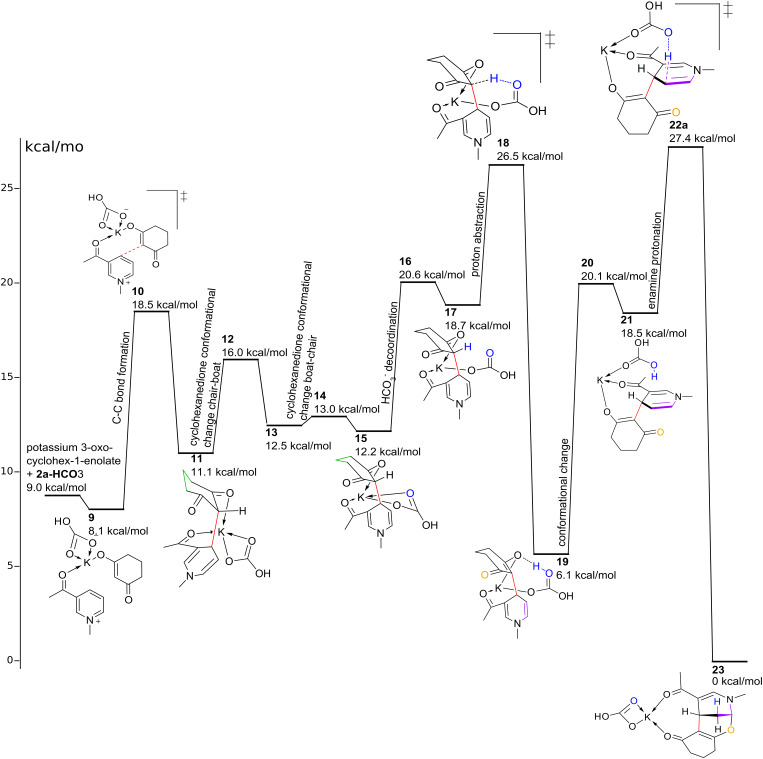
Plausible reaction mechanism.

A plausible mechanism is presented in Figure 2. After initial deprotonation of the diketone and formation of the hydrogencarbonate anion, the latter is exchanged with iodine in the salt of **2a**, and an initial complex consisting of **2a**^+^HCO_3_^−^ with potassium 3-oxocyclohex-1-enolate is assembled (intermediate **9**). Nucleophilic attack yields intermediate **11** through TS **10**. The latter TS was first localized without HCO_3_^−^ (TS **K-S1a-1**, see Supporting Information File 2); however, an inclusion of HCO_3_^−^ resulted in a slight energy drop. Cyclohexanedione ring inversion (intermediates **13**, **15** and TSs **12**, **14**) and HCO_3_^−^ semi-decoordination (TS **16**) lead to intermediate **17**, which undergoes proton abstraction under action of hydrogencarbonate (TS **18** and intermediate **19**). Structural rearrangement of **19** through TS **20** shows an intermediate **21**. Finally **21** is protonated by coordinated H_2_CO_3_ (highest rate-determining TS **22a**) to yield the final product **23** with a great release of free energy. It is worth mentioning that the last reaction is a protonation of enamine in essence; the imaginary frequency inherent for TS **22a** shows only proton displacement, without formation of a C–O bond. The latter was formed during IRC computation, starting from TS **22a**. Previously, Hui Li and Chao-Guo Yan [50] proposed the nucleophilic attack of oxygen onto the carbon atom with subsequent protonation. We have explored this process by decreasing the C–O distance in relaxed scans: the scan from intermediate **19** showed only a constant rise of energy, while the scan from intermediate **21** showed a maximum, which was not optimized to any TS. Hence, the attack by oxygen on the last stage must be excluded. Within the scope of this mechanism the activation energy is 21.3 kcal/mol (energy gap between **22a** and **19**).

Other TSs (**K-S1a-2**, **K-S1a-3**, see Supporting Information File 2), inherent for the initial stage of new C–C bond formation turned out to be unimportant, because in both cases reactions run again through TS **22a** (rate limiting), as was shown by additional computations. On the other hand, we have computed four conformers of TS **22**: the conformation of the cyclohexane ring and that of the acetyl group were changed. While the cyclohexane-ring conformation has only a negligible effect on the TS energy (TS **22b**, see Supporting Information File 2), the change of the acetyl group conformation prevents potassium from coordinating to it and makes the TS energy somewhat higher (TSs **22c** and **22d** from Supporting Information File 2).

**Scheme 3:**
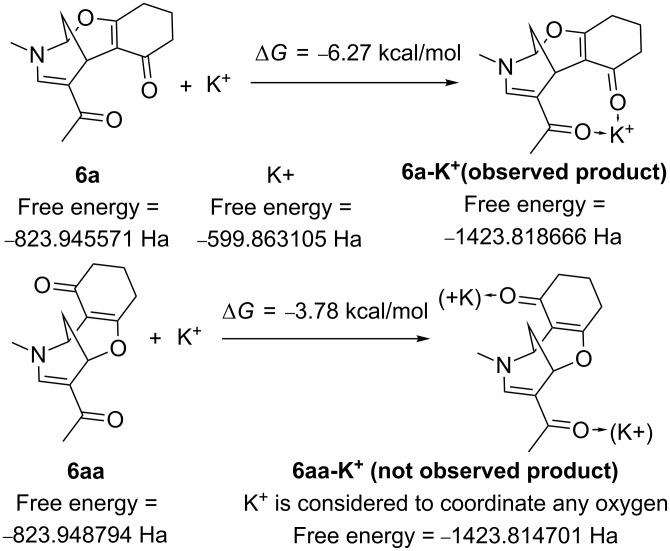
The influence of K^+^ on the product free energy.

In the proposed mechanism the regioselective formation of product **6a** is not explained (see Supporting Information File 2 for discussion). We attribute this to a higher thermodynamic stabilization of compound **6a** by a potassium cation (Scheme 3), as compared with that of regioisomeric **6aa** (its formation was not observed); the energetic distance between **6a**-**K**^+^ and **6aa**-**K**^+^ is 2.5 kcal/mol at the B3LYP/6-31G(d,p) level and 2.0 kcal/mol at the B3LYP/6-311++G(d,p)//B3LYP/6-31G(d,p) level. As mentioned before, the assumption is hard to prove due to lack of information about the real product composition.

## Conclusion

In summary, we have studied the dicomponent binucleophilic cycloaddition of 1,3-diketones with diverse substituted pyridinium salts. The reaction took place under mild conditions and provided an efficient route to 8-oxa-10-aza-tricyclo[7.3.1.0^2,7^]trideca-2(7),11-dienes with a wide range of substituents. Further studies to extend the scope of the synthetic utility of these cyclizations are in progress in both of our laboratories.

## Experimental

### General Information

Solvents were purchased from ACROS and directly used without further purification. Analytical thin-layer chromatography was performed on 0.20 mm 60 Å silica gel plates. Column chromatography was performed by using 60 Å silica gel (60–200 mesh). Pyridinium salts were synthesized according to the published procedure [56].

The density functional B3LYP [52–54] with 6-31G(d,p) basis set for all atoms except iodine and solvation by acetonitrile within the IEFPCM method as implemented in the Gaussian 09 package [55], was employed for the calculations. For cases when iodine was included, the LANL2DZ basis set and core potential were assigned. All compounds were computed in the closed-shell singlet state (confirmed by “stable” calculation). Frequency calculations were performed on all optimized geometries to ensure only positive eigenvalues for minima and one imaginary frequency for transition states. Free energies at 298.15 K and 1 bar pressure were calculated according to the recommendation of Gaussian, Inc. [57–58], and the frequency scaling factor (0.9608) was applied. For every located transition state an IRC calculation at the same level of theory confirmed that this TS connects the corresponding educts/intermediates/products. The methodology of local softness computation [59–60] uses Fukui functions; for their approximation we used Mulliken, ESP, NPA and Hirshfeld charges. However, only the Fukui functions approximated by NPA and Hirshfeld charges were unambiguous and taken into consideration.

### General procedures for the synthesis of compounds **3**–**8**

#### Procedure (A)

In a 25 mL Schlenk flask, under argon flow, 2.0 mmol of diketone, 1.0 mmol of the appropriate pyridinium salt, and 1.0 mmol (138 mg) of K_2_CO_3_ were loaded. The flask was covered with a septum stopper and 7 mL of absolute CH_3_CN was added by syringe. The reaction mixture was left under intensive stirring at room temperature for 24 hours. Then the solvent was removed under reduced pressure and the crude material was subjected to column chromatography.

#### Procedure (B)

In the case of the 3-cyanopyridinium salt, NaHCO_3_ (2.0 mmol, 168 mg) was used as a base, and the reaction mixture was left over 4 days.

#### Procedure (C)

In the case of 2-cyanopyridinium salts the reaction was completed within 1 hour.

## Supporting Information

File 1 Details on synthetic procedures, list of pyridinium salts, characterization of new compounds, copies of NMR spectra, X-ray structures of compounds **6d**, **7c** and **8**.

File 2 Computational results, optimized structures (atomic coordinates as reported by Gaussian 09), charges and local softness indexes (nucleophilic attack) for cations **2a**^+^, **2b**^+^ and **2e**^+^, additional discussions related to computations.
