# Supplementary material for: Tandem dinucleophilic cyclization of cyclohexane-1,3-diones with pyridinium salts
Source: Beilstein J Org Chem. 2013 Jun 10;9:1119–26. doi: 10.3762/bjoc.9.124 (PMC3701411; doi:10.3762/bjoc.9.124)
Supplement: File 1 — Details on synthetic procedures, list of pyridinium salts, characterization of new compounds, copies of NMR spectra, X-ray structures of compounds 6d, 7c and 8. [file Beilstein_J_Org_Chem-09-1119-s001.pdf]

Supporting Information File 1

for

Tandem dinucleophilic cyclization of cyclohexane-1,3-diones with pyridinium salts.

Mostafa Kiamehr[1,2,3], Firouz Matloubi Moghaddam[2]*, Satenik Mkrtchyan[1], Volodymyr Semeniuchenko[4], Linda Supe[1], Alexander Villinger[1], Peter Langer[1,5]*, Viktor O. Iaroshenko[1,4,§,]*

Address: [1]Institut für Chemie der Universität Rostock, Albert-Einstein-Straße 3a, D-18059 Rostock, Fax: (+49-381-498-6411), [2]Laboratory of Organic Synthesis and Natural Products, Department of Chemistry, Sharif University of Technology, P. O. Box 11155-9516 Tehran, Iran, [3]Department of Chemistry, Faculty of Science, University of Qom, Qom, Iran, [4]National Taras Shevchenko University, Volodymyrska st 62, Kyiv-33, 01033, Ukraine and [5]Leibniz Institut für Katalyse e.V. an der Universität Rostock, Albert-Einstein-Straße 29a, D-18059 Rostock.

Email: Firouz Matloubi Moghaddam* - Matloubi@sharif.edu, Peter Langer* – peter.langer@uni-rostock.de, Viktor O. Iaroshenko* - viktor.iaroshenko@uni-rostock.de

[§]further email address iva108@gmail.com

Details on synthetic procedures, list of pyridinium salts, characterization of new compounds, copies of NMR spectra, X-ray structures of compounds **6d**, **7c** and **8**.

Table of Contents

General information......S3

List of pyridinium salts used......S4

NMR, HRMS and IR spectral data......S5

Copies of ^{1}H and ^{13}C NMR spectra for compounds **3**, **4**, **5**, **6**, **7** and **8**......S16

X-ray structures of compounds **6d**, **7c**, **8**......S80

General information

Chemical shifts of the ^{1}H and ^{13}C NMR are reported in parts per million using the solvent internal standard ($CDCl_3$ 7.26 ppm and 77.0 ppm, DMSO-d_6 2.49 ppm and 39.7 ppm). Infrared spectra were recorded in an ATR apparatus. Mass spectrometric data (MS) were obtained by electron ionization (EI, 70 eV), chemical ionization (CI, isobutane) or electrospray ionization (ESI). Melting points are uncorrected. The solvents were purchased directly from ACROS and used without further purification. Analytical thin-layer chromatography was performed on 0.20 mm 60 A silica gel plates. Column chromatography was performed using 60 A silica gel (60–200 mesh).

General procedure for the synthesis of pyridinium salts.

Alkyl bromide or iodide (0.2 mol) was added dropwise to the acetone solution (150–200 mL) of the corresponding pyridine derivative (0.1 mol). The mixture was stirred under argon for 2–3 days (progress of alkylation was controlled by TLC). After completion, the formed precipitate was filtered, washed with acetone and dried in vacuum at room temperature (heating caused decomposition). The obtained pyridinium salt was used without further purification or characterization.

General Procedures for the synthesis of compounds 3–8.

Procedure (A):

In a 25 mL Schlenk flask, under argon flow, 2.0 mmol of diketone, 1.0 mmol of the appropriate pyridinium salt, and 1.0 mmol (138 mg) of K_2CO_3 were loaded. The flask was covered with a septum stopper and 7 mL of absolute CH_3CN was added by syringe. The reaction mixture was left under intensive stirring at room temperature for 24 hours. Then the solvent was removed under reduced pressure and the crude material was subjected to column chromatography.

Procedure (B):

In the case of the 3-cyanopyrdinium salt $NaHCO_3$ (2.0 mmol, 168 mg) was used as a base and the reaction mixture was left over 4 days.

Procedure (C):

In the case of 2-cyanopyridinium salts the reaction was completed within 1 hour.

List of pyridinium salts used

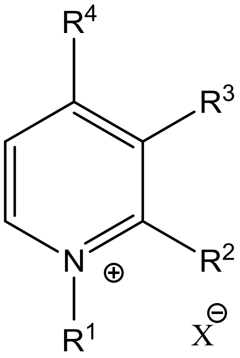

Figure S1

Table 1

Entry	2	R^1	R^2	R^3	R^4	X^-	Known	Reactivity
1	**2a**	Me	H	COMe	H	I	Yes	Yes
2	**2b**	Me	H	COPh	H	I	Yes	Yes
3	**2c**	Me	H	CO_2Et	H	I	Yes	Yes
4	**2d**	Me	H	CN	H	I	Yes	Yes
5	**2e**	Et	H	COMe	H	Br	Yes	Yes
6	**2f**	Et	H	COPh	H	Br	No	Yes
7	**2g**	Allyl	H	COMe	H	Br	No	Yes
8	**2h**	Allyl	H	COPh	H	Br	Yes	Yes
9	**2i**	Allyl	H	CN	H	Br	No	Yes
10	**2j**	Bn	H	COMe	H	Br	Yes	Yes
11	**2k**	Bn	H	COPh	H	Br	No	Yes
12	**2l**	Bn	H	CN	H	Br	Yes	Yes
13	**2m**	Me	CN	H	H	I	Yes	Yes
14	**2n**	Allyl	CN	H	H	Br	No	Yes
15	**2o**	Bn	CN	H	H	Br	Yes	Yes
16	**2p**	Me	H	H	H	I	Yes	No
17	**2q**	Me	COMe	H	H	I	Yes	No
18	**2r**	Me	Me	H	H	I	Yes	No
19	**2s**	Me	H	CHO	H	I	Yes	No
20	**2t**	Me	H	NO_2	H	I	Yes	No
21	**2u**	Me	H	Me	H	I	Yes	No
22	**2v**	Me	H	H	CN	I	Yes	No
23	**2w**	Me	H	H	COMe	I	Yes	No
24	**2x**	Propargyl	CN	H	H	Br	No	No
25	**2y**	Propargyl	H	CN	H	Br	No	No

NMR, HRMS and IR spectral data

12-Acetyl-5,5,10-trimethyl-8-oxa-10-aza-tricyclo[7.3.1.0^{2,7}]trideca-2(7),11-dien-3-one (3a)

Following General Procedure (A); yellow solid, mp 120–122 °C, yield 85% (233 mg). ^{1}H NMR (300 MHz, $CDCl_3$) δ = 0.98 (s, 3H, Me), 0.99 (s, 3H, Me), 1.67 (dt, 1H, ^2J = 13.1 Hz, ^3J = 2.5 Hz, CHCH_2CH), 1.85 (dt, 1H, ^2J = 13.4 Hz, ^3J = 2.6 Hz, CHCH_2CH), 2.14 (s, 3H, COMe), 2.19 (d, 2H, ^2J = 3.4 Hz, CCH_2), 2.25 (d, 2H, ^2J = 8.0 Hz, CCH_2), 3.24 (s, 3H, NMe), 4.45-4.46 (m, 1H, COCC*H*), 5.61 (s, 1H, NC*H*O), 7.35 (s, 1H, MeNC*H*C). ^{13}C NMR (62.9 MHz, $CDCl_3$): δ = 24.3 (CH), 25.6 (CH_2), 27.3, 29.1 (CH_3), 32.2 (C), 41.9 (CH_2), 42.7, 44.6 (CH_3), 50.4 (CH_2), 65.7 (CH), 109.4, 111.3 (C), 149.0 (CH), 172.5, 192.0, 196.9 (C). MS (GC, 70 eV): *m/z* (%) = 275 (M^+, 55), 260 (100), 232 (20), 139 (94). HRMS (EI): calcd for $C_{16}H_{21}NO_3$ (M^+) 275.15160, found 275.151294. IR (ATR, cm^{-1}): $\tilde{\nu}$ = 2958 (w), 1637 (m), 1592 (s), 1567 (s), 1415 (w), 1380 (s), 1351 (m), 1325 (s), 1305 (s), 1210 (m), 1169 (s), 1121 (s), 1058 (m), 1035 (s), 1008 (m), 964 (m), 935 (m), 919 (m), 840 (s), 801 (m), 770 (w), 712 (m), 684 (w), 613 (s), 593 (m).

12-Benzoyl-5,5,10-trimethyl-8-oxa-10-aza-tricyclo[7.3.1.0^{2,7}]trideca-2(7),11-dien-3-one (3b)

Following General Procedure (A); light orange solid, mp 117–118 °C, yield 72% (243 mg). ^{1}H NMR (300 MHz, $CDCl_3$) δ = 1.02 (s, 6H, 2 x Me), 1.81 (m, 2H, CHCH_2CH), 2.22 (d, 2H, ^2J = 3.3 Hz, CCH_2), 2.34 (d, 2H, ^2J = 5.2 Hz, CCH_2), 3.19 (s, 3H, NMe), 4.50 (s, 1H, COCC*H*), 5.83 (q, 1H, ^3J = 2.5 Hz, NC*H*O), 7.15 (s, 1H, MeNC*H*C), 7.35-7.46 (m, 5H, CH_{Ar}). ^{13}C NMR (75.5 MHz, $CDCl_3$): δ = 25.5 (CH_2), 27.7, 28.7 (CH_3), 32.4 (C), 42.0 (CH_2), 42.9, 44.9 (CH), 50.4 (CH_2), 65.8 (CH_3), 108.7, 111.2 (C), 128.0, 128.1, 129.8 (CH, Ar), 140.5 (C), 152.3 (CH), 172.8, 191.6, 196.8 (C). MS (GC, 70 eV): *m/z* (%) = 337 (M^+, 45), 322 (59), 198 (100), 105 (28), 83 (24). HRMS (ESI): calcd for $C_{21}H_{24}NO_3$ (M + H) 338.17507, found 338.17489. IR (ATR, cm^{-1}): $\tilde{\nu}$ = 2952 (w), 1631 (m), 1615 (m), 1588 (s), 1563 (s), 1444 (w), 1379 (s), 1326 (s), 1307 (s), 1229 (w), 1212 (s), 1197 (m), 1128 (s), 1047 (s), 1026 (s), 979 (m), 964 (m), 896 (m), 842 (m), 829 (m), 801 (m), 782 (m), 766 (m), 742 (s), 699 (s), 670 (s), 644 (s), 611 (s), 573 (m).

5,5,10-Trimethyl-3-oxo-8-oxa-10-aza-tricyclo[7.3.1.0^{2,7}]trideca-2(7),11-diene-12-carboxylic acid ethyl ester (3c)

Following General Procedure (A); light orange solid, mp 107–109 °C, yield 90% (275 mg). ^{1}H NMR (300 MHz, $CDCl_3$) δ = 0.97 (s, 3H, CH_3), 0.99 (s, 3H, CH_3), 1.24 (t, 3H, ^3J = 7.0 Hz, OCH_2CH_3), 1.64-1.84 (m, 2H, CHCH_2CH), 2.10-2.34 (m, 4H, CCH_2), 3.17 (s, 3H, NMe), 4.14 (m, 2H, OC$H_2$$CH_3$), 4.41-4.42 (m, 1H, CC*H*C), 5.46 (q, 1H, ^3J = 2.8 Hz, NC*H*O), 7.41 (s, 1H, MeNC*H*C). ^{13}C NMR (62.9 MHz, $CDCl_3$): δ = 14.6 (CH_3), 25.6 (CH_2), 27.2, 29.1, (CH_3), 32.2 (C), 41.9 (CH_2), 42.3, 44.3 (CH), 50.4, 59.2 (CH_2), 67.0 (NCH_3), 96.2, 111.3 (C), 148.1 (CH), 167.1, 172.3 (C). MS (GC, 70 eV): *m/z* (%) = 305 (M^+, 51), 290 (100), 276 (22), 262 (16), 232 (12), 166 (96), 138 (27). HRMS (ESI): calcd for $C_{17}H_{24}NO_4$ (M + H) 306.16998, found 306.16976.

IR (ATR, cm^{-1}): $\tilde{\nu}$ = 2961 (w), 1668 (m), 1603 (s), 1495 (w), 1445 (w), 1376 (s), 1326 (m), 1291 (s), 1272 (s), 1211 (m), 1165 (s), 1130 (m), 1099 (m), 1069 (s), 1028 (s), 978 (m), 944 (m), 908 (m), 835 (m), 802 (m), 771 (m), 698 (m), 657 (m), 609 (m).

5,5,10-Trimethyl-3-oxo-8-oxa-10-aza-tricyclo[7.3.1.0^{2,7}]trideca-2(7),11-diene-12-carbonitrile (3d)

Following General Procedure (A); yellow solid, mp 139–141 °C, yield 63% (163 mg).
^{1}H NMR (300 MHz, $CDCl_3$) δ = 1.01 (s, 6H, 2 x Me), 1.71-1.87 (m, 2H, CHC*H$_2$*CH), 2.22 (dd, 4H, ^2J = 10.0 Hz, ^4J = 1.8 Hz, CC*H$_2$*), 3.08 (s, 3H, NMe), 3.68-3.69 (m, 1H, CNCC*H*), 5.21 (q, 1H, ^3J = 2.3 Hz, NC*H*O), 6.67 (s, 1H, MeNC*H*C). ^{13}C NMR (62.9 MHz, $CDCl_3$): δ = 20.4 (CH), 25.2 (CH_2), 27.7, 28.8 (CH_3), 32.1 (C), 40.4, 41.5 (CH_2), 50.1 (CH_3), 81.8 (CH), 84.2, 115.6, 119.9 (C), 145.1 (CH), 167.1, 195.2 (C). MS (GC, 70 eV): *m/z* (%) = 258 (M^+, 79), 241 (52), 174 (56), 159 (14), 146 (49), 119 (100). HRMS (ESI): calcd for $C_{15}H_{19}N_2O_2$ (M + H) 259.1441, found 259.14452. IR (ATR, cm^{-1}): $\tilde{\nu}$ = 2925 (w), 2184 (w), 1649 (m), 1618 (s), 1470 (w), 1440 (w), 1413 (w), 1387 (s), 1331 (s), 1231 (w), 1203 (m), 1114 (m), 1090 (m), 1035 (s), 979 (m), 904 (w), 866 (w), 826 (m), 792 (m), 769 (m), 721 (m), 649 (w), 622 (m), 577 (m).

12-Acetyl-10-ethyl-5,5-dimethyl-8-oxa-10-aza-tricyclo[7.3.1.0^{2,7}]trideca-2(7),11-dien-3-one (3e)

Following General Procedure (A); yellow solid, mp 88–90 °C, yield 57% (165 mg).
^{1}H NMR (300 MHz, $CDCl_3$) δ = 0.98 (s, 6H, 2 x Me), 1.26 (t, 3H, ^3J = 7.3 Hz, NCH$_2$C*H$_3$*), 1.58-1.63 (m, 1H, CHC*H$_2$*CH), 1.88 (dt, 1H, ^2J = 13.3 Hz, ^3J = 3.0 Hz, CHC*H$_2$*CH), 2.16 (s, 3H, COMe), 2.17-2.27 (m, 4H, CC*H$_2$*), 3.24-3.36 (m, 1H, NC*H$_2$*CH$_3$), 3.72-3.83 (m, 1H, NC*H$_2$*CH$_3$), 4.56-4.57 (m, 1H, COCC*H*), 5.62 (s, 1H, NC*H*O), 7.42 (s, 1H, MeNC*H*C). ^{13}C NMR (62.9 MHz, $CDCl_3$): δ = 15.2 (CH_3), 24.4 (CH), 26.2 (CH_2), 27.3, 29.1 (CH_3), 32.2 (C), 42.0 (CH_2), 42.8 (CH_3), 49.9, 50.4 (CH_2), 66.0 (CH), 109.4, 111.4 (C), 147.8 (CH), 172.4, 192.1, 196.9 (C). MS (GC, 70 eV): *m/z* (%) = 289 (M^+, 71), 260 (100), 246 (31), 204 (27), 150 (64). HRMS (EI): calcd for $C_{17}H_{23}NO_3$ (M^+) 289.16725, found 289.166641. IR (ATR, cm^{-1}): $\tilde{\nu}$ = 2961 (w), 2873 (w), 1642 (w), 1596 (s), 1574 (s), 1428 (w), 1380 (s), 1348 (s), 1302 (s), 1258 (s), 1222 (m), 1207 (m), 1163 (s), 1111 (m), 1033 (s), 981 (m), 946 (s), 931 (m), 917 (m), 836 (s), 800 (m), 766 (m), 710 (m), 681 (m), 611 (m), 591 (m), 562 (m).

12-Benzoyl-10-ethyl-5,5-dimethyl-8-oxa-10-aza-tricyclo[7.3.1.0^{2,7}]trideca-2(7),11-dien-3-one (3f)

Following General Procedure (A); pale orange solid, mp 147–149 °C, yield 64% (225 mg).
^{1}H NMR (300 MHz, $CDCl_3$) δ = 1.02 (s, 6H, 2 x Me), 1.19 (t, 3H, ^3J = 7.1 Hz, NCH$_2$C*H$_3$*), 1.67 (dt, 1H, ^2J = 13.3 Hz, ^3J = 2.4 Hz, CHC*H$_2$*CH), 1.95 (dt, 1H, ^2J = 13.3 Hz, ^3J = 2.6 Hz, CHC*H$_2$*CH), 2.21 (d, 2H, ^2J = 2.2 Hz, CC*H$_2$*), 2.33 (d, 2H, ^2J = 3.7 Hz, CC*H$_2$*), 3.17-3.79 (m, 2H, NC*H$_2$*CH$_3$), 4.61 (s, 1H, COCC*H*), 5.84 (br. q, 1H, ^3J = 2.0 Hz, NC*H*O), 7.23 (s, 1H, MeNC*H*C), 7.34-7.47 (m, 5H, CH_{Ar}). ^{13}C NMR (75.5 MHz, $CDCl_3$): δ = 15.1 (CH_3), 26.2 (CH_2), 27.7, 28.7 (CH_3), 32.4 (C), 42.1 (CH_2), 43.1 (CH), 50.0, 50.5 (CH_2), 66.1 (CH), 108.7, 111.4 (C), 128.0, 128.1, 129.8 (CH, Ar), 140.6 (C), 151.0 (CH), 172.7, 191.6, 196.8 (C). MS

(GC, 70 eV): *m/z* (%) = 351 (M^+, 38), 322 (56), 246 (15), 212 (100), 184 (26), 105 (72). HRMS (ESI): calcd for $C_{22}H_{26}NO_3$ (M + H) 352.19072, found 352.19132. IR (ATR, cm^{-1}): $\tilde{\nu}$ = 2945 (w), 1639 (m), 1592 (s), 1565 (s), 1443 (w), 1402 (m), 1379 (s), 1363 (s), 1316 (m), 1286 (m), 1260 (s), 1229 (m), 1211 (m), 1191 (m), 1130 (s), 1035 (s), 980 (m), 946 (m), 897 (m), 834 (s), 800 (m), 765 (m), 746 (m), 705 (s), 669 (m), 647 (m), 615 (m), 566 (m).

12-Acetyl-10-allyl-5,5-dimethyl-8-oxa-10-aza-tricyclo[7.3.1.0^{2,7}]trideca-2(7),11-dien-3-one (3g)

Following General Procedure (A); yellow solid, mp 106–108 °C, yield 65% (196 mg).
^{1}H NMR (300 MHz, $CDCl_3$) δ = 0.99 (s, 3H, Me), 1.00 (s, 3H, Me), 1.64 (dt, 1H, ^2J = 13.3 Hz, ^3J = 2.5 Hz, CHC*H*$_2$CH), 1.88 (dt, 1H, ^2J = 13.3 Hz, ^3J = 2.8 Hz, CHC*H*$_2$CH), 2.16 (s, 3H, COMe), 2.18-2.27 (m, 4H, CC*H*$_2$), 3.82-3.89 (m, 1H, NC*H*$_2$CHCH$_2$), 4.30-4.38 (m, 1H, NC*H*$_2$CHCH$_2$), 4.53-4.54 (m, 1H, COCC*H*), 5.25-5.37 (m, 2H, NCH$_2$CHC*H*$_2$), 5.64 (s, 1H, NC*H*O), 5.75-5.88 (m, 1H, NCH$_2$C*H*CH$_2$), 7.40 (s, 1H, NC*H*CCO). ^{13}C NMR (75.5 MHz, $CDCl_3$): δ = 26.0 (CH_2), 27.3 (CH_3), 29.1 (CH), 28.2 (CH_3), 32.3 (C), 41.9 (CH_2), 43.0 (CH_3), 50.4, 57.5 (CH_2), 66.0 (CH), 109.9, 111.5 (C), 119.0 (CH_2), 133.2, 148.2 (CH), 172.5, 192.5, 196.9 (C). MS (GC, 70 eV): *m/z* (%) = 301 (M^+, 81), 260 (73), 204 (29), 162 (69), 43 (100). HRMS (EI): calcd for $C_{18}H_{23}NO_3$ (M^+) 301.16725, found 301.166587. IR (ATR, cm^{-1}): $\tilde{\nu}$ = 2955 (w), 1644 (w), 1600 (s), 1571 (s), 1451 (w), 1421 (m), 1380 (s), 1353 (s), 1303 (m), 1238 (m), 1208 (s), 1160 (m), 1116 (s), 1033 (m), 956 (m), 937 (m), 915 (s), 837 (m), 774 (w), 715 (m), 609 (m).

10-Allyl-12-benzoyl-5,5-dimethyl-8-oxa-10-aza-tricyclo[7.3.1.0^{2,7}]trideca-2(7),11-dien-3-one (3h)

Following General Procedure (A); pale orange solid, mp 106–108 °C, yield 62% (225 mg).
^{1}H NMR (300 MHz, $CDCl_3$) δ = 1.01 (s, 3H, Me), 1.02 (s, 3H, Me), 1.69 (dt, 1H, ^2J = 13.6 Hz, ^3J = 2.6 Hz, CHC*H*$_2$CH), 1.94 (dt, 1H, ^2J = 13.4 Hz, ^3J = 2.9 Hz, CHC*H*$_2$CH), 2.21 (d, 2H, ^2J = 2.1 Hz, CC*H*$_2$), 2.32 (d, 2H, ^2J = 4.0 Hz, CC*H*$_2$), 3.72-3.80 (m, 1H, NC*H*$_2$CHCH$_2$), 4.27-4.35 (m, 1H, NC*H*$_2$CHCH$_2$), 4.57 (q, 1H, ^4J = 2.1 Hz, CC*H*CH$_2$), 5.20-5.32 (m, 2H, NCH$_2$CHC*H*$_2$), 5.67-5.81 (m, 1H, NCH$_2$C*H*CH$_2$), 5.85 (q, 1H, ^4J = 2.6 Hz, NCHO), 7.21 (s, 1H, NCHC), 7.34-7.46 (m, 5H, Ph). ^{13}C NMR (75.5 MHz, $CDCl_3$): δ = 25.9 (CH_2), 27.6, 28.7 (CH_3), 32.3 (C), 42.0 (CH_2), 43.1 (CH), 50.4, 57.6 (CH_2), 66.0 (CH), 108.9, 111.4 (C), 119.0 (CH_2), 128.0, 128.1, 130.0, 133.0 (CH), 140 4 (C), 151.4 (CH), 172.7, 191.7, 196.8 (C). MS (GC, 70 eV): *m/z* (%) = 363 (M^+, 63), 322 (36), 258 (45), 224 (51), 105 (100). HRMS (ESI): calcd for $C_{23}H_{26}NO_3$ (M + H) 364.19072, found 364.19069. IR (ATR, cm^{-1}): $\tilde{\nu}$ = 2959 (w), 1738 (w), 1640 (m), 1597 (s), 1566 (s), 1445 (w), 1416 (m), 1378 (s), 1349 (s), 1327 (m), 1220 (s), 1180 (m), 1134 (m), 1104 (s), 1034 (s), 992 (m), 949 (m), 935 (m), 888 (m), 842 (m), 807 (w), 783 (m), 741 (m), 699 (s), 646 (s), 622 (m).

10-Allyl-5,5-dimethyl-3-oxo-8-oxa-10-aza-tricyclo[7.3.1.0^{2,7}]trideca-2(7),11-diene-12-carbonitrile (3i)

Following General Procedure (B); yellow solid, mp 125–127 °C, yield 72% (204 mg).

^{1}H NMR (250 MHz, $CDCl_3$) δ = 1.01 (s, 3H, Me), 1.02 (s, 3H, Me), 1.70-1.91 (m, 2H, CHC*H₂*CH), 2.21 (s, 2H, CC*H₂*), 2.22 (s, 2H, CC*H₂*), 3.70-3.71 (m, 1H, CC*H*CH₂), 3.77-3.98 (m, 2H, NC*H₂*CHCH₂), 5.15-5.32 (m, 3H, NCH₂CHC*H₂*, NC*H*O), 5.70-5.86 (m, 1H, NCH₂C*H*CH₂), 6.72 (s, 1H, NC*H*CCN). ^{13}C NMR (62.9 MHz, $CDCl_3$): δ = 20.7 (CH), 25.4 (CH_2), 27.8, 28.8 (CH_3), 32.1 (C), 41.6, 50.1, 55.9 (CH_2), 80.6 (CH), 84.7, 115.5, 118.7 (CH_2), 119.9 (C), 133.0, 144.3 (CH), 167.2, 195.4 (C). MS (GC, 70 eV): *m/z* (%) = 284 (M^+, 54), 267 (27), 243 (100), 200 (27), 172 (24), 145 (54), 104 (15), 83 (31). HRMS (ESI): calcd for $C_{17}H_{21}N_2O_2$ (M + H) 285.15975, found 285.16023. IR (ATR, cm^{-1}): $\tilde{\nu}$ = 2956 (w), 2925 (w), 2193 (m), 1623 (s), 1449 (w), 1383 (s), 1285 (w), 1235 (m), 1208 (m), 1106 (m), 1044 (s), 1033 (s), 994 (w), 948 (m), 922 (m), 837 (m), 794 (w), 728 (w), 668 (w), 604 (w).

12-Acetyl-10-benzyl-5,5-dimethyl-8-oxa-10-aza-tricyclo[7.3.1.0^{2,7}]trideca-2(7),11-dien-3-one (3j)

Following General Procedure (A); orange solid, mp 156–158 °C, yield 73% (324 mg).
^{1}H NMR (300 MHz, $CDCl_3$) δ = 0.98 (s, 3H, Me), 1.01 (s, 3H, Me), 1.50 (dt, 1H, ^2J = 13.5 Hz, ^3J = 2.5 Hz, CHC*H₂*CH), 1.78 (dt, 1H, ^2J = 13.3 Hz, ^3J = 3.0 Hz, CHC*H₂*CH), 2.17 (s, 3H, CH_3), 2.19-2.28 (m, 4H, CC*H₂*), 4.39 (d, 1H, ^2J = 14.8 Hz, PhCH₂), 4.48 (s, 1H, OCCC*H*), 4.90 (d, 1H, ^2J = 14.8 Hz, PhCH₂), 5.62 (s, 1H, NC*H*O), 7.28-7.37 (m, 5H, CH_{Ar}), 7.54 (s, 1H, NCHC). ^{13}C NMR (62.9 MHz, $CDCl_3$): δ = 24.4 (CH_3), 26.0 (CH_2), 27.2, 29.2 (CH_3), 32.3 (C), 42.0 (CH_2), 42.5 (CH), 50.4, 58.9 (CH_2), 66.0 (CH), 109.7, 111.4 (C), 128.0, 128.1, 128.9 (Ar), 136.6 (C), 148.4 (CH), 172.6, 192.4, 197.1 (C). MS (GC, 70 eV): *m/z* (%) = 351 (M^+, 41), 308 (20), 260 (96), 204 (14), 106 (18), 91 (100). HRMS (EI): calcd for $C_{22}H_{25}NO_3$ (M^+) 351.18290, found 351.183121. IR (ATR, cm^{-1}): $\tilde{\nu}$ = 2957 (w), 1642 (w), 1597 (s), 1573 (s), 1431 (w), 1382 (s), 1351 (s), 1300 (m), 1207 (s), 1154 (m), 1115 (s), 1046 (s), 1033 (s), 981 (w), 936 (m), 918 (m), 838 (m), 799 (w), 750 (w), 699 (m), 603 (m).

12-Benzoyl-10-benzyl-5,5-dimethyl-8-oxa-10-aza-tricyclo[7.3.1.0^{2,7}]trideca-2(7),11-dien-3-one (3k)

Following General Procedure (A); orange solid, mp 156–158 °C, yield 73% (324 mg).
^{1}H NMR (250 MHz, $CDCl_3$) δ = 1.02 (s, 3H, Me), 1.05 (s, 3H, Me), 1.55 (dt, 1H, ^2J = 13.5 Hz, ^3J = 2.4 Hz, CHC*H₂*CH), 1.85 (dt, 1H, ^2J = 13.5 Hz, ^3J = 2.3 Hz, CHC*H₂*CH), 2.25 (d, 2H, ^2J = 5.3 Hz, CC*H₂*), 2.34 (d, 2H, ^2J = 6.8 Hz, CC*H₂*), 4.32 (d, 1H, ^2J = 14.5 Hz, NCH₂), 4.52-4.53 (m, 1H, OCCC*H*), 4.87 (d, 1H, ^2J = 14.5 Hz, NCH₂), 5.84 (q, 1H, ^3J = 2.4 Hz, NC*H*O), 7.26-7.47 (m, 11H, NCHC, CH_{Ar}). ^{13}C NMR (62.9 MHz, $CDCl_3$): δ = 25.9 (CH_2), 27.6, 28.8 (CH_3), 32.4 (C), 42.0 (CH_2), 42.7 (CH), 50.4, 59.0 (CH_2), 66.0 (CH), 108.8, 111.3 (C), 128.0, 128.0, 128.1, 128.3, 129.9 (CH, Ar), 136.3, 140.4 (C), 151.7 (CH), 172.9, 191.8, 197.0 (C). MS (GC, 70 eV): *m/z* (%) = 413 (M^+, 53), 322 (75), 308 (22), 275 (16), 183 (31), 105 (79), 91 (100). HRMS (ESI): calcd for $C_{27}H_{28}NO_3$ (M + H) 414.20637, found 414.20648. IR (ATR, cm^{-1}): $\tilde{\nu}$ = 2928 (w), 1644 (w), 1598 (s), 1557 (s), 1443 (w), 1378 (s), 1303 (m), 1210 (s), 1107 (m), 1047 (m), 1021 (m), 956 (w), 890 (w), 831 (w), 791 (w), 727 (m), 697 (s), 617 (m).

10-Benzyl-5,5-dimethyl-3-oxo-8-oxa-10-aza-tricyclo[7.3.1.0^{2,7}]trideca-2(7),11-diene-12-carbonitrile (3l)

Following General Procedure (B); light pink solid, mp 139–141 °C, yield 70% (234 mg). ^{1}H NMR (250 MHz, $CDCl_3$) δ = 0.98-1.00 (m, 6H, 2 x Me), 1.71-1.88 (m, 2H, CHC*H$_2$*CH), 2.05-2.19 (m, 4H, 2 x CC*H$_2$*), 3.71 (s, 1H, CNCC*H*), 4.35-4.55 (m, 2H, NCH_2), 5.31 (s, 1H, NC*H*O), 6.83 (s, 1H NC*H*CCN), 7.19-7.33 (m, 5H, CH_{Ar}). ^{13}C NMR (62.9 MHz, $CDCl_3$): δ = 20.7 (CH), 25.4 (CH_2), 27.9, 28.6 (CH_3), 32.0 (C), 41.4, 50.0, 57.3 (CH_2), 80.6 (CH), 84.8, 115.4, 119.9 (C), 127.6, 128.2, 128.9 (CH, Ar), 136.3 (C), 144.7 (CH), 167.2, 195.4 (C). MS (GC, 70 eV): *m/z* (%) = 334 (M^+, 31), 243 (100), 222 (12), 91 (96). HRMS (ESI): calcd for $C_{21}H_{23}N_2O_2$ (M + H) 335.1754, found 335.17541. IR (ATR, cm^{-1}): $\tilde{\nu}$ = 2956 (w), 2188 (m), 1615 (s), 1467 (w), 1414 (m), 1383 (s), 1357 (m), 1318 (m), 1290 (w), 1223 (m), 1179 (m), 1105 (m), 1046 (s), 1024 (m), 979 (m), 947 (m), 844 (m), 794 (w), 755 (m), 696 (s), 668 (m), 645 (m), 613 (m).

5,5,10-Trimethyl-3-oxo-8-oxa-10-aza-tricyclo[7.3.1.0^{2,7}]trideca-2(7),11-diene-11-carbonitrile (4a)

Following General Procedure (C); yellow solid, mp 84–86 °C, yield 82% (212 mg). ^{1}H NMR (300 MHz, $CDCl_3$) δ = 1.01 (s, 3H, Me), 1.02 (s, 3H, Me), 1.81 (t, 2H, ^3J = 2.6 Hz, CHC*H$_2$*CH), 2.18 (s, 2H, CC*H$_2$*), 2.26 (s, 2H, CC*H$_2$*), 3.04 (s, 3H, NMe), 3.55-3.58 (m, 1H, CC*H*CH), 5.29 (q, 1H, ^3J = 2.2 Hz, NC*H*O), 5.88 (d, 1H, ^3J = 7.1 Hz, NCCC*H*). ^{13}C NMR (62.9 MHz, $CDCl_3$): δ = 19.9 (CH), 25.0 (CH_2), 27.8, 28.6 (CH_3), 32.1 (C), 37.5 (CH_3), 41.8, 50.2 (CH_2), 83.6 (CH), 114.9, 115.0, 118.8 (C), 119.6 (CH), 169.2, 195.6 (C). MS (GC, 70 eV): *m/z* (%) = 258 (M^+, 44), 243 (28), 201 (11), 119 (100). HRMS (ESI): calcd for $C_{15}H_{19}N_2O_2$ (M + H) 259.1441, found 259.14439. IR (ATR, cm^{-1}): $\tilde{\nu}$ = 2968 (w), 2878 (w), 2226 (w), 1649 (s), 1620 (s), 1450 (w), 1407 (w), 1379 (s), 1342 (m), 1322 (s), 1278 (m), 1231 (w), 1202 (w), 1172 (m), 1155 (m), 1105 (m), 1075 (m), 1052 (s), 1030 (m), 980 (s), 917 (w), 901 (w), 830 (s), 815 (m), 761 (s), 605 (s), 593 (s).

10-Allyl-5,5-dimethyl-3-oxo-8-oxa-10-aza-tricyclo[7.3.1.0^{2,7}]trideca-2(7),11-diene-11-carbonitrile (4b)

Following General Procedure (C); yellow solid, mp 95–97 °C, yield 77% (219 mg). ^{1}H NMR (300 MHz, $CDCl_3$) δ = 1.01 (s, 3H, Me), 1.03 (s, 3H, Me), 1.72-1.88 (m, 2H, CHC*H$_2$*CH), 2.19 (s, 2H, CC*H$_2$*), 2.25 (s, 2H, CC*H$_2$*), 3.56-3.59 (m, 1H, CC*H*CH), 3.95-4.00 (m, 2H, NC*H$_2$*CHCH$_2$), 5.20-5.28 (m, 2H, NCH$_2$CHC*H$_2$*), 5.39 (m, 1H, NC*H*O), 5.75-5.86 (m, 1H, NCH$_2$C*H*CH$_2$), 5.90 (dd, 1H, ^3J = 7.4 Hz, ^4J = 1.4 Hz, NCCC*H*). ^{13}C NMR (62.9 MHz, $CDCl_3$): δ = 20.0 (CH), 25.1 (CH_2), 28.0, 28.6 (CH_3), 32.1 (C), 41.8, 50.2, 53.1 (CH_2), 81.9 (CH), 114.7, 115.0, 118.1 (C), 118.5 (CH_2), 119.6, 133.5 (CH), 168.9, 195.7 (C). MS (GC, 70 eV): *m/z* (%) = 284 (M^+, 11), 243 (100), 145 (10). HRMS (ESI): calcd for $C_{17}H_{21}N_2O_2$ (M + H) 285.15975, found 285.1594. IR (ATR, cm^{-1}): $\tilde{\nu}$ = 2957 (w), 2874 (w), 2225 (w), 1643 (m), 1617 (s), 1455 (w), 1420 (m), 1382 (s), 1368 (m), 1344 (m), 1327 (w), 1290 (m), 1213 (w), 1185 (w), 1167 (m), 1118 (m), 1105 (m), 1046 (m), 1009 (w), 990 (m), 962 (m), 916 (m), 891 (w), 833 (m), 819 (m), 791 (m), 767 (m), 654 (m), 611 (m), 594 (m).

10-Benzyl-5,5-dimethyl-3-oxo-8-oxa-10-aza-tricyclo[7.3.1.0^{2,7}]trideca-2(7),11-diene-11-carbonitrile (4c)

Following General Procedure (C); pale yellow solid, mp 129–131 °C, yield 67% (224 mg).
^{1}H NMR (300 MHz, $CDCl_3$) δ = 0.97 (s, 3H, Me), 1.00 (s, 3H, Me), 1.69-1.83 (m, 2H, CHC*H*$_2$CH), 2.04-2.25 (m, 4H, 2 x CC*H*$_2$), 3.58-3.60 (m, 1H, CC*H*CH), 4.48-4.60 (m, 2H, NCH_2), 5.30 (q, 1H, ^3J = 2.6 Hz, NC*H*O), 5.93 (dd, 1H, ^3J = 7.2 Hz, ^4J = 1.1 Hz, NCCC*H*), 7.27-7.38 (m, 5H, CH_{Ar}). ^{13}C NMR (75.5 MHz, $CDCl_3$): δ = 20.1 (CH), 25.1 (CH_2), 27.9, 28.6 (CH_3), 32.0 (C), 41.6, 50.2, 53.9 (CH_2), 81.2 (CH), 114.5, 115.2, 118.6 (C), 119.5 (CH), 127.8, 128.0, 128.7 (CH, Ar), 137.0, 168.9, 195.7 (C). MS (GC, 70 eV): *m/z* (%) = 334 (M^+, 7), 243 (100), 91 (81). HRMS (ESI): calcd for $C_{21}H_{23}N_2O_2$ (M + H) 335.1754, found 335.17563. IR (ATR, cm^{-1}): $\tilde{\nu}$ = 2955 (w), 2868 (w), 2223 (w), 1650 (s), 1618 (s), 1495 (w), 1449 (w), 1418 (w), 1382 (s), 1282 (w), 1199 (m), 1107 (s), 1055 (m), 1031 (m), 1002 (m), 964 (m), 917 (w), 835 (m), 770 (m), 729 (s), 696 (s), 591 (m).

5,5,12-Trimethyl-3-oxo-8-oxa-12-aza-tricyclo[7.3.1.0^{2,7}]trideca-2(7),10-diene-10-carbonitrile (5)

Following General Procedure (A); yellow solid, mp 117–119 °C, yield 20% (52 mg).
^{1}H NMR (300 MHz, $CDCl_3$) δ = 1.00 (s, 3H, Me), 1.03 (s, 3H, Me),1.70-1.86 (m, 2H, CHC*H*$_2$CH), 2.20-2.38 (m, 4H, CC*H*$_2$), 3.16 (s, 3H, NMe), 4.44-4.45 (m, 1H, NC*H*C), 5.02 (q, 1H, ^3J = 2.7 Hz, NCCC*H*O), 6.89 (s, 1H, MeNC*H*C). ^{13}C NMR (62.9 MHz, $CDCl_3$): δ = 25.2 (CH_2), 27.4, 28.9 (CH_3), 32.3 (C), 41.7 (CH_2), 42.4 (CH_3), 44.0 (CH), 50.4 (CH_2), 84.2, 68.1 (CH), 75.0, 111.0, 121.7 (C), 149.4 (CH), 171.5, 196.9 (C). MS (GC, 70 eV): *m/z* (%) = 258 (M^+, 41), 243 (100), 119 (71). HRMS (ESI): calcd for $C_{15}H_{19}N_2O_2$ (M + H) 259.1441, found 259.14412. IR (ATR, cm^{-1}): $\tilde{\nu}$ = 2961 (w), 2187 (s), 1642 (m), 1612 (s), 1467 (w), 1414 (w), 1391 (s), 1381 (s), 1336 (s), 1319 (m), 1206 (m), 1163 (w), 1122 (s), 1082 (w), 1038 (s), 981 (m), 958 (m), 922 (w), 843 (m), 798 (w), 772 (w), 709 (m), 681 (m), 605 (m), 570 (m).

12-Acetyl-10-methyl-8-oxa-10-aza-tricyclo[7.3.1.0^{2,7}]trideca-2(7),11-dien-3-one (6a)

Following General Procedure (A); yellow solid, mp 108–110 °C, yield 90% (222 mg).
^{1}H NMR (300 MHz, $CDCl_3$) δ = 1.65-1.93 (m, 4H, C*H*$_2$), 2.13 (s, 3H, COMe), 2.28-2.40 (m, 4H, CC*H*$_2$), 3.24 (s, 3H, NMe), 4.42-4.43 (m, 1H, COCC*H*), 5.60 (s, 1H, NC*H*O), 7.34 (s, 1H, MeNC*H*C). ^{13}C NMR (75.5 MHz, $CDCl_3$): δ = 20.6 (CH_2), 24.2 (CH), 25.5, 28.3, 36.4 (CH_2), 42.8, 44.7 (CH_3), 65.6 (CH), 109.2, 112.5 (C), 149.2 (CH), 174.0, 192.0, 197.2 (C). MS (GC, 70 eV): *m/z* (%) = 247 (M^+, 67), 232 (79), 204 (24), 136 (100). HRMS (EI): calcd for $C_{14}H_{17}NO_3$ (M^+) 247.12029, found 247.120569. IR (ATR, cm^{-1}): $\tilde{\nu}$ = 2939 (w), 1636 (m), 1593 (s), 1574 (s), 1415 (m), 1381 (s), 1341 (s), 1298 (m), 1172 (s), 1126 (s), 1065 (m), 1025 (s), 971 (m), 958 (m), 933 (m), 915 (m), 860 (m), 826 (s), 713 (m), 867 (m), 601 (m).

12-Benzoyl-10-methyl-8-oxa-10-aza-tricyclo[7.3.1.0^{2,7}]trideca-2(7),11-dien-3-one (6b)

Following General Procedure (A); yellow solid, mp 108–110 °C, yield 75% (232 mg).
^{1}H NMR (300 MHz, $CDCl_3$) δ = 1.70-2.02 (m, 4H, 2 x CH_2), 2.35 (t, 2H, ^3J = 6.3 Hz, C*H*$_2$CH$_2$CH$_2$), 2.46 (t, 2H, ^3J = 6.3 Hz, CH$_2$CH$_2$C*H*$_2$), 3.20 (s, 3H, NMe), 4.49 (br. q, 1H,

^3J = 2.1 Hz, COCC*H*), 5.81 (br. q, 1H, ^3J = 2.3 Hz, NC*H*O), 7.16 (s, 1H, MeNC*H*), 7.35-7.47 (m, 5H, CH_{Ar}). ^{13}C NMR (62.9 MHz, $CDCl_3$): δ = 20.7, 25.4, 28.4, 36.5 (CH_2), 42.9, 44.9 (CH), 65.7 (CH_3), 108.4, 112.4 (C), 128.0, 128.1, 129.7 (CH, Ar), 140.4 (C), 152.4 (CH), 174.3, 191.6, 197.1 (C). MS (GC, 70 eV): *m/z* (%) = 309 (M^+, 25), 294 (25), 198 (100), 105 (18). HRMS (ESI): calcd for $C_{19}H_{20}NO_3$ (M + H) 310.14377, found 310.14336. IR (ATR, cm^{-1}): $\tilde{\nu}$ = 2943 (w), 1592 (s), 1556 (s), 1413 (m), 1376 (s), 1329 (s), 1306 (s), 1198 (m), 1181 (m), 1124 (s), 1032 (s), 968 (m), 909 (m), 895 (m), 838 (m), 816 (m), 793 (w), 724 (s), 699 (s), 644 (m), 592 (m).

10-Methyl-3-oxo-8-oxa-10-aza-tricyclo[7.3.1.0^{2,7}]trideca-2(7),11-diene-12-carboxylic acid ethyl ester (6c)

Following General Procedure (A); light orange solid, mp 99–101 °C, yield 88% (244 mg).
^1H NMR (300 MHz, $CDCl_3$) δ = 1.25 (t, 3H, ^3J = 7.1 Hz, OCH_2CH_3), 1.63-1.94 (m, 4H, C*H*$_2$), 2.29-2.42 (m, 4H, CC*H*$_2$), 3.19 (s, 3H, NMe), 4.15 (m, 2H, OC*H*$_2$CH$_3$), 4.41-4.42 (m, 1H, COCC*H*), 5.46-5.49 (m, 1H, NC*H*O), 7.43 (s, 1H, MeNC*H*C). ^{13}C NMR (63 MHz, $CDCl_3$): δ = 14.6 (CH_3), 20.7, 25.6, 28.4, 36.5 (CH_2), 42.5, 44.5 (CH), 59.2 (CH_2), 66.9 (CH_3), 96.1, 112.6 (C), 148.2 (CH), 167.2, 173.9, 197.3 (C). MS (GC, 70 eV): *m/z* (%) = 277 (M^+, 59), 262 (81), 248 (23), 232 (20), 166 (100), 138 (37). HRMS (EI): calcd for $C_{15}H_{19}NO_4$ (M^+) 277.13086, found 277.131066. IR (ATR, cm^{-1}): $\tilde{\nu}$ = 3451 (w), 2941 (w), 1976 (m), 1643 (m), 1603 (s), 1388 (s), 1376 (s), 1325 (m), 1293 (s), 1277 (s), 1221 (w), 1163 (s), 1125 (s), 1066 (s), 1032 (s), 963 (m), 859 (w), 822 (m), 769 (m), 695 (w).

10-Methyl-3-oxo-8-oxa-10-aza-tricyclo[7.3.1.0^{2,7}]trideca-2(7),11-diene-12-carbonitrile (6d)

Following General Procedure (A); light pink solid, mp 150–152 °C, yield 61% (140 mg).
^1H NMR (300 MHz, $CDCl_3$) δ = 1.79-1.95 (m, 4H, 2 x CH_2), 2.30-2.38 (m, 4H, 2 x CH_2), 3.08 (s, 3H, NMe), 3.66 (s, 1H, CNCC*H*), 5.21 (s, 1H, NC*H*O), 6.67 (s, 1H, MeNC*H*). ^{13}C NMR (62.9 MHz, $CDCl_3$): δ = 20.4 (CH), 20.5, 25.1, 27.7, 36.1 (CH_2), 40.4 (CH_3), 81.7 (CH), 84.0, 116.8, 120.0 (C), 145.2 (CH), 168.7, 1956 (C). MS (GC, 70 eV): *m/z* (%) = 230 (M^+, 53), 213 (43), 174 (46), 146 (29), 119 (100). HRMS (EI): calcd for $C_{13}H_{14}N_2O_2$ (M^+) 230.104526, found 230.10498. IR (ATR, cm^{-1}): $\tilde{\nu}$ = 2952 (w), 2187 (m), 1651 (m), 1622 (s), 1491 (w), 1434 (w), 1384 (s), 1331 (m), 1316 (m), 1288 (w), 1219 (w), 1190 (m), 1120 (s), 1094 (m), 1057 (m), 1028 (s), 966 (s), 930 (m), 911 (m), 870 (m), 811 (s), 744 (m), 723 (m), 661 (m), 622 (s), 593 (m), 573 (s).

12-Acetyl-10-ethyl-8-oxa-10-aza-tricyclo[7.3.1.0^{2,7}]trideca-2(7),11-dien-3-one (6e)

Following General Procedure (A); yellow solid, mp 82–84 °C, yield 74% (193 mg).
^1H NMR (250 MHz, $CDCl_3$) δ = 1.26 (t, 3H, ^3J = 7.0 Hz, NCH_2CH_3), 1.56-1.94 (m, 1H, CHC*H*$_2$CH), 1.85-1.94 (m, 3H, CHC*H*$_2$CH, CH_2), 2.16 (s, 3H, COMe), 2.28-2.41 (m, 4H, 2 x C*H*$_2$), 3.23-3.38 (m, 1H, NC*H*$_2$CH$_3$), 3.74-3.88 (m, 1H, NC*H*$_2$CH$_3$), 4.55 (s, 1H, COCC*H*), 5.62 (s, 1H, NC*H*O), 7.42 (s, 1H, MeNC*H*C). ^{13}C NMR (62.9 MHz, $CDCl_3$): δ = 15.2 (CH_3), 20.7 (CH_2), 24.4 (CH), 26.1, 28.3, 36.5 (CH_2), 42.9 (CH_3), 49.9 (CH_2), 66.2 (CH), 100.4, 112.6 (C), 147.6 (CH), 173.9, 192.0, 197.2 (C). MS (GC, 70 eV): *m/z* (%) = 261 (M^+, 63), 232 (100), 218 (26), 190 (11), 176 (20), 150 (50), 122 (16). HRMS (ESI): calcd for

$C_{15}H_{20}NO_3$ (M + H) 262.14377, found 262.14352. IR (ATR, cm^{-1}): $\tilde{\nu}$ = 2932 (w), 1640 (w), 1593 (s), 1573 (s), 1427 (w), 1380 (s), 1350 (s), 1301 (m), 1263 (m), 1220 (m), 1194 (m), 1163 (s), 1128 (m), 1063 (m), 1027 (s), 974 (m), 930 (m), 913 (m), 858 (w), 819 (m), 732 (w), 711 (w), 684 (w), 607 (m).

12-Benzoyl-10-ethyl-8-oxa-10-aza-tricyclo[7.3.1.0^{2,7}]trideca-2(7),11-dien-3-one (6f)

Following General Procedure (A); yellow solid, mp 135–137 °C, yield 45% (145 mg).
^{1}H NMR (300 MHz, $CDCl_3$) δ = 1.19 (t, 3H, ^3J = 7.2 Hz, NCH_2CH_3), 1.62-1.68 (m, 1H, CH_2), 1.90-1.98 (m,3H, CH_2), 2.32-2.36 (m, 2H, CH_2), 2.43-2.47 (m, 2H, CH_2), 3.16-3.27 (m, 1H, NCH_2CH_3), 3.71-3.83 (m, 1H, NCH_2CH_3), 4.58-4.59 (m, 1H, COCC*H*), 5.82 (br. q, 1H, ^4J = 2.9 Hz, NC*H*O), 7.24 (s, 1H, EtNC*H*), 7.35-7.47 (m, 5H, Ph). ^{13}C NMR (62.9 MHz, $CDCl_3$): δ = 15.0 (CH_3), 20.7, 26.0, 28.4, 36.5 (CH_2), 43.0 (CH), 50.0 (CH_2), 65.9 (CH_3), 108.4, 112.5 (C), 128.0, 128.1, 129.7 (Ph), 140.5 (C), 151.1 (CH), 174.2, 191.5, 197.1 (C). MS (GC, 70 eV): *m/z* (%) = 323 (M^+, 67), 294 (84), 212 (100), 184 (24), 105 (92). HRMS (EI): calcd for $C_{20}H_{21}NO_3$ (M^+) 323.15160, found 323.151205. IR (ATR, cm^{-1}): $\tilde{\nu}$ = 2935 (w), 1735 (w), 1643 (w), 1592 (s), 1557 (s), 1443 (w), 1427 (w), 1377 (s), 1357 (m), 1304 (m), 1261 (s), 1223 (m), 1196 (m), 1131 (s), 1102 (m), 1061 (m), 1030 (s), 971 (m), 901 (m), 859 (m), 812 (s), 795 (m), 728 (s), 699 (s), 655 (s).

12-Acetyl-10-allyl-8-oxa-10-aza-tricyclo[7.3.1.0^{2,7}]trideca-2(7),11-dien-3-one (6g)

Following General Procedure (A); yellow solid, mp 97–99 °C, yield 58% (158 mg).
^{1}H NMR (300 MHz, $CDCl_3$) δ = 1.58-1.93 (m, 4H, CH_2), 2.15 (s, 3H, COMe), 2.28-2.40 (m, 4H, 2 x CH_2), 3.82-3.89 (m, 1H, NCH_2CHCH_2), 4.32-4.51 (m, 1H, NCH_2CHCH_2), 4.50-4.51 (m, 1H, COCC*H*), 5.24-5.35 (m, 2H, NCH_2CHCH_2), 5.62 (s, 1H, NC*H*O), 5.74-5.87 (m, 1H, NCH_2CHCH_2), 7.39 (s, 1H, NC*H*CCO). ^{13}C NMR (62.9 MHz, $CDCl_3$): δ = 20.6 (CH_2), 24.3 (CH), 25.9, 28.3, 36.4 (CH_2), 43.1 (CH_3), 57.6 (CH_2), 65.7 (CH), 109.6, 112.7 (C), 118.8 (CH_2), 133.2, 148.3 (CH), 174.0, 192.3, 197.2 (C). MS (GC, 70 eV): *m/z* (%) = 273 (M^+, 100), 232 (71), 176 (19), 162 (45), 135 (18). HRMS (EI): calcd for $C_{16}H_{19}NO_3$ (M^+) 273.13594, found 273.135508. IR (ATR, cm^{-1}): $\tilde{\nu}$ = 2937(w), 1638 (m), 1595 (s), 1567 (s), 1435 (m), 1380 (s), 1351 (s), 1301 (m), 1236 (m), 1213 (s), 1182 (s), 1154 (s), 1115 (s), 1066 (m), 1028 (s), 1001 (m), 971 (m), 939 (s), 911 (s), 864 (m), 823 (s), 761 (m), 661 (m).

10-Allyl-12-benzoyl-8-oxa-10-aza-tricyclo[7.3.1.0^{2,7}]trideca-2(7),11-dien-3-one (6h)

Following General Procedure (A); yellow solid, mp 97–99 °C, yield 50% (168 mg).
^{1}H NMR (300 MHz, $CDCl_3$) δ = 1.67-1.73 (m, 1H, CH_2), 1.90-1.99 (m, 3H, 2 x CH_2), 2.34-2.50 (m, 4H, 2 x CH_2), 3.74-3.81 (m, 1H, NCH_2CHCH_2), 4.32-4.39 (m, 1H, NCH_2CHCH_2), 4.58-4.59 (m, 1H, $CCHCH_2$), 5.21-5.34 (m, 2H, NCH_2CHCH_2), 5.70-5.87 (m, 2H, NCH_2CHCH_2, NC*H*O), 7.24 (s, 1H, NCHC), 7.34-7.49 (m, 5H, CH_{Ar}). ^{13}C NMR (62.9 MHz, $CDCl_3$): δ = 20.7, 25.8, 28.4, 36.5 (CH_2), 43.1 (CH), 57.6 (CH_2), 65.9 (CH), 108.8, 112.6 (C), 118.9 (CH_2), 128.0, 128.1, 129.8 (CH, Ar), 133.0 (CH), 140.3 (C), 151.6 (CH), 174.2, 191.7, 197.2 (C). MS (GC, 70 eV): *m/z* (%) = 335 (M^+, 82), 294 (43), 224 (59), 184 (23), 105 (100). HRMS (ESI): calcd for $C_{21}H_{22}NO_3$ (M + H) 336.15942, found 336.15971. IR (ATR, cm^{-1}): $\tilde{\nu}$ = 2942 (w), 2240 (w), 1640 (w), 1592 (s), 1556 (s), 1427 (w), 1377 (s), 1344 (m),

1305 (m), 1218 (s), 1196 (m), 1119 (m), 1102 (m), 1062 (w), 1032 (s), 962 (w), 916 (m), 860 (w), 818 (m), 724 (s), 700 (s), 643 (m).

10-Allyl-3-oxo-8-oxa-10-aza-tricyclo[7.3.1.0^{2,7}]trideca-2(7),11-diene-12-carbonitrile (6i)
Following General Procedure (B); yellow solid, mp 118–120 °C, yield 75% (192 mg).
^{1}H NMR (250 MHz, $CDCl_3$) δ = 1.68-1.96 (m, 4H, 2 x CH_2), 2.30-2.38 (m, 4H, 2 x CH_2), 3.69-3.70 (m, 1H, NCCC*H*), 3.77-3.98 (m, 2H, NCH_2CHCH_2), 5.14-5.32 (m, 3H, NCH_2CHCH_2, NC*H*O), 5.70-5.86 (m, 1H, NCH_2C*H*CH_2), 6.72 (s, 1H, NC*H*C). ^{13}C NMR (62.9 MHz, $CDCl_3$): δ = 20.5 (CH), 20.8, 25.3, 27.7, 36.1, 55.8 (CH_2), 80.3 (CH), 84.6, 116.7 (C), 118.6 (CH_2), 120 (C), 133.0, 144.4 (CH), 168.8, 195.6 (C). MS (GC, 70 eV): *m/z* (%) = 256 (M^+, 37), 239 (24), 215 (100), 200 (15), 172 (17), 145 (26). HRMS (ESI): calcd for $C_{15}H_{17}N_2O_2$ (M + H) 257.12845, found 257.12873. IR (ATR, cm^{-1}): $\tilde{\nu}$ = 2943 (w), 2193 (m), 1625 (s), 1603 (s), 1415 (m), 1385 (s), 1319 (m), 1284 (w), 1232 (m), 1197 (s), 1122 (m), 1060 (m), 1027 (s), 972 (m), 936 (s), 913 (s), 870 (m), 820 (s), 740 (m), 681 (m), 659 (m), 624 (m).

12-Acetyl-10-benzyl-8-oxa-10-aza-tricyclo[7.3.1.0^{2,7}]trideca-2(7),11-dien-3-one (6j)
Following General Procedure (A); yellow solid, mp 58–60 °C, yield 50% (162 mg).
^{1}H NMR (300 MHz, $CDCl_3$) δ = 1.46-1.52 (m, 1H, C*H$_2$*), 1.79 (t, 1H, ^{3}J = 7.2 Hz, C*H$_2$*), 1.85-1.97 (m, 2H, C*H$_2$*), 2.18 (s, 3H, CH_3), 2.23-2.42 (m, 4H, 2 x C*H$_2$*), 4.40 (d, 1H, ^{2}J = 14.8 Hz, CH_2Ph), 4.46-4.47 (m, 1H, COCC*H*), 4.93 (d, 1H, ^{2}J = 14.8 Hz, CH_2Ph), 5.61 (s, 1H, NC*H*O), 7.30-7.37 (m, 5H, CH_{Ar}), 7.54 (s, 1H, NC*H*C). ^{13}C NMR (62.9 MHz, DMSO): δ = 20.4 (CH_2), 24.3 (CH), 25.4, 27.6, 35.9 (CH_2), 41.6 (CH_3), 57.4 (CH_2), 65.0 (CH), 108.9, 112.2 (C), 127.6, 127.7, 128.7 (CH, Ar), 137.5 (c), 149.5 (CH), 173.2, 190.7, 196.2 (C). MS (GC, 70 eV): *m/z* (%) = 323 (M^+, 36), 301 (33), 260 (30), 232 (72), 176 (13), 162 (36), 106 (28), 91 (93), 41 (100). HRMS (EI): calcd for $C_{20}H_{21}NO_3$ (M^+) 323.1516, found 323.151743. IR (ATR, cm^{-1}): $\tilde{\nu}$ = 2938 (w), 1640 (w), 1595 (s), 1573 (s), 1428 (w), 1382 (s), 1352 (s), 1300 (m), 1194 (s), 1153 (m), 1119 (m), 1062 (m), 1028 (s), 971 (m), 932 (m), 914 (m), 859 (w), 821 (m), 740 (m), 699 (m), 602 (m).

12-Benzoyl-10-benzyl-8-oxa-10-aza-tricyclo[7.3.1.0^{2,7}]trideca-2(7),11-dien-3-one (6k)
Following General Procedure (A); orange solid, mp 153–155 °C, yield 55% (212 mg).
^{1}H NMR (300 MHz, $CDCl_3$) δ = 1.55 (dt, 1H, ^{2}J = 13.4 Hz, ^{3}J = 2.7 Hz, CHC*H$_2$*CH), 1.86 (dt, 1H, ^{2}J = 13.4 Hz, ^{3}J = 2.8 Hz, CHC*H$_2$*CH), 1.92-2.01 (m, 2H, CH_2C*H$_2$*CH_2), 2.38 (t, 2H, ^{3}J = 7.1 Hz, C*H$_2$*), 2.48 (t, 2H, ^{3}J = 6.4 Hz, C*H$_2$*), 4.31 (d, 1H, ^{2}J = 14.4 Hz, NCH_2), 4.51-4.52 (m, 1H, OCCC*H*), 4.90 (d, 1H, ^{2}J = 14.4 Hz, NCH_2), 5.83 (q, 1H, ^{3}J = 2.7 Hz, NC*H*O), 7.27-7.50 (m, 11H, NCHC, CH_{Ar}). ^{13}C NMR (62.9 MHz, $CDCl_3$): δ = 20.8, 25.9, 28.4, 36.5 (CH_2), 42.8 (CH), 59.1 (CH_2), 65.9 (CH), 108.7, 112.6 (C), 127.9, 128.0, 128.1, 128.2, 128.9, 129.9 (CH, Ar), 136.4, 140.4 (C), 151.8 (CH), 174.4, 191.8, 197.3 (C). MS (GC, 70 eV): *m/z* (%) = 385 (M^+, 43), 294 (50), 280 (15), 183 (30), 105 (62), 91 (100). HRMS (ESI): calcd for $C_{25}H_{24}NO_3$ (M + H) 386.17507, found 386.17549. IR (ATR, cm^{-1}): $\tilde{\nu}$ = 2945 (w), 1738 (w), 1636 (w), 1592 (s), 1556 (s), 1493 (w), 1431 (m), 1377 (s), 1346 (s), 1305 (m), 1232 (m),

1220 (s), 1196 (m), 1113 (s), 1064 (m), 1033 (s), 993 (m), 970 (m), 914 (m), 888 (m), 842 (m), 820 (m), 800 (m), 728 (s), 704 (s), 658 (m).

10-Benzyl-3-oxo-8-oxa-10-aza-tricyclo[7.3.1.0^{2,7}]trideca-2(7),11-diene-12-carbonitrile (6l)
Following General Procedure (B); light pink solid, mp 145–147 °C, yield 64% (196 mg).
^{1}H NMR (250 MHz, $CDCl_3$) δ = 1.69-1.95 (m, 4H, 2 x CH_2), 2.16-2.36 (m, 4H, 2 x CH_2), 3.71 (s, 1H, CNCC*H*), 4.35-4.56 (m, 2H, NCH_2), 5.28-5.31 (m, 1H, NC*H*O), 6.84 (s, 1H NC*H*CCN), 7.16-7.19 (m, 2H, CH_{Ar}), 7.31-7.36 (m, 3H, CH_{Ar}). ^{13}C NMR (62.9 MHz, $CDCl_3$): δ = 20.5 (CH), 20.9, 25.4, 27.6, 36.2, 57.2 (CH_2), 80.4 (CH), 85.0, 116.7, 119.9 (C), 127.5, 128.2, 128.9 (CH, Ar), 136.3 (C), 144.7 (CH), 168.7, 195.6 (C). MS (GC, 70 eV): *m/z* (%) = 306 (M^+, 19), 222 (12), 215 (73), 91 (100). HRMS (ESI): calcd for $C_{19}H_{19}N_2O_2$ (M + H) 307.1441, found 307.14387. IR (ATR, cm^{-1}): $\tilde{\nu}$ = 2976 (w), 2193 (m), 1625 (s), 1448 (w), 1420 (m), 1386 (s), 1315 (w), 1222 (w), 1195 (m), 1117 (m), 1063 (m), 1034 (s), 982 (m), 957 (w), 916 (m), 872 (w), 827 (s), 756 (m), 740 (m), 699 (s), 633 (s), 588 (m).

10-Methyl-3-oxo-8-oxa-10-aza-tricyclo[7.3.1.0^{2,7}]trideca-2(7),11-diene-11-carbonitrile (7a)
Following General Procedure (C); pale yellow solid, mp 116–117 °C, yield 68% (156 mg).
^{1}H NMR (300 MHz, $CDCl_3$) δ = 1.79-1.80 (m, 2H, CHCH_2CH), 1.93 (quintet, 2H, ^3J = 6.7 Hz, CH_2C$H_2$$CH_2$), 2.29-2.34 (m, 2H, CH_2), 2.39 (t, 2H, ^3J = 6.0 Hz, CH_2), 3.05 (s, 3H, NMe), 3.55-3.58 (m, 1H, CC*H*CH), 5.29 (q, 1H, ^3J = 2.3 Hz, NC*H*O), 5.87 (d, 1H, ^3J = 7.2 Hz, NCCC*H*). ^{13}C NMR (62.9 MHz, $CDCl_3$): δ = 20.0 (CH), 20.6, 25.0, 27.9, 36.2 (CH_2), 37.6 (CH_3), 83.6 (CH), 115.0, 116.3, 118.8 (C), 119.6 (CH), 170.8, 195.9 (C). MS (GC, 70 eV): *m/z* (%) = 230 (M^+, 70), 215 (29), 174 (10), 119 (100). HRMS (ESI): calcd for $C_{13}H_{15}N_2O_2$ (M + H) 231.1128, found 231.1133. IR (ATR, cm^{-1}): $\tilde{\nu}$ = 2950 (w), 2227 (w), 1636 (s), 1614 (s), 1597 (w), 1470 (w), 1433 (w), 1381 (m), 1326 (m), 1284 (w), 1235 (w), 1201 (w), 1171 (m), 1114 (m), 1081 (m), 1047 (m), 1019 (m), 978 (m), 951 (w), 912 (m), 810 (m), 767 (s), 711 (m), 605 (m), 589 (s).

10-Allyl-3-oxo-8-oxa-10-aza-tricyclo[7.3.1.0^{2,7}]trideca-2(7),11-diene-11-carbonitrile (7b)
Following General Procedure (C); pale yellow solid, mp 90–92 °C, yield 62% (160 mg).
^{1}H NMR (300 MHz, $CDCl_3$) δ = 1.70-1.87 (m, 2H, CH_2), 1.92 (quintet, 2H, ^3J = 6.4 Hz, CH_2C$H_2$$CH_2$), 2.29-2.40 (m, 4H, 2 x CH_2), 3.56-3.59 (m, 1H, CC*H*CH), 3.97-3.99 (m, 2H, NCH_2CHCH_2), 5.20-5.27 (m, 2H, NCH_2CHCH_2), 5.37-5.39 (m, 1H, NC*H*O), 5.75-5.86 (m, 1H, NCH_2C*H*CH_2), 5.89 (dd, 1H, ^3J = 7.2 Hz, ^4J = 1.3 Hz, NCCC*H*). ^{13}C NMR (75.5 MHz, $CDCl_3$): δ = 20.2 (CH), 20.6, 25.0, 28.0, 36.2, 52.9 (CH_2), 81.7 (CH), 115.0, 116.0, 118.1 (C), 118.3 (CH_2), 119.4, 133.5 (CH), 170.5, 196.0 (C). MS (GC, 70 eV): *m/z* (%) = 256 (M^+, 13), 215 (100), 145 (12). HRMS (ESI): calcd for $C_{15}H_{17}N_2O_2$ (M + H) 257.12845, found 257.12833. IR (ATR, cm^{-1}): $\tilde{\nu}$ = 2942 (w), 2226 (w), 1644 (s), 1614 (s), 1446 (w), 1432 (w), 1383 (s), 1328 (m), 1285 (m), 1236 (w), 1195 (m), 1162 (m), 1141 (m), 1104 (m), 1048 (m), 1025 (m), 967 (m), 934 (s), 914 (s), 808 (s), 790 (s), 770 (s), 630 (m), 606 (m), 587 (s).

10-Benzyl-3-oxo-8-oxa-10-aza-tricyclo[7.3.1.0^{2,7}]trideca-2(7),11-diene-11-carbonitrile (7c)
Following General Procedure (C); yellow solid, mp 127–128 °C, yield 74% (227 mg).
^{1}H NMR (300 MHz, $CDCl_3$) δ 1.69-1.93 (m, 4H, 2 x CH_2), 2.11-2.37 (m, 4H, 2 x CH_2), 3.57-3.60 (m, 1H, CC*H*CH), 4.49-4.61 (m, 2H, NCH_2), 5.30 (q, 1H, ^3J = 2.6 Hz, NC*H*O), 5.95 (dd, 1H, ^3J = 7.4 Hz, ^4J = 1.1 Hz, NCCC*H*), 7.25-7.38 (m, 5H, CH_{Ar}). ^{13}C NMR (75.5 MHz, $CDCl_3$): δ = 20.2 (CH), 20.6, 25.1, 27.8, 36.2, 53.9 (CH_2), 81.1 (CH), 115.2, 115.6, 118.7 (C), 119.6 (CH), 127.8, 127.9, 128.7 (CH, Ar), 137.2, 170.5, 196.0 (C). MS (GC, 70 eV): *m/z* (%) = 306 (M^+, 8), 215 (100), 91 (82). HRMS (ESI): calcd for $C_{19}H_{19}N_2O_2$ (M + H) 307.1441, found 307.14459. IR (ATR, cm^{-1}): $\tilde{\nu}$ = 2928 (w), 2224 (w), 1650 (m), 1616 (s), 1495 (w), 1450 (w), 1418 (m), 1384 (m), 1358 (w), 1282 (w), 1192 (w), 1170 (w), 1123 (m), 1060 (m), 1026 (m), 999 (m), 968 (m), 942 (w), 913 (m), 813 (m), 785 (m), 736 (s), 694 (s), 607 (m), 589 (m).

12-Methyl-3-oxo-8-oxa-12-aza-tricyclo[7.3.1.0^{2,7}]trideca-2(7),10-diene-10-carbonitrile (8)
Following General Procedure (A); yellow solid, mp 95–97 °C, yield 32% (74 mg).
^{1}H NMR (300 MHz, $CDCl_3$) δ = 1.64-2.02 (m, 4H, 2 x C*H*$_2$), 2.32-2.47 (m, 4H, C*H*$_2$), 3.18 (s, 3H, NMe), 4.43-4.44 (m, 1H, NC*H*C), 5.02 (q, 1H, ^3J = 2.8 Hz, NCCC*H*O), 6.90 (s, 1H, MeNC*H*C). ^{13}C NMR (62.9 MHz, $CDCl_3$): δ = 20.6, 25.5, 28.1, 36.4 (CH_2), 68.0 (CH_3), 74.8, 112.3, 121.7 (C), 149.5 (CH), 173.0, 197.2 (C). MS (GC, 70 eV): *m/z* (%) = 230 (M^+, 61), 215 (100), 119 (63). HRMS (EI): calcd for $C_{13}H_{14}N_2O_2$ (M^+) 230.10498, found 230.104576. IR (ATR, cm^{-1}): $\tilde{\nu}$ = 2936 (w), 2184 (s), 1645 (m), 1612 (s), 1455 (w), 1411 (m), 1389 (s), 1331 (s), 1240 (w), 1196 (w), 1181 (m), 1125 (s), 1060 (m), 1025 (s), 960 (m), 914 (m), 856 (m), 825 (s), 746 (m), 712 (m), 675 (m), 647 (m).

Copies of ^{1}H and ^{13}C NMR spectra for compounds 3, 4, 5, 6, 7 and 8

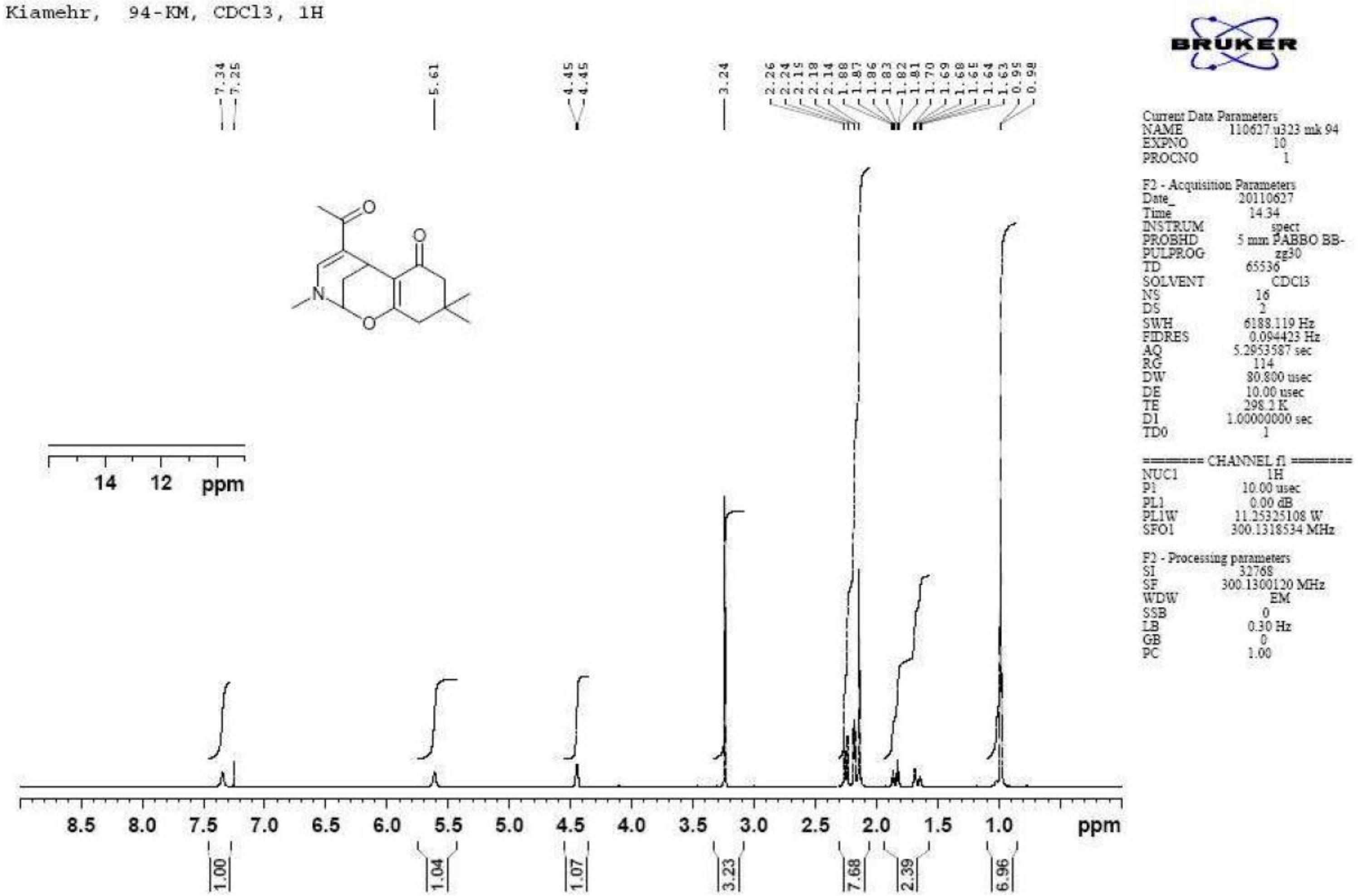

^{1}H NMR spectra for compound 3a

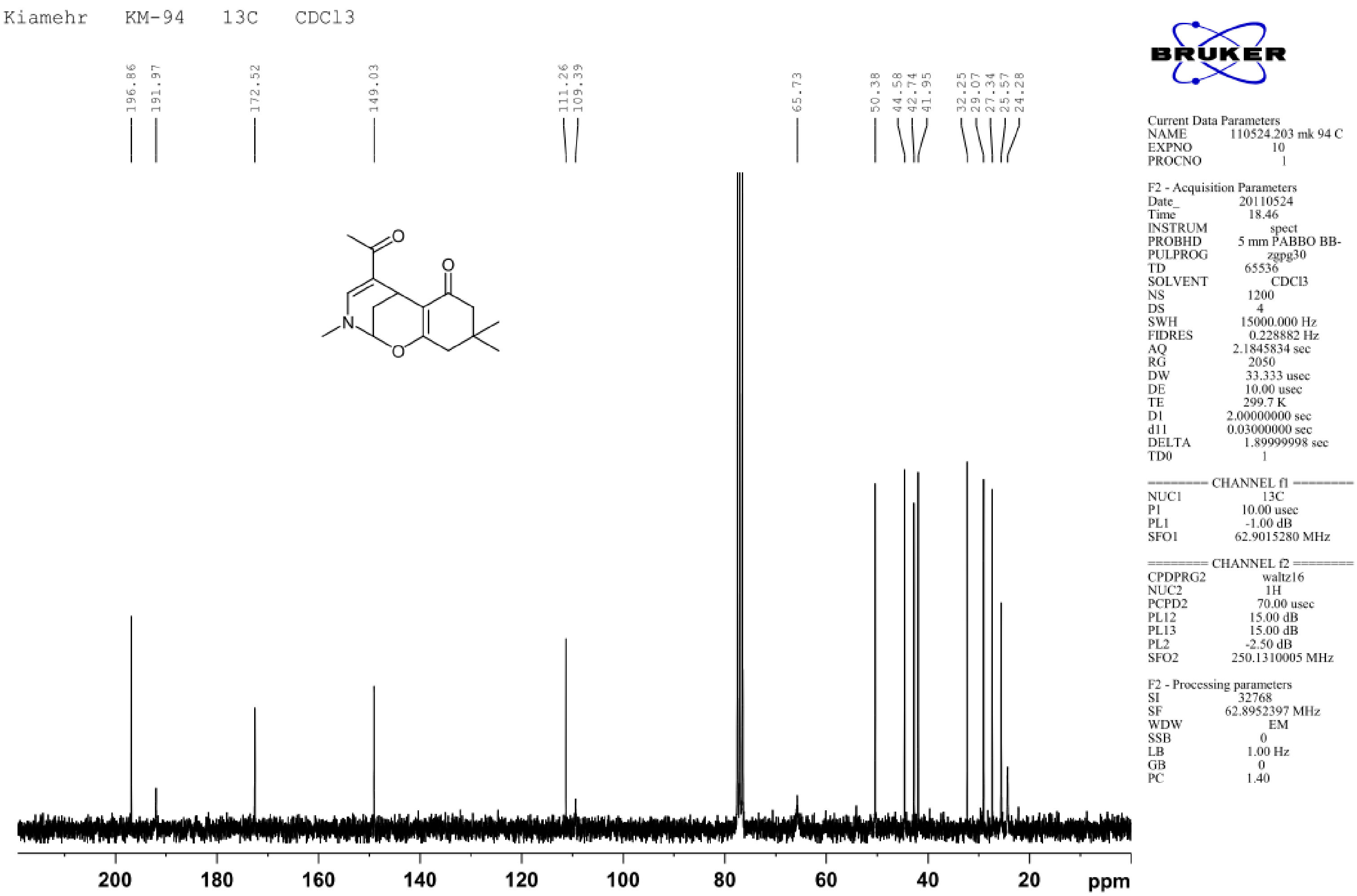

^{13}C NMR spectra for compound 3a

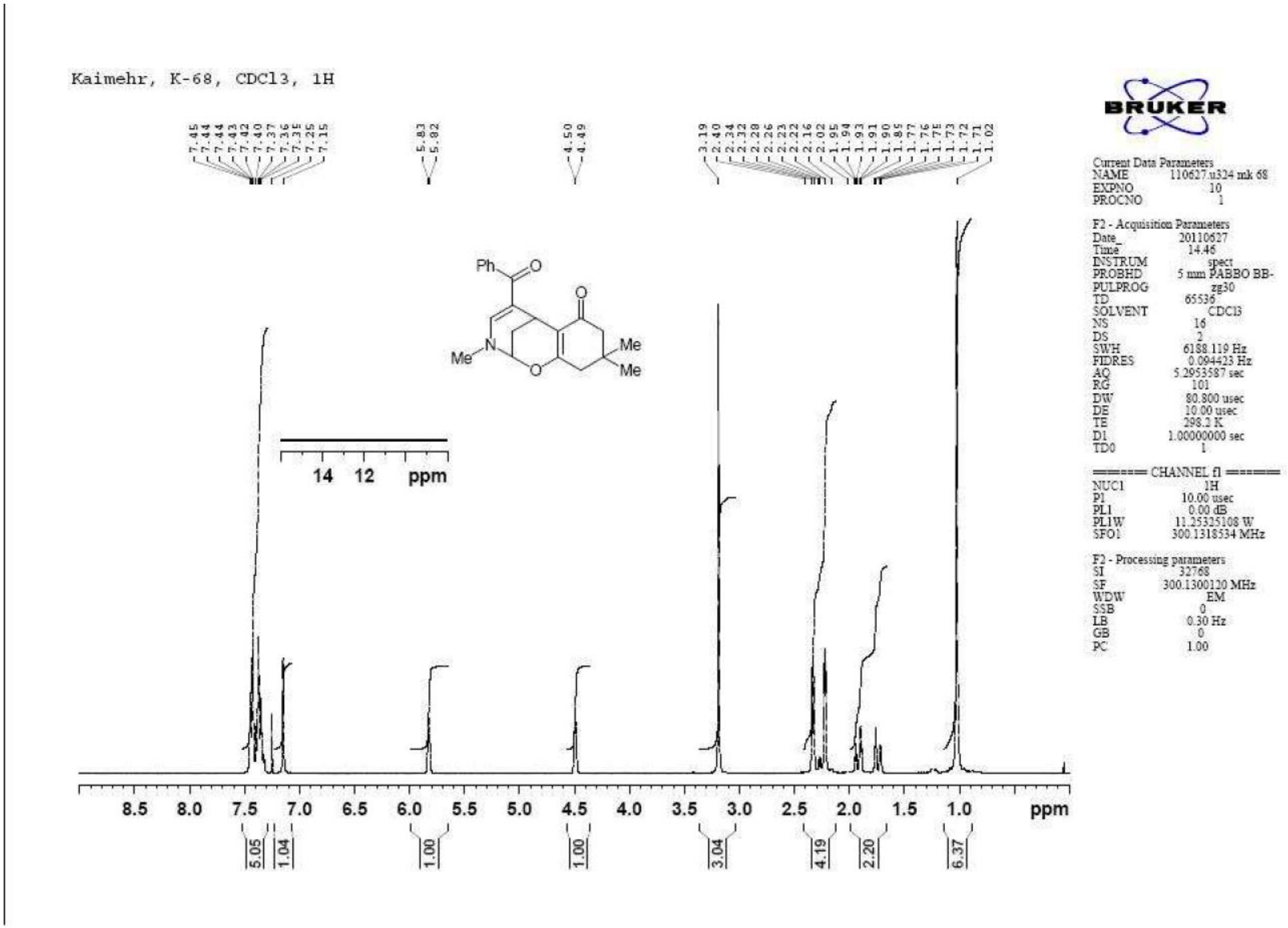

^{1}H NMR spectra for compound 3b

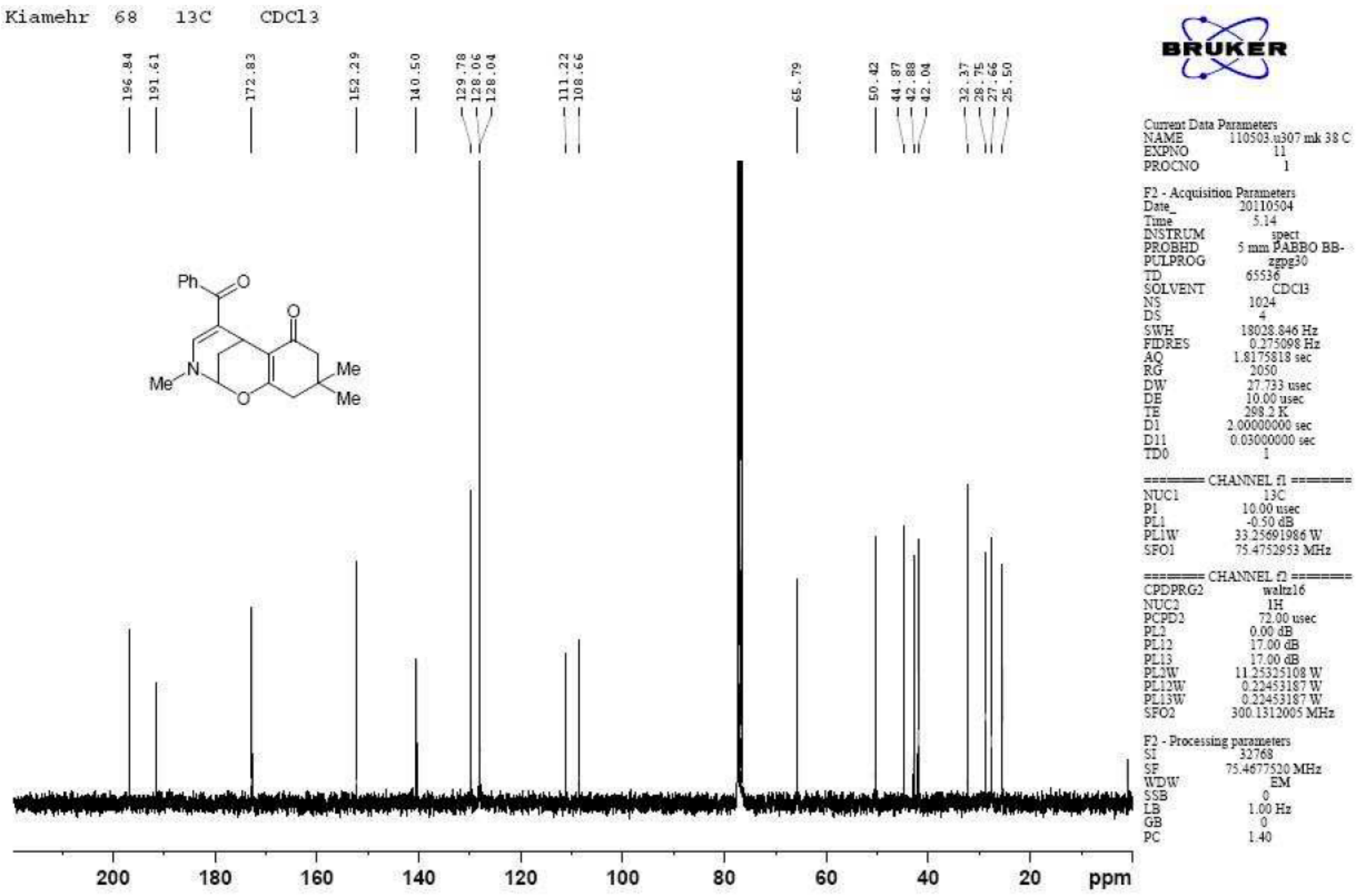

^{13}C NMR spectra for compound 3b

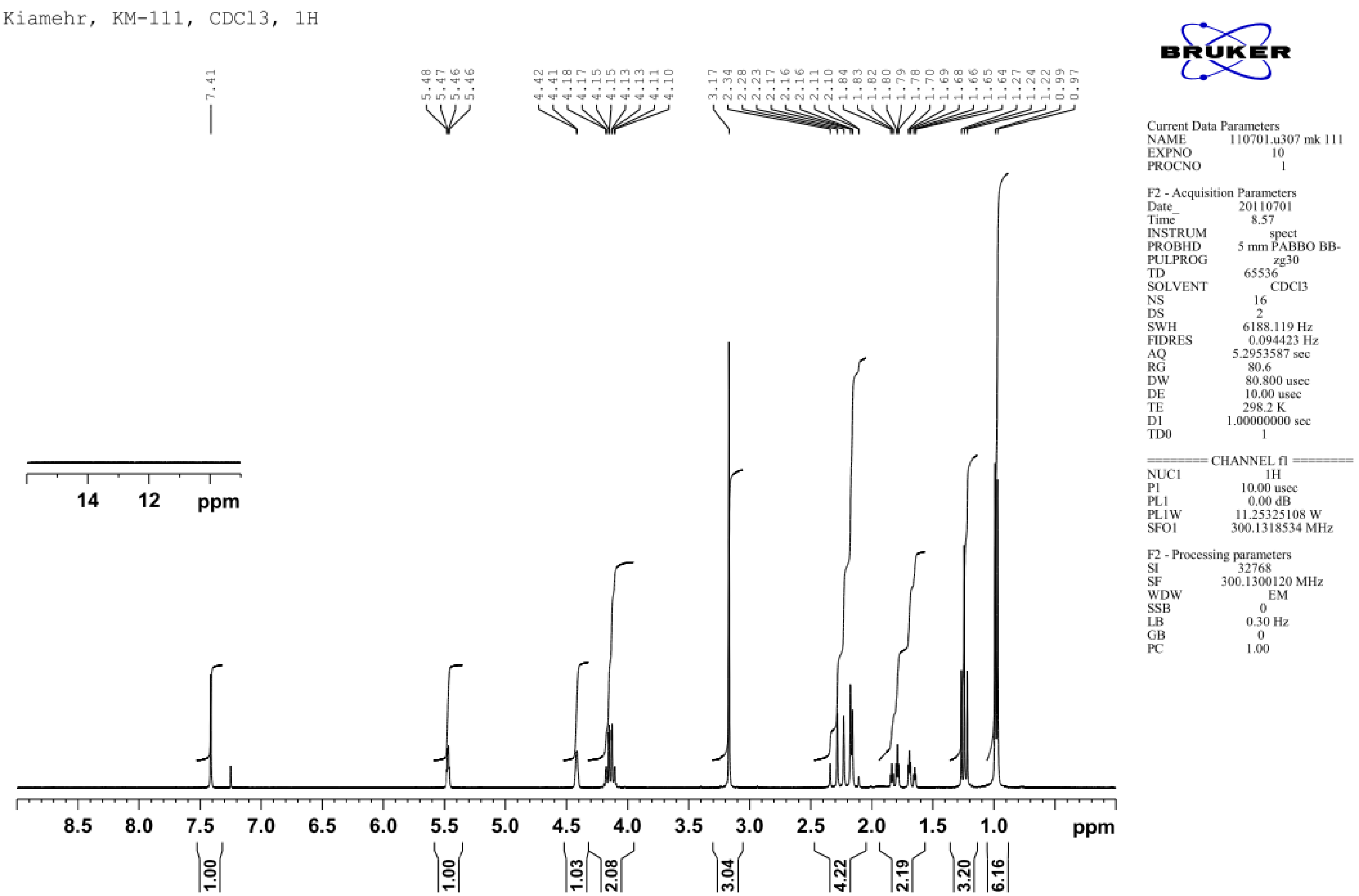

^{1}H NMR spectra for compound 3c

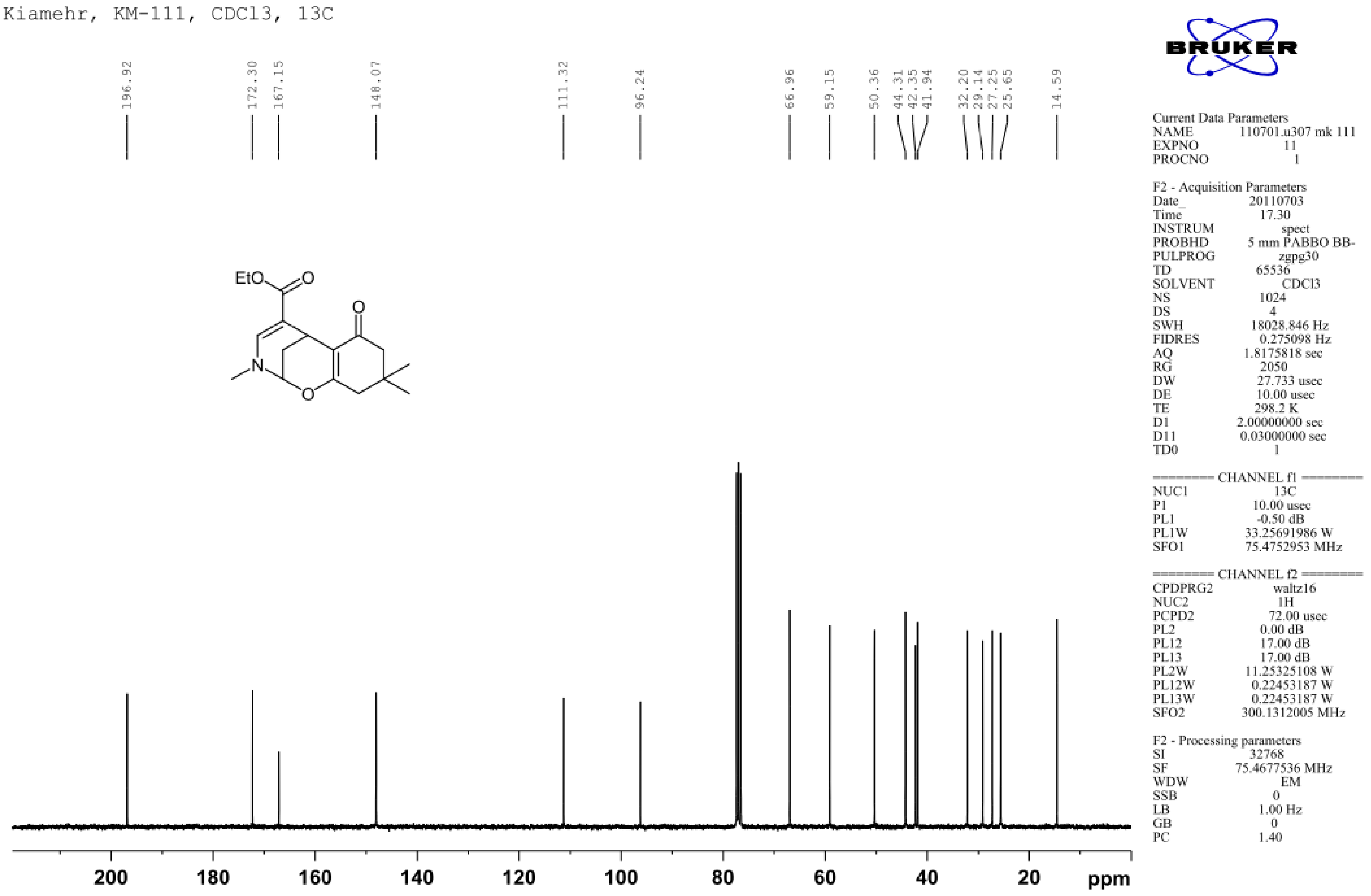

^{13}C NMR spectra for compound 3c

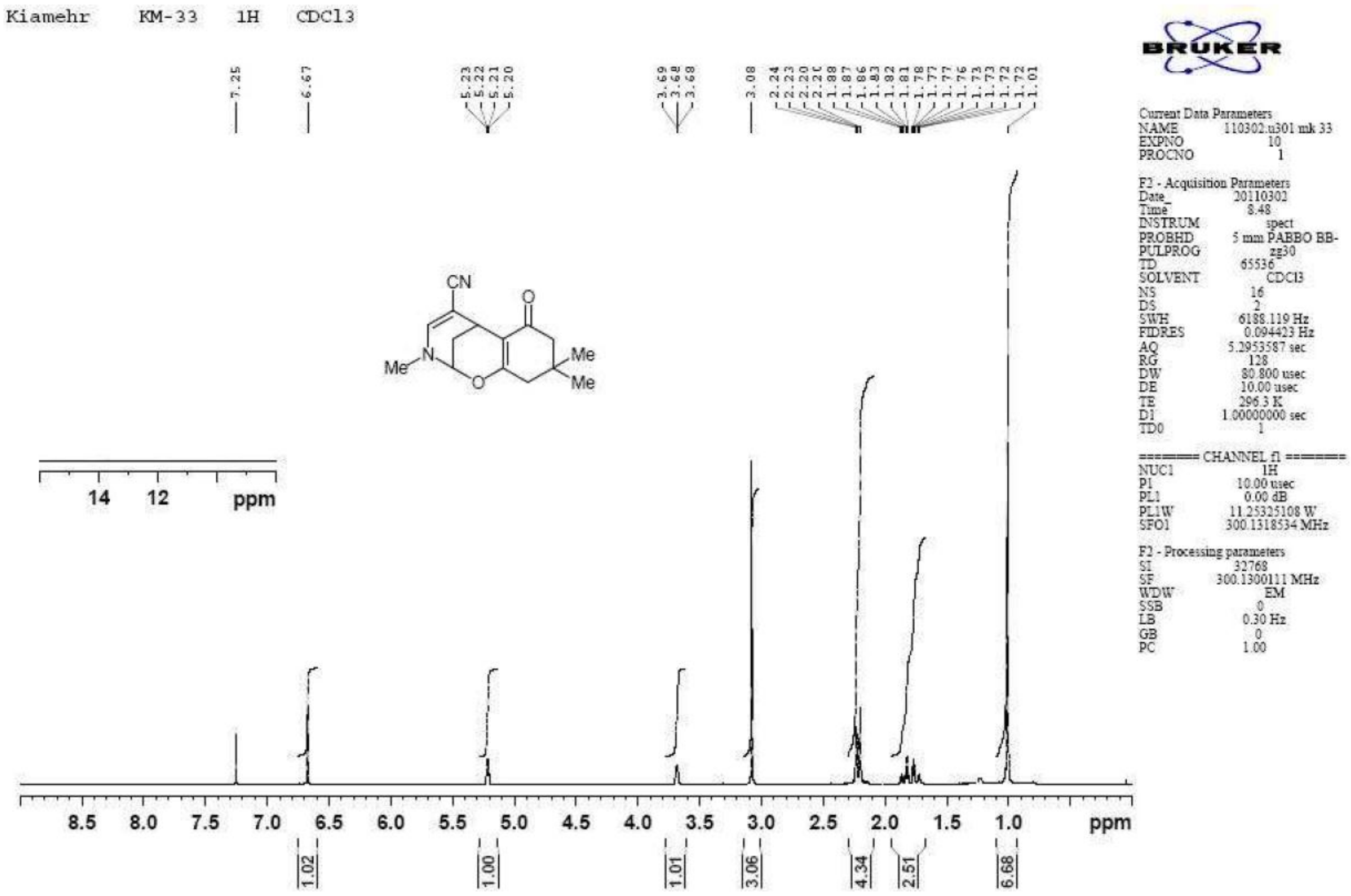

^{1}H NMR spectra for compound 3d

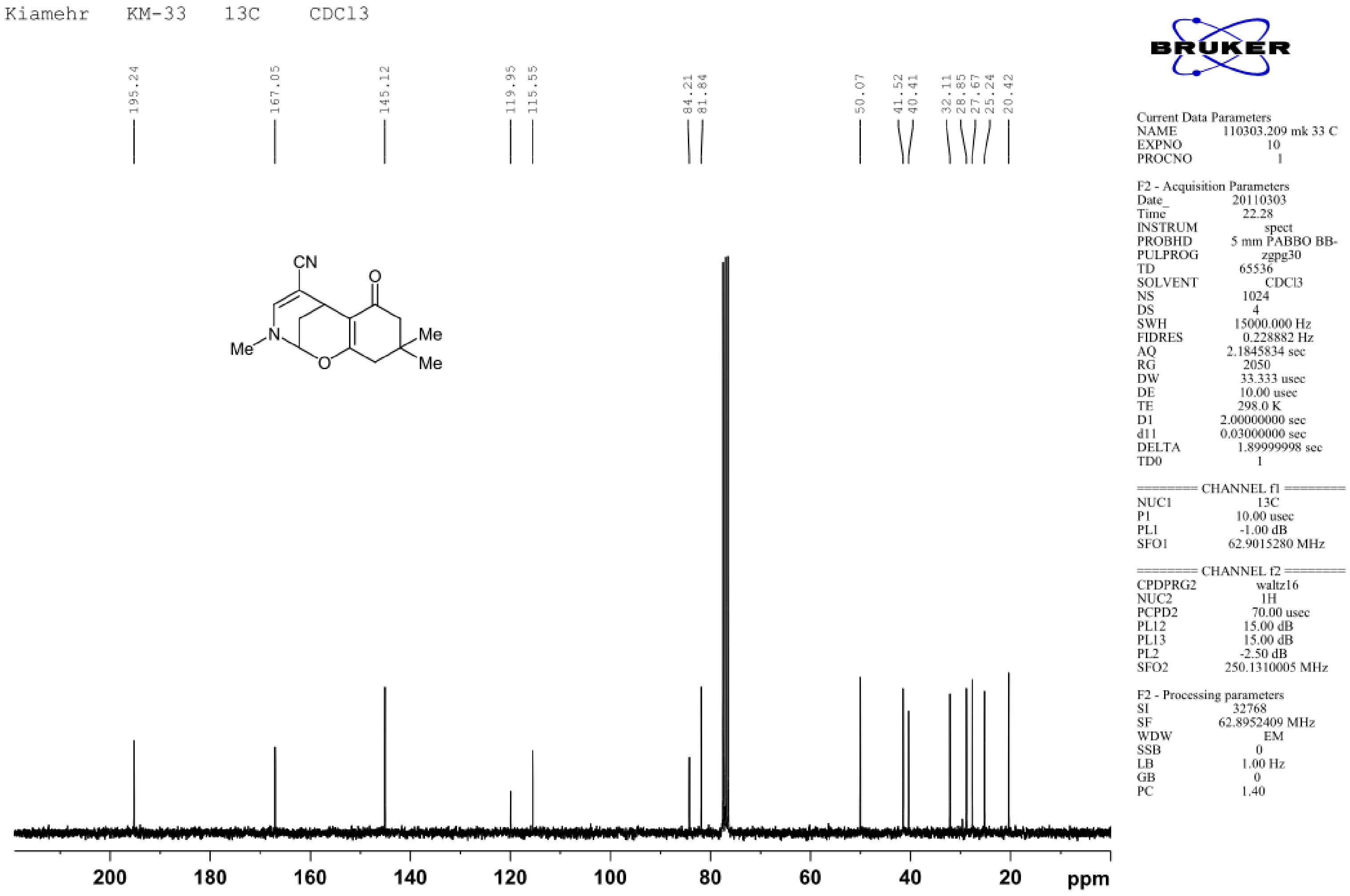

^{13}C NMR spectra for compound 3d

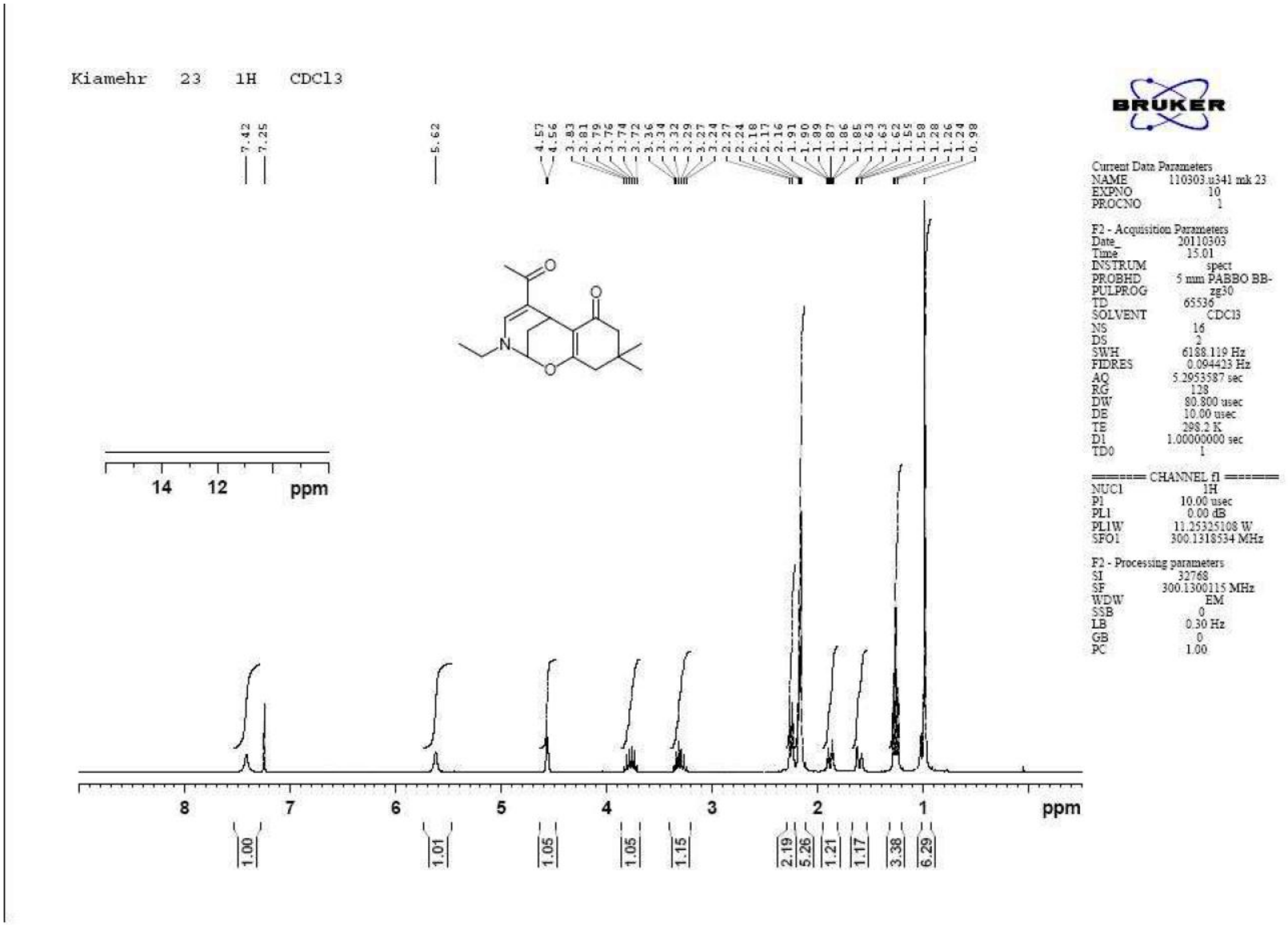

^{1}H NMR spectra for compound 3e

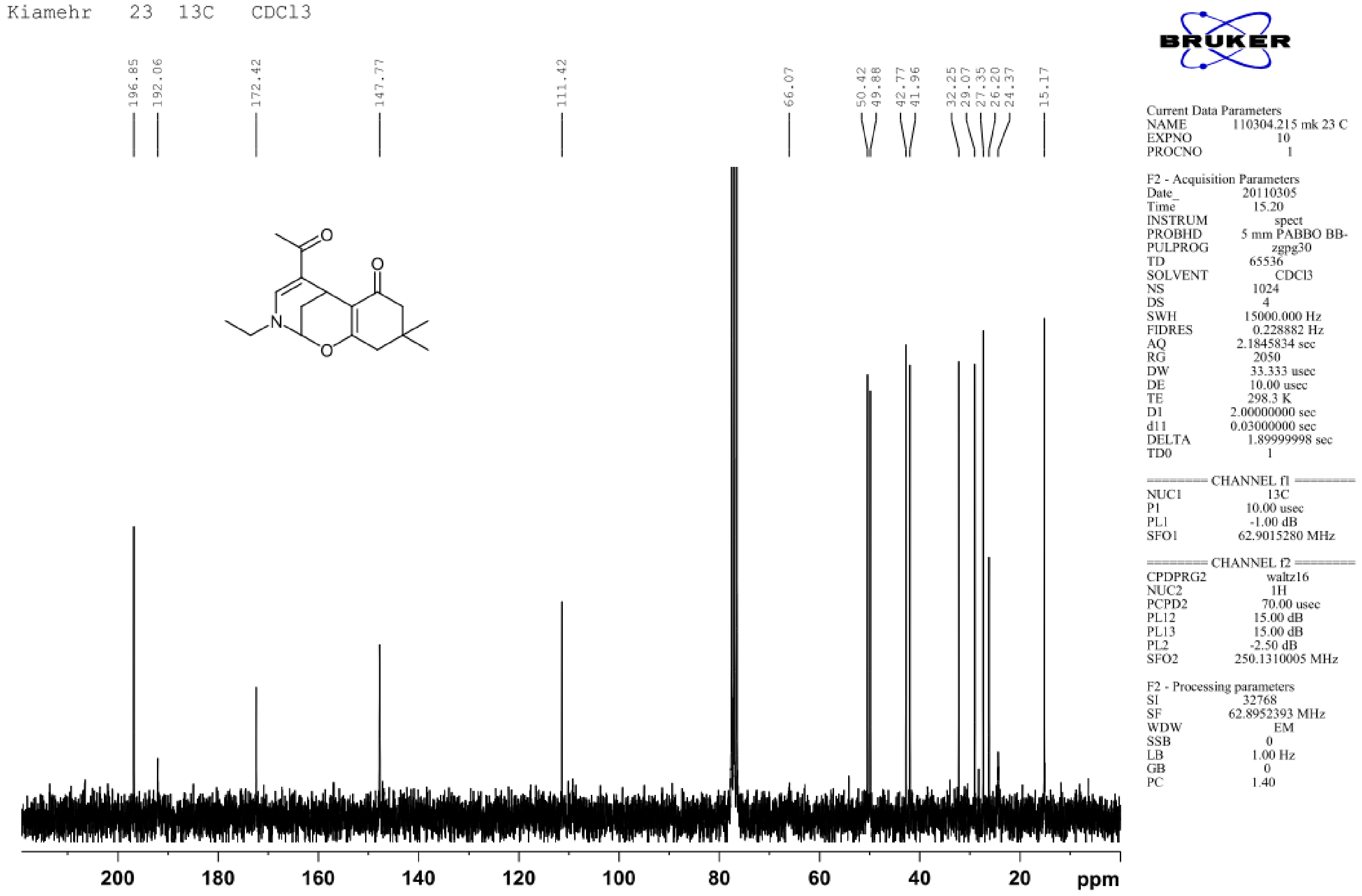

^{13}C NMR spectra for compound 3e

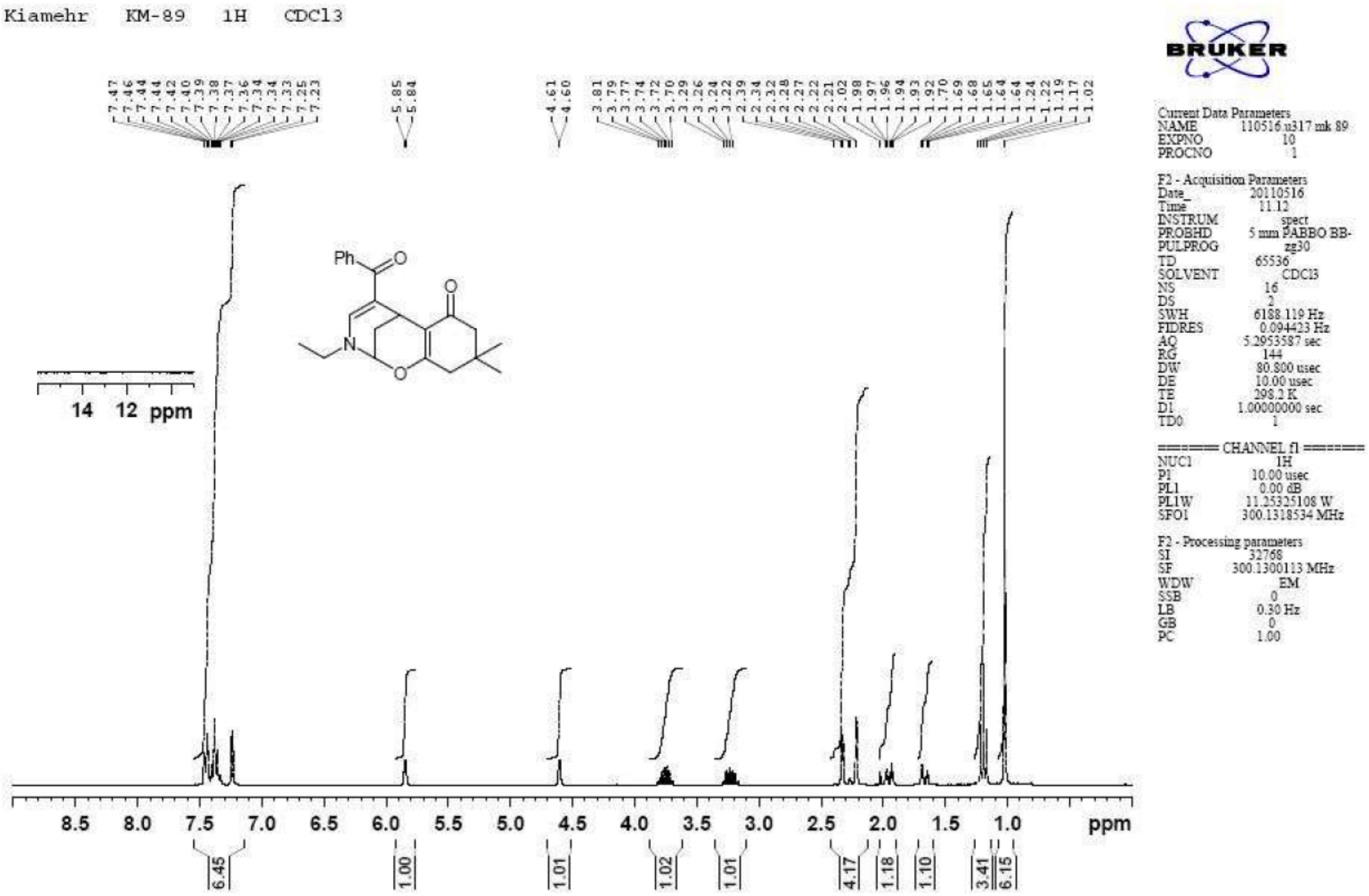

^{1}H NMR spectra for compound 3f

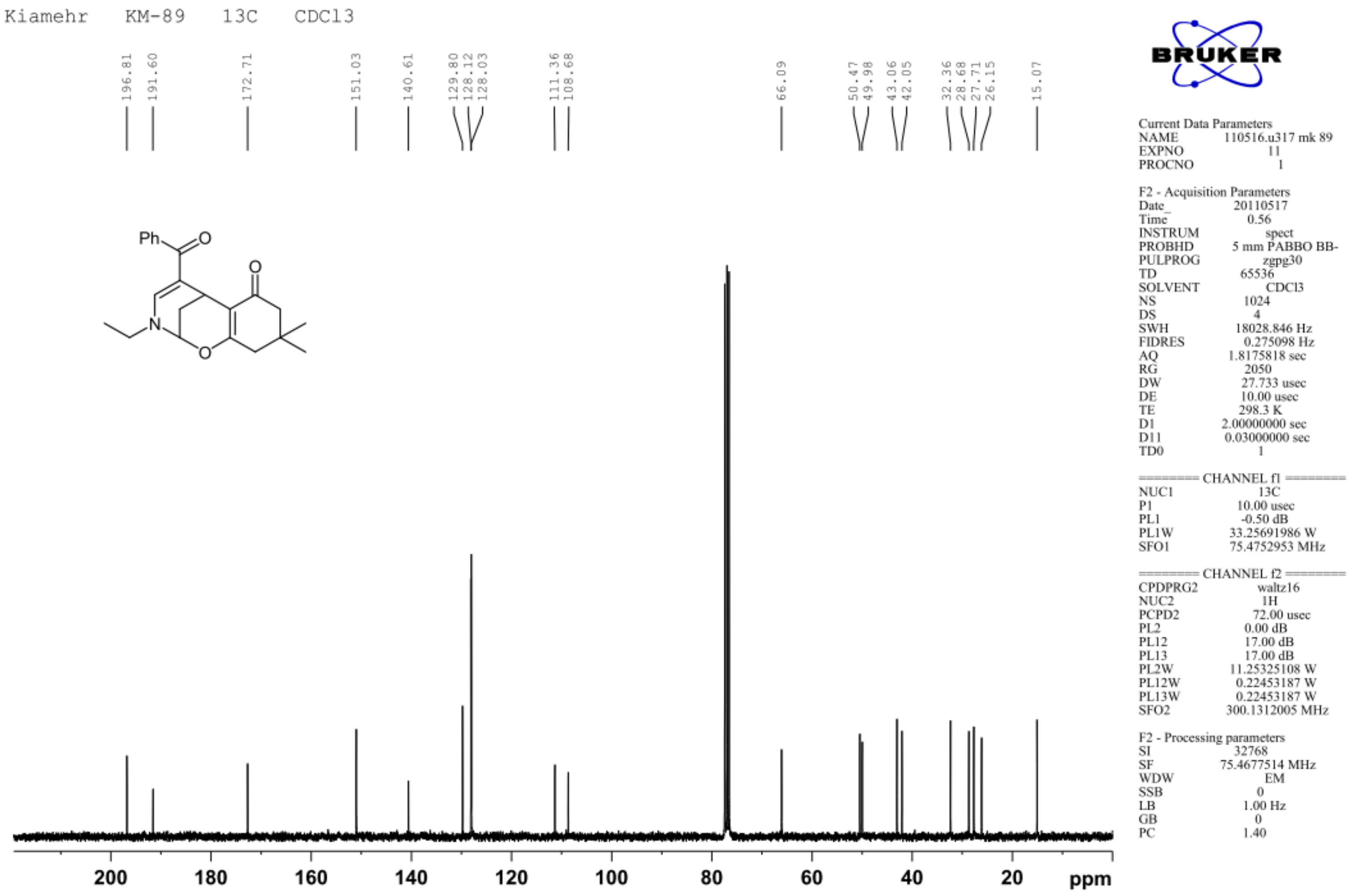

^{13}C NMR spectra for compound 3f

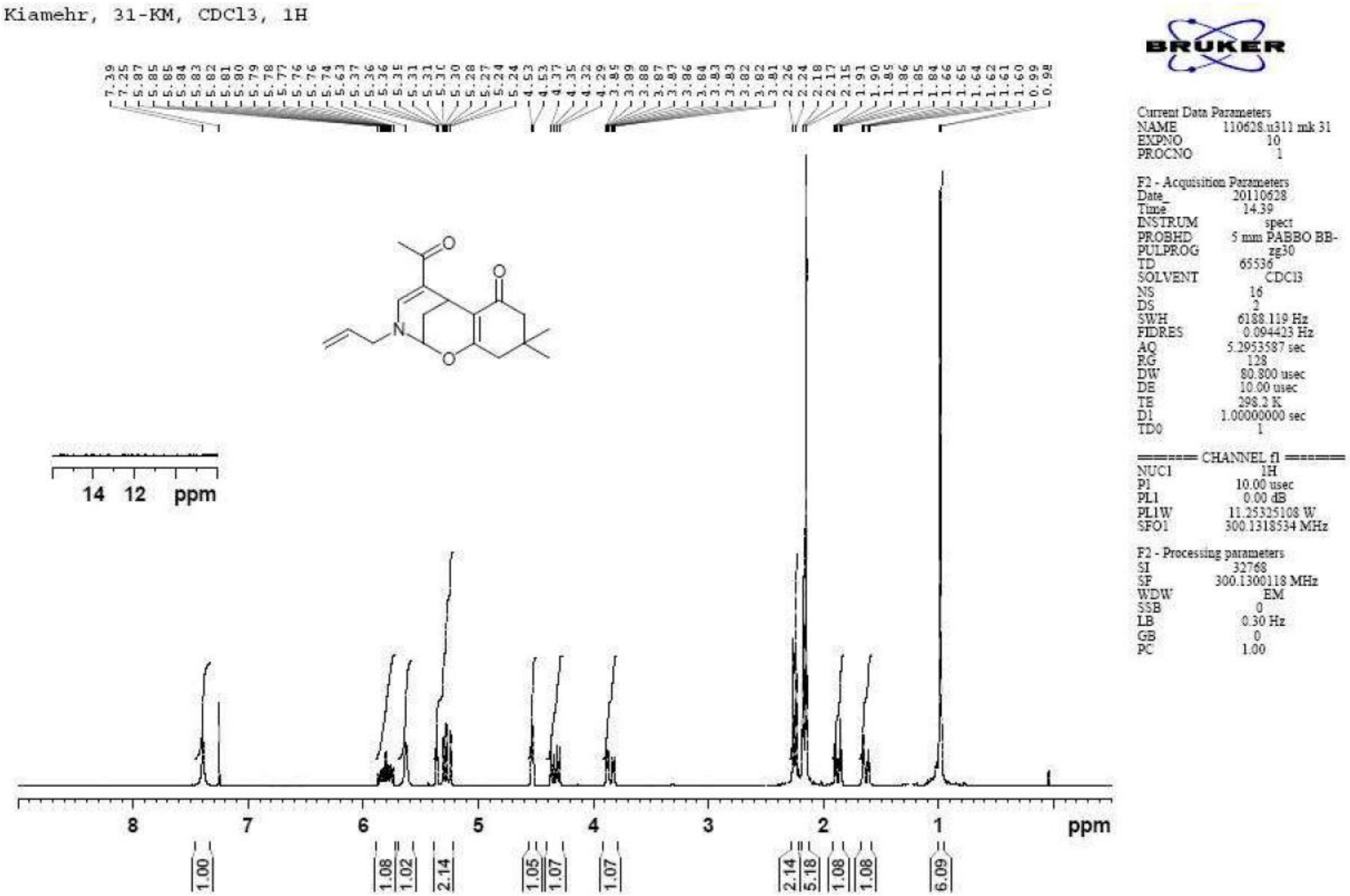

^{1}H NMR spectra for compound 3g

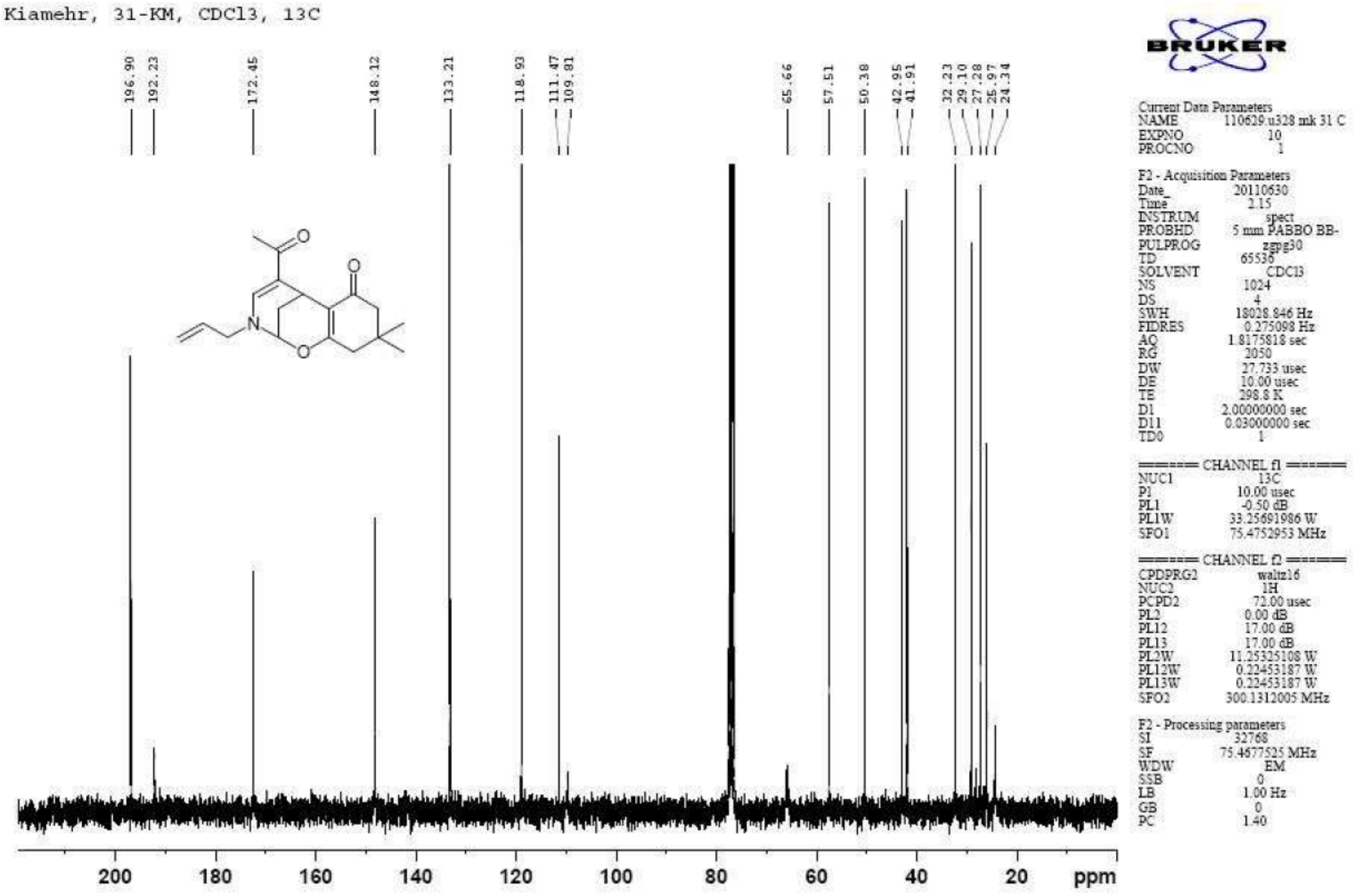

^{13}C NMR spectra for compound 3g

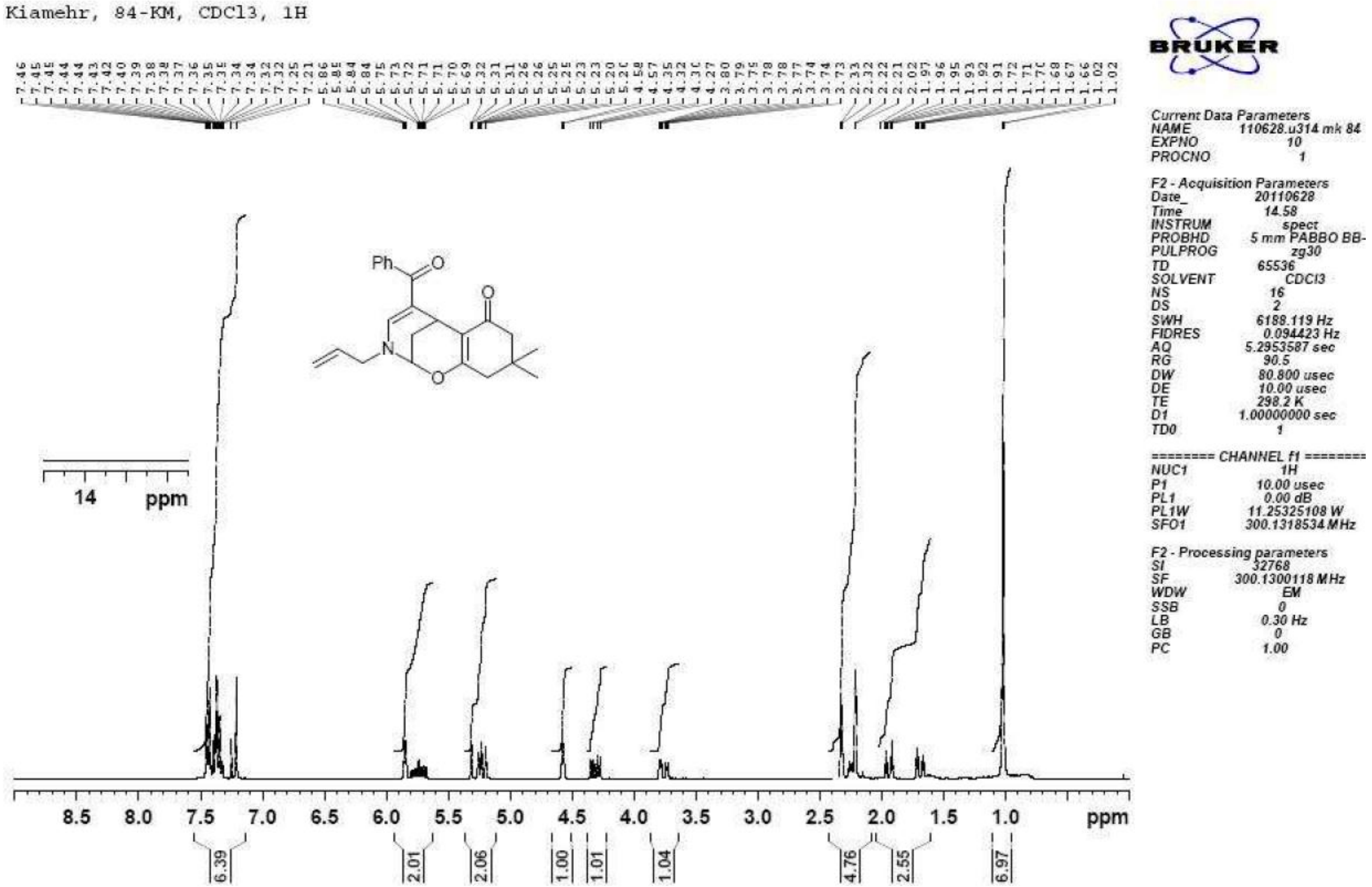

^{1}H NMR spectra for compound 3h

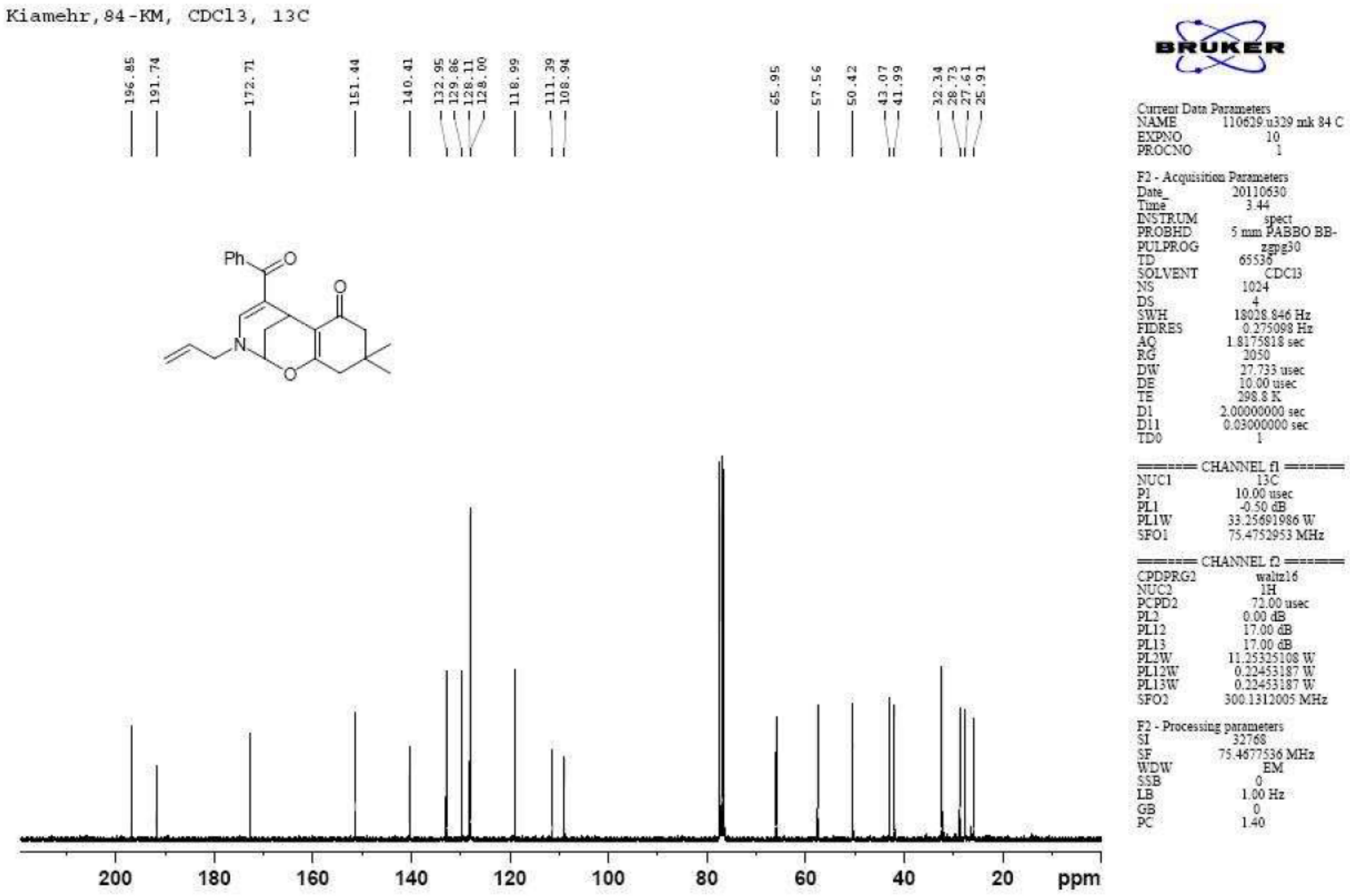

^{13}C NMR spectra for compound 3h

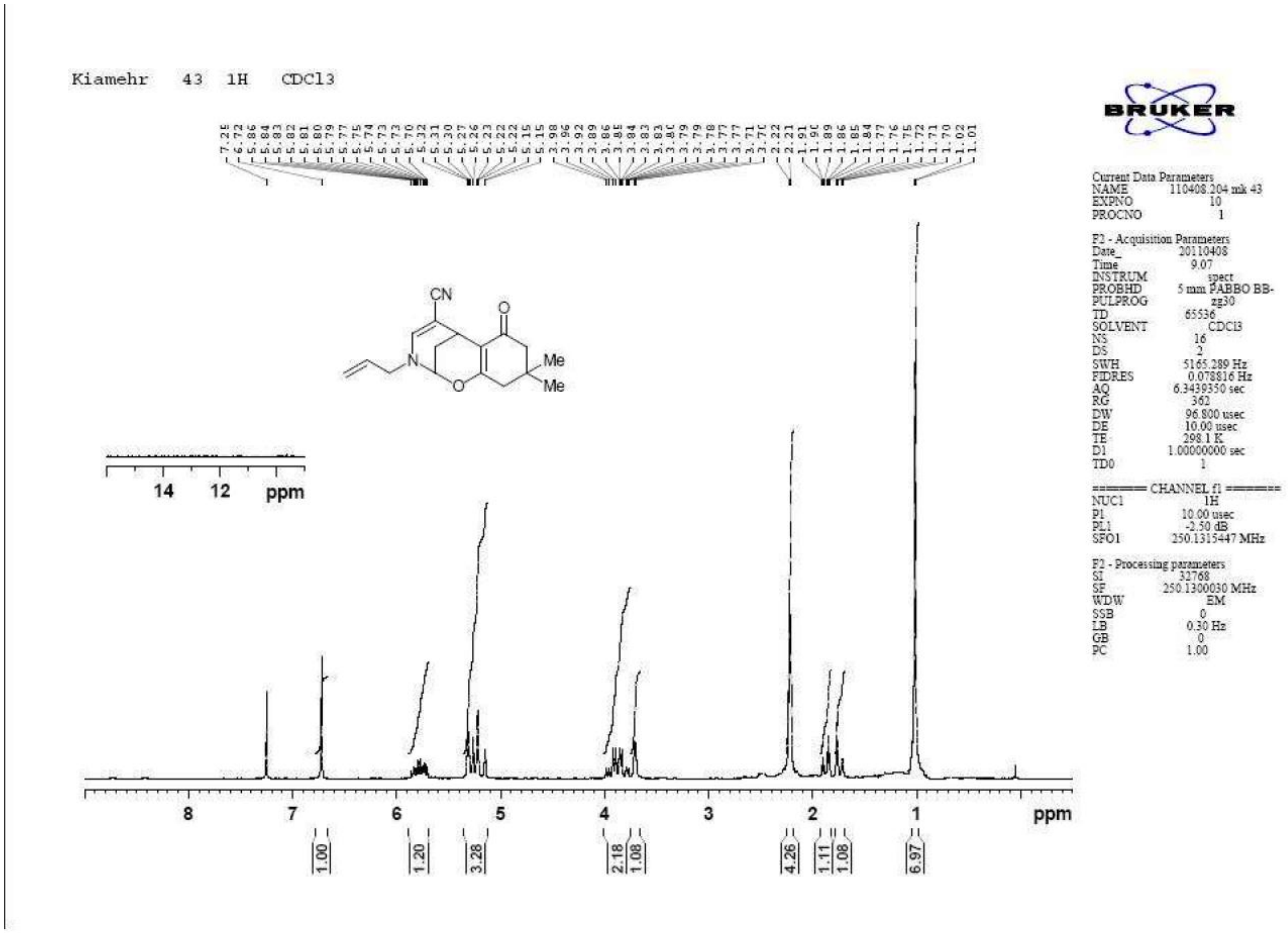

^{1}H NMR spectra for compound 3i

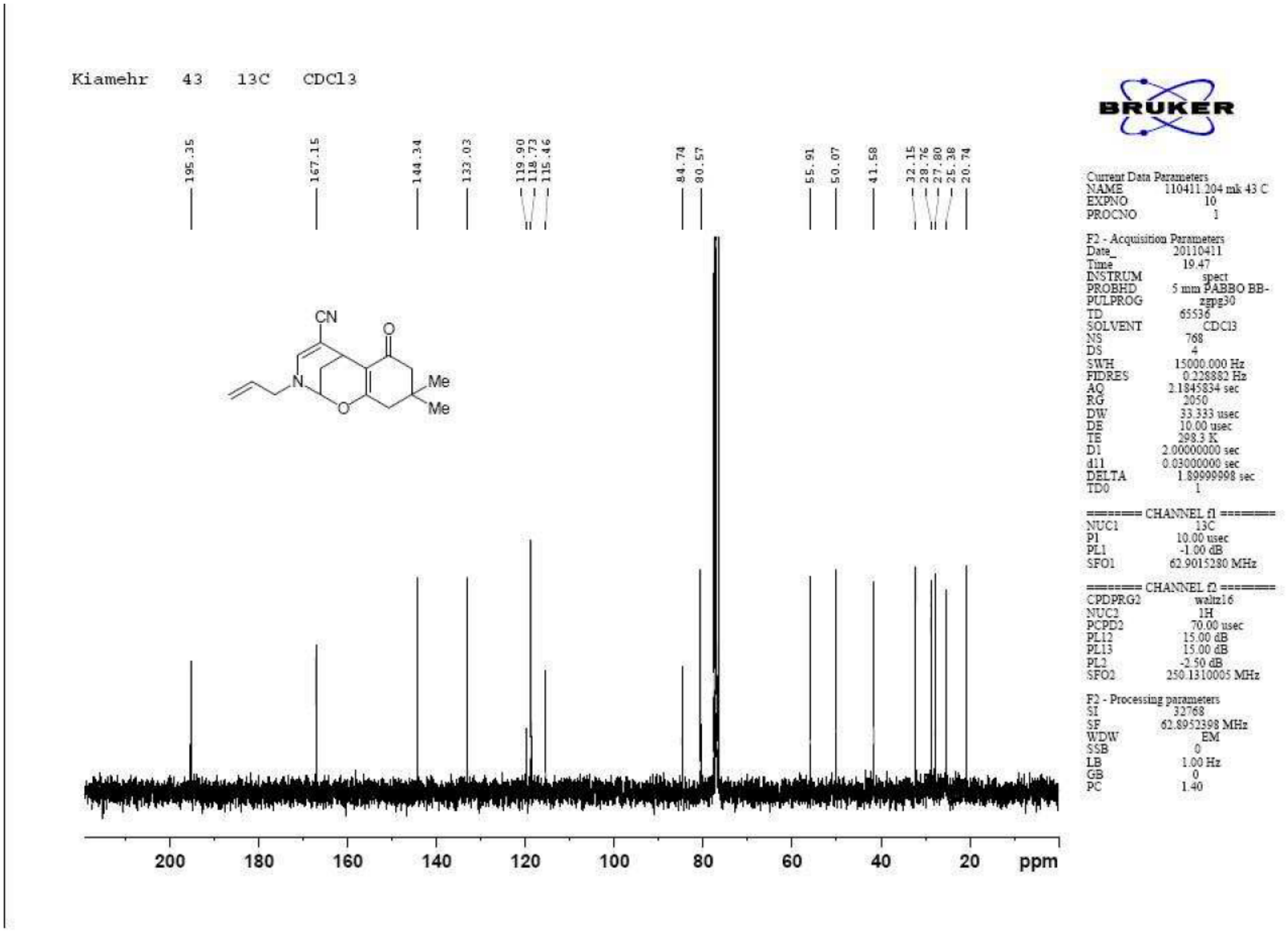

^{13}C NMR spectra for compound 3i

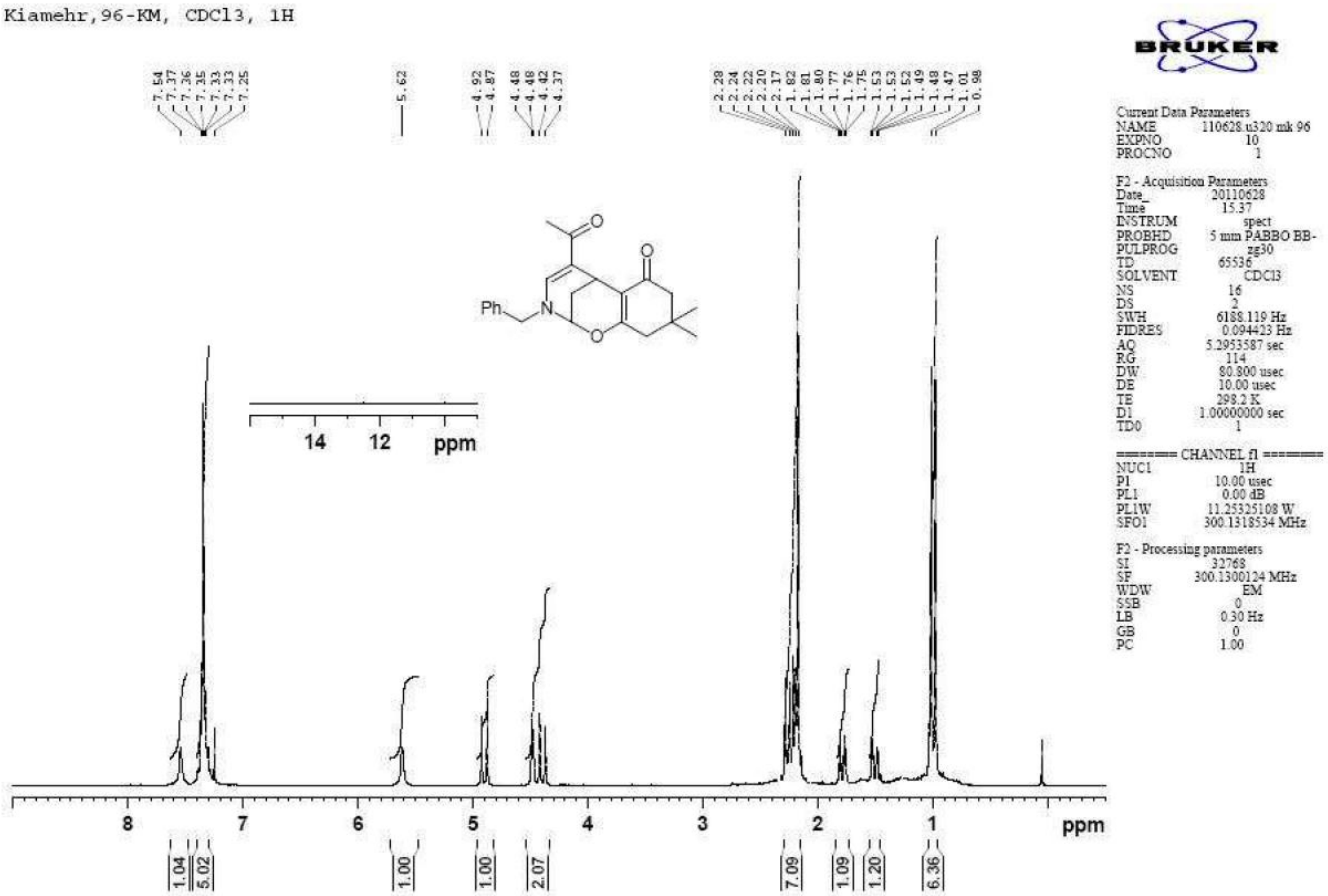

^{1}H NMR spectra for compound 3j

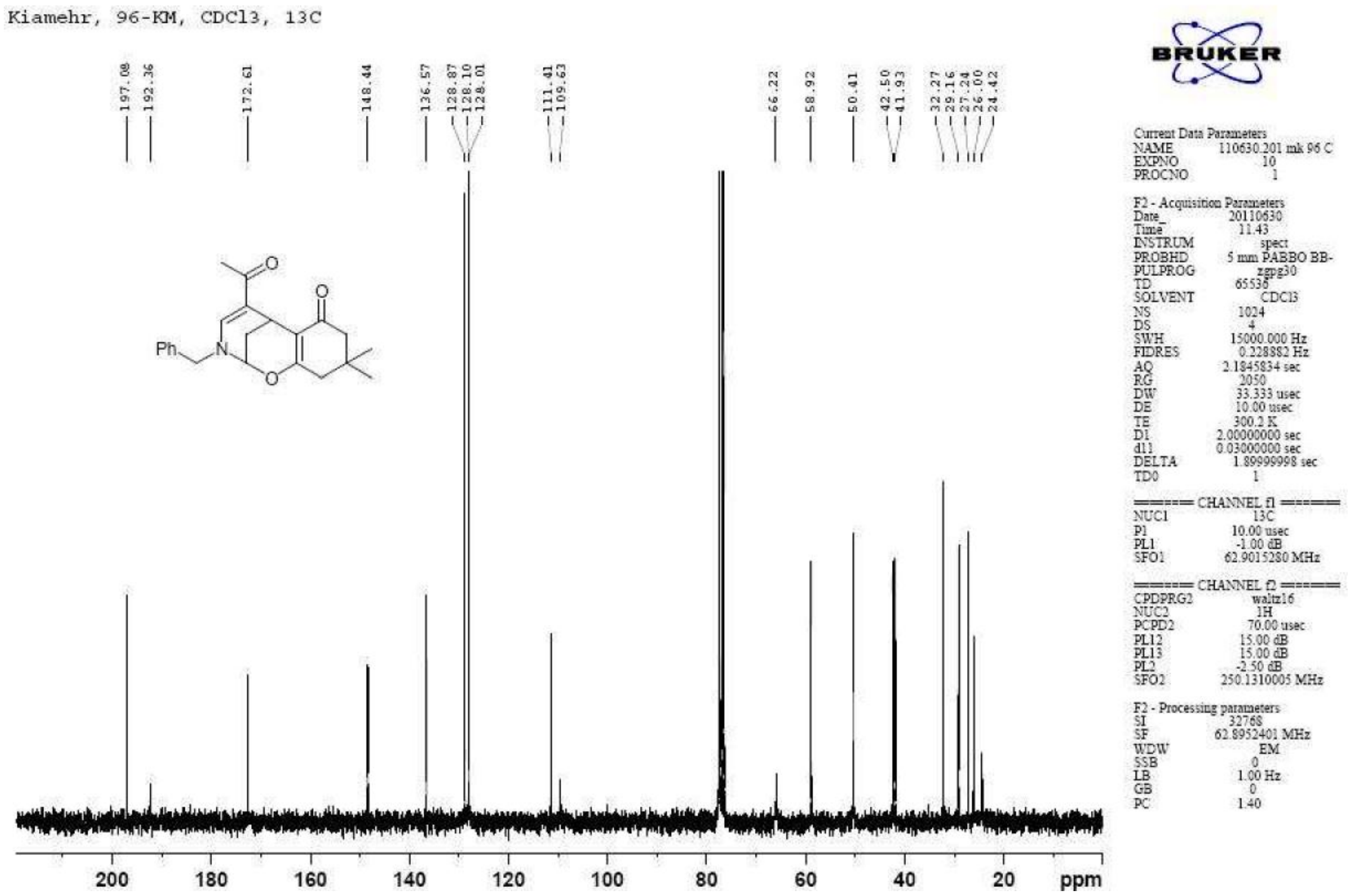

^{13}C NMR spectra for compound 3j

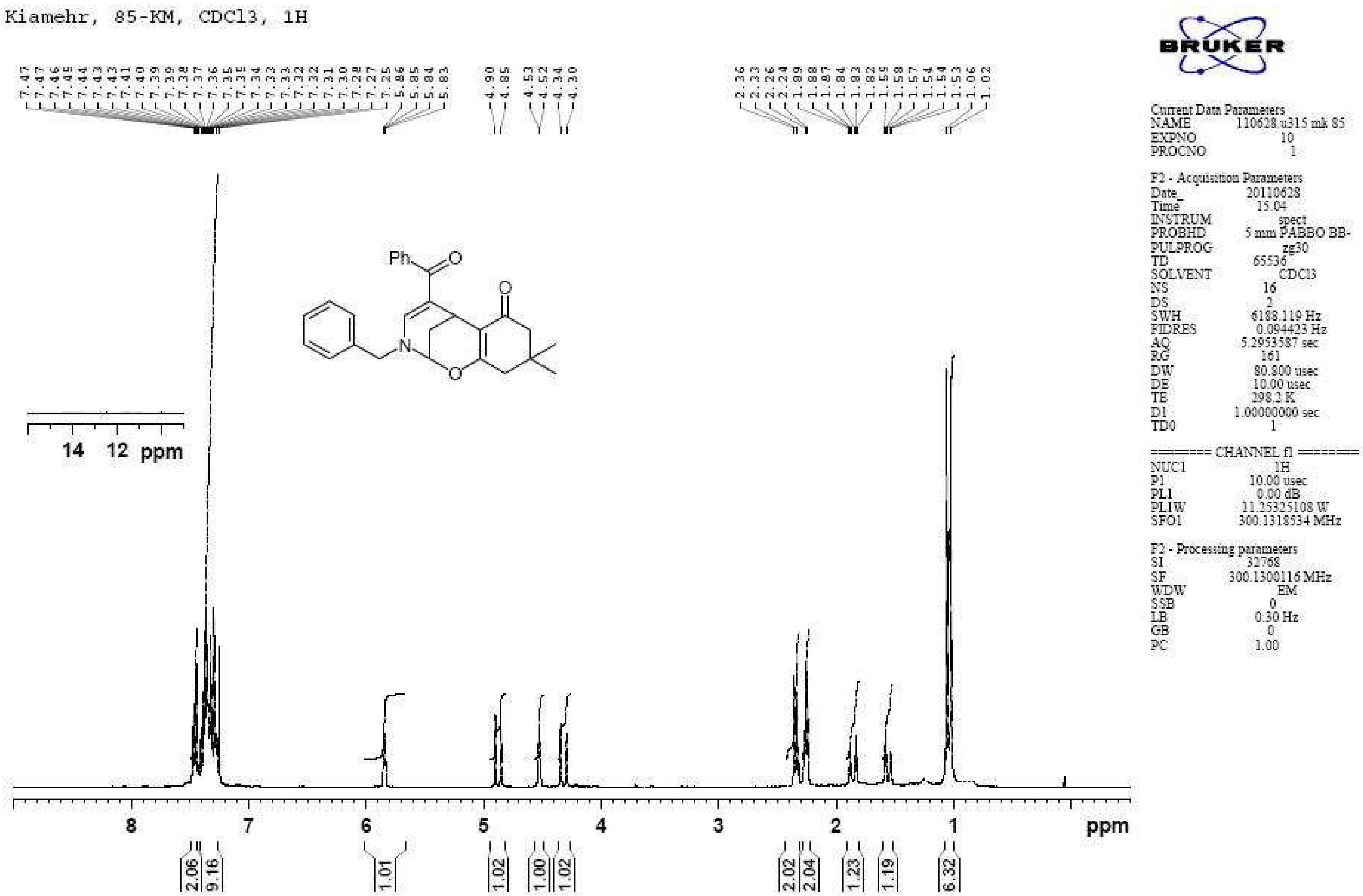

^{1}H NMR spectra for compound 3k

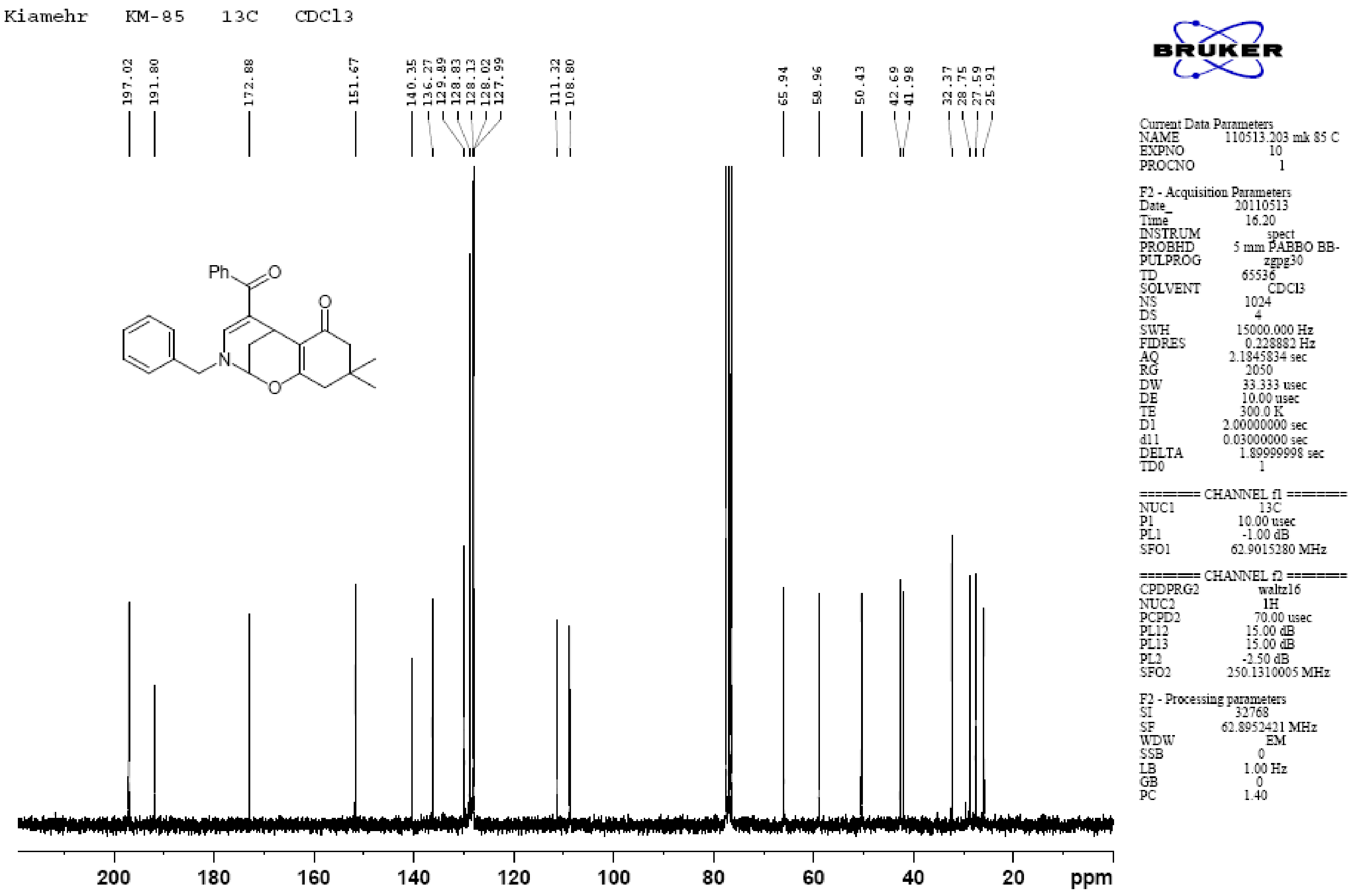

^{13}C NMR spectra for compound 3k

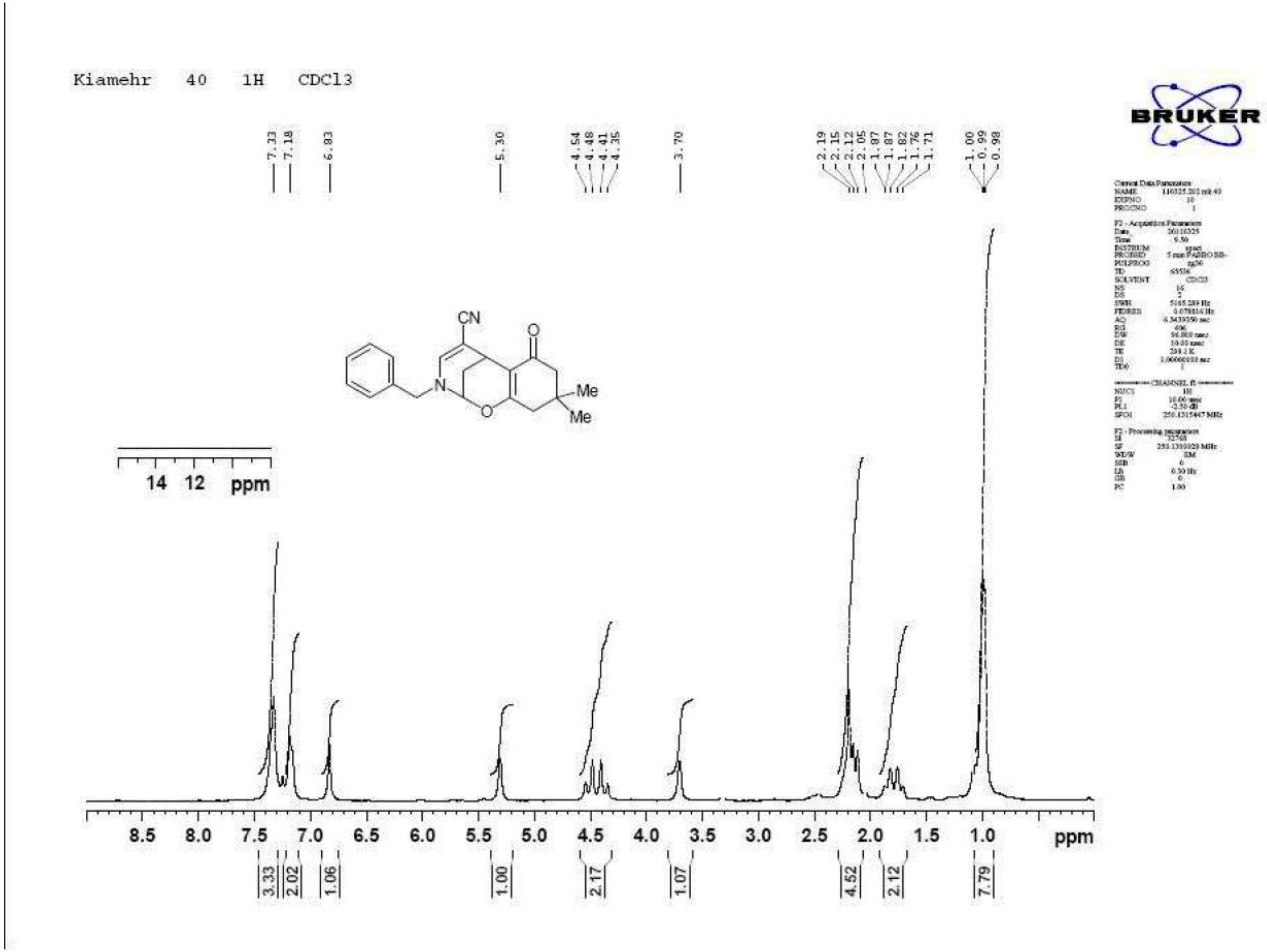

^{1}H NMR spectra for compound 3l

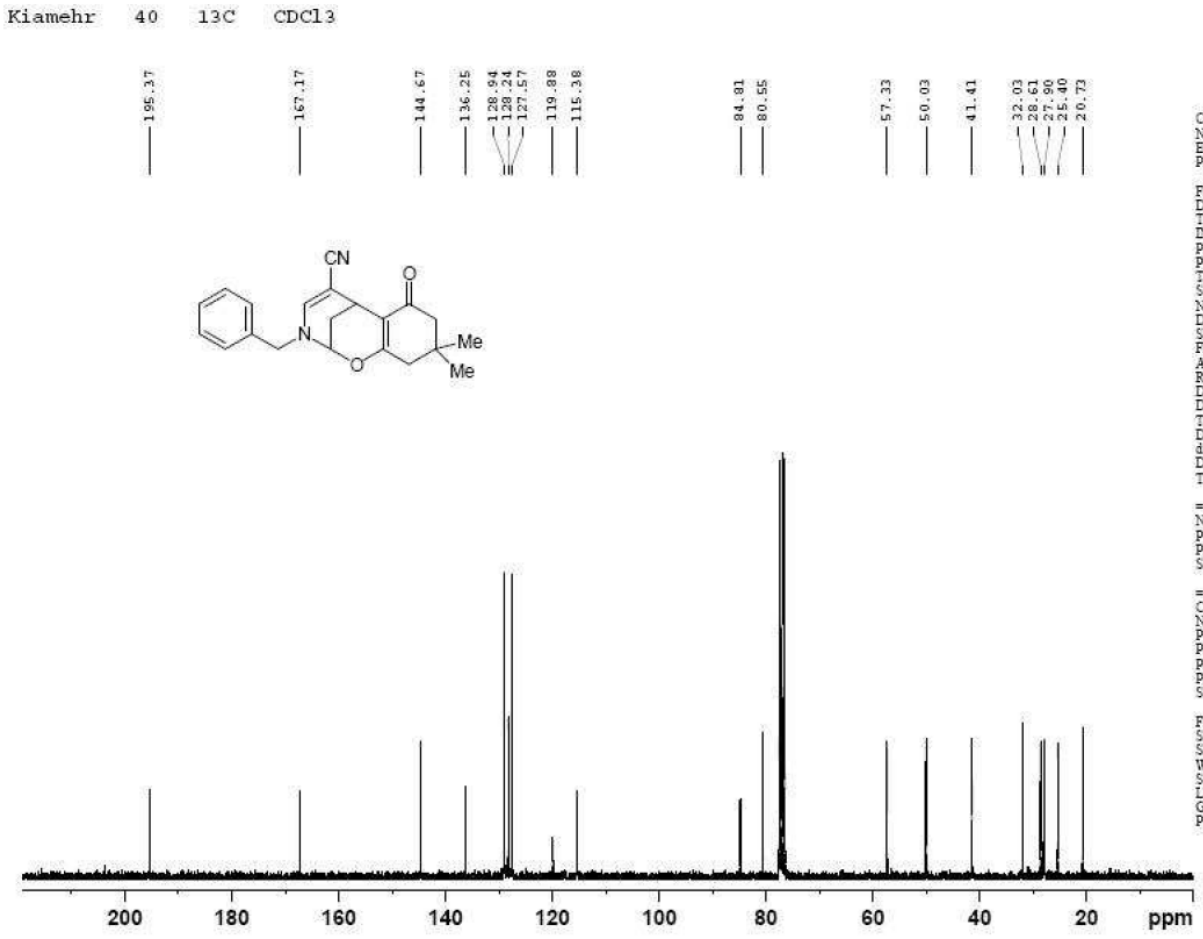

Current Data Parameters
NAME 110328.206 mk 40 C
EXPNO 10
PROCNO 1

F2 - Acquisition Parameters
Date_ 20110328
Time 15.06
INSTRUM spect
PROBHD 5 mm PABBO BB-
PULPROG zgpg30
TD 65536
SOLVENT CDCl3
NS 1024
DS 4
SWH 15000.000 Hz
FIDRES 0.228882 Hz
AQ 2.1845834 sec
RG 2050
DW 33.333 usec
DE 10.00 usec
TE 298.2 K
D1 2.00000000 sec
d11 0.03000000 sec
DELTA 1.89999998 sec
TD0 1

======= CHANNEL f1 =======
NUC1 13C
P1 10.00 usec
PL1 -1.00 dB
SFO1 62.9015280 MHz

======= CHANNEL f2 =======
CPDPRG2 waltz16
NUC2 1H
PCPD2 70.00 usec
PL12 15.00 dB
PL13 15.00 dB
PL2 -2.50 dB
SFO2 250.1310005 MHz

F2 - Processing parameters
SI 32768
SF 62.8952416 MHz
WDW EM
SSB 0
LB 1.00 Hz
GB 0
PC 1.40

^{13}C NMR spectra for compound 3l

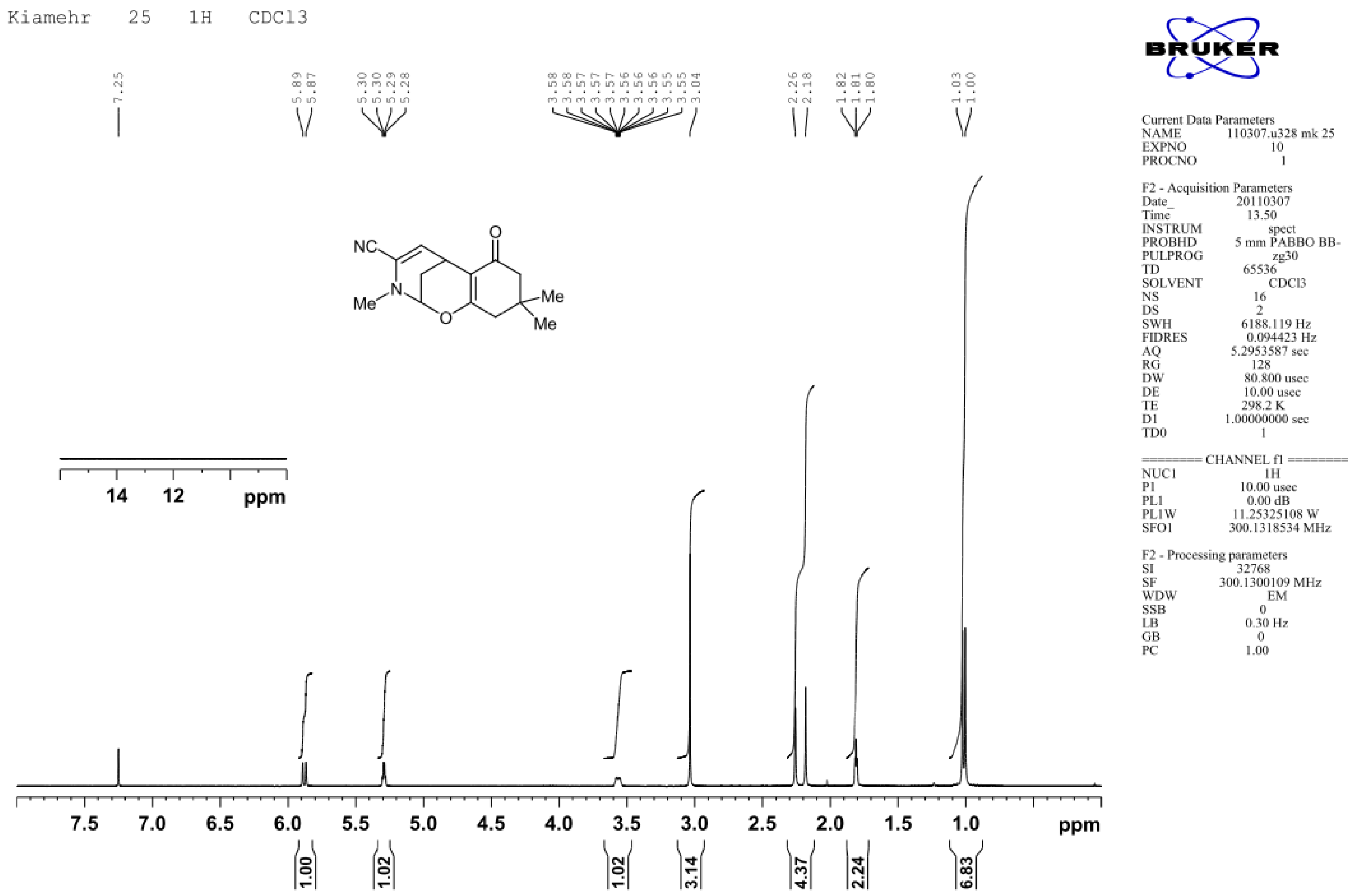

^{1}H NMR spectra for compound 4a

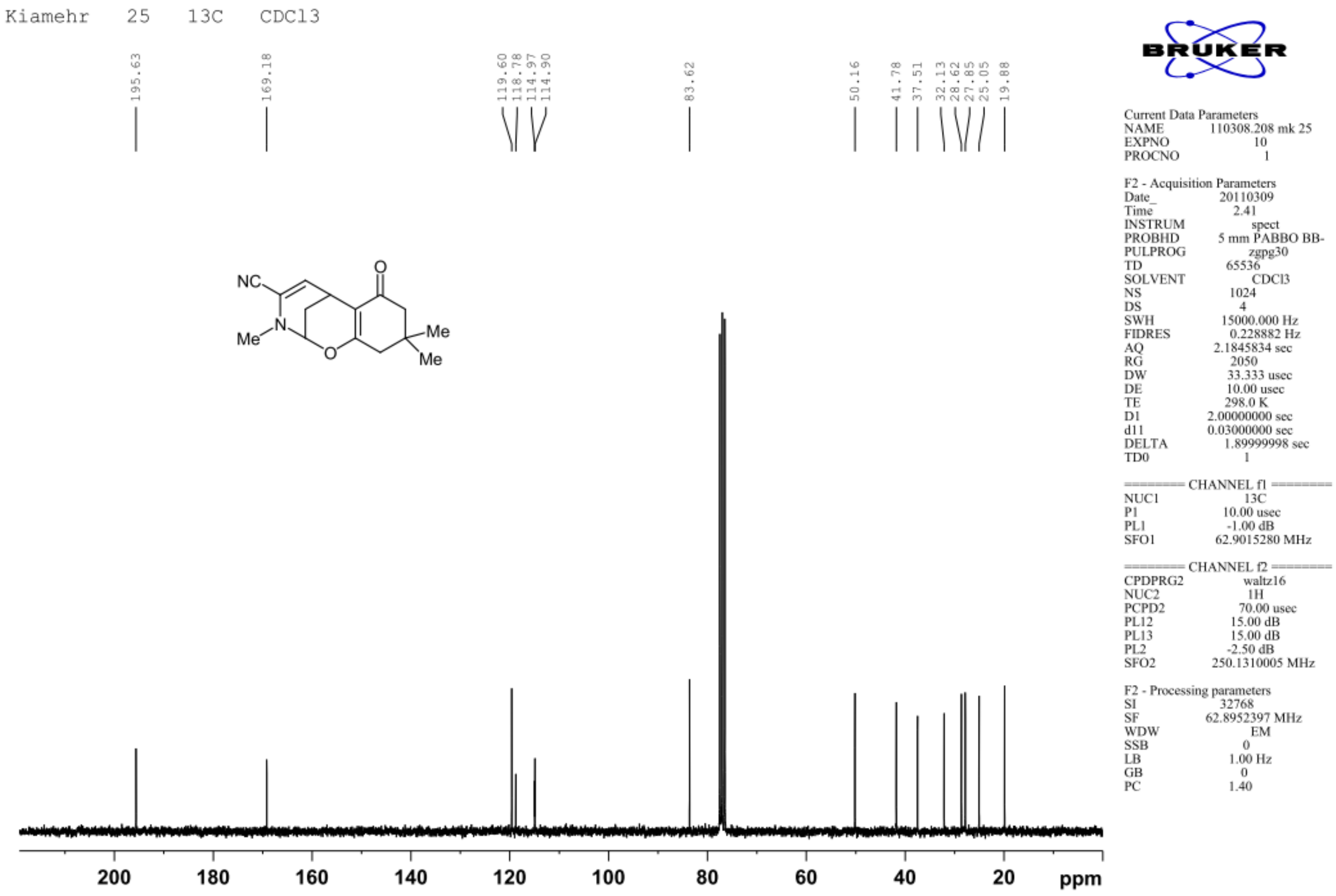

^{13}C NMR spectra for compound 4a

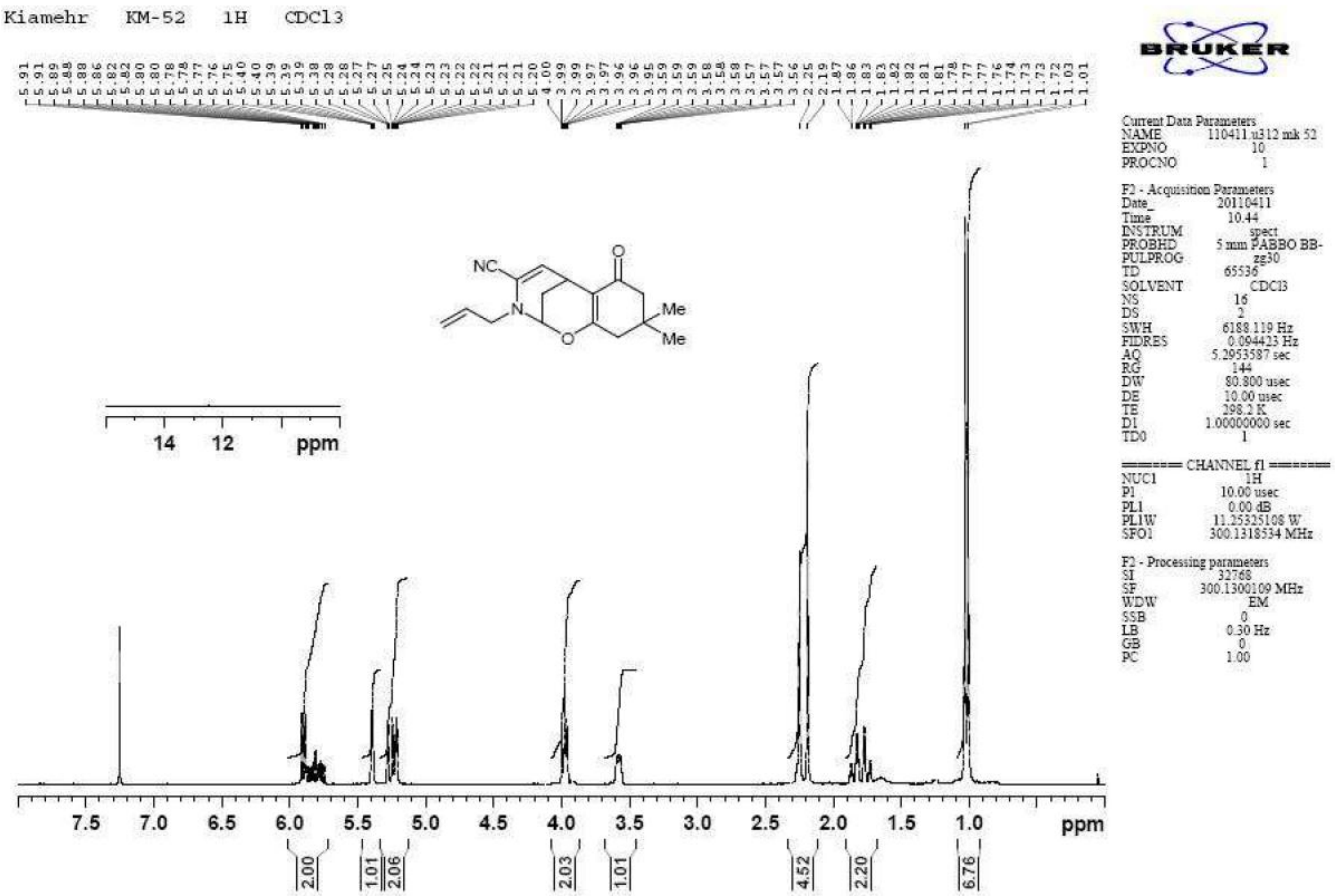

^{1}H NMR spectra for compound 4b

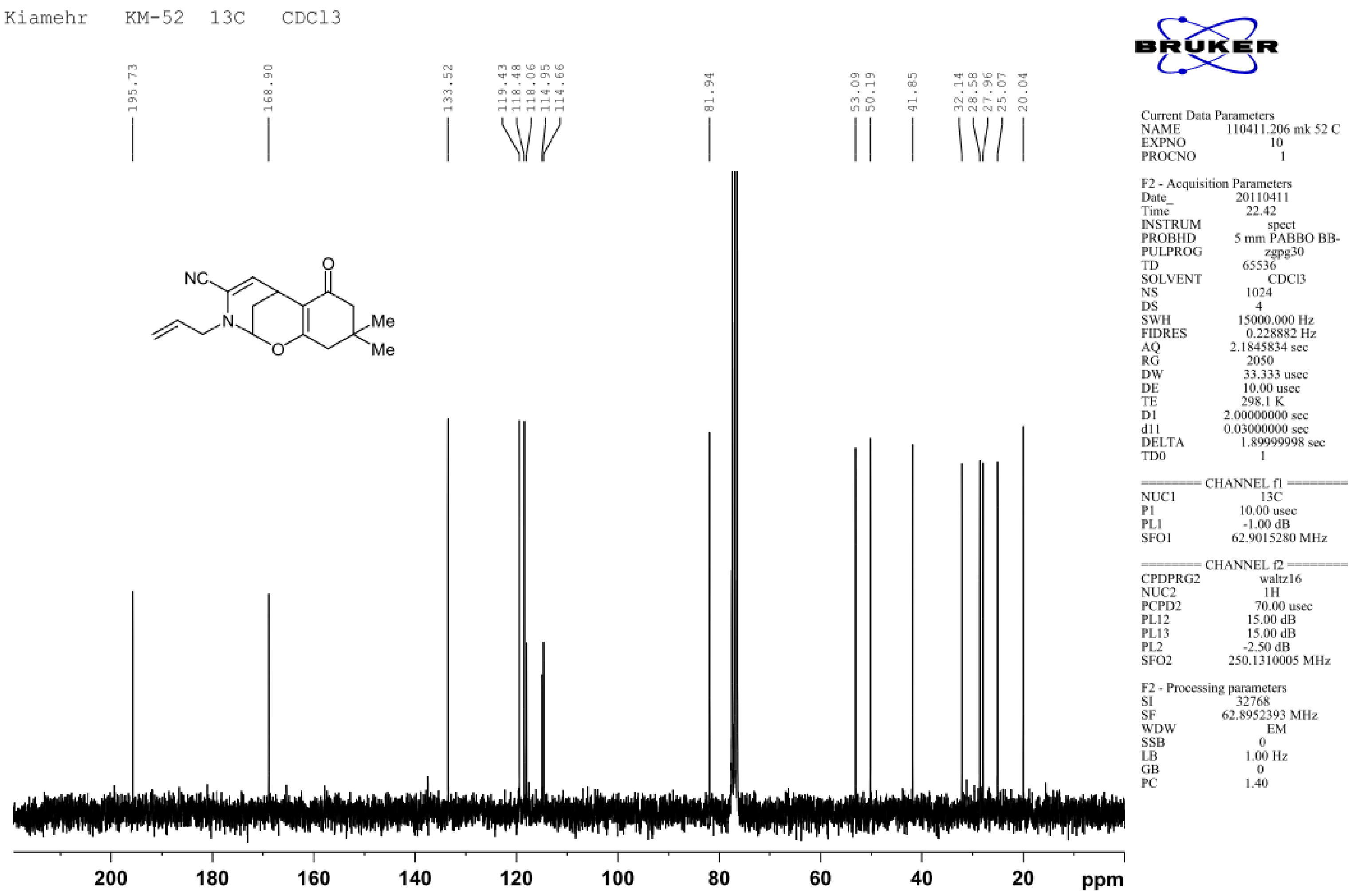

^{13}C NMR spectra for compound 4b

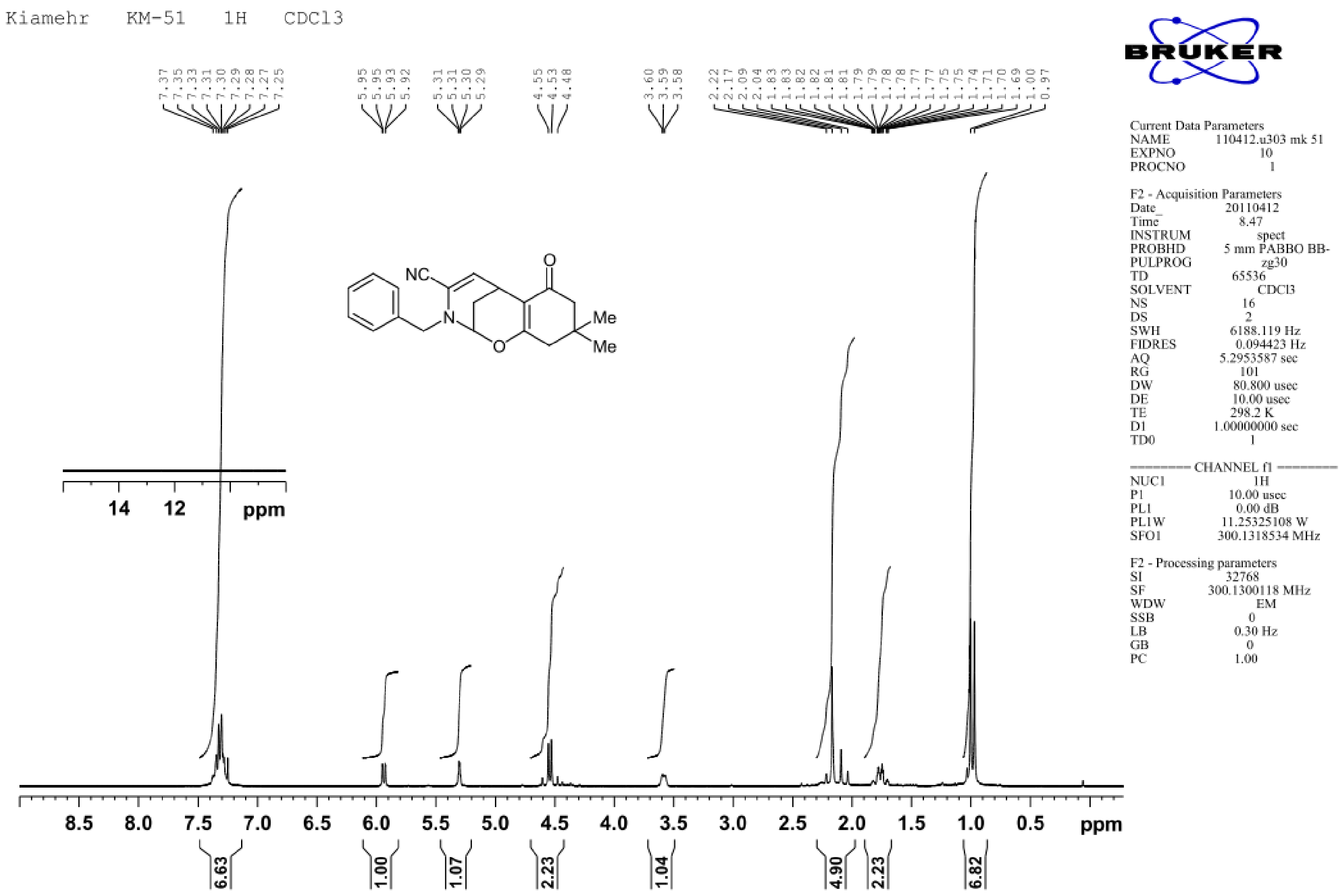

^{1}H NMR spectra for compound 4c

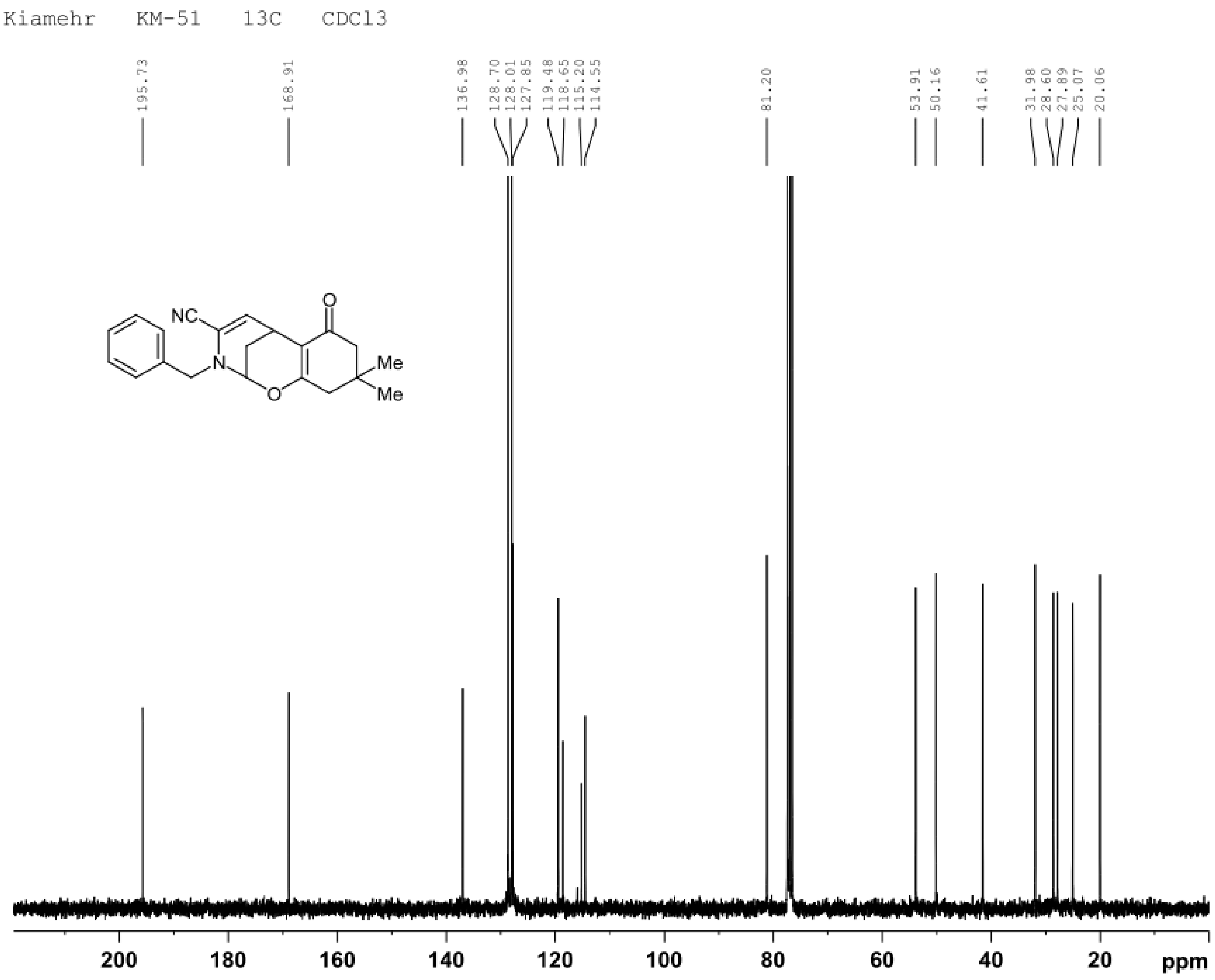

Current Data Parameters
NAME 110412.u303 mk 51
EXPNO 11
PROCNO 1

F2 - Acquisition Parameters
Date_ 20110412
Time 18.08
INSTRUM spect
PROBHD 5 mm PABBO BB-
PULPROG zgpg30
TD 65536
SOLVENT CDCl3
NS 1024
DS 4
SWH 18028.846 Hz
FIDRES 0.275098 Hz
AQ 1.8175818 sec
RG 2050
DW 27.733 usec
DE 10.00 usec
TE 298.9 K
D1 2.00000000 sec
D11 0.03000000 sec
TD0 1

======== CHANNEL f1 ========
NUC1 13C
P1 10.00 usec
PL1 -0.50 dB
PL1W 33.25691986 W
SFO1 75.4752953 MHz

======== CHANNEL f2 ========
CPDPRG2 waltz16
NUC2 1H
PCPD2 72.00 usec
PL2 0.00 dB
PL12 17.00 dB
PL13 17.00 dB
PL2W 11.25325108 W
PL12W 0.22453187 W
PL13W 0.22453187 W
SFO2 300.1312005 MHz

F2 - Processing parameters
SI 32768
SF 75.4677525 MHz
WDW EM
SSB 0
LB 1.00 Hz
GB 0
PC 1.40

^{13}C NMR spectra for compound 4c

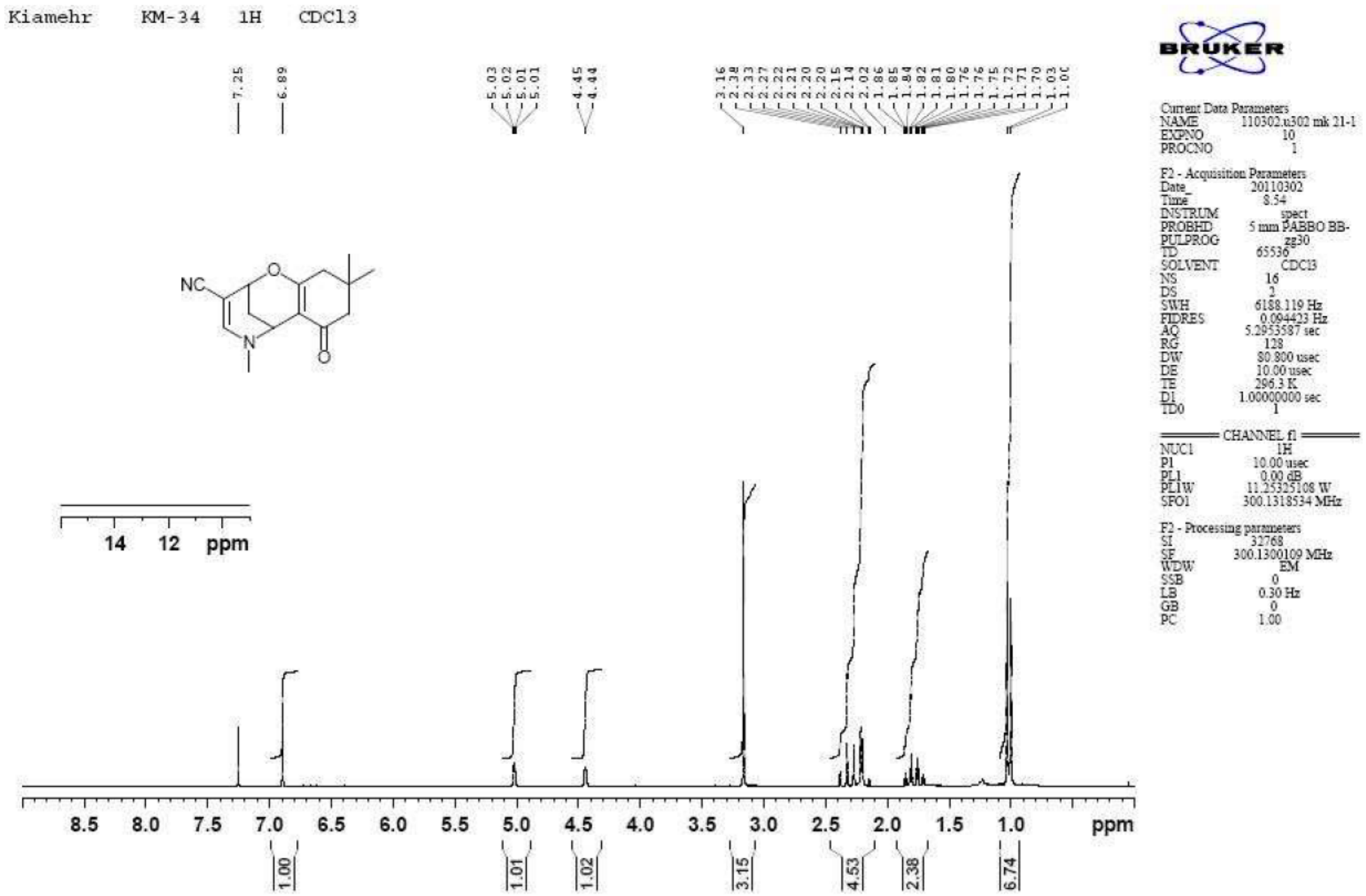

^{1}H NMR spectra for compound 5

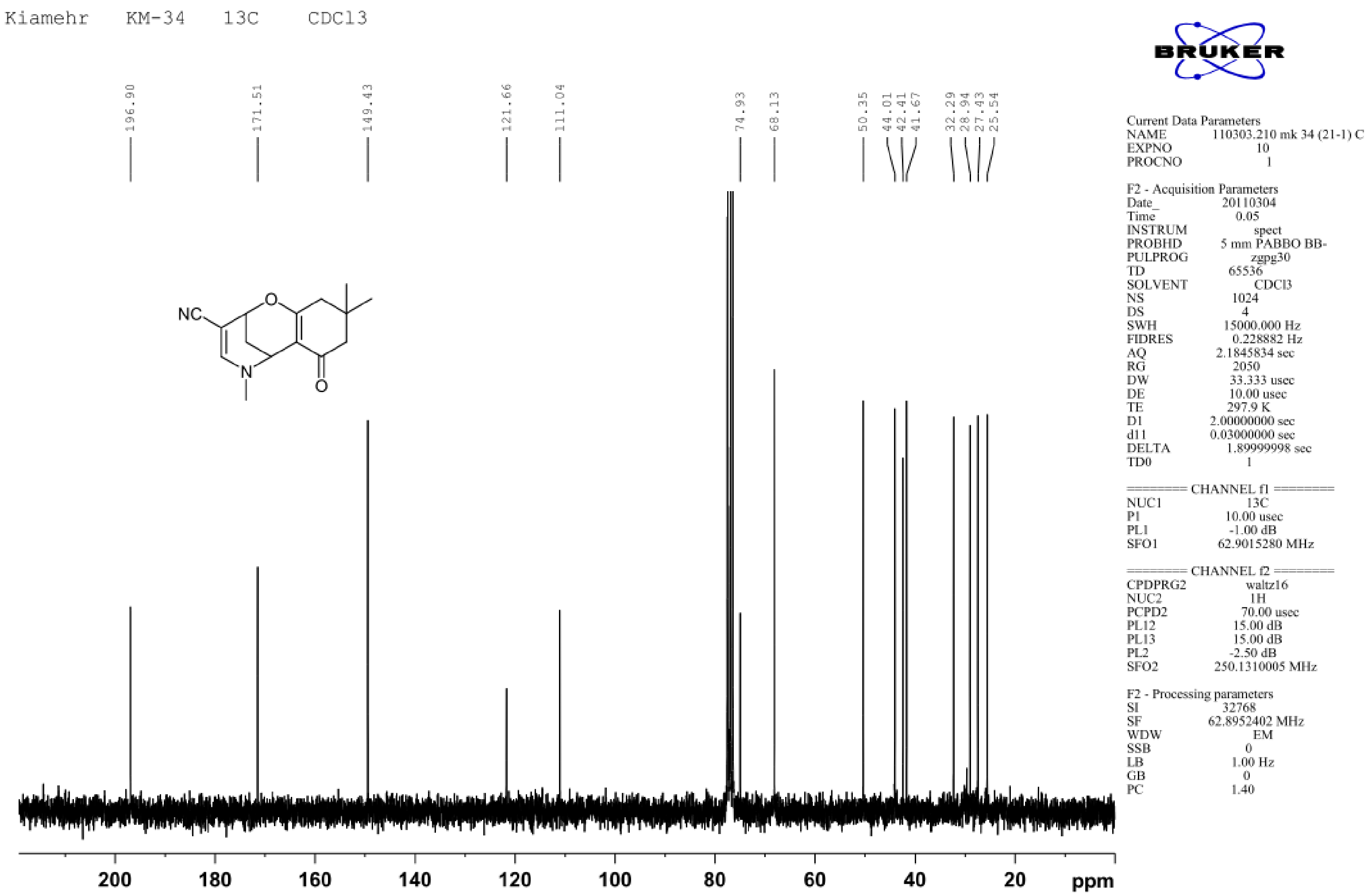

^{13}C NMR spectra for compound 5

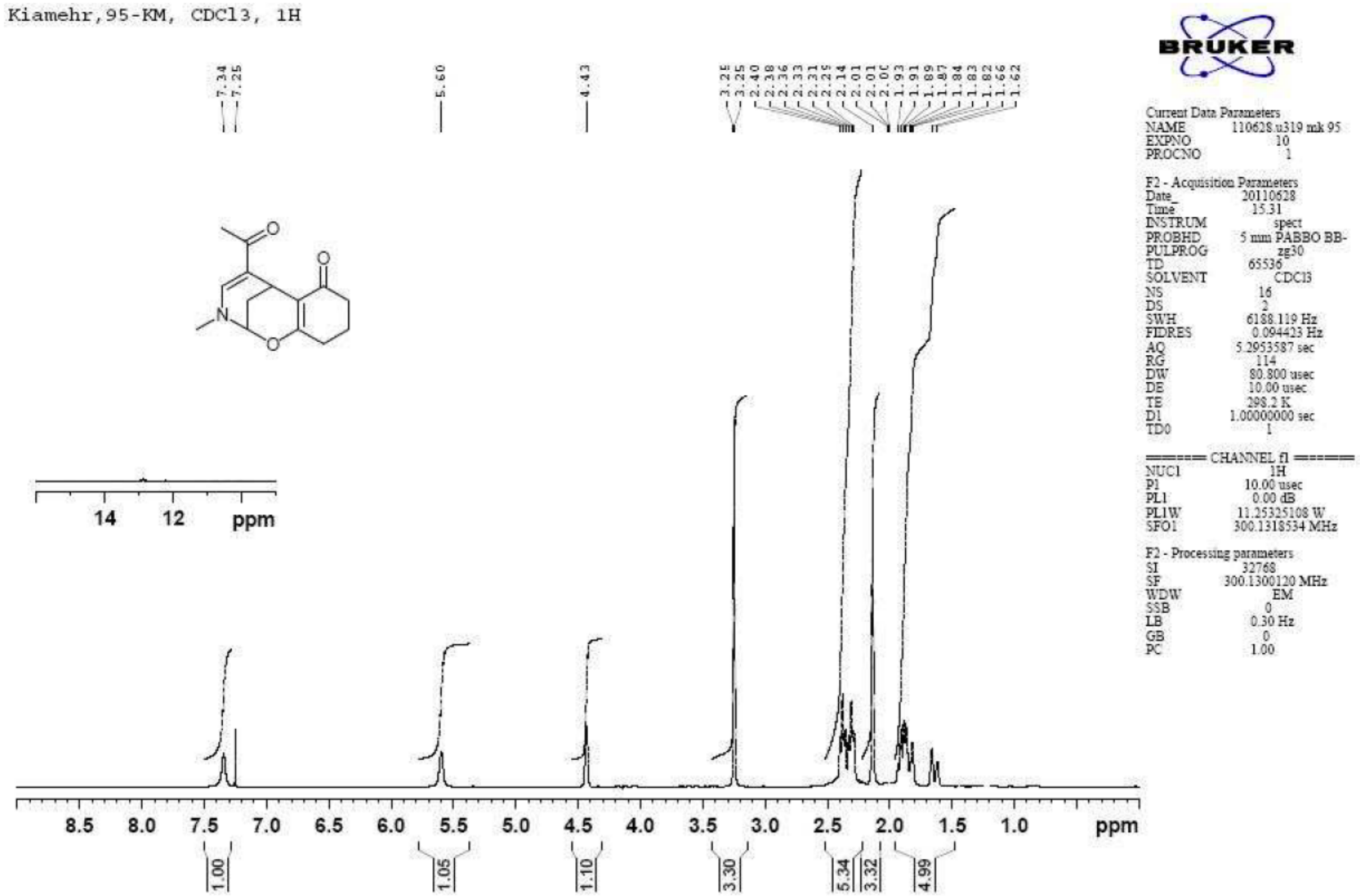

^{1}H NMR spectra for compound 6a

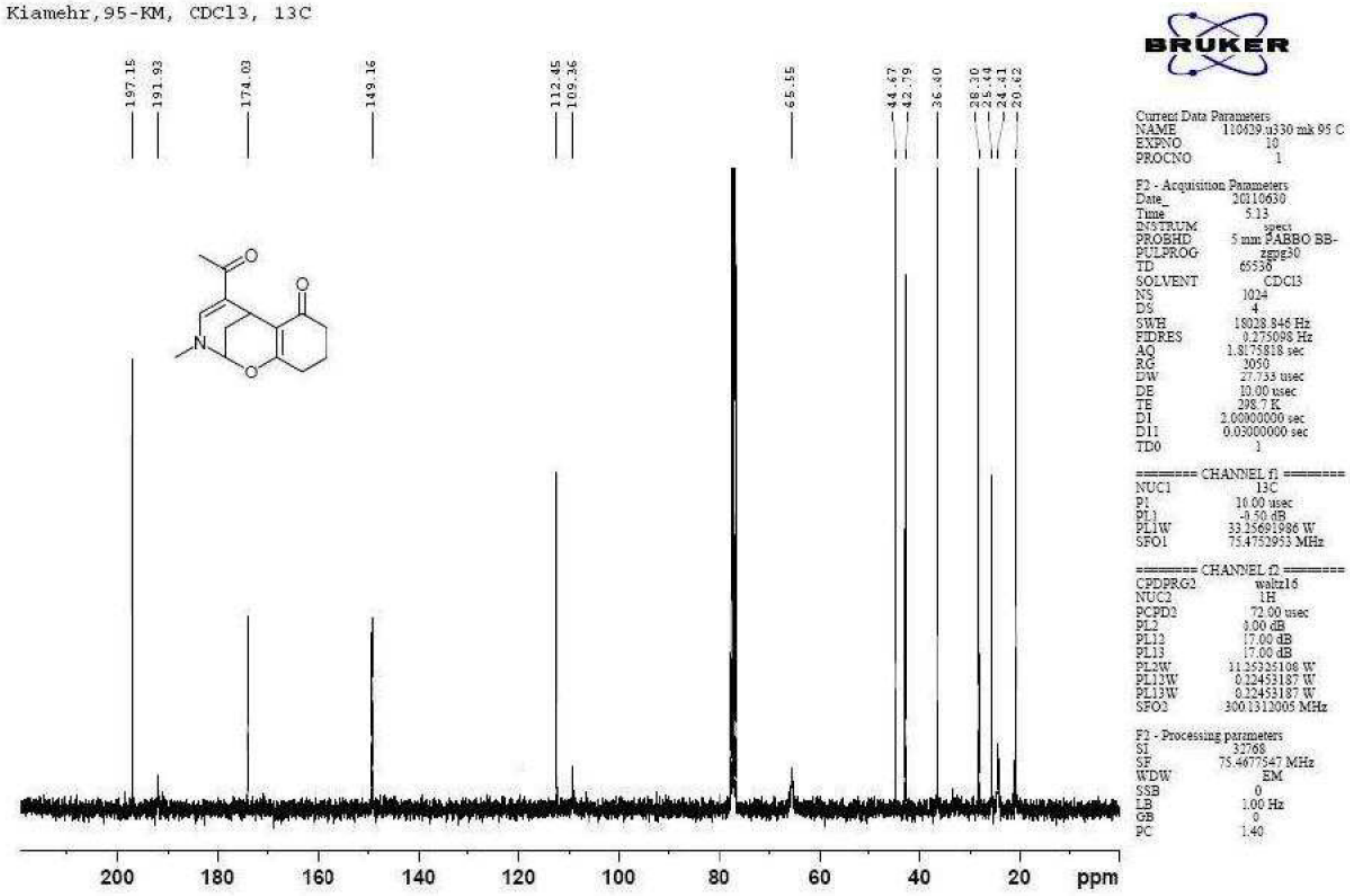

^{13}C NMR spectra for compound 6a

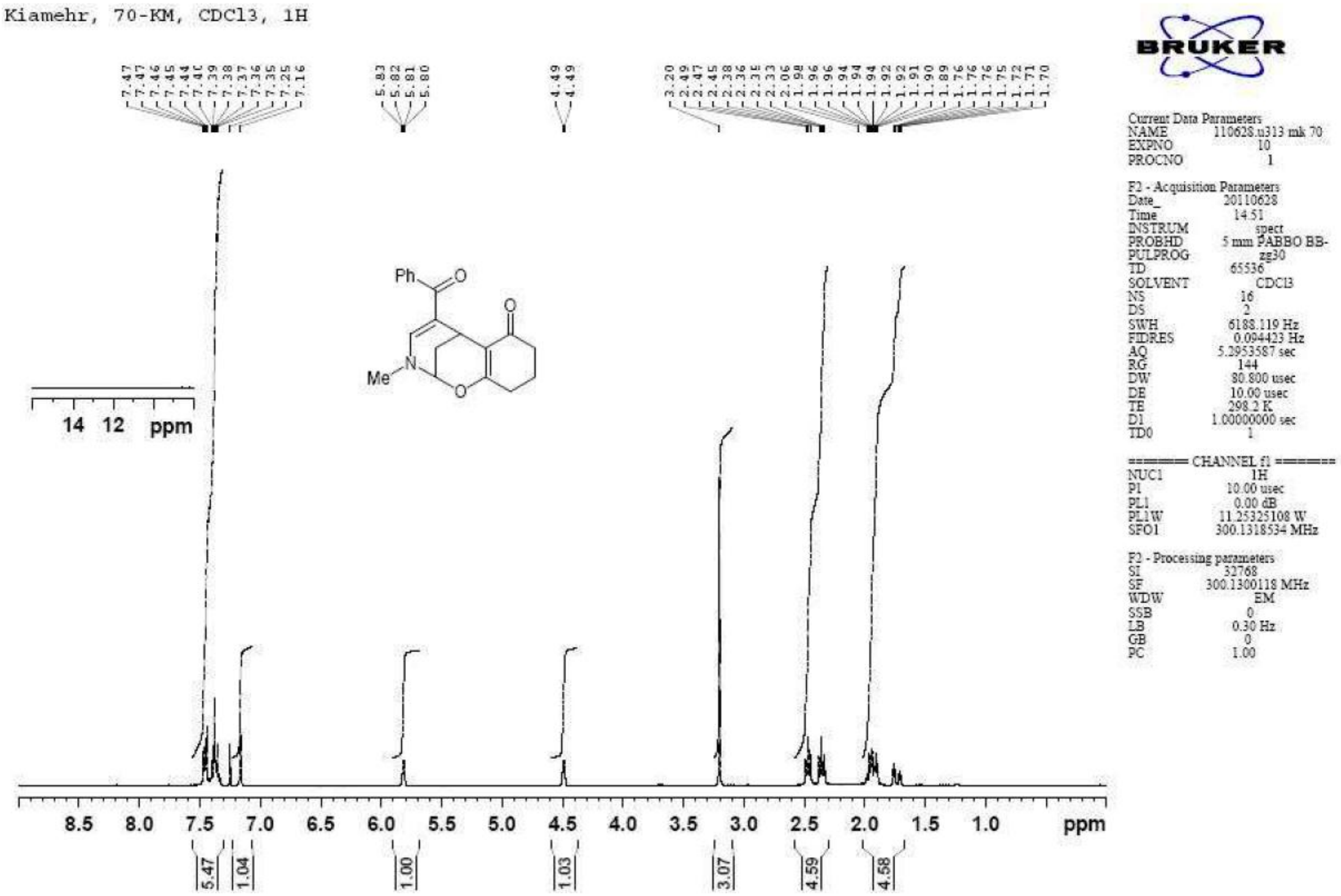

^{1}H NMR spectra for compound 6b

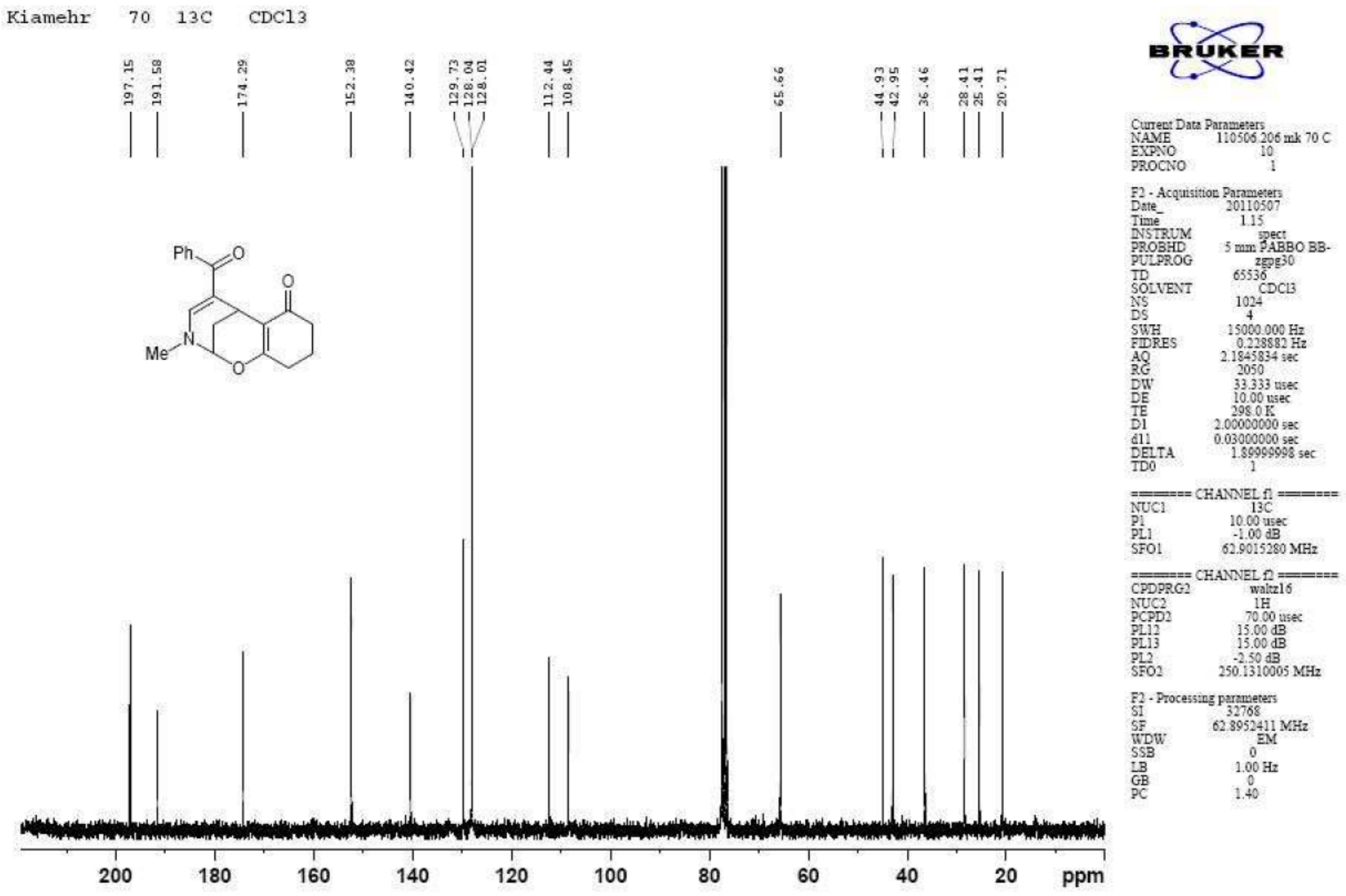

^{13}C NMR spectra for compound 6b

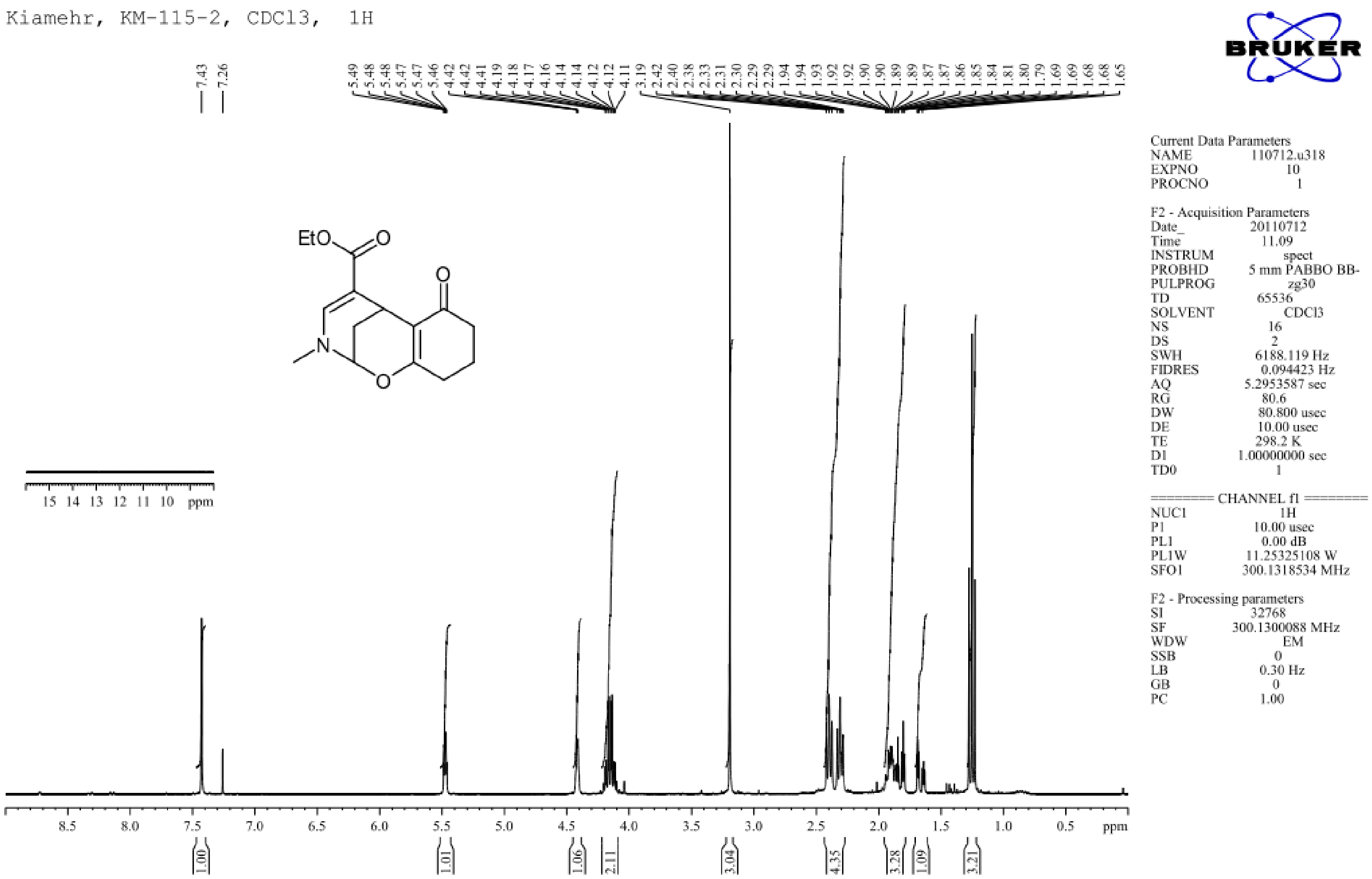

^{1}H NMR spectra for compound 6c

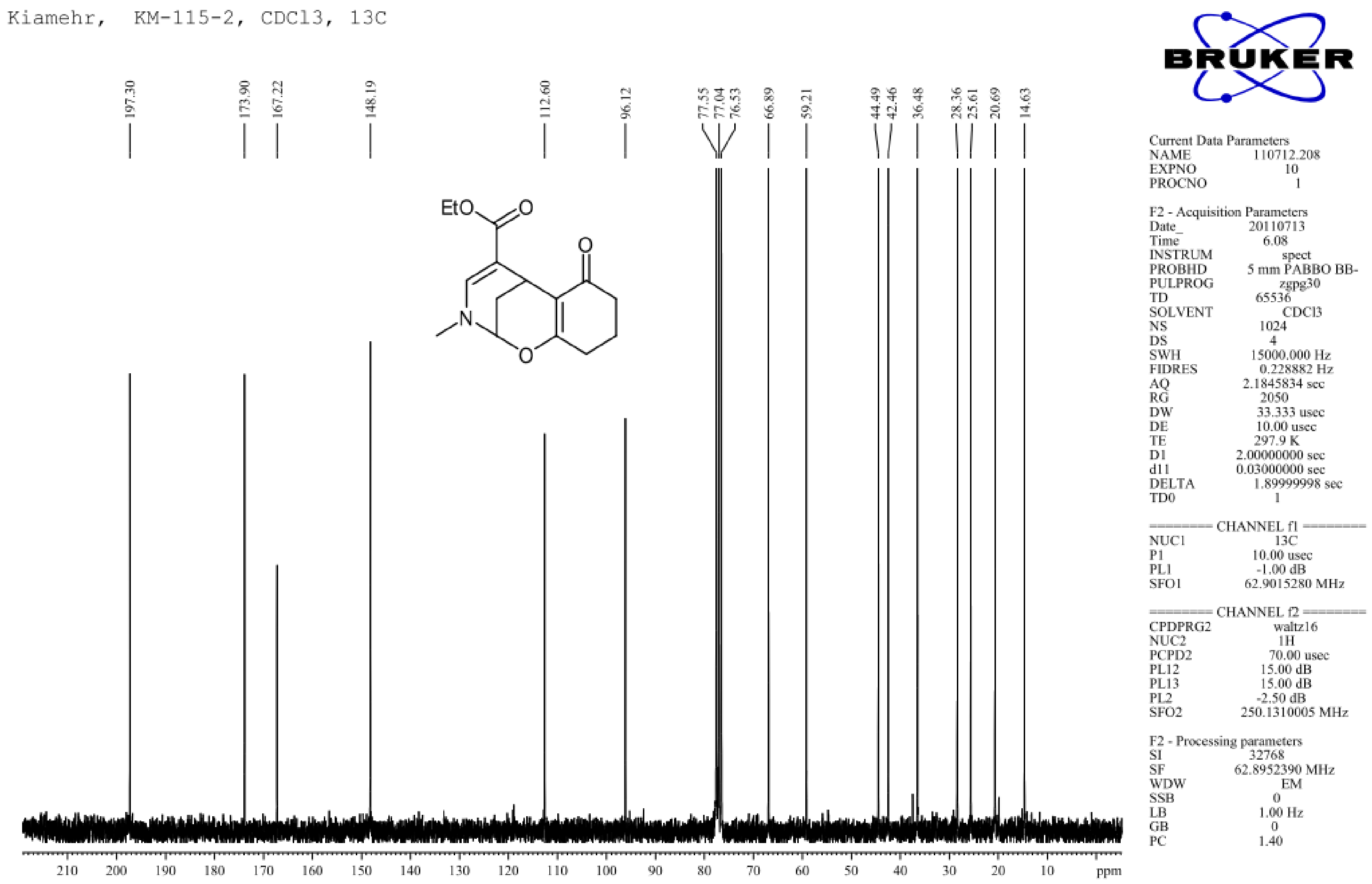

^{13}C NMR spectra for compound 6c

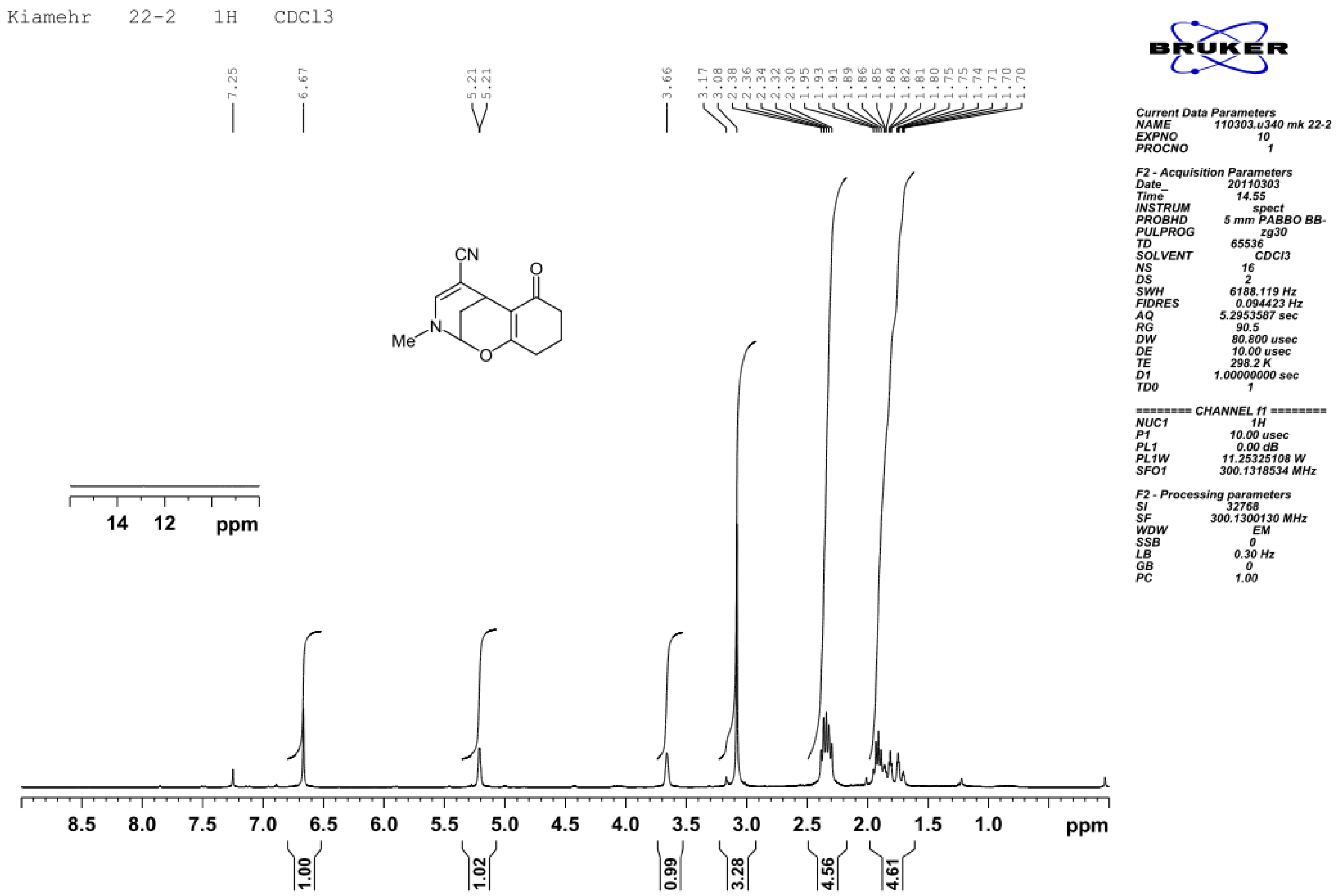

^{1}H NMR spectra for compound 6d

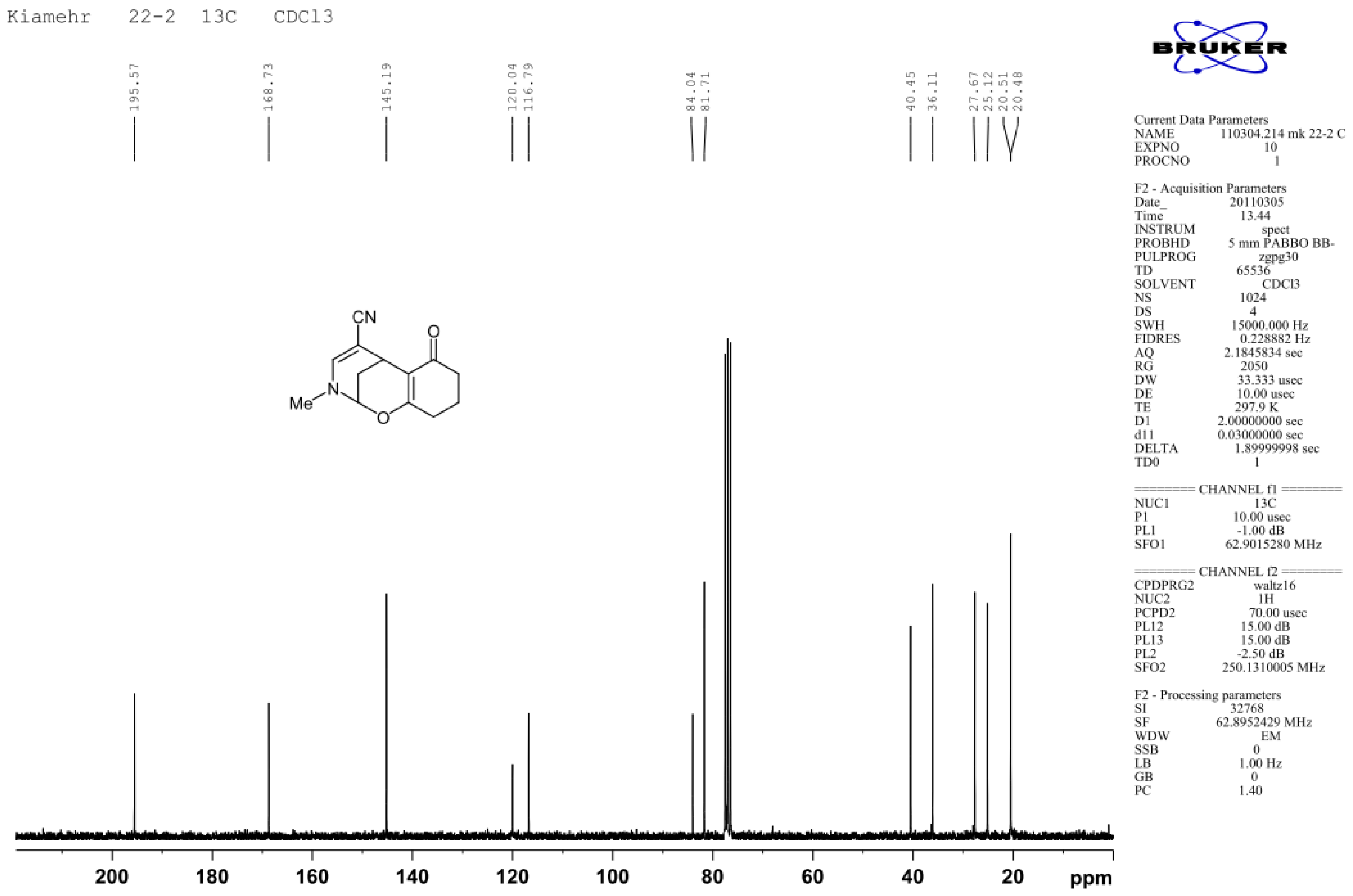

^{13}C NMR spectra for compound 6d

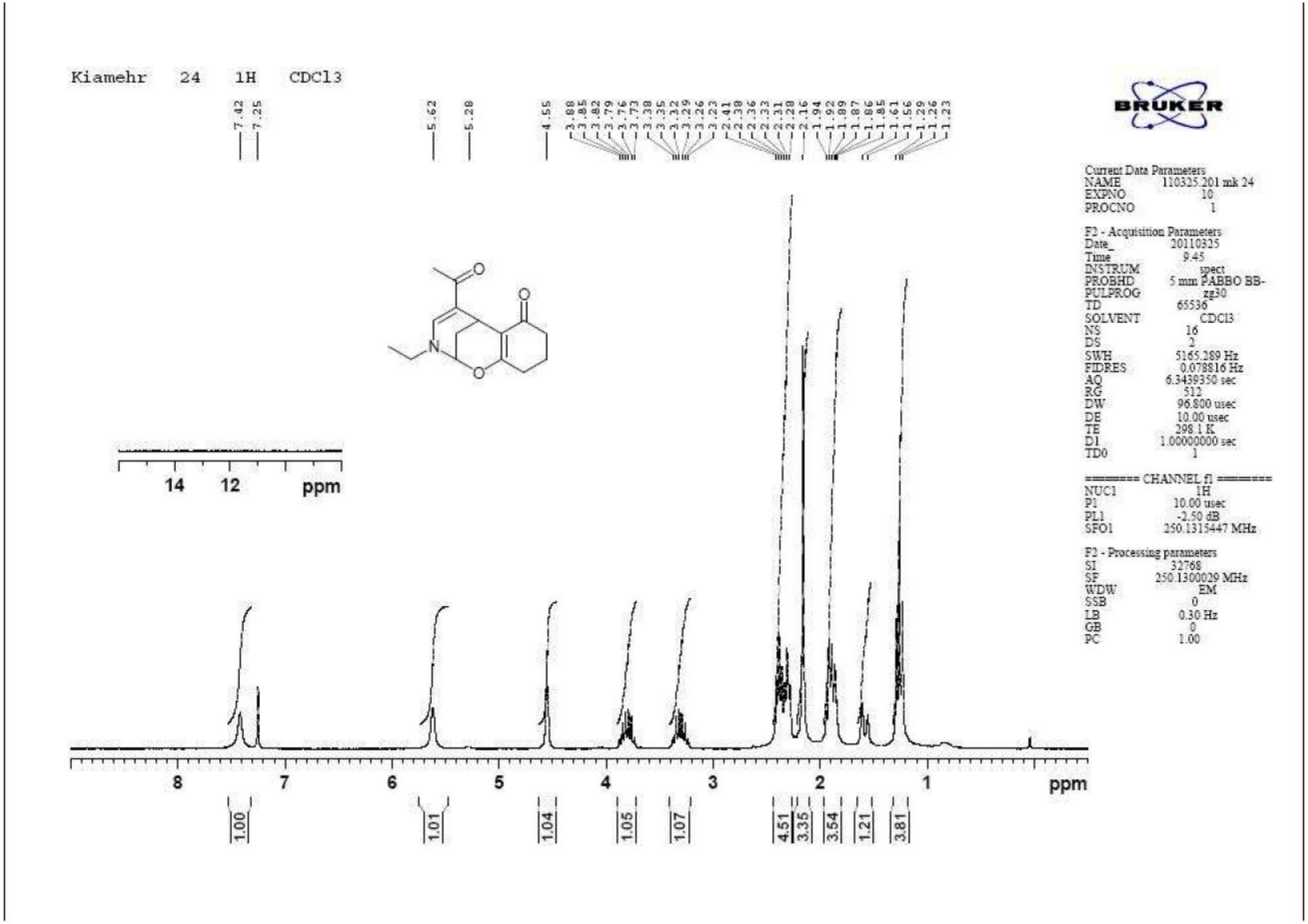

^{1}H NMR spectra for compound 6e

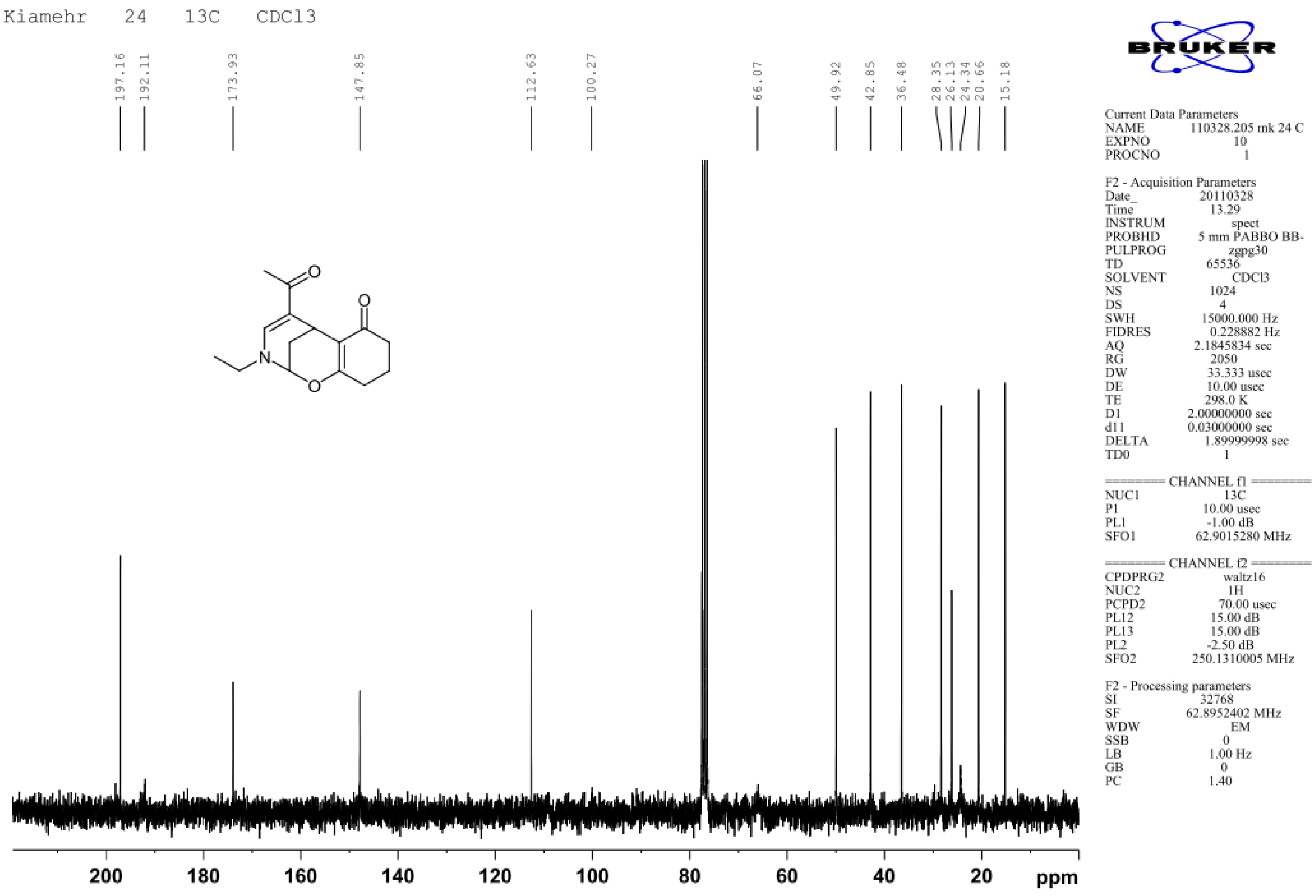

^{13}C NMR spectra for compound 6e

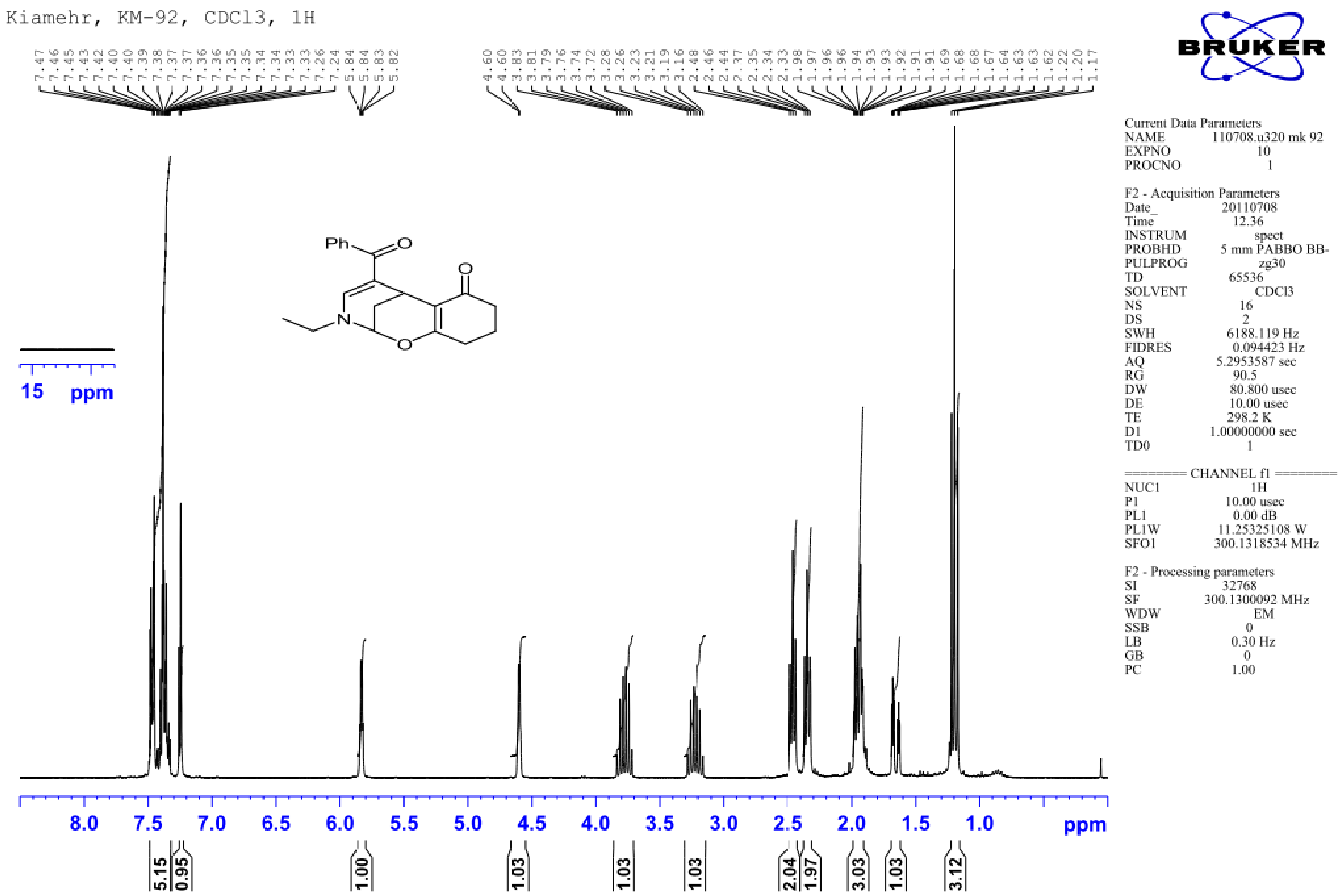

^{1}H NMR spectra for compound 6f

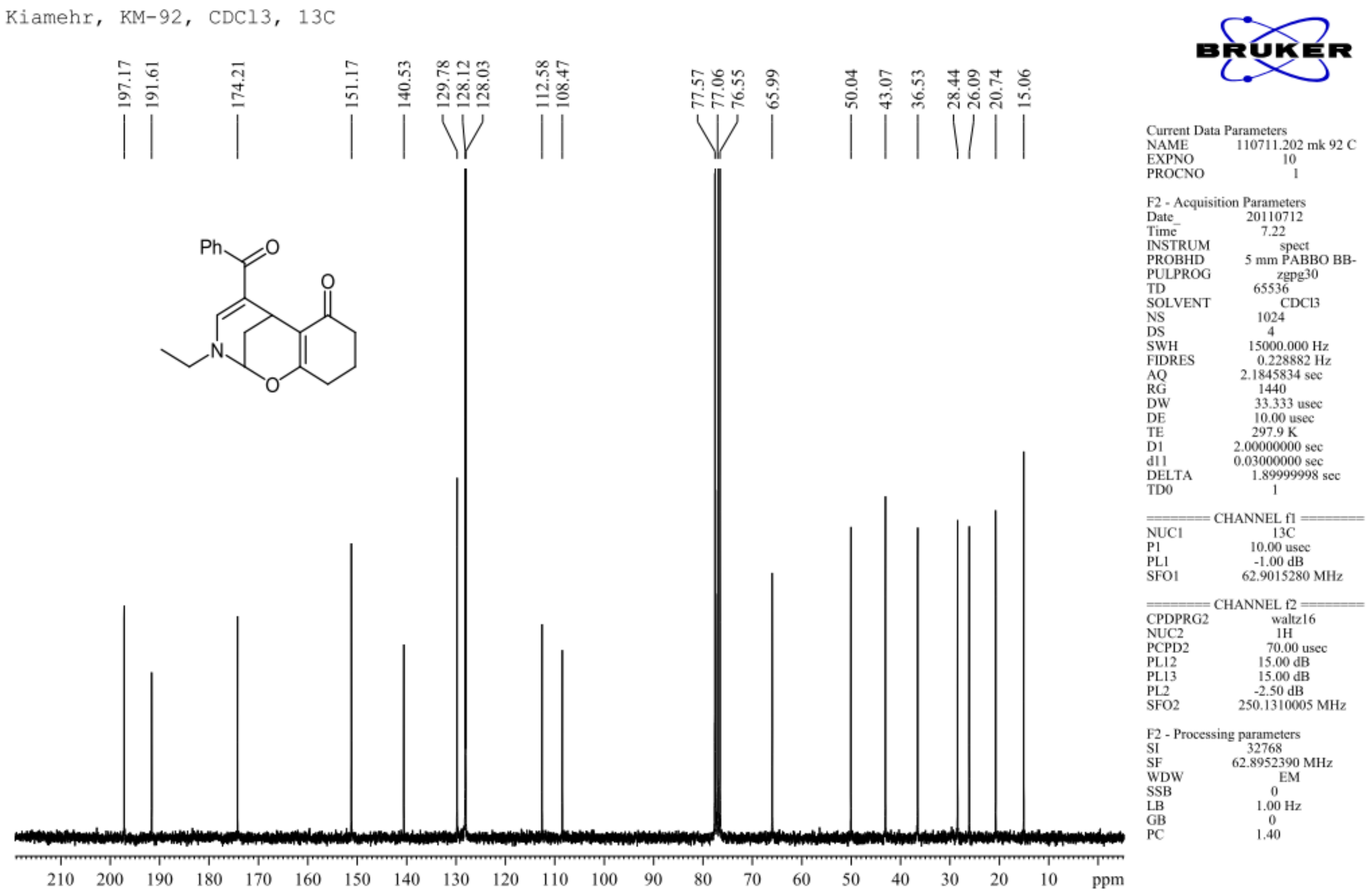

^{13}C NMR spectra for compound 6f

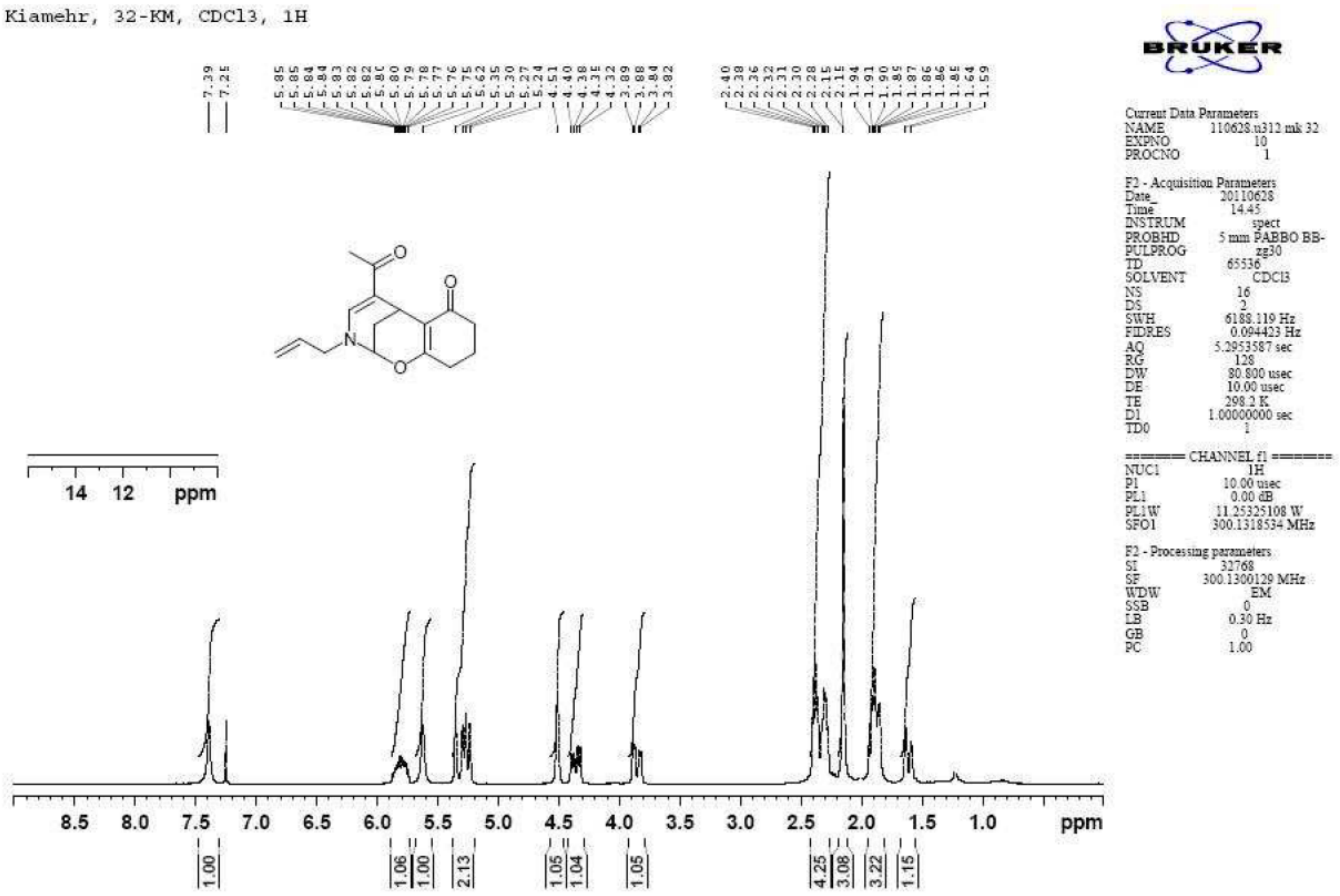

^{1}H NMR spectra for compound 6g

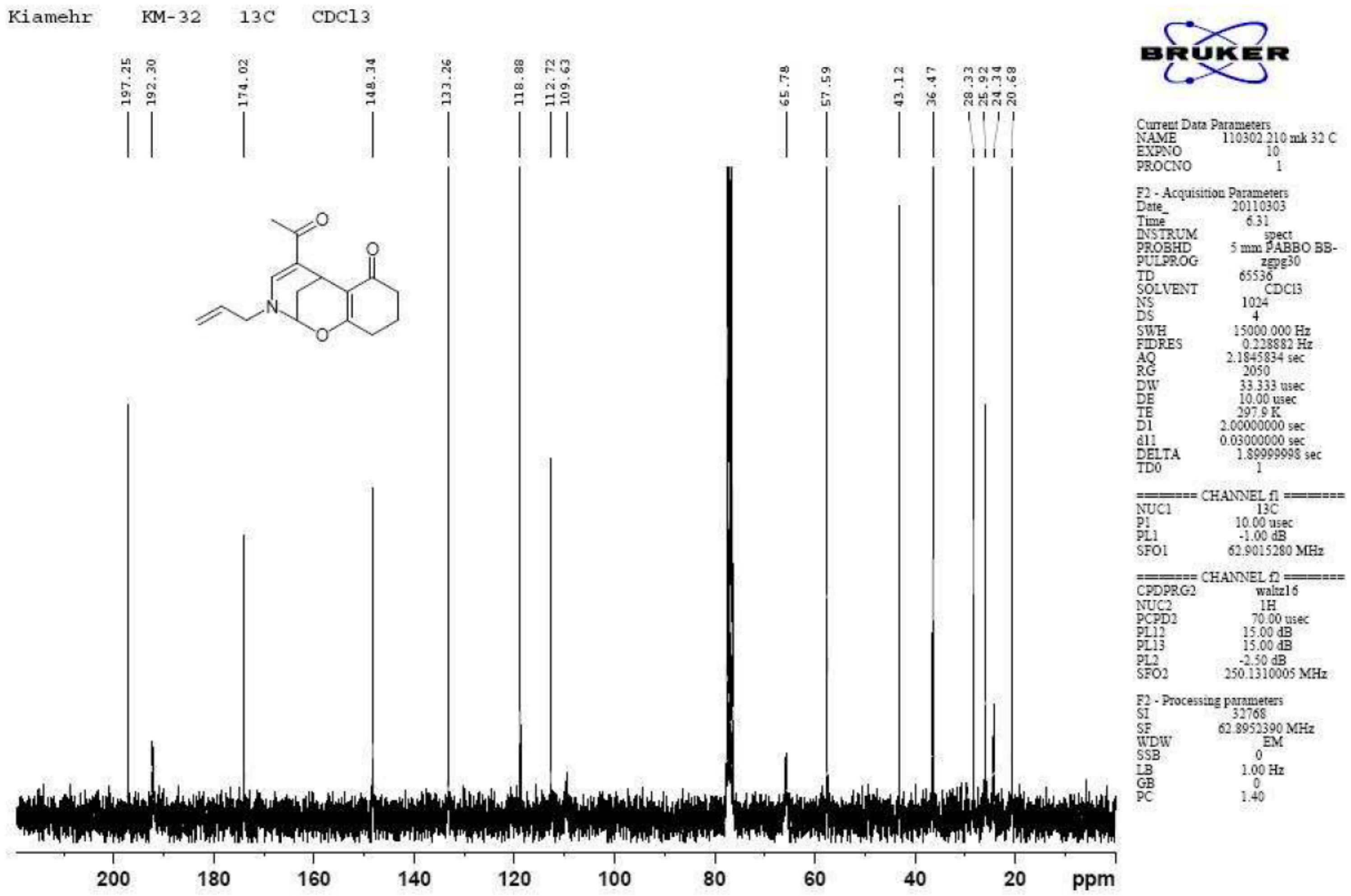

^{13}C NMR spectra for compound 6g

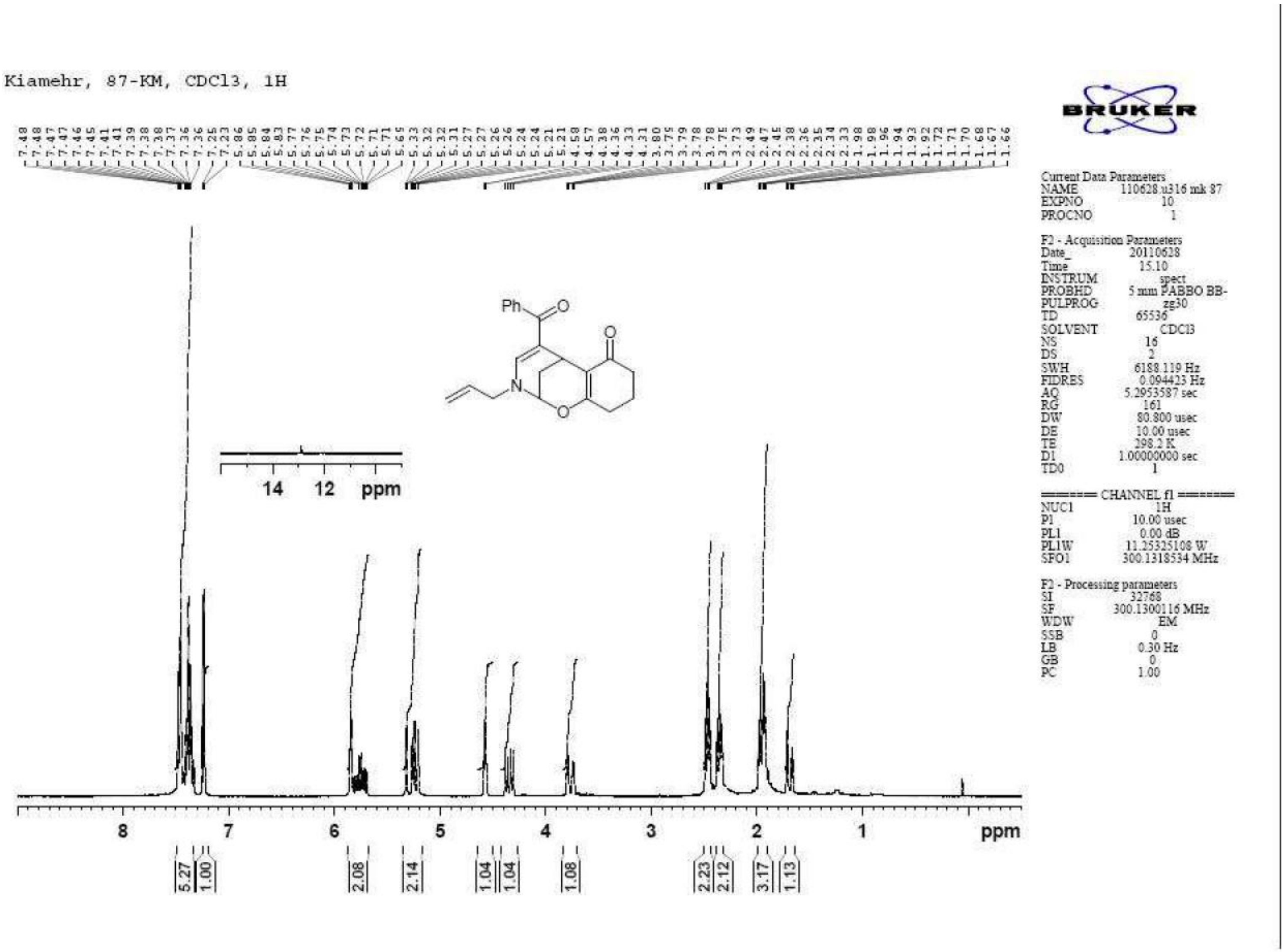

^{1}H NMR spectra for compound 6h

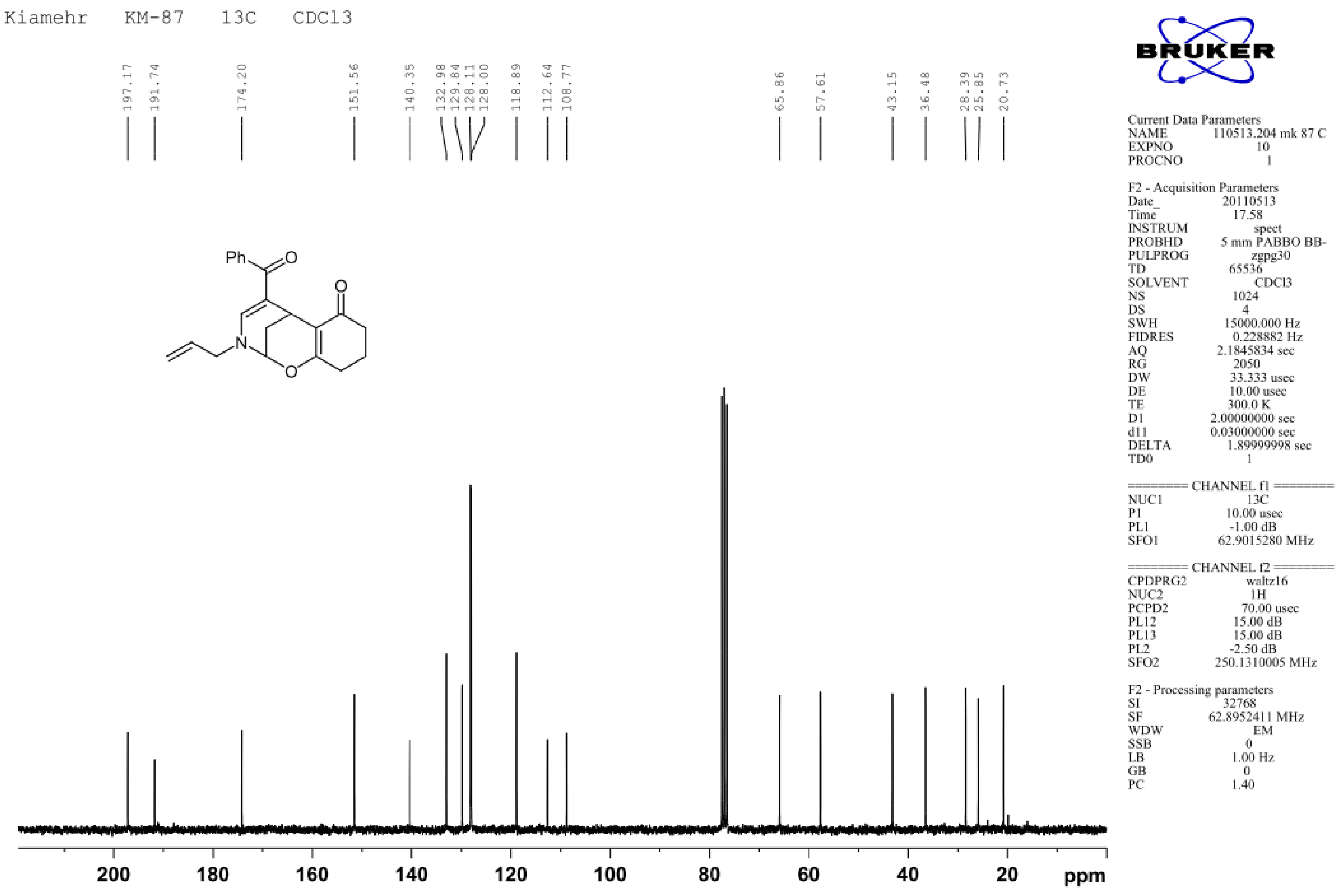

^{13}C NMR spectra for compound 6h

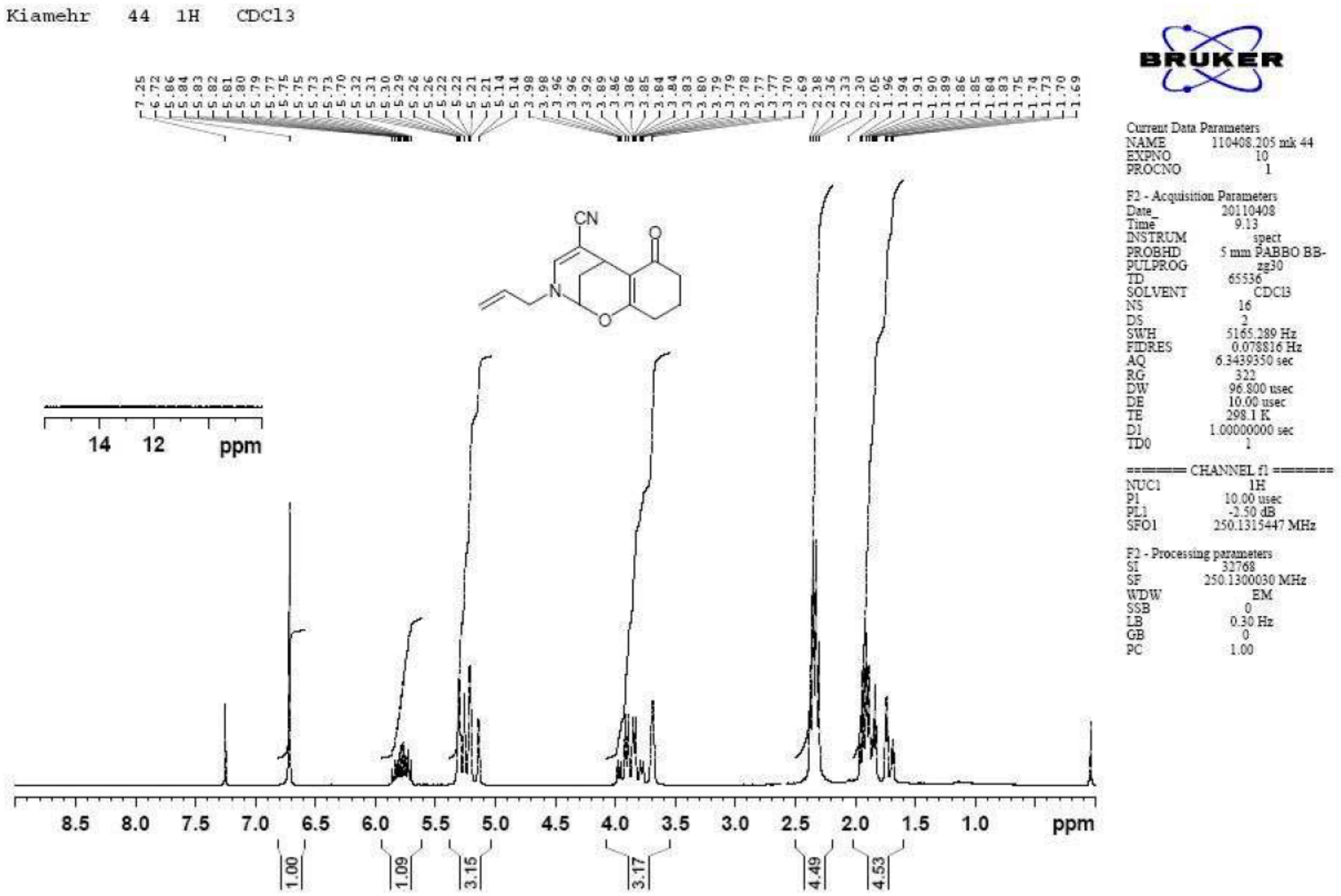

^{1}H NMR spectra for compound 6i

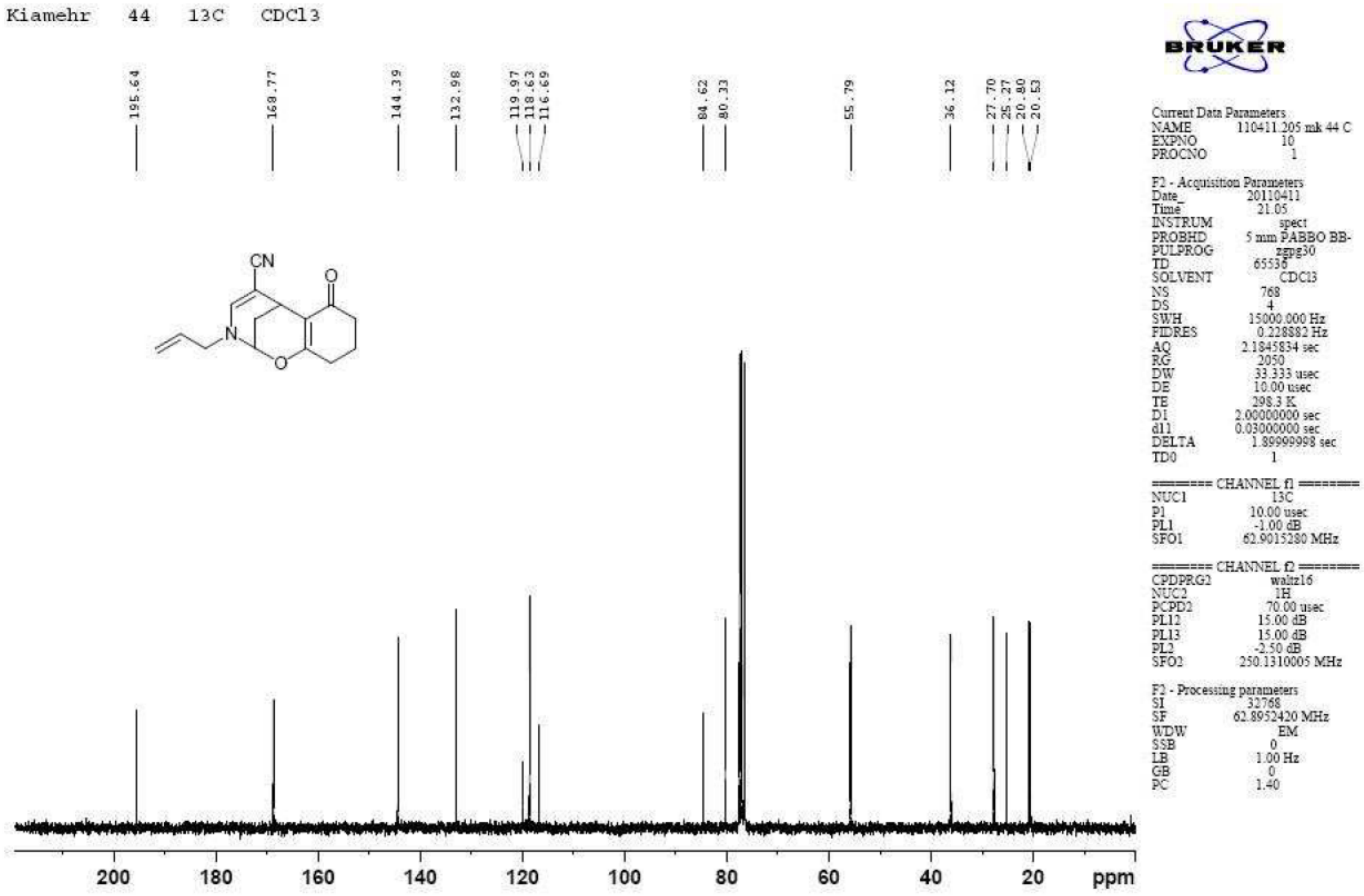

^{13}C NMR spectra for compound 6i

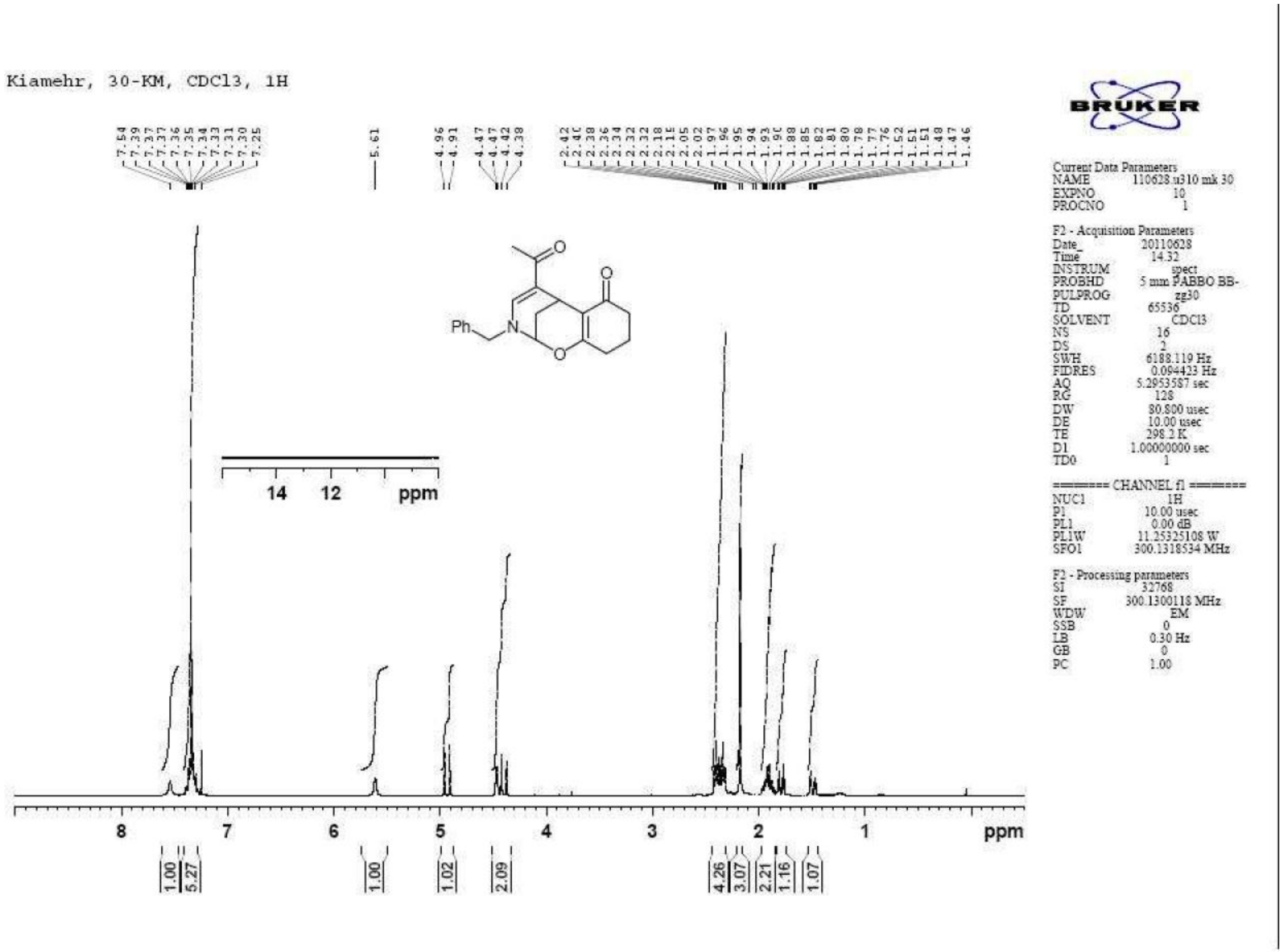

^{1}H NMR spectra for compound 6j

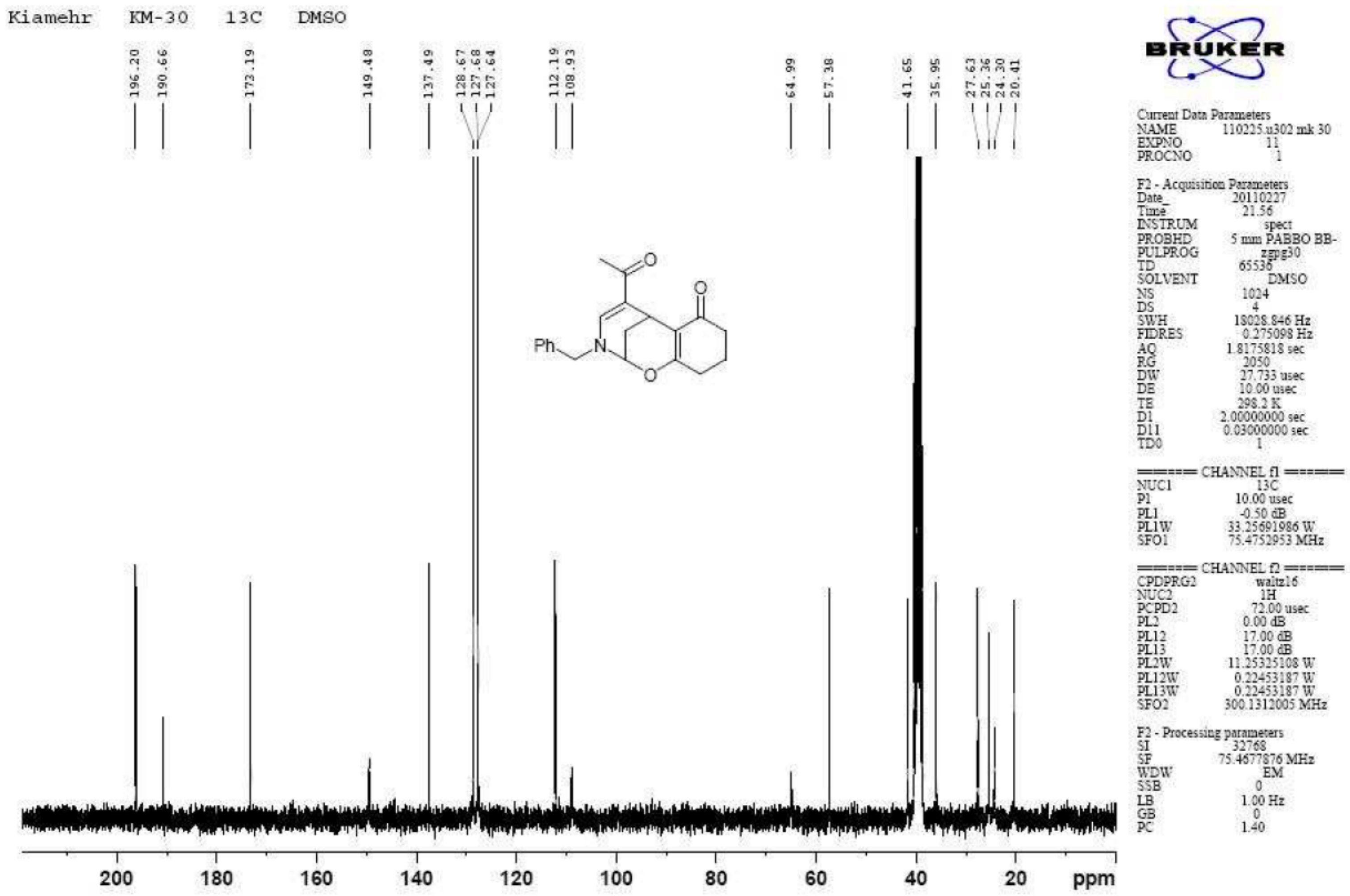

^{13}C NMR spectra for compound 6j

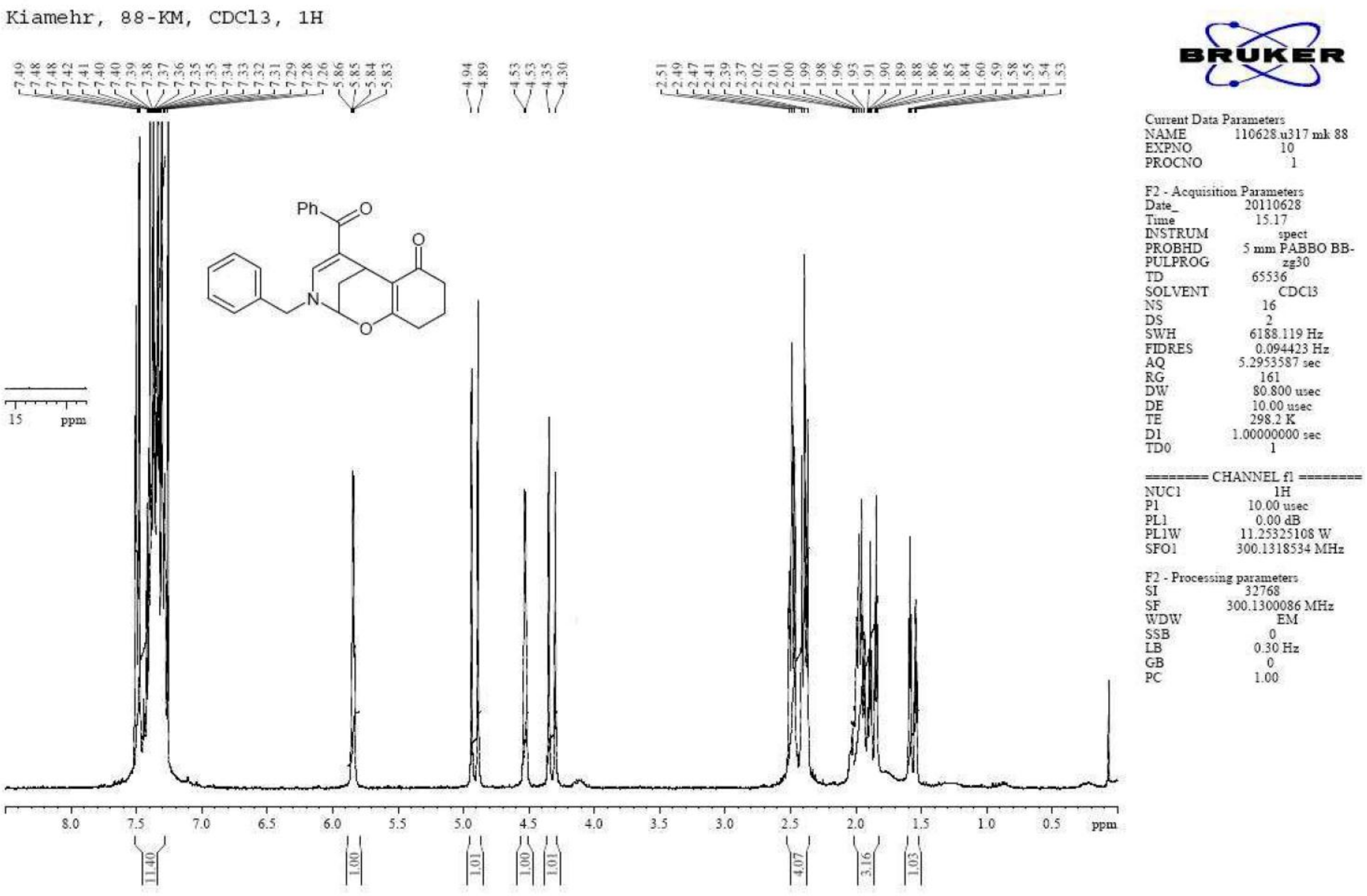

^{1}H NMR spectra for compound 6k

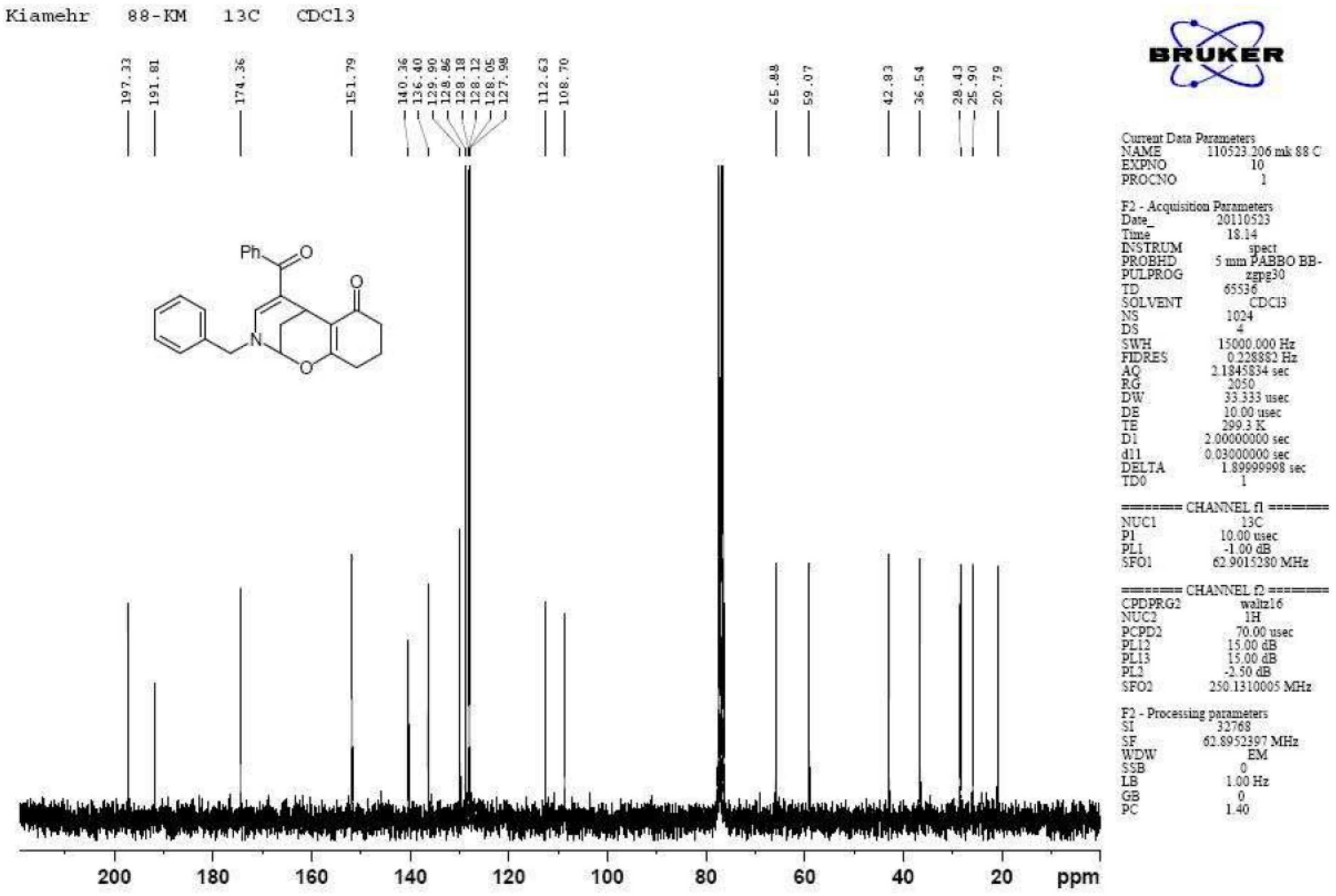

^{13}C NMR spectra for compound 6k

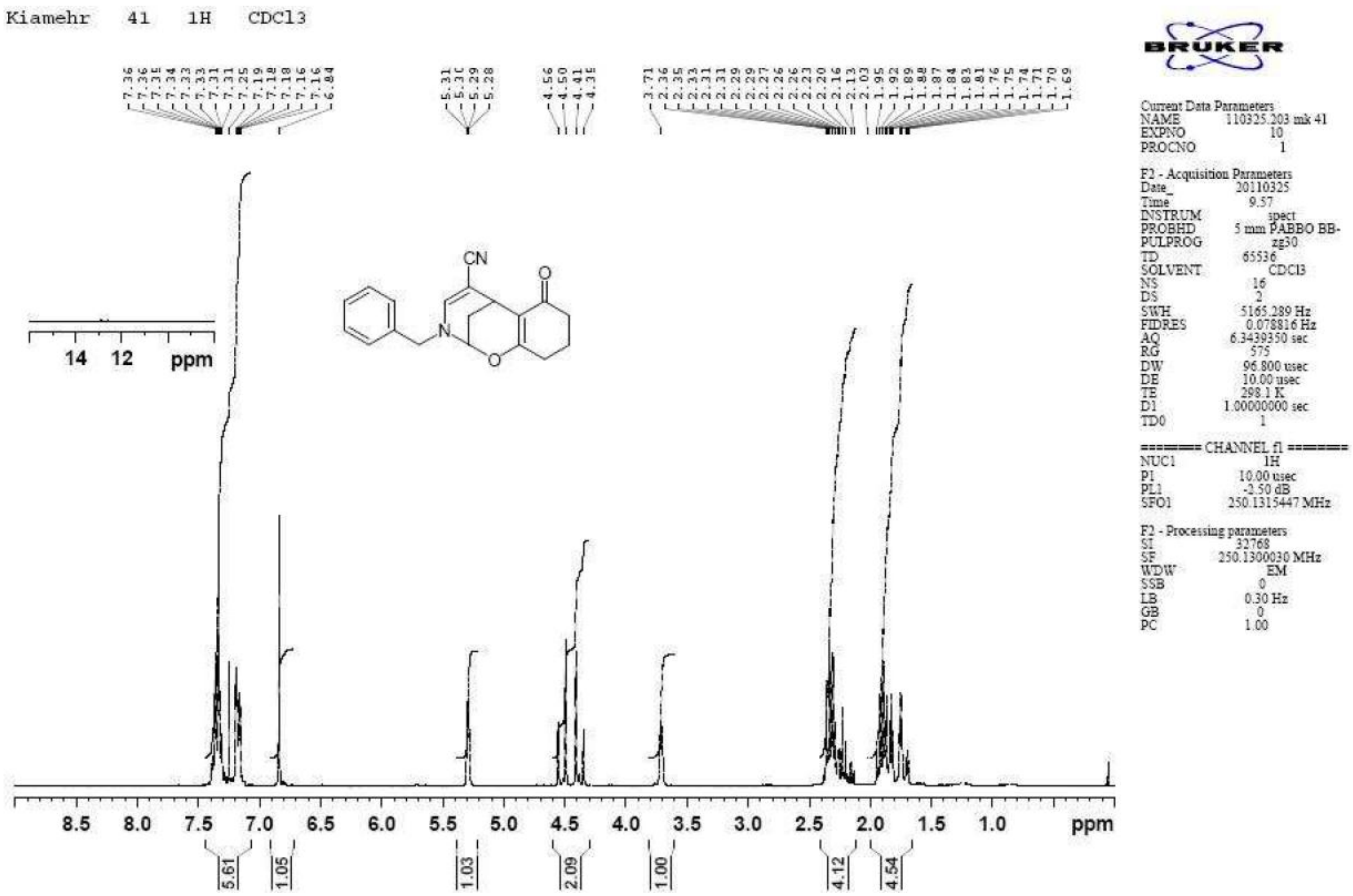

^{1}H NMR spectra for compound 6l

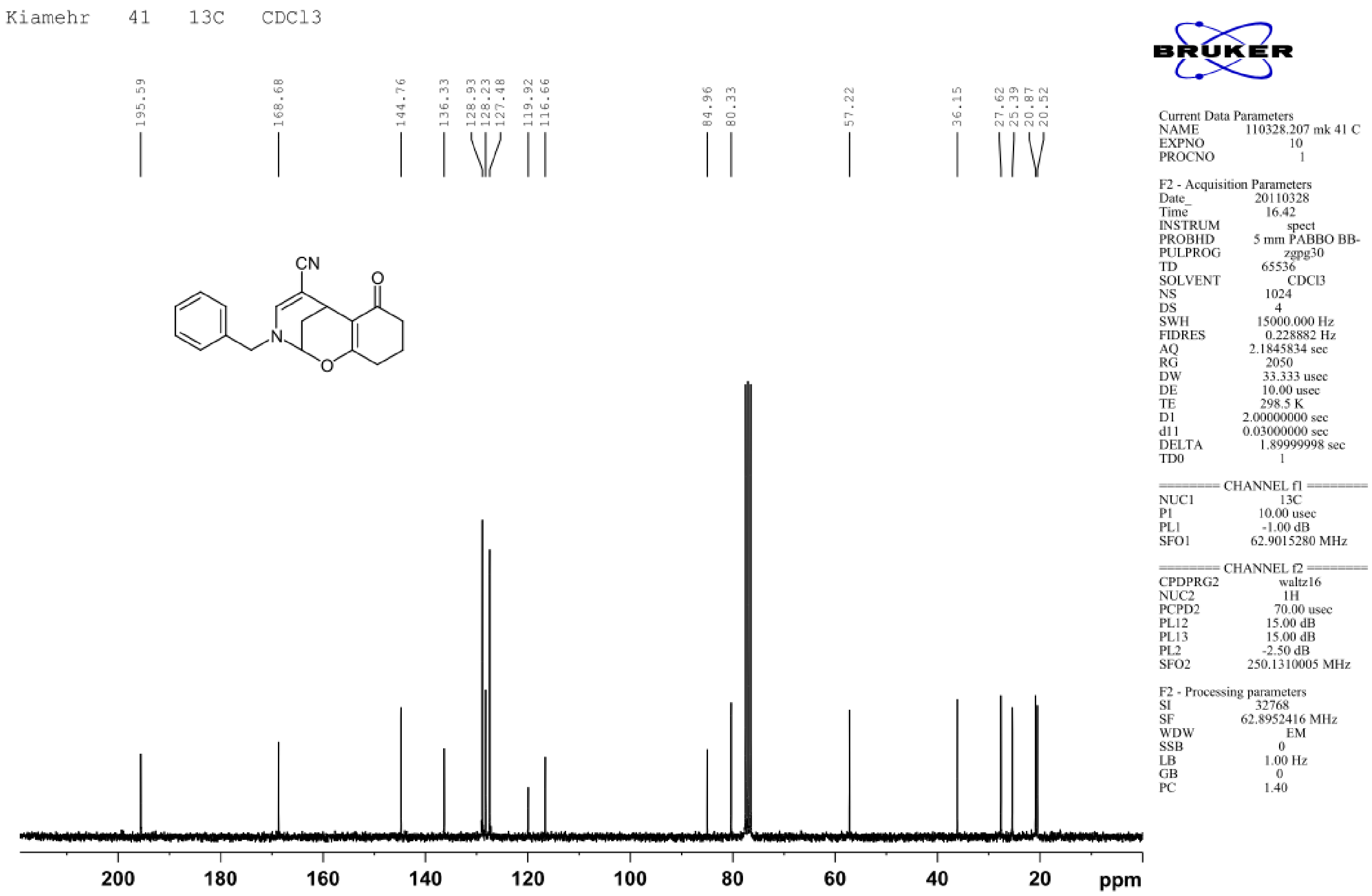

^{13}C NMR spectra for compound 6l

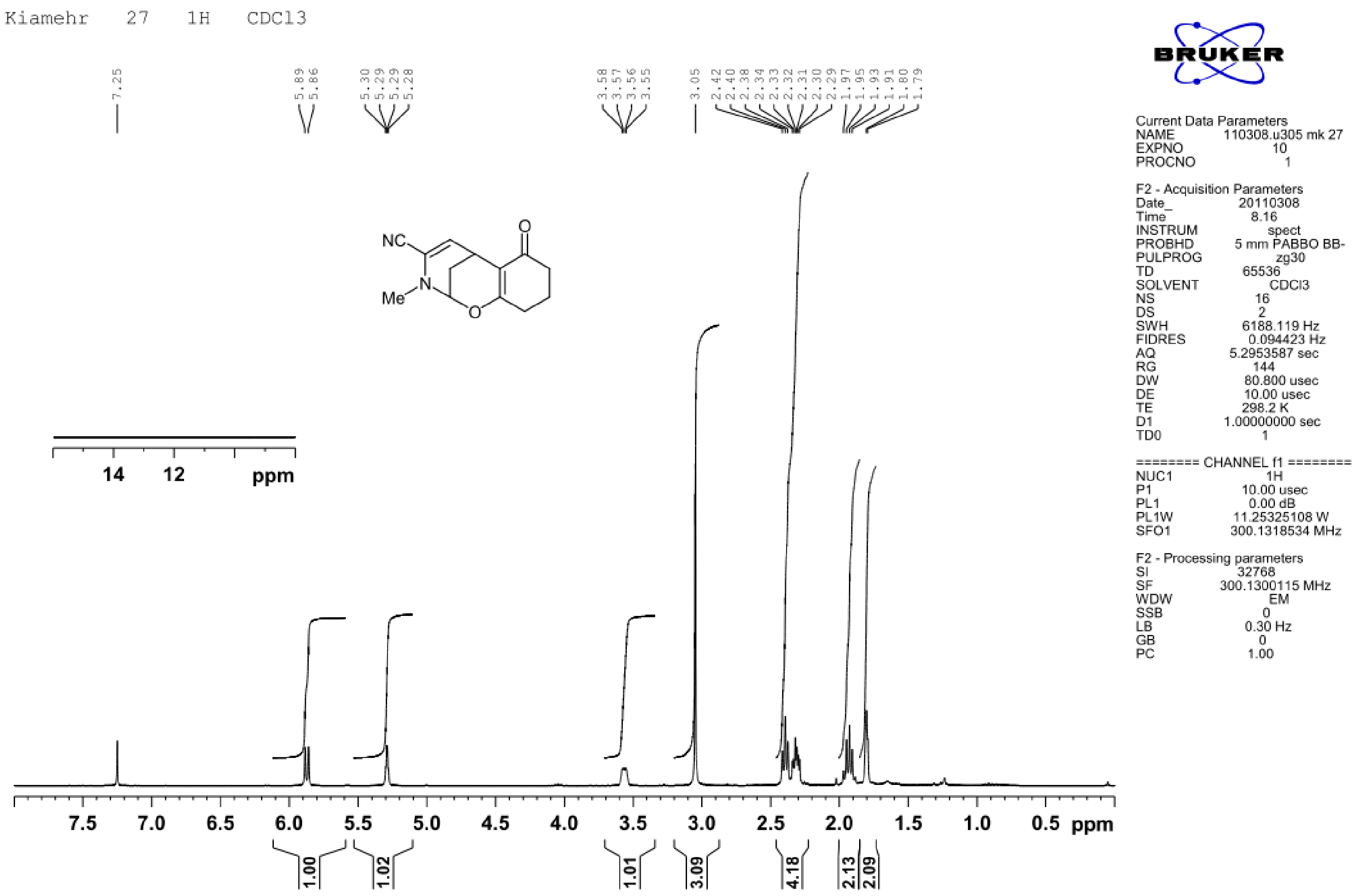

^{1}H NMR spectra for compound 7a

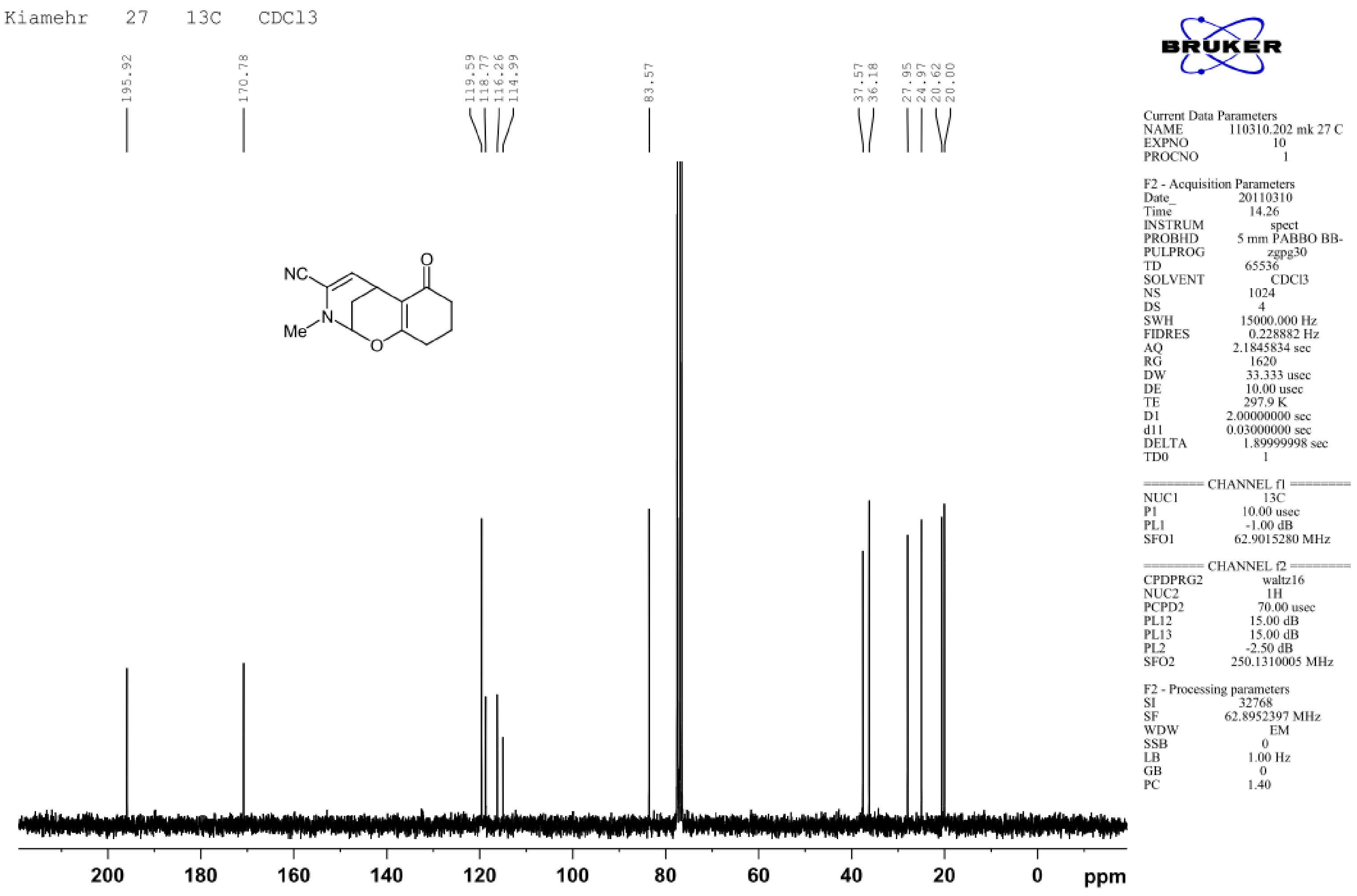

^{13}C NMR spectra for compound 7a

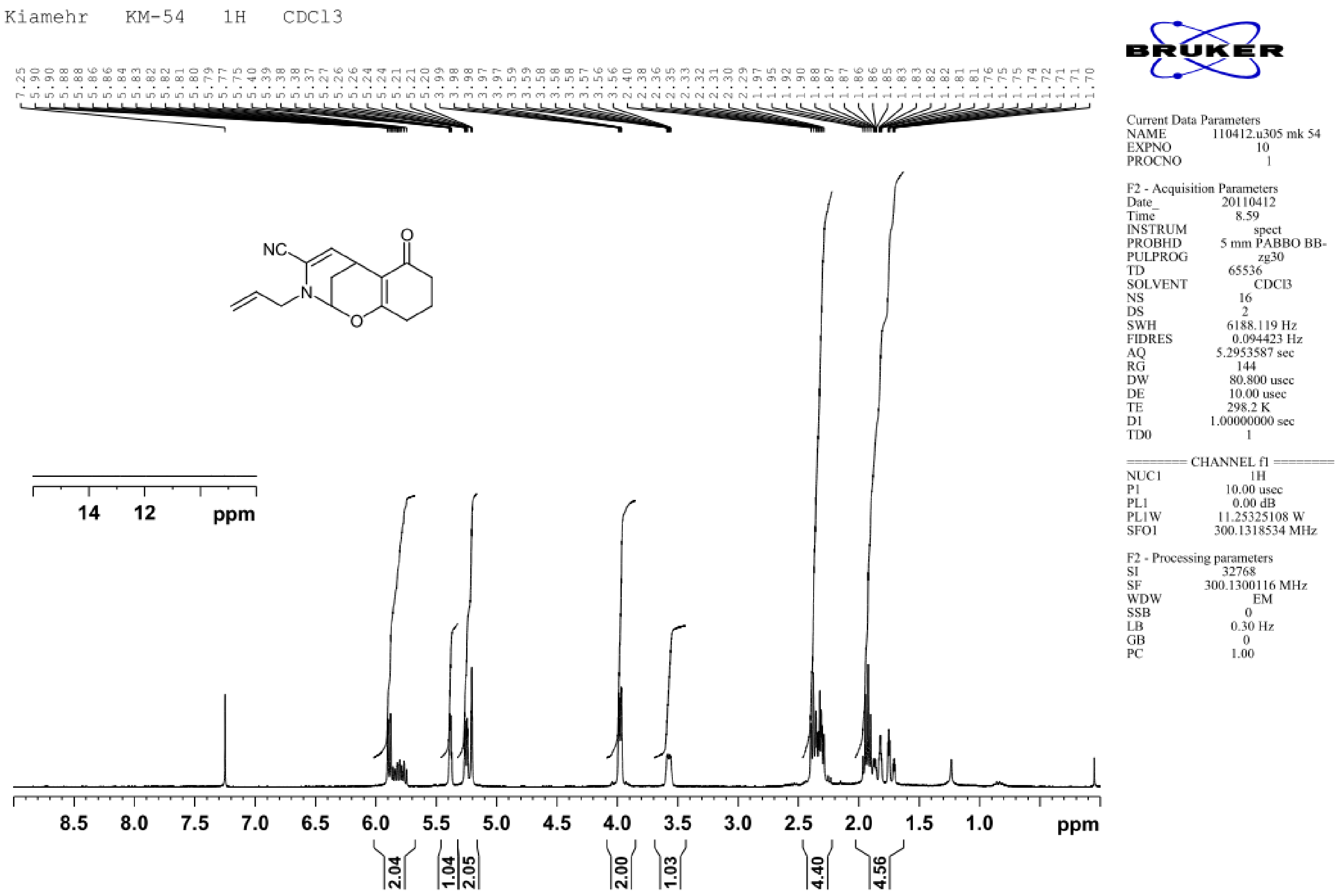

^{1}H NMR spectra for compound 7b

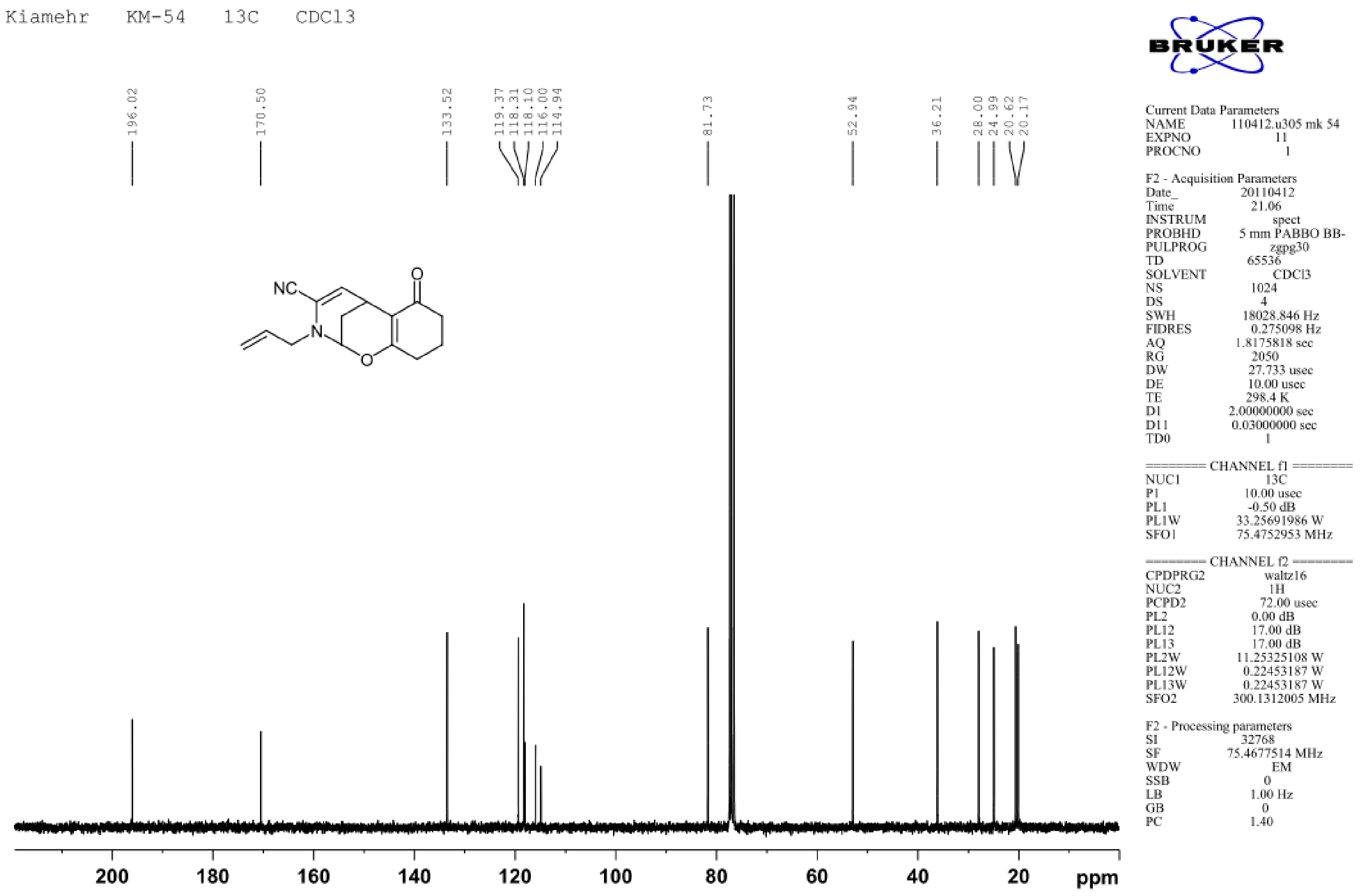

^{13}C NMR spectra for compound 7b

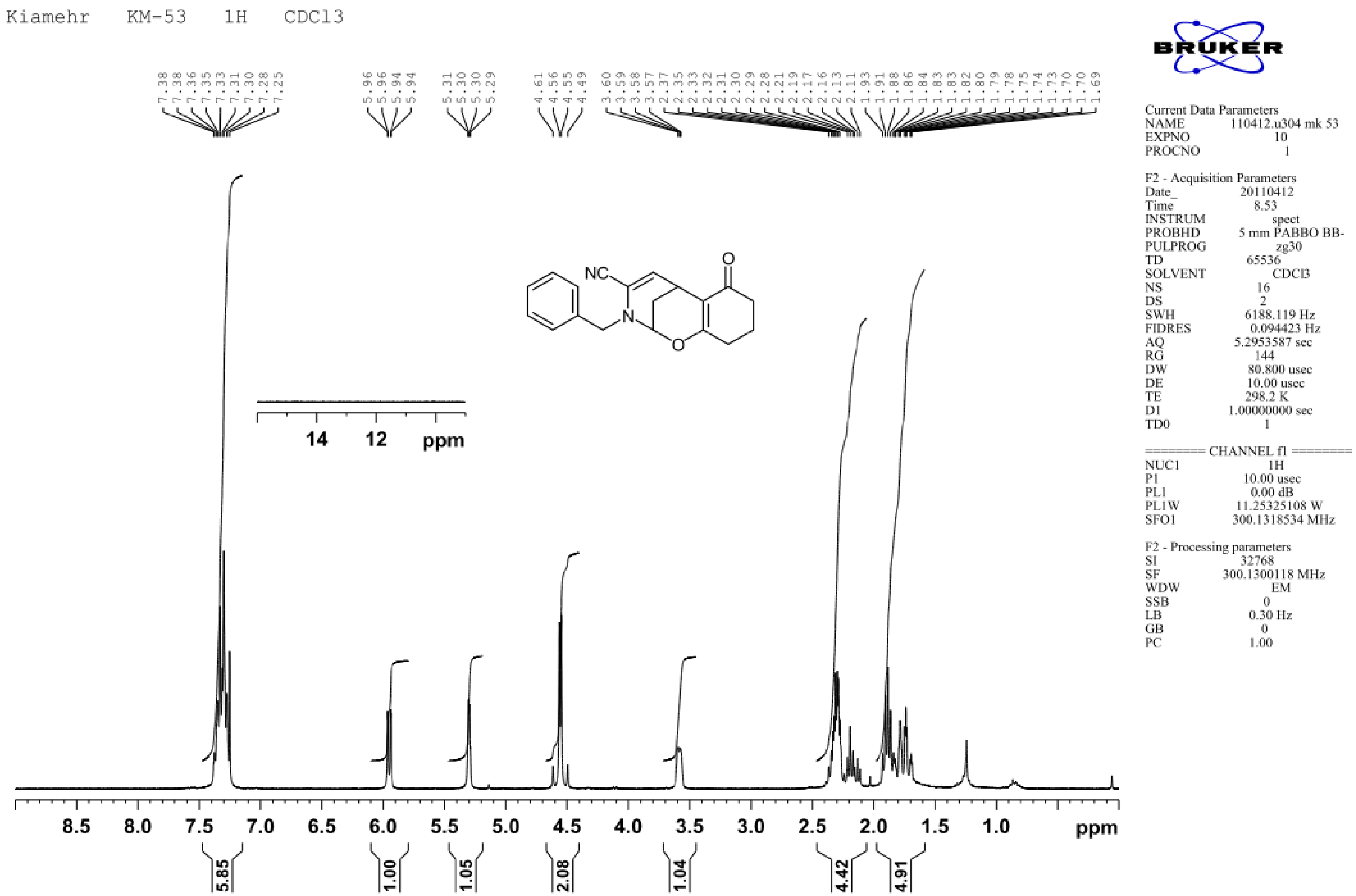

^{1}H NMR spectra for compound 7c

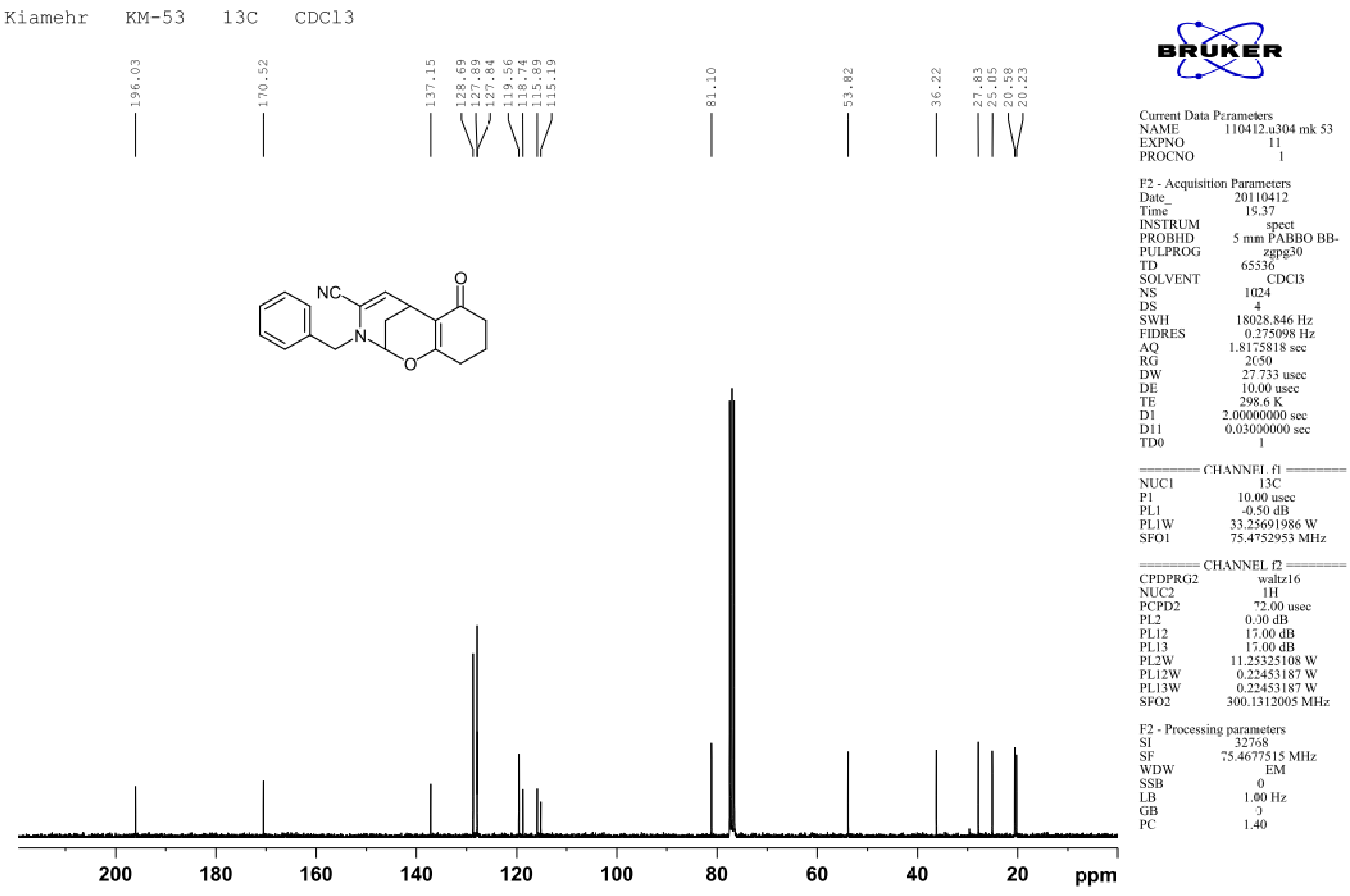

^{13}C NMR spectra for compound 7c

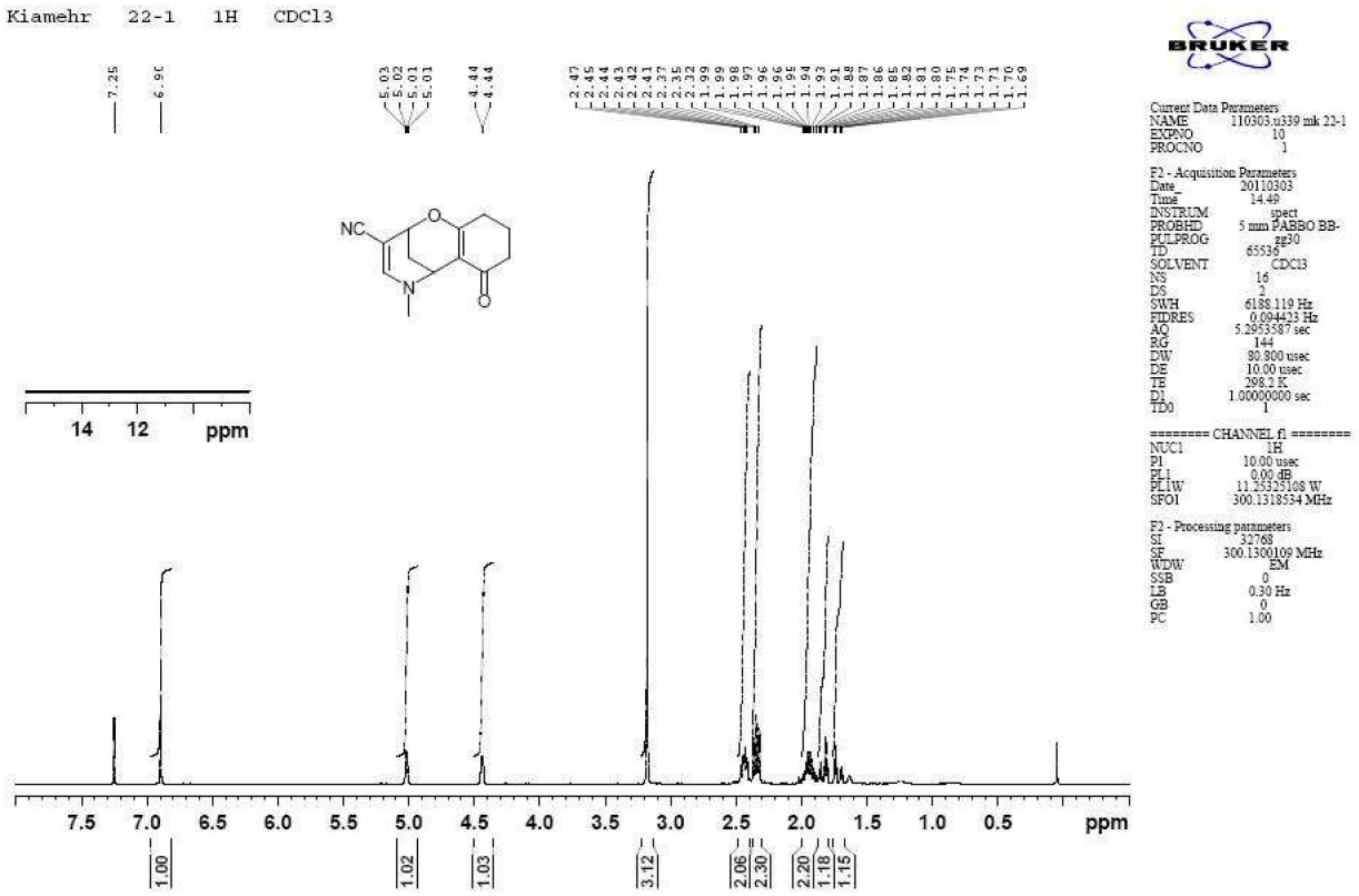

^{1}H NMR spectra for compound 8

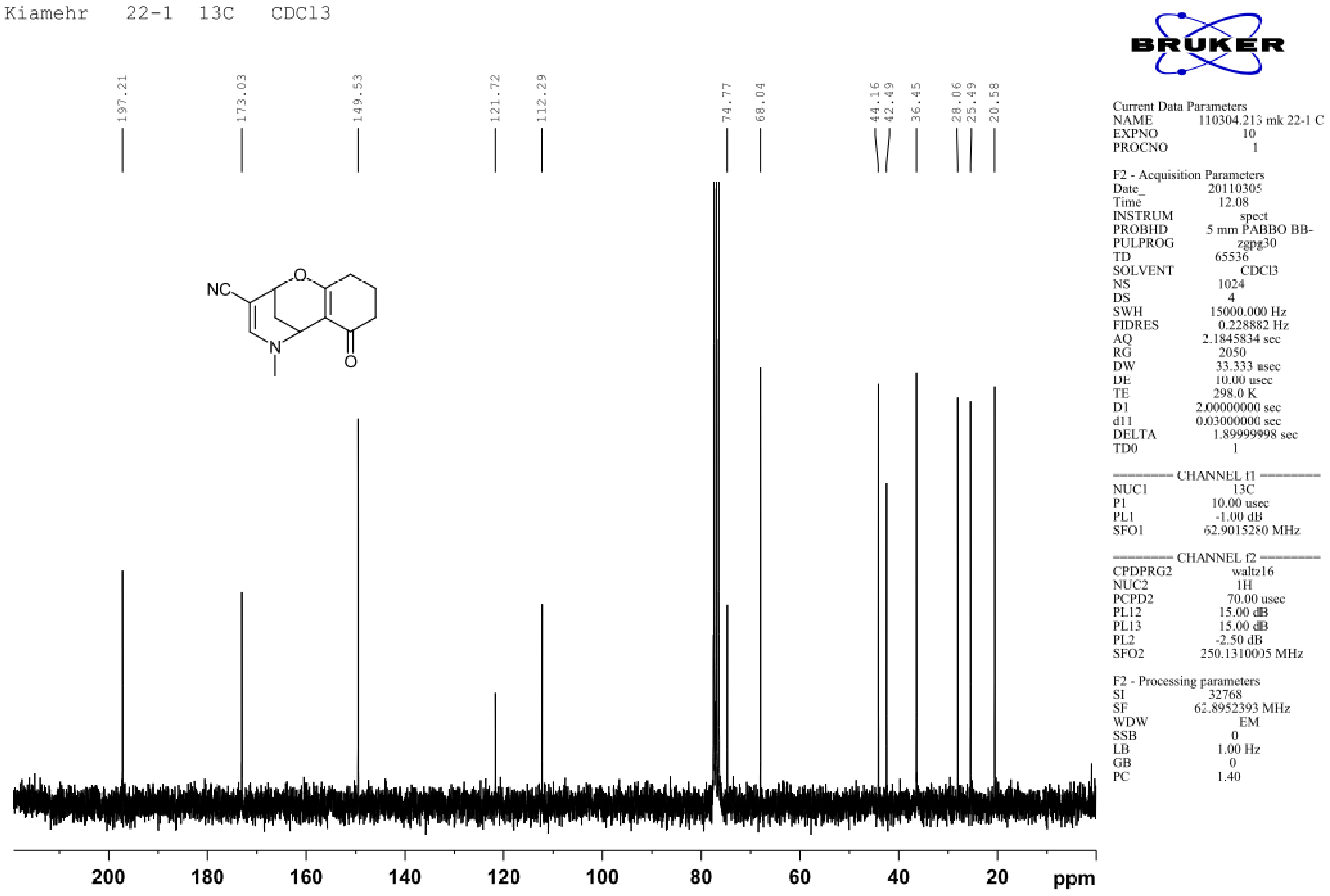

^{13}C NMR spectra for compound 8

X-ray structures of compounds 6d, 7c, 8

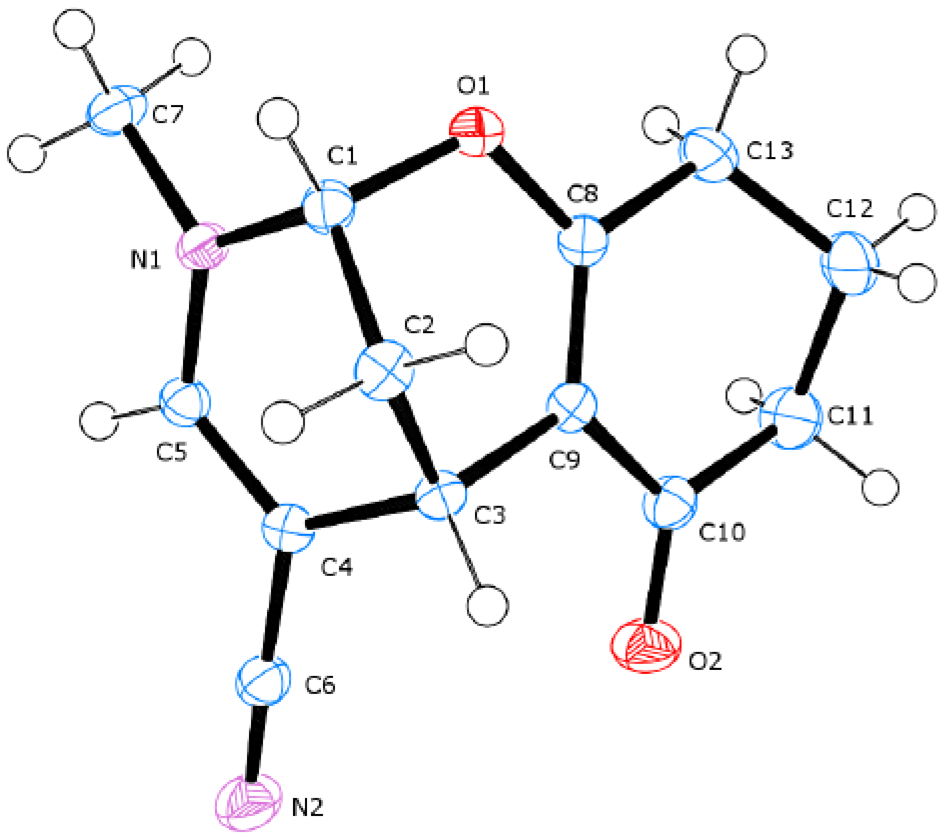

Figure S2: ORTEP plot of structure **6d.**

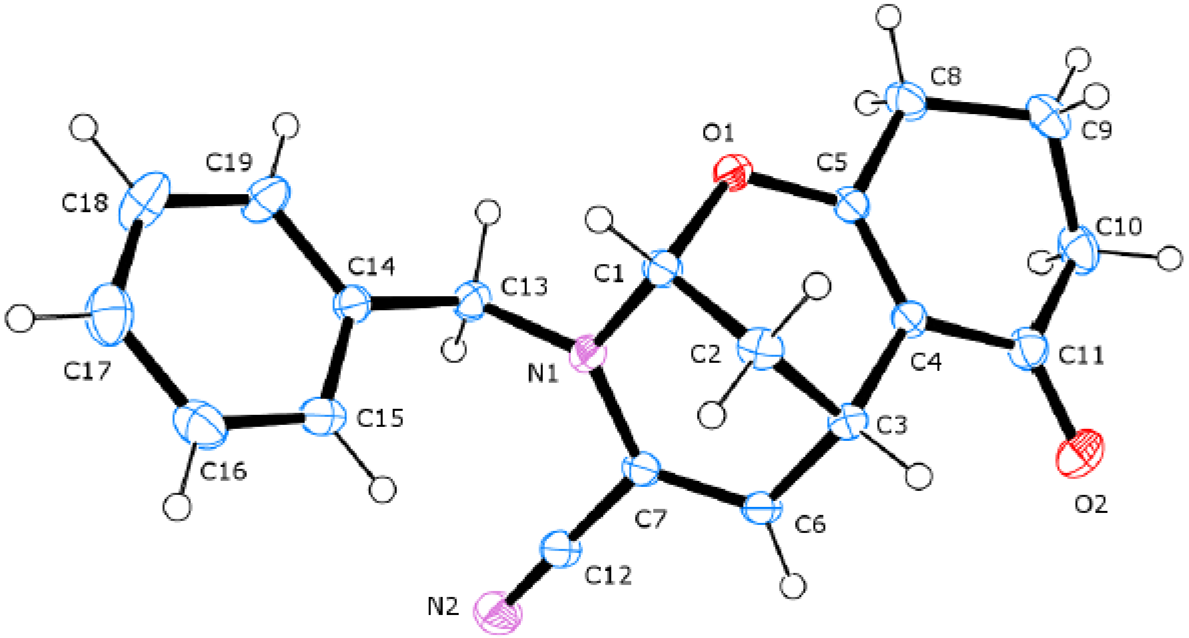

Figure S3: ORTEP plot of structure **7c**.

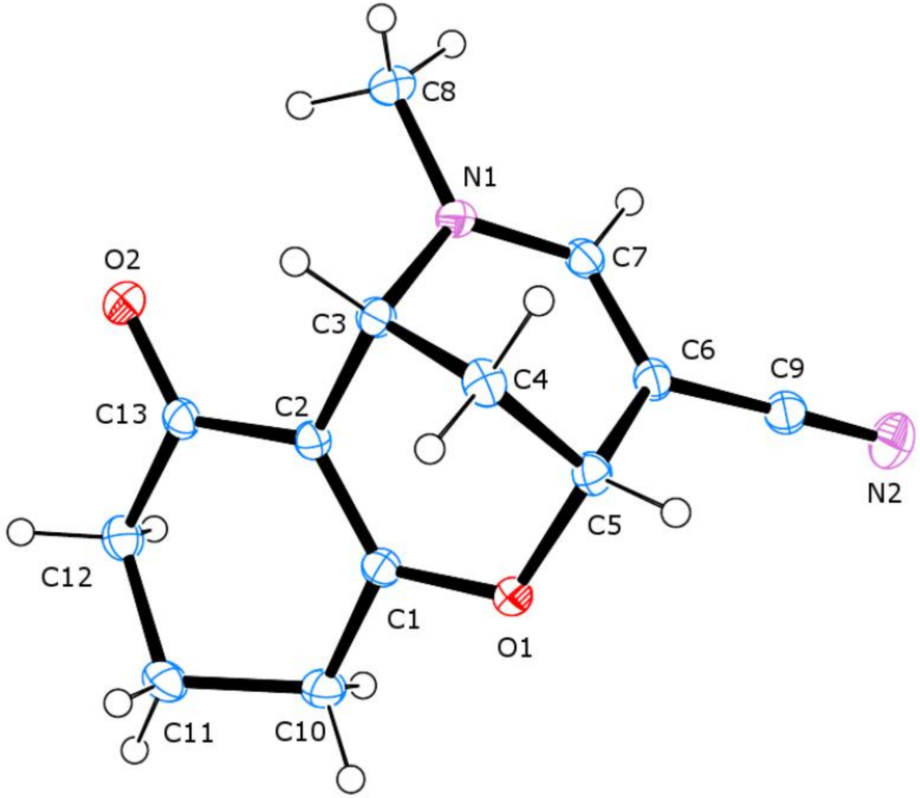

Figure S4: ORTEP plot of structure **8.**
