# Supplementary material for: Tandem dinucleophilic cyclization of cyclohexane-1,3-diones with pyridinium salts
Source: Beilstein J Org Chem. 2013 Jun 10;9:1119–26. doi: 10.3762/bjoc.9.124 (PMC3701411; doi:10.3762/bjoc.9.124)
Supplement: File 2 — Computational results, optimized structures (atomic coordinates as reported by Gaussian 09), charges and local softness indexes (nucleophilic attack) for cations 2a+, 2b+ and 2e+, additional discussions related to computations. [file Beilstein_J_Org_Chem-09-1119-s002.pdf]

Supporting Information File 2

for

Tandem dinucleophilic cyclization of cyclohexane-1,3-diones with pyridinium salts.

Mostafa Kiamehr[1,2,3], Firouz Matloubi Moghaddam[2]*, Satenik Mkrtchyan[1], Volodymyr Semeniuchenko[4], Linda Supe[1], Alexander Villinger[1], Peter Langer[1,5]*, Viktor O. Iaroshenko[1,4,§,]*

Address: [1]Institut für Chemie der Universität Rostock, Albert-Einstein-Straße 3a, D-18059 Rostock, Fax: (+49-381-498-6411), [2]Laboratory of Organic Synthesis and Natural Products, Department of Chemistry, Sharif University of Technology, P. O. Box 11155-9516 Tehran, Iran, [3]Department of Chemistry, Faculty of Science, University of Qom, Qom, Iran, [4]National Taras Shevchenko University, Volodymyrska st 62, Kyiv-33, 01033, Ukraine and [5]Leibniz Institut für Katalyse e.V. an der Universität Rostock, Albert-Einstein-Straße 29a, D-18059 Rostock.

Email: Firouz Matloubi Moghaddam* - Matloubi@sharif.edu, Peter Langer* – peter.langer@uni-rostock.de, Viktor O. Iaroshenko* - viktor.iaroshenko@uni-rostock.de

[§]further email address iva108@gmail.com

Computational results, optimized structures (atomic coordinates as reported by Gaussian 09), charges and local softness indexes (nucleophilic attack) for cations **2a**$^+$, **2b**$^+$ and **2e**$^+$, additional discussions related to computations.

Table of Contents

Local softness computation S3

Organic, inorganic compounds, complexes and ions...S13

Comparison of conformational energies and Boltzmann weighted average Gibbs free energies S95

State of ionizable compounds...S101

Formation of 6a and 6aa, thermodynamic analysis..S103

Reaction of 3-oxocyclohex-1-enolate with 3-acetyl-1-methyl pyridinium,
Impossibility of initial C-O bond formation...S105

Reaction intermediates and transition states..S142

Hypothetical reaction leading to 10-Acetyl-12-methyl-8-oxa-12-aza-tricyclo[7.3.1.0^{2,7}]trideca-2(7),10-dien-3-one (6aa)...S172

Explicit inclusion of acetonitrile S181

Local softness computation

2a⁺. Local softness was computed only for this conformer.

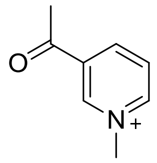

Center Number	Atomic Number	Atomic Type	X	Y	Z
1	6	0	-2.037324	0.964616	-0.000023
2	6	0	-0.469850	-0.787737	0.000001
3	6	0	0.591082	0.112767	0.000010
4	6	0	0.304647	1.480475	0.000006
5	6	0	-1.025929	1.904229	-0.000012
6	1	0	-3.087527	1.226940	-0.000040
7	1	0	-0.293408	-1.855346	0.000004
8	1	0	1.100986	2.215521	0.000013
9	1	0	-1.281972	2.956062	-0.000020
10	7	0	-1.745625	-0.361657	-0.000014
11	6	0	-2.863136	-1.337780	0.000021
12	1	0	-3.465797	-1.182045	0.894628
13	1	0	-3.466163	-1.181687	-0.894273
14	1	0	-2.452375	-2.344320	-0.000269
15	6	0	1.987108	-0.471079	0.000016
16	8	0	2.116684	-1.684182	-0.000033
17	6	0	3.159953	0.473373	0.000023
18	1	0	3.134987	1.121215	-0.882807
19	1	0	3.134970	1.121236	0.882835
20	1	0	4.082903	-0.105702	0.000036

(Coordinates in Angstroms)

E(UB3LYP) = -440.415125422 Ha (for dication)
E(RB3LYP) = -440.709714651 Ha (for cation)
ΔG (298.15 K, 1 atm) = -440.584330 Ha (for cation)
E(UB3LYP) = -440.818616500 Ha (for neutral)

Summary of Natural Population Analysis for dication:

Atom	No	Natural Charge	Core	Valence	Rydberg	Total
C	1	0.14055	1.99917	3.84184	0.01845	5.85945
C	2	0.16583	1.99910	3.81638	0.01870	5.83417
C	3	-0.14863	1.99883	4.13593	0.01387	6.14863
C	4	-0.07089	1.99905	4.05886	0.01298	6.07089
C	5	-0.21870	1.99912	4.20533	0.01425	6.21870
H	6	0.30291	0.00000	0.69592	0.00117	0.69709
H	7	0.30078	0.00000	0.69772	0.00150	0.69922
H	8	0.30572	0.00000	0.69298	0.00130	0.69428
H	9	0.31549	0.00000	0.68351	0.00100	0.68451
N	10	-0.27999	1.99930	5.26961	0.01108	7.27999
C	11	-0.48297	1.99944	4.47143	0.01211	6.48297
H	12	0.28520	0.00000	0.71381	0.00098	0.71480
H	13	0.28520	0.00000	0.71382	0.00098	0.71480
H	14	0.27625	0.00000	0.72283	0.00091	0.72375
C	15	0.62736	1.99926	3.34265	0.03073	5.37264
O	16	-0.04279	1.99980	6.02526	0.01773	8.04279
C	17	-0.75090	1.99929	4.74348	0.00813	6.75090
H	18	0.33600	0.00000	0.66312	0.00088	0.66400
H	19	0.33600	0.00000	0.66312	0.00088	0.66400
H	20	0.31760	0.00000	0.68129	0.00111	0.68240
* Total *		2.00000	19.99236	50.83888	0.16876	71.00000

(Core, Valence, Rydberg, Total: Natural Population)

Summary of Natural Population Analysis for cation:

Atom	No	Natural Charge	Core	Valence	Rydberg	Total
C	1	0.08861	1.99917	3.89401	0.01821	5.91139
C	2	0.12980	1.99912	3.85112	0.01997	5.87020
C	3	-0.15331	1.99895	4.13886	0.01550	6.15331
C	4	-0.11298	1.99907	4.10152	0.01239	6.11298
C	5	-0.23404	1.99913	4.22097	0.01394	6.23404
H	6	0.28539	0.00000	0.71342	0.00119	0.71461
H	7	0.28818	0.00000	0.70927	0.00255	0.71182
H	8	0.28509	0.00000	0.71367	0.00124	0.71491
H	9	0.29455	0.00000	0.70439	0.00106	0.70545
N	10	-0.28758	1.99930	5.27766	0.01062	7.28758
C	11	-0.48123	1.99945	4.47017	0.01161	6.48123

H	12	0.27473	0.00000	0.72423	0.00104	0.72527
H	13	0.27473	0.00000	0.72423	0.00104	0.72527
H	14	0.26965	0.00000	0.72945	0.00090	0.73035
C	15	0.59435	1.99932	3.37152	0.03480	5.40565
O	16	-0.54923	1.99980	6.53172	0.01771	8.54923
C	17	-0.79042	1.99936	4.78423	0.00683	6.79042
H	18	0.27456	0.00000	0.72431	0.00113	0.72544
H	19	0.27456	0.00000	0.72432	0.00113	0.72544
H	20	0.27458	0.00000	0.72369	0.00173	0.72542
* Total *		1.00000	19.99265	51.83276	0.17459	72.00000

Summary of Natural Population Analysis for neutral:

Atom	No	Natural Charge	Natural Population Core	Valence	Rydberg	Total
C	1	-0.11372	1.99918	4.09252	0.02202	6.11372
C	2	0.10434	1.99912	3.87678	0.01975	5.89566
C	3	-0.23229	1.99895	4.21597	0.01736	6.23229
C	4	-0.30534	1.99908	4.29115	0.01511	6.30534
C	5	-0.23503	1.99913	4.22188	0.01402	6.23503
H	6	0.24133	0.00000	0.75733	0.00134	0.75867
H	7	0.25683	0.00000	0.74020	0.00297	0.74317
H	8	0.23935	0.00000	0.75923	0.00142	0.76065
H	9	0.25513	0.00000	0.74365	0.00122	0.74487
N	10	-0.34912	1.99929	5.33834	0.01149	7.34912
C	11	-0.47178	1.99947	4.46168	0.01063	6.47178
H	12	0.24695	0.00000	0.75109	0.00196	0.75305
H	13	0.24696	0.00000	0.75109	0.00196	0.75304
H	14	0.25138	0.00000	0.74765	0.00097	0.74862
C	15	0.52551	1.99933	3.43849	0.03667	5.47449
O	16	-0.64589	1.99979	6.62868	0.01741	8.64589
C	17	-0.77667	1.99936	4.77097	0.00633	6.77667
H	18	0.25443	0.00000	0.74406	0.00151	0.74557
H	19	0.25443	0.00000	0.74406	0.00151	0.74557
H	20	0.25319	0.00000	0.74453	0.00228	0.74681
* Total *		0.00000	19.99271	52.81937	0.18792	73.00000

Hirshfeld spin densities, charges and dipoles using IRadAn= 4 for dication:

		1	2	3	4	5
1	C	-0.006363	0.140999	0.296298	-0.158425	0.000005
2	C	0.002900	0.130939	-0.105600	0.315336	-0.000003
3	C	0.087947	0.044171	-0.191731	0.033762	-0.000011
4	C	0.003590	0.078903	-0.232327	-0.231998	-0.000007
5	C	0.009317	0.039542	0.061226	-0.324080	-0.000001
6	H	-0.000271	0.119011	0.993840	-0.314329	0.000007
7	H	0.003603	0.108505	-0.147733	1.032731	-0.000013
8	H	0.001158	0.107829	-0.710943	-0.772583	-0.000006
9	H	0.001647	0.111062	0.249247	-1.057522	0.000008
10	N	0.002972	0.096630	0.123693	0.123161	0.000043
11	C	0.001329	0.023961	0.122640	0.115127	-0.000007
12	H	0.000315	0.086444	0.591656	-0.108325	-0.884643
13	H	0.000315	0.086444	0.592019	-0.108628	0.884346
14	H	0.000057	0.085032	-0.306370	0.995404	0.000294
15	C	0.057402	0.297284	-0.020843	-0.197711	-0.000044
16	O	0.716319	0.116871	-0.260897	1.564713	0.000062
17	C	0.098766	-0.000890	-0.105636	-0.054056	-0.000006
18	H	0.007805	0.114652	-0.036930	-0.583320	0.872531
19	H	0.007806	0.114650	-0.036915	-0.583340	-0.872520
20	H	0.003386	0.097898	-0.937946	0.545652	-0.000015
Tot		0.999999	1.999937	-0.063252	0.231566	0.000020
Dip from Atomic Chgs				0.277178	0.341366	0.000011
Total Dipole				0.213926	0.572932	0.000030

Hirshfeld spin densities, charges and dipoles using IRadAn= 4 for cation:

		1	2	3	4	5
1	C	0.000000	0.099356	0.303548	-0.161354	0.000005
2	C	0.000000	0.098739	-0.098312	0.312493	-0.000004
3	C	0.000000	0.012306	-0.201472	0.030742	-0.000010
4	C	0.000000	0.041447	-0.232511	-0.232771	-0.000007
5	C	0.000000	0.009509	0.061981	-0.330102	-0.000001
6	H	0.000000	0.101366	1.020096	-0.325059	0.000007
7	H	0.000000	0.087088	-0.134318	1.079359	-0.000014
8	H	0.000000	0.087520	-0.724864	-0.802650	-0.000007
9	H	0.000000	0.092567	0.258847	-1.086148	0.000008
10	N	0.000000	0.079260	0.125033	0.124777	0.000045

```
     11  C    0.000000   0.014028   0.123347   0.115931  -0.000006
     12  H    0.000000   0.077640   0.601780  -0.106787  -0.894805
     13  H    0.000000   0.077640   0.602146  -0.107093   0.894504
     14  H    0.000000   0.077994  -0.304958   1.007431   0.000297
     15  C    0.000000   0.189076  -0.029718  -0.183939  -0.000049
     16  O    0.000000  -0.251966  -0.329817   1.776245   0.000069
     17  C    0.000000  -0.079040  -0.103662  -0.050258  -0.000007
     18  H    0.000000   0.063904  -0.062414  -0.636744   0.928725
     19  H    0.000000   0.063902  -0.062398  -0.636765  -0.928713
     20  H    0.000000   0.057610  -1.011348   0.564867  -0.000017
        Tot   0.000000   0.999947  -0.199013   0.352175   0.000021
  Dip from Atomic Chgs             -2.416931   1.040352   0.000021
          Total Dipole             -2.615944   1.392526   0.000042

 Hirshfeld spin densities, charges and dipoles using IRadAn=      4 for neutral:
              1          2          3          4          5
      1  C    0.271934  -0.035975   0.328482  -0.176506   0.000004
      2  C   -0.000252   0.049995  -0.102180   0.321766  -0.000004
      3  C    0.098519  -0.064514  -0.212033   0.046440  -0.000011
      4  C    0.281335  -0.094163  -0.245182  -0.261681  -0.000007
      5  C   -0.010351  -0.054237   0.062805  -0.344734  -0.000002
      6  H    0.019763   0.049480   1.103156  -0.359562   0.000008
      7  H   -0.000455   0.059222  -0.139021   1.123266  -0.000015
      8  H    0.019932   0.036633  -0.773276  -0.876397  -0.000007
      9  H   -0.001594   0.057322   0.272548  -1.144247   0.000008
     10  N    0.115274   0.015388   0.124864   0.138387   0.000051
     11  C    0.007100  -0.009174   0.122927   0.119238  -0.000005
     12  H    0.006581   0.054872   0.628018  -0.103694  -0.923660
     13  H    0.006579   0.054875   0.628392  -0.104007   0.923347
     14  H    0.000271   0.061179  -0.303382   1.035728   0.000304
     15  C    0.070395   0.124910  -0.023449  -0.183077  -0.000054
     16  O    0.104762  -0.342799  -0.353405   1.842797   0.000072
     17  C    0.003771  -0.096265  -0.106142  -0.047104  -0.000007
     18  H    0.003295   0.046567  -0.073153  -0.654062   0.951822
     19  H    0.003295   0.046566  -0.073137  -0.654082  -0.951810
     20  H   -0.000141   0.040040  -1.041421   0.573995  -0.000017
        Tot   1.000016  -0.000078  -0.174590   0.292463   0.000017
  Dip from Atomic Chgs             -1.930952   0.347876   0.000020
          Total Dipole             -2.105541   0.640339   0.000036
```

For cation:

E_{HOMO} = -0.2855 Ha

E_{LUMO} = -0.10901Ha

Global hardness η = 0.088245

Global softness S = 0.0441225

Local softness (electrophilic attack):

Atom	Center number	NPA charges	Hirshfeld charges
C	1	-0.0089273	-0.005971142
C	2	-0.0011234	-0.002150707
C	3	-0.0034848	-0.00338949
C	4	-0.0084874	-0.005983452
C	5	-4.368E-05	-0.002812633
H	6	-0.001944	-0.00228934
H	7	-0.0013832	-0.001229518
H	8	-0.0020182	-0.002245262
H	9	-0.0017393	-0.001555098
N	10	-0.0027153	-0.002818192
C	11	0.00041696	-0.00102373
H	12	-0.0012257	-0.001004581
H	13	-0.0012253	-0.001004449
H	14	-0.0008061	-0.00074192
C	15	-0.0030374	-0.002831164
O	16	-0.0042649	-0.004007779
C	17	0.00060668	-0.00076001

H	18	-0.0008882	-0.000764952
H	19	-0.0008882	-0.000764908
H	20	-0.0009438	-0.000775232

⇨ 4-C (center number 4) and 6-C (center number 1) are soft; regioselectivity should not be expected (contradicts to the experiment).

2b$^+$

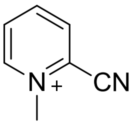

Center Number	Atomic Number	Atomic Type	Coordinates (Angstroms) X	Y	Z
1	6	0	-1.349390	1.105219	-0.000149
2	6	0	0.419928	-0.445857	0.000039
3	6	0	-1.847627	-1.242342	-0.000021
4	6	0	-2.283676	0.077525	-0.000128
5	1	0	-1.634954	2.148472	-0.000236
6	1	0	-2.557384	-2.061115	-0.000003
7	1	0	-3.336576	0.329076	-0.000199
8	7	0	-0.026812	0.847567	-0.000066
9	6	0	0.947181	1.968295	-0.000028
10	1	0	1.566061	1.902703	-0.894925
11	1	0	0.394574	2.903766	-0.000383
12	1	0	1.565548	1.903065	0.895254
13	6	0	-0.476266	-1.506155	0.000062
14	1	0	-0.091876	-2.517828	0.000142
15	6	0	1.833444	-0.674064	0.000126
16	7	0	2.974389	-0.890977	0.000201

E(UB3LYP) = -379.959711079 Ha (for dication)
E(RB3LYP) = -380.286907058 Ha (for cation)
ΔG (298.15 K, 1 atm) = -380.195353 Ha (for cation)
E(UB3LYP) = -380.413200906 Ha (for neutral)

Summary of Natural Population Analysis for dication:

Atom	No	Natural Charge	Natural Population Core	Valence	Rydberg	Total
C	1	0.25618	1.99921	3.72530	0.01931	5.74382
C	2	0.28388	1.99888	3.69929	0.01796	5.71612
C	3	-0.14009	1.99917	4.12561	0.01530	6.14009
C	4	-0.04439	1.99914	4.03137	0.01387	6.04439
H	5	0.32268	0.00000	0.67618	0.00114	0.67732
H	6	0.32973	0.00000	0.66925	0.00101	0.67027
H	7	0.33108	0.00000	0.66786	0.00106	0.66892
N	8	-0.27923	1.99928	5.26774	0.01221	7.27923
C	9	-0.49613	1.99942	4.48428	0.01243	6.49613
H	10	0.29862	0.00000	0.70044	0.00094	0.70138
H	11	0.29399	0.00000	0.70510	0.00091	0.70601
H	12	0.29863	0.00000	0.70044	0.00094	0.70137
C	13	-0.03464	1.99907	4.02181	0.01376	6.03464
H	14	0.33764	0.00000	0.66130	0.00105	0.66236
C	15	0.28632	1.99937	3.68519	0.02912	5.71368
N	16	-0.04427	1.99965	5.02435	0.02027	7.04427
* Total *		2.00000	17.99319	42.84552	0.16129	61.00000

Summary of Natural Population Analysis for cation:

Atom	No	Natural Charge	Natural Population Core	Valence	Rydberg	Total
C	1	0.10323	1.99918	3.87905	0.01854	5.89677
C	2	0.13651	1.99886	3.84695	0.01767	5.86349
C	3	-0.13227	1.99911	4.11917	0.01399	6.13227
C	4	-0.21833	1.99912	4.20577	0.01344	6.21833
H	5	0.28964	0.00000	0.70922	0.00113	0.71036
H	6	0.29440	0.00000	0.70450	0.00110	0.70560
H	7	0.29861	0.00000	0.70035	0.00104	0.70139
N	8	-0.26584	1.99927	5.25527	0.01130	7.26584
C	9	-0.48824	1.99944	4.47754	0.01126	6.48824
H	10	0.28038	0.00000	0.71859	0.00103	0.71962
H	11	0.27688	0.00000	0.72220	0.00091	0.72312
H	12	0.28038	0.00000	0.71859	0.00103	0.71962
C	13	-0.17577	1.99906	4.16381	0.01290	6.17577
H	14	0.30358	0.00000	0.69538	0.00104	0.69642
C	15	0.26507	1.99936	3.70454	0.03103	5.73493
N	16	-0.24822	1.99965	5.22803	0.02054	7.24822
* Total *		1.00000	17.99306	43.84898	0.15796	62.00000

Summary of Natural Population Analysis for neutral:

Atom	No	Natural Charge	Natural Population Core	Valence	Rydberg	Total
C	1	0.02467	1.99919	3.95715	0.01899	5.97533
C	2	-0.01860	1.99886	3.99958	0.02016	6.01860
C	3	-0.28732	1.99911	4.27209	0.01612	6.28732
C	4	-0.30694	1.99912	4.29299	0.01483	6.30694
H	5	0.25274	0.00000	0.74600	0.00126	0.74726
H	6	0.25172	0.00000	0.74700	0.00128	0.74828
H	7	0.25911	0.00000	0.73971	0.00118	0.74089
N	8	-0.35511	1.99926	5.34334	0.01251	7.35511
C	9	-0.47741	1.99947	4.46787	0.01008	6.47741
H	10	0.24894	0.00000	0.74931	0.00175	0.75106
H	11	0.25447	0.00000	0.74455	0.00098	0.74553
H	12	0.24893	0.00000	0.74931	0.00175	0.75107
C	13	-0.19324	1.99906	4.18122	0.01296	6.19324
H	14	0.26646	0.00000	0.73237	0.00116	0.73354
C	15	0.24394	1.99939	3.72126	0.03541	5.75606
N	16	-0.41236	1.99964	5.39006	0.02266	7.41236
* Total *		0.00000	17.99310	44.83381	0.17309	63.00000

Hirshfeld spin densities, charges and dipoles using IRadAn= 4 for dication:

		1	2	3	4	5
1	C	0.212785	0.229453	0.164443	-0.273575	0.000024
2	C	0.202447	0.224927	-0.112512	0.073252	-0.000008
3	C	-0.028251	0.103064	0.217563	0.251166	-0.000007
4	C	0.208546	0.148930	0.310238	-0.058068	0.000016
5	H	0.009062	0.145519	0.324260	-0.929452	0.000074
6	H	-0.002537	0.129885	0.692005	0.795773	-0.000018
7	H	0.009231	0.141859	1.009255	-0.233392	0.000067
8	N	-0.016990	0.110934	-0.114895	-0.143236	0.000059
9	C	-0.001731	0.029340	-0.110819	-0.127842	-0.000005
10	H	-0.002107	0.093132	-0.596922	-0.010094	0.897472
11	H	-0.000187	0.098144	0.436868	-0.921217	0.000345
12	H	-0.002108	0.093140	-0.596417	-0.010356	-0.897810
13	C	0.155465	0.136680	-0.076012	0.316087	-0.000025
14	H	0.006046	0.140131	-0.349599	1.004423	-0.000078
15	C	0.000810	0.187447	0.150669	0.016422	-0.000007
16	N	0.249509	-0.012640	-1.818240	0.377998	-0.000128
	Tot	0.999990	1.999946	-0.470117	0.127888	-0.000028
Dip from Atomic Chgs				-2.278619	0.464636	-0.000130
Total Dipole				-2.748736	0.592524	-0.000158

Hirshfeld spin densities, charges and dipoles using IRadAn= 4 for cation:

		1	2	3	4	5
1	C	0.000000	0.110196	0.174250	-0.286992	0.000025
2	C	0.000000	0.120402	-0.114441	0.075837	-0.000008
3	C	0.000000	0.051028	0.225591	0.258671	-0.000007
4	C	0.000000	0.023646	0.326704	-0.066604	0.000017
5	H	0.000000	0.104746	0.349717	-0.987461	0.000079
6	H	0.000000	0.098395	0.725584	0.833951	-0.000018
7	H	0.000000	0.097907	1.079947	-0.248691	0.000072
8	N	0.000000	0.083256	-0.117269	-0.146219	0.000062
9	C	0.000000	0.013068	-0.111051	-0.131328	-0.000004
10	H	0.000000	0.077542	-0.613705	-0.019565	0.917442
11	H	0.000000	0.082483	0.439129	-0.946629	0.000353
12	H	0.000000	0.077547	-0.613190	-0.019829	-0.917786
13	C	0.000000	0.027985	-0.083444	0.329335	-0.000027
14	H	0.000000	0.099276	-0.367926	1.070829	-0.000083
15	C	0.000000	0.106839	0.142286	0.023114	-0.000009
16	N	0.000000	-0.174361	-1.924586	0.402552	-0.000136
	Tot	0.000000	0.999953	-0.482404	0.140971	-0.000029
Dip from Atomic Chgs				-1.994862	0.913012	-0.000139
Total Dipole				-2.477265	1.053983	-0.000168

Hirshfeld spin densities, charges and dipoles using IRadAn= 4 for neutral:

		1	2	3	4	5
1	C	0.078077	0.028134	0.189834	-0.299491	0.000026
2	C	0.200775	0.019549	-0.130399	0.086684	-0.000010
3	C	0.192379	-0.068636	0.236257	0.285949	-0.000008
4	C	0.086678	-0.069577	0.344169	-0.081847	0.000019
5	H	0.004782	0.067802	0.378585	-1.041252	0.000084
6	H	0.013126	0.049926	0.779593	0.900377	-0.000020
7	H	0.005749	0.056322	1.149645	-0.268021	0.000077
8	N	0.211341	0.002920	-0.117651	-0.157621	0.000073
9	C	0.008884	-0.012489	-0.110425	-0.135439	-0.000002

10	H	0.008978	0.051923	-0.641155	-0.031675	0.951666
11	H	0.000217	0.062429	0.442191	-0.979835	0.000362
12	H	0.008983	0.051923	-0.640629	-0.031946	-0.952028
13	C	-0.009321	-0.033753	-0.081082	0.345480	-0.000028
14	H	-0.001104	0.065556	-0.382385	1.128417	-0.000087
15	C	0.035860	0.035334	0.142420	0.033698	-0.000010
16	N	0.154596	-0.307414	-2.029661	0.427048	-0.000144
	Tot	0.999999	-0.000051	-0.470693	0.180525	-0.000031
Dip from Atomic Chgs				-1.577375	1.240027	-0.000147
Total Dipole				-2.048067	1.420552	-0.000178

For cation:
E_{HOMO} = -0.32472 Ha
E_{LUMO} = -0.1275 Ha
Global hardness η = 0.09861
Global softness S = 0.049305
Local softness (electrophilic attack):

Atom	Center number	NPA charges	Hirshfeld charges
C	1	-0.0038734	-0.004046067
C	2	-0.0076477	-0.004972557
C	3	-0.0076447	-0.005900034
C	4	-0.0043689	-0.00459636
H	5	-0.0018194	-0.001821524
H	6	-0.0021043	-0.002389764
H	7	-0.0019475	-0.002050348
N	8	-0.0044015	-0.003960966
C	9	0.00053397	-0.001260088
H	10	-0.0015501	-0.001263145
H	11	-0.0011049	-0.000988762
H	12	-0.0015506	-0.001263391
C	13	-0.0008614	-0.003043992
H	14	-0.0018302	-0.001662565
C	15	-0.0010418	-0.003525554
N	16	-0.0080929	-0.006560178

⇨ 2-C (center number 2) and 4C (center number 3) are soft, 1-C (center number 1) is hard, but 2-C is shielded by a nitrile group; hence regioselectivity is expected (in line with experiment)

2e⁺

Center Number	Atomic Number	Atomic Type	Coordinates (Angstroms) X	Y	Z
1	6	0	-1.630974	0.902360	-0.005860
2	6	0	-0.067561	-0.857157	-0.006574
3	6	0	0.985847	0.055226	0.000777
4	6	0	0.707692	1.430370	0.004897
5	6	0	-0.620356	1.846645	0.002305
6	1	0	-2.680943	1.164425	-0.009548
7	1	0	0.088658	-1.927400	-0.008946
8	1	0	1.516321	2.151443	0.008603
9	1	0	-0.878373	2.897767	0.003792
10	7	0	-1.341711	-0.421671	-0.010468
11	6	0	-2.458759	-1.401049	0.009390
12	1	0	-2.888619	-1.419662	1.011273
13	1	0	-3.206691	-1.090815	-0.718459
14	1	0	-2.072308	-2.383279	-0.251661
15	6	0	2.334615	-0.430836	0.000748
16	7	0	3.430129	-0.816305	0.000590

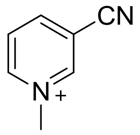

E(UB3LYP) = -379.967059858 Ha (for dication)
E(RB3LYP) = -380.293153931 Ha (for cation)
ΔG (298.15 K, 1 atm) = -380.202633 Ha (for cation)
E(UB3LYP) = -380.409293144 Ha (for neutral)

Summary of Natural Population Analysis for dication:

Atom	No	Natural Charge	Natural Population Core	Valence	Rydberg	Total
C	1	0.26971	1.99918	3.71200	0.01910	5.73029
C	2	0.28274	1.99914	3.69953	0.01860	5.71726
C	3	-0.02828	1.99886	4.01647	0.01296	6.02828
C	4	-0.07351	1.99910	4.05997	0.01443	6.07351
C	5	-0.14287	1.99914	4.12925	0.01448	6.14287
H	6	0.32212	0.00000	0.67666	0.00122	0.67788
H	7	0.32875	0.00000	0.67017	0.00108	0.67125
H	8	0.33244	0.00000	0.66659	0.00098	0.66756
H	9	0.33404	0.00000	0.66495	0.00101	0.66596
N	10	-0.29829	1.99933	5.28740	0.01156	7.29829
C	11	-0.48565	1.99943	4.47339	0.01283	6.48565
H	12	0.29955	0.00000	0.69949	0.00095	0.70045
H	13	0.29403	0.00000	0.70503	0.00094	0.70597
H	14	0.28692	0.00000	0.71216	0.00092	0.71308
C	15	0.34543	1.99939	3.62501	0.03017	5.65457
N	16	-0.06713	1.99966	5.04707	0.02040	7.06713
* Total *		2.00000	17.99323	42.84513	0.16165	61.00000

Summary of Natural Population Analysis for cation:

Atom	No	Natural Charge	Natural Population Core	Valence	Rydberg	Total
C	1	0.10077	1.99917	3.88165	0.01841	5.89923
C	2	0.13402	1.99912	3.84910	0.01775	5.86598
C	3	-0.17455	1.99886	4.16286	0.01284	6.17455
C	4	-0.09038	1.99907	4.07809	0.01321	6.09038
C	5	-0.23060	1.99912	4.21777	0.01372	6.23060
H	6	0.29029	0.00000	0.70853	0.00118	0.70971
H	7	0.29542	0.00000	0.70349	0.00109	0.70458
H	8	0.29864	0.00000	0.70031	0.00105	0.70136
H	9	0.30126	0.00000	0.69771	0.00103	0.69874
N	10	-0.28516	1.99930	5.27513	0.01073	7.28516
C	11	-0.48090	1.99945	4.46974	0.01172	6.48090
H	12	0.27929	0.00000	0.71967	0.00104	0.72071
H	13	0.27647	0.00000	0.72254	0.00099	0.72353
H	14	0.27199	0.00000	0.72709	0.00092	0.72801
C	15	0.30532	1.99937	3.66338	0.03193	5.69468
N	16	-0.29188	1.99965	5.27172	0.02050	7.29188
* Total *		1.00000	17.99312	43.84877	0.15812	62.00000

Summary of Natural Population Analysis for neutral:

Atom	No	Natural Charge	Natural Population			
			Core	Valence	Rydberg	Total
C	1	-0.10500	1.99919	4.08370	0.02211	6.10500
C	2	0.08815	1.99913	3.89490	0.01782	5.91185
C	3	-0.25447	1.99885	4.24105	0.01458	6.25447
C	4	-0.31006	1.99907	4.29431	0.01667	6.31006
C	5	-0.23187	1.99912	4.21891	0.01384	6.23187
H	6	0.24404	0.00000	0.75464	0.00132	0.75596
H	7	0.26010	0.00000	0.73866	0.00124	0.73990
H	8	0.24747	0.00000	0.75126	0.00127	0.75253
H	9	0.25960	0.00000	0.73923	0.00117	0.74040
N	10	-0.36753	1.99929	5.35617	0.01208	7.36753
C	11	-0.47130	1.99948	4.46123	0.01060	6.47130
H	12	0.24497	0.00000	0.75298	0.00205	0.75503
H	13	0.24824	0.00000	0.75000	0.00177	0.75176
H	14	0.25063	0.00000	0.74836	0.00101	0.74937
C	15	0.30830	1.99939	3.65757	0.03474	5.69170
N	16	-0.41126	1.99964	5.38966	0.02196	7.41126
* Total *		0.00000	17.99316	44.83260	0.17424	63.00000

Hirshfeld spin densities, charges and dipoles using IRadAn= 4 for dication:

		1	2	3	4	5
1	C	0.230615	0.231116	0.282936	-0.156888	0.000290
2	C	0.204820	0.226180	-0.115255	0.319905	0.001292
3	C	0.193380	0.149971	-0.125331	0.025513	-0.002815
4	C	-0.015921	0.110947	-0.244415	-0.239336	-0.003223
5	C	0.076083	0.104850	0.062781	-0.314008	-0.003587
6	H	0.009903	0.145954	0.951871	-0.300282	-0.003923
7	H	0.008123	0.145818	-0.190767	0.993377	-0.004404
8	H	-0.002046	0.131181	-0.789581	-0.728870	-0.003851
9	H	0.002826	0.134682	0.245134	-1.020488	-0.001724
10	N	-0.006153	0.115577	0.122799	0.118033	0.031227
11	C	0.001592	0.035623	0.122353	0.113699	-0.003186
12	H	-0.000846	0.097427	0.415888	0.039989	-0.972472
13	H	-0.000439	0.096234	0.725497	-0.238256	0.715525
14	H	-0.000036	0.094809	-0.285186	0.953692	0.260542
15	C	0.016247	0.204778	0.135059	-0.037738	-0.000242
16	N	0.281835	-0.025254	-1.757002	0.618599	0.000264
	Tot	0.999983	1.999891	-0.443220	0.146940	0.009711
Dip from Atomic Chgs				-2.204103	0.569156	0.003381
Total Dipole				-2.647323	0.716095	0.013093

Hirshfeld spin densities, charges and dipoles using IRadAn= 4 for cation:

		1	2	3	4	5
1	C	0.000000	0.110582	0.297484	-0.162971	0.000718
2	C	0.000000	0.112972	-0.120209	0.331762	0.001429
3	C	0.000000	0.045427	-0.126351	0.030259	-0.003315
4	C	0.000000	0.057394	-0.252152	-0.245082	-0.003354
5	C	0.000000	0.019493	0.070342	-0.326902	-0.003967
6	H	0.000000	0.106040	1.010178	-0.323610	-0.004413
7	H	0.000000	0.106455	-0.203150	1.054813	-0.005064
8	H	0.000000	0.100319	-0.824996	-0.767000	-0.004039
9	H	0.000000	0.098671	0.258518	-1.077923	-0.001811
10	N	0.000000	0.084619	0.127445	0.119418	0.032963
11	C	0.000000	0.017940	0.123504	0.116064	-0.002667
12	H	0.000000	0.080568	0.430350	0.046526	-0.994304
13	H	0.000000	0.080710	0.745887	-0.236799	0.730623
14	H	0.000000	0.080561	-0.283058	0.976666	0.265884
15	C	0.000000	0.104866	0.122282	-0.036588	-0.000333
16	N	0.000000	-0.206712	-1.870712	0.658286	0.000225
	Tot	0.000000	0.999905	-0.494637	0.156920	0.008575
Dip from Atomic Chgs				-3.031963	0.400484	0.001318
Total Dipole				-3.526600	0.557404	0.009893

Hirshfeld spin densities, charges and dipoles using IRadAn= 4 for cation:

		1	2	3	4	5
1	C	0.279279	-0.029606	0.322255	-0.181906	-0.000395
2	C	0.035850	0.051161	-0.129205	0.345142	0.001156
3	C	0.074083	-0.032700	-0.137028	0.049531	-0.003655
4	C	0.321930	-0.095725	-0.271897	-0.276175	-0.003316
5	C	-0.011959	-0.048929	0.075411	-0.341728	-0.004340
6	H	0.020120	0.051968	1.095779	-0.360917	-0.005045
7	H	0.002289	0.073641	-0.218490	1.107428	-0.005640
8	H	0.022009	0.041325	-0.899701	-0.843380	-0.004676
9	H	-0.001688	0.061410	0.273837	-1.139131	-0.001887

```
     10  N    0.169802   0.005656   0.128922   0.133205   0.039117
     11  C    0.008571  -0.008586   0.123738   0.119416  -0.001473
     12  H    0.009532   0.053053   0.455770   0.055543  -1.032315
     13  H    0.006556   0.056720   0.778160  -0.236298   0.755782
     14  H    0.000684   0.061289  -0.280827   1.008141   0.272925
     15  C    0.019341   0.059982   0.121985  -0.030579  -0.000236
     16  N    0.043612  -0.300776  -1.941670   0.682996   0.000209
        Tot   1.000013  -0.000116  -0.502961   0.091290   0.006210
 Dip from Atomic Chgs             -2.814101  -0.317743  -0.007810
          Total Dipole            -3.317062  -0.226453  -0.001600
```

For cation:
E_{HOMO} = -0.32351Ha
E_{LUMO} = -0.11635Ha
Global hardness η = 0.10358
Global softness S = 0.05179
Local softness (electrophilic attack):

Atom	Center number	NPA charges	Hirshfeld charges
C	1	-0.010656828	-0.007260337
C	2	-0.002375607	-0.003201192
C	3	-0.004139057	-0.004046197
C	4	-0.011377227	-0.007930033
C	5	-6.57733E-05	-0.003543575
H	6	-0.002395288	-0.002800389
H	7	-0.001829223	-0.001699437
H	8	-0.002650094	-0.003055299
H	9	-0.002157571	-0.001929747
N	10	-0.004265942	-0.004089494
C	11	0.000497184	-0.001373782
H	12	-0.001777433	-0.001425002
H	13	-0.001462032	-0.001242442
H	14	-0.001106234	-0.000998097
C	15	0.000154334	-0.002324542
N	16	-0.00618269	-0.004871575

⇨ 4-C (center number 4) and 6-C (center number 1) are soft; regioselectivity should not be expected (in line with experiment).

Organic, inorganic compounds, complexes and ions

2a^{+} - conformation 1

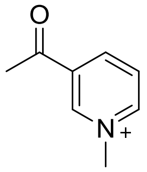

Center Number	Atomic Number	Atomic Type	Coordinates (Angstroms) X	Y	Z
1	6	0	2.095688	0.847081	0.000148
2	6	0	0.385738	-0.772360	-0.000162
3	6	0	-0.593098	0.213452	-0.000117
4	6	0	-0.190381	1.553872	0.000064
5	6	0	1.165295	1.870262	0.000192
6	1	0	3.164066	1.020925	0.000257
7	1	0	0.160605	-1.830042	-0.000385
8	1	0	-0.951429	2.325867	0.000106
9	1	0	1.508678	2.896948	0.000327
10	7	0	1.697049	-0.447572	-0.000030
11	6	0	2.731730	-1.511766	-0.000133
12	1	0	3.345463	-1.405451	-0.894351
13	1	0	3.345217	-1.405883	0.894305
14	1	0	2.242233	-2.482551	-0.000402
15	6	0	-2.072460	-0.109420	-0.000207
16	8	0	-2.866946	0.815144	-0.000325
17	6	0	-2.507565	-1.552228	0.000343
18	1	0	-2.123413	-2.075657	-0.881880
19	1	0	-2.128464	-2.072596	0.886618
20	1	0	-3.596414	-1.593071	-0.002547

E(RB3LYP/6-31G(d,p)) = -440.709019338 Ha

ΔG (298.15 K, 1 atm, B3LYP/6-31G(d,p)) = -440.584093 Ha

2a$^+$ - conformation 2

Center Number	Atomic Number	Atomic Type	Coordinates (Angstroms) X	Y	Z
1	6	0	-2.037324	0.964616	-0.000023
2	6	0	-0.469850	-0.787737	0.000001
3	6	0	0.591082	0.112767	0.000010
4	6	0	0.304647	1.480475	0.000006
5	6	0	-1.025929	1.904229	-0.000012
6	1	0	-3.087527	1.226940	-0.000040
7	1	0	-0.293408	-1.855346	0.000004
8	1	0	1.100986	2.215521	0.000013
9	1	0	-1.281972	2.956062	-0.000020
10	7	0	-1.745625	-0.361657	-0.000014
11	6	0	-2.863136	-1.337780	0.000021
12	1	0	-3.465797	-1.182045	0.894628
13	1	0	-3.466163	-1.181687	-0.894273
14	1	0	-2.452375	-2.344320	-0.000269
15	6	0	1.987108	-0.471079	0.000016
16	8	0	2.116684	-1.684182	-0.000033
17	6	0	3.159953	0.473373	0.000023
18	1	0	3.134987	1.121215	-0.882807
19	1	0	3.134970	1.121236	0.882835
20	1	0	4.082903	-0.105702	0.000036

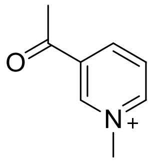

E(RB3LYP/6-31G(d,p)) = -440.709714651 Ha

ΔG (298.15 K, 1 atm, B3LYP/6-31G(d,p)) = -440.584330 Ha

2a-I – conformation 1

Center Number	Atomic Number	Atomic Type	X	Y	Z
1	6	0	2.634385	-2.180663	-0.153479
2	6	0	1.202810	-0.373040	0.341659
3	6	0	2.202760	0.541222	0.030289
4	6	0	3.448214	0.057084	-0.388417
5	6	0	3.664279	-1.314921	-0.478273
6	1	0	2.732902	-3.257416	-0.201099
7	1	0	0.196813	-0.091158	0.637513
8	1	0	4.225937	0.770035	-0.638239
9	1	0	4.615598	-1.721045	-0.798591
10	7	0	1.432294	-1.702833	0.244816
11	6	0	0.352653	-2.650738	0.616093
12	1	0	-0.609264	-2.180406	0.405530
13	1	0	0.465345	-3.558821	0.026641
14	1	0	0.436972	-2.880605	1.679550
15	6	0	1.990468	2.037319	0.116718
16	8	0	2.887558	2.773757	-0.260455
17	6	0	0.693244	2.557889	0.676350
18	1	0	-0.169757	2.153939	0.133897
19	1	0	0.584985	2.251889	1.723376
20	1	0	0.691779	3.646313	0.617060
21	53	0	-2.706240	0.048154	-0.146818

(Coordinates in Angstroms)

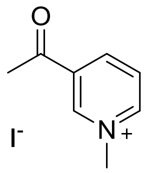

E(RB3LYP/6-31G(d,p)) = -452.278319951 Ha

ΔG (298.15 K, 1 atm, B3LYP/6-31G(d,p)) = -452.159073 Ha

2a-I – conformation 2

Center Number	Atomic Number	Atomic Type	Coordinates (Angstroms) X	Y	Z
1	6	0	2.578298	-2.318015	-0.052570
2	6	0	1.219609	-0.400508	0.095522
3	6	0	2.334242	0.432679	0.012091
4	6	0	3.598560	-0.150675	-0.109342
5	6	0	3.716449	-1.542047	-0.141987
6	1	0	2.597620	-3.400083	-0.069730
7	1	0	0.211247	-0.004383	0.180335
8	1	0	4.489164	0.462959	-0.179260
9	1	0	4.681138	-2.024555	-0.235962
10	7	0	1.355712	-1.739654	0.064211
11	6	0	0.160258	-2.612963	0.175696
12	1	0	-0.734450	-1.991163	0.130393
13	1	0	0.168220	-3.322898	-0.651308
14	1	0	0.204459	-3.143882	1.127482
15	6	0	2.091510	1.924746	0.059673
16	8	0	0.948542	2.335313	0.174296
17	6	0	3.278629	2.849515	-0.033219
18	1	0	3.971714	2.674225	0.796796
19	1	0	3.831699	2.683081	-0.963798
20	1	0	2.927386	3.880597	0.001037
21	53	0	-2.892299	0.161977	-0.038019

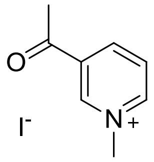

E(RB3LYP/6-31G(d,p)) = -452.277327229 Ha

ΔG (298.15 K, 1 atm, B3LYP/6-31G(d,p)) = -452.159213 Ha

2a-HCO_3 – conformation 1

Center Number	Atomic Number	Atomic Type	X	Y	Z
1	6	0	2.105415	-2.121067	-0.223370
2	6	0	0.558692	-0.405405	0.279038
3	6	0	1.530559	0.564277	0.042553
4	6	0	2.813267	0.159784	-0.339206
5	6	0	3.102845	-1.196686	-0.470136
6	1	0	2.256738	-3.189953	-0.306018
7	1	0	-0.483003	-0.199195	0.558404
8	1	0	3.564238	0.918538	-0.528709
9	1	0	4.086401	-1.542896	-0.761974
10	7	0	0.865320	-1.715738	0.141014
11	6	0	-0.177565	-2.740332	0.407619
12	1	0	-1.130430	-2.228332	0.547282
13	1	0	-0.224930	-3.418680	-0.444228
14	1	0	0.098431	-3.292478	1.307215
15	6	0	1.234906	2.042899	0.171978
16	8	0	2.107948	2.839616	-0.139272
17	6	0	-0.116286	2.465412	0.673450
18	1	0	-0.919133	2.059023	0.038346
19	1	0	-0.285277	2.069587	1.681648
20	1	0	-0.161107	3.554688	0.699426
21	6	0	-2.966733	0.095370	-0.152782
22	8	0	-2.374158	-0.460201	0.809123
23	8	0	-4.264423	-0.380099	-0.368971
24	1	0	-4.595501	0.135997	-1.118834
25	8	0	-2.566150	0.995478	-0.925196

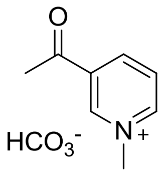

E(RB3LYP/6-31G(d,p)) = -705.267582236 Ha

ΔG (298.15 K, 1 atm, B3LYP/6-31G(d,p)) = -705.123579 Ha

2a-HCO_3 – conformation 2

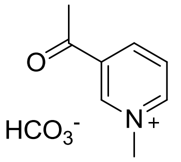

Center Number	Atomic Number	Atomic Type	X	Y	Z
1	6	0	1.363972	2.424828	0.167918
2	6	0	0.360651	0.368594	-0.409413
3	6	0	1.526622	-0.320129	-0.063796
4	6	0	2.620103	0.403183	0.416705
5	6	0	2.534550	1.793103	0.531929
6	1	0	1.226473	3.496843	0.227736
7	1	0	-0.542757	-0.119510	-0.775940
8	1	0	3.537974	-0.099207	0.698960
9	1	0	3.366007	2.382003	0.897528
10	7	0	0.307328	1.708883	-0.292694
11	6	0	-0.939764	2.424158	-0.660992
12	1	0	-1.669201	1.681834	-0.985153
13	1	0	-1.307546	2.953566	0.217972
14	1	0	-0.709863	3.133447	-1.456989
15	6	0	1.529255	-1.818588	-0.238896
16	8	0	0.550307	-2.369158	-0.717204
17	6	0	2.755259	-2.590752	0.184138
18	1	0	3.632645	-2.269664	-0.387657
19	1	0	2.975401	-2.424769	1.243911
20	1	0	2.581332	-3.652485	0.010823
21	6	0	-2.792957	-0.398277	0.221030
22	8	0	-2.563950	-0.323460	-1.013111
23	8	0	-4.000204	-1.035342	0.539786
24	1	0	-4.037958	-1.018321	1.507634
25	8	0	-2.104897	0.010129	1.185316

E(RB3LYP/6-31G(d,p)) = -705.263406102 Ha

ΔG (298.15 K, 1 atm, B3LYP/6-31G(d,p)) = -705.119678 Ha

K+

Center Number	Atomic Number	Atomic Type	Coordinates (Angstroms) X	Y	Z
1	19	0	0.000000	0.000000	0.000000

E(RB3LYP/6-31G(d,p)) = -599.847929488 Ha

ΔG (298.15 K, 1 atm, B3LYP/6-31G(d,p)) = -599.863105 Ha

I-

Center Number	Atomic Number	Atomic Type	Coordinates (Angstroms) X	Y	Z
1	53	0	0.000000	0.000000	0.000000

E(RB3LYP/6-31G(d,p)) = -11.5711925877 Ha

ΔG (298.15 K, 1 atm, B3LYP/LANL2DZ) = -11.5880406 Ha

KI

Center Number	Atomic Number	Atomic Type	Coordinates (Angstroms) X	Y	Z
1	19	0	0.000000	0.000000	-2.582345
2	53	0	0.000000	0.000000	0.925746

E(RB3LYP/6-31G(d,p)) = -611.427779696 Ha

ΔG (298.15 K, 1 atm, B3LYP/6-31G(d,p):LANL2DZ) = -611.453959 Ha

K_2CO_3

Center Number	Atomic Number	Atomic Type	Coordinates (Angstroms) X	Y	Z
1	6	0	0.000053	0.845607	0.000515
2	8	0	-1.127906	1.478415	-0.000619
3	8	0	1.127466	1.479118	0.000176
4	8	0	0.000381	-0.474227	0.002007
5	19	0	-2.595424	-0.656320	-0.000338
6	19	0	2.595433	-0.656316	-0.000483

E(RB3LYP/6-31G(d,p)) = -1463.77321889 Ha

ΔG (298.15 K, 1 atm, B3LYP/6-31G(d,p)) = -1463.791289 Ha

$KHCO_3$

Center Number	Atomic Number	Atomic Type	Coordinates (Angstroms) X	Y	Z
1	6	0	1.050596	0.036156	-0.000040
2	8	0	0.486305	1.155010	-0.000014
3	8	0	2.444371	0.080297	0.000030
4	1	0	2.724293	-0.847046	0.000084
5	8	0	0.528117	-1.110687	-0.000022
6	19	0	-1.931485	-0.019308	0.000011

E(RB3LYP/6-31G(d,p)) = -864.412400617 Ha

ΔG (298.15 K, 1 atm, B3LYP/6-31G(d,p)) = -864.416808 Ha

H_2CO_3

Center Number	Atomic Number	Atomic Type	Coordinates (Angstroms) X	Y	Z
1	6	0	-0.052597	-0.126805	-0.000041
2	8	0	-0.705021	-1.144307	0.000030
3	8	0	1.285508	-0.117110	-0.000059
4	1	0	1.609215	0.797936	0.000898
5	8	0	-0.552142	1.124783	-0.000169
6	1	0	-1.520388	1.055955	0.000940

E(RB3LYP/6-31G(d,p)) = -265.012200221 Ha

ΔG (298.15 K, 1 atm, B3LYP/6-31G(d,p)) = -265.000483 Ha

HCO_3^-

Center Number	Atomic Number	Atomic Type	Coordinates (Angstroms) X	Y	Z
1	6	0	0.143089	0.061970	-0.000080
2	8	0	1.204971	-0.591083	0.000025
3	8	0	-1.030859	-0.742511	0.000009
4	1	0	-1.752241	-0.096942	0.000052
5	8	0	-0.062398	1.299234	0.000020

E(RB3LYP/6-31G(d,p)) = -264.533941217 Ha

ΔG (298.15 K, 1 atm, B3LYP/6-31G(d,p)) = -264.534460 Ha

cyclohexane-1,3-dione

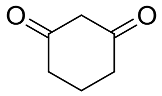

Center Number	Atomic Number	Atomic Type	Coordinates (Angstroms) X	Y	Z
1	6	0	1.277756	1.061477	0.224547
2	6	0	-0.000063	-1.160988	0.475362
3	6	0	-1.277626	1.061554	0.224510
4	6	0	0.000086	1.692373	-0.361425
5	1	0	-0.000083	-2.191892	0.117660
6	1	0	1.325132	1.274352	1.302179
7	1	0	2.180132	1.468243	-0.237570
8	1	0	-2.179986	1.468448	-0.237524
9	1	0	-1.324938	1.274278	1.302176
10	1	0	0.000090	1.573377	-1.451198
11	1	0	0.000112	2.767008	-0.160490
12	1	0	-0.000176	-1.177427	1.576240
13	6	0	-1.281453	-0.445361	0.052194
14	6	0	1.281522	-0.445490	0.052544
15	8	0	-2.233133	-1.064776	-0.388529
16	8	0	2.232930	-1.064947	-0.388704

E(RB3LYP/6-31G(d,p)) = -383.922421027 Ha

ΔG (298.15 K, 1 atm, B3LYP/6-31G(d,p)) = -383.828410 Ha

2,6-dioxocyclohexan-1-ide

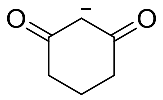

Center Number	Atomic Number	Atomic Type	Coordinates (Angstroms) X	Y	Z
1	6	0	0.000001	1.706352	-0.346796
2	6	0	-1.259068	1.031410	0.203398
3	6	0	-0.000001	-1.138963	-0.078737
4	6	0	1.259070	1.031409	0.203398
5	1	0	-1.350416	1.239483	1.280824
6	1	0	-2.168256	1.425974	-0.264017
7	1	0	0.000000	1.625861	-1.442230
8	1	0	0.000001	2.778046	-0.111639
9	1	0	-0.000001	-2.222153	-0.199143
10	1	0	1.350421	1.239488	1.280822
11	1	0	2.168257	1.425970	-0.264022
12	6	0	1.258328	-0.493634	0.014471
13	6	0	-1.258329	-0.493632	0.014462
14	8	0	-2.367291	-1.085644	-0.021359
15	8	0	2.367290	-1.085646	-0.021362

E(RB3LYP/6-31G(d,p)) = -383.436265008 Ha

ΔG (298.15 K, 1 atm, B3LYP/6-31G(d,p)) = -383.353093 Ha

potassium 3-oxocyclohex-1-enolate

Center Number	Atomic Number	Atomic Type	Coordinates (Angstroms) X	Y	Z
1	6	0	2.288491	-1.379189	-0.354800
2	6	0	2.788240	-0.043549	0.201379
3	6	0	0.403125	0.749028	-0.072903
4	6	0	0.897174	-1.708809	0.194052
5	1	0	2.989585	-0.142123	1.279097
6	1	0	3.731590	0.263544	-0.262753
7	1	0	2.235122	-1.315163	-1.449769
8	1	0	2.994674	-2.184719	-0.120996
9	1	0	-0.309730	1.566006	-0.186138
10	1	0	0.964744	-1.941375	1.267996
11	1	0	0.472604	-2.596629	-0.287352
12	6	0	-0.099898	-0.560241	0.024567
13	6	0	1.780119	1.098108	0.015080
14	8	0	2.209423	2.274647	-0.022217
15	8	0	-1.334676	-0.855577	0.006130
16	19	0	-3.601057	0.319253	-0.008191

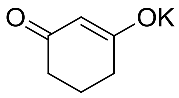

E(RB3LYP/6-31G(d,p)) = -983.305387703 Ha

ΔG (298.15 K, 1 atm, B3LYP/6-31G(d,p)) = -983.227434 Ha

DIPEA (N,N-Diisopropylethylamine)

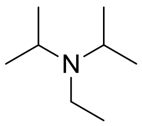

Center Number	Atomic Number	Atomic Type	Coordinates (Angstroms) X	Y	Z
1	6	0	1.162903	-0.752650	-0.116293
2	1	0	0.886604	-1.800653	-0.261480
3	6	0	0.113857	1.475906	-0.583543
4	1	0	1.058277	1.659270	-1.101086
5	1	0	-0.661441	1.847818	-1.262632
6	6	0	-1.289933	-0.397639	0.235103
7	1	0	-1.268247	-0.099596	1.300180
8	7	0	-0.052943	0.018738	-0.462414
9	6	0	-2.523946	0.267964	-0.398155
10	1	0	-3.431741	-0.105806	0.084862
11	1	0	-2.524508	1.355060	-0.291497
12	1	0	-2.580265	0.028811	-1.466084
13	6	0	-1.489943	-1.921914	0.200033
14	1	0	-0.756113	-2.465964	0.799529
15	1	0	-2.477734	-2.166037	0.602174
16	1	0	-1.439747	-2.294284	-0.829445
17	6	0	1.668037	-0.626380	1.337509
18	1	0	2.432138	-1.386462	1.536501
19	1	0	2.120400	0.351633	1.527405
20	1	0	0.859748	-0.772303	2.061018
21	6	0	2.306853	-0.494565	-1.107972
22	1	0	3.079618	-1.259553	-0.980357
23	1	0	1.944699	-0.542819	-2.139614
24	1	0	2.788819	0.476645	-0.955710
25	6	0	0.076566	2.337177	0.694853
26	1	0	0.899815	2.109795	1.376333
27	1	0	0.153594	3.395389	0.420225
28	1	0	-0.859674	2.210499	1.247368

E(RB3LYP/6-31G(d,p)) = -371.063401947 Ha

ΔG (298.15 K, 1 atm, B3LYP/6-31G(d,p)) = -370.847984 Ha

DIPEA-H$^+$ (N,N-Diisopropylethylammonium)

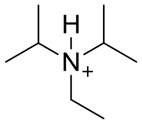

Center Number	Atomic Number	Atomic Type	Coordinates (Angstroms) X	Y	Z
1	6	0	1.259821	-0.736848	-0.065858
2	1	0	0.972363	-1.784472	-0.143657
3	6	0	0.117567	1.535340	-0.569502
4	1	0	1.128410	1.718965	-0.928272
5	1	0	-0.563877	1.855349	-1.357781
6	6	0	-1.313450	-0.436515	0.239577
7	1	0	-1.241698	-0.009551	1.240721
8	6	0	-2.530276	0.136378	-0.491754
9	1	0	-3.430438	-0.187581	0.035238
10	1	0	-2.545009	1.226657	-0.521007
11	1	0	-2.590588	-0.243938	-1.517318
12	6	0	-1.410631	-1.959550	0.341590
13	1	0	-0.652592	-2.398245	0.991948
14	1	0	-2.383989	-2.199353	0.776199
15	1	0	-1.362163	-2.438532	-0.641563
16	6	0	1.689519	-0.438969	1.366468
17	1	0	2.475931	-1.150464	1.631683
18	1	0	2.108677	0.564263	1.469674
19	1	0	0.878303	-0.564552	2.087310
20	6	0	2.372750	-0.490907	-1.084786
21	1	0	3.165363	-1.219584	-0.896552
22	1	0	2.028000	-0.639610	-2.112805
23	1	0	2.817183	0.503525	-0.999341
24	6	0	-0.152791	2.314648	0.711632
25	1	0	0.488163	2.016409	1.541159
26	1	0	0.058448	3.365078	0.492785
27	1	0	-1.194280	2.254097	1.033073
28	1	0	-0.158984	-0.285364	-1.458954
29	7	0	-0.027467	0.010777	-0.486678

E(RB3LYP/6-31G(d,p)) = -371.532379033 Ha

ΔG (298.15 K, 1 atm, B3LYP/6-31G(d,p)) = -371.301547 Ha

DIPEA-H^+I^- (N,N-Diisopropylethylammonium iodide)

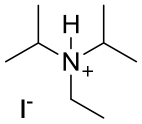

Center Number	Atomic Number	Atomic Type	Coordinates (Angstroms) X	Y	Z
1	6	0	-0.050072	0.035898	0.021982
2	1	0	-0.016323	0.126195	1.108723
3	6	0	1.784847	0.087138	-1.815929
4	1	0	1.260684	0.992086	-2.118300
5	1	0	2.848303	0.326128	-1.827279
6	6	0	2.094937	-1.392673	0.246627
7	1	0	1.629888	-2.238929	-0.262484
8	6	0	3.602182	-1.393492	-0.030478
9	1	0	4.037090	-2.261886	0.470128
10	1	0	3.845391	-1.476102	-1.091095
11	1	0	4.078838	-0.495585	0.374127
12	6	0	1.828531	-1.486083	1.752131
13	1	0	0.776841	-1.643152	1.996544
14	1	0	2.381494	-2.345071	2.139978
15	1	0	2.186448	-0.592954	2.273464
16	6	0	-0.930063	-1.157665	-0.346092
17	1	0	-1.910737	-0.995175	0.109852
18	1	0	-1.082286	-1.245121	-1.423261
19	1	0	-0.549821	-2.107959	0.033743
20	6	0	-0.603403	1.351695	-0.527350
21	1	0	-1.553797	1.549728	-0.024357
22	1	0	0.064915	2.191897	-0.319264
23	1	0	-0.805032	1.306453	-1.600900
24	6	0	1.484087	-1.061052	-2.771696
25	1	0	0.417047	-1.260748	-2.874029
26	1	0	1.860171	-0.770543	-3.756856
27	1	0	1.987194	-1.989237	-2.491279
28	53	0	3.086928	2.717098	1.183330
29	1	0	1.901856	0.669067	0.132000
30	7	0	1.439417	-0.135172	-0.350115

E(RB3LYP/6-31G(d,p):LANL2DZ) = -383.108113165 Ha

ΔG (298.15 K, 1 atm, B3LYP/6-31G(d,p):LANL2DZ) = -382.884539 Ha

6a (12-Acetyl-10-methyl-8-oxa-10-aza-tricyclo[7.3.1.0^{2,7}]trideca-2(7),11-dien-3-one) – conformation 1

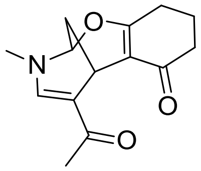

Center Number	Atomic Number	Atomic Type	Coordinates (Angstroms) X	Y	Z
1	7	0	-1.338447	1.968539	-0.182454
2	6	0	-1.929008	0.772964	-0.483314
3	6	0	-1.575566	-0.413274	0.091722
4	6	0	-0.396876	-0.386391	1.056761
5	6	0	-0.501500	0.914456	1.865010
6	6	0	-0.345654	2.050247	0.863707
7	6	0	-1.546675	3.150125	-1.013797
8	8	0	1.001279	2.031077	0.297981
9	1	0	-0.407846	3.037002	1.325121
10	6	0	1.553271	0.818638	0.041624
11	6	0	0.952387	-0.366221	0.343077
12	6	0	1.617525	-1.626502	-0.003781
13	8	0	1.140665	-2.720866	0.302601
14	6	0	2.919868	-1.547954	-0.794126
15	6	0	3.743223	-0.306094	-0.443303
16	6	0	2.898781	0.959333	-0.611936
17	1	0	-0.445499	-1.271678	1.692077
18	8	0	-2.153522	-2.647947	0.560663
19	6	0	-2.358054	-1.629050	-0.108696
20	6	0	-3.474568	-1.640052	-1.151066
21	1	0	-2.741940	0.847160	-1.197590
22	1	0	0.289799	0.997176	2.614838
23	1	0	-1.468392	1.001264	2.368199
24	1	0	-2.427883	3.003344	-1.640132
25	1	0	-0.683428	3.338155	-1.662085
26	1	0	-1.708436	4.029432	-0.383399
27	1	0	3.474577	-2.473806	-0.619410
28	1	0	2.655294	-1.528805	-1.861723
29	1	0	4.637969	-0.247318	-1.070372
30	1	0	4.083794	-0.376806	0.596678
31	1	0	2.741830	1.176477	-1.678526
32	1	0	3.398607	1.837464	-0.190192
33	1	0	-4.313020	-1.010130	-0.834786
34	1	0	-3.833286	-2.664591	-1.258857
35	1	0	-3.133307	-1.273573	-2.123917

E(RB3LYP/6-31G(d,p)) = -824.181858153 Ha

ΔG (298.15 K, 1 atm, B3LYP/6-31G(d,p)) = -823.945084 Ha

6a (12-Acetyl-10-methyl-8-oxa-10-aza-tricyclo[7.3.1.0^{2,7}]trideca-2(7),11-dien-3-one)– conformation 2 under computation 03-series

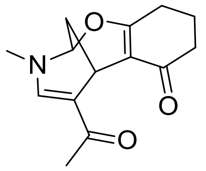

Center Number	Atomic Number	Atomic Type	Coordinates (Angstroms) X	Y	Z
1	6	0	-0.601502	1.980296	0.916588
2	6	0	-2.035043	0.562182	-0.438993
3	6	0	-1.508473	-0.586131	0.078025
4	6	0	-0.603861	0.815599	1.896579
5	1	0	-0.739553	2.946547	1.403659
6	1	0	-2.849815	0.547127	-1.154582
7	1	0	0.180544	0.973444	2.641464
8	7	0	-1.629329	1.817518	-0.082157
9	6	0	-2.011802	2.988827	-0.865021
10	1	0	-1.210442	3.293878	-1.547706
11	1	0	-2.902510	2.758384	-1.451570
12	1	0	-2.240537	3.824554	-0.197778
13	6	0	-2.077192	-1.894430	-0.233561
14	8	0	-1.687277	-2.920942	0.334342
15	6	0	-3.191206	-2.001473	-1.273422
16	1	0	-4.104865	-1.507435	-0.926289
17	1	0	-2.907182	-1.541362	-2.224865
18	1	0	-3.407377	-3.057847	-1.437864
19	6	0	-0.353150	-0.450342	1.063822
20	1	0	-0.311562	-1.347918	1.681720
21	6	0	3.297309	0.065241	-1.331108
22	6	0	2.746968	1.291198	-0.597528
23	6	0	3.272746	-1.157928	-0.413907
24	1	0	3.450137	1.623064	0.179900
25	1	0	2.612917	2.139755	-1.276081
26	1	0	2.682248	-0.128571	-2.217964
27	1	0	4.314437	0.262551	-1.682722
28	1	0	3.992107	-1.023240	0.407304
29	1	0	3.566117	-2.072424	-0.937514
30	6	0	1.910949	-1.406731	0.230355
31	6	0	1.425063	0.982872	0.046576
32	8	0	0.709111	2.110343	0.276519
33	8	0	1.629197	-2.530424	0.652567
34	6	0	1.002737	-0.267021	0.388001
35	1	0	-1.568854	0.782099	2.410127

E(RB3LYP/6-31G(d,p)) = -824.181740015 Ha

ΔG (298.15 K, 1 atm, B3LYP/6-31G(d,p)) = -823.944761 Ha

6a (12-Acetyl-10-methyl-8-oxa-10-aza-tricyclo[7.3.1.0^{2,7}]trideca-2(7),11-dien-3-one)– conformation 3

Center Number	Atomic Number	Atomic Type	Coordinates (Angstroms) X	Y	Z
1	6	0	-0.455074	2.043630	0.871077
2	6	0	-1.977412	0.749075	-0.514798
3	6	0	-1.593341	-0.443119	0.028105
4	6	0	-0.584721	0.882626	1.845605
5	1	0	-0.543445	3.017305	1.354965
6	1	0	-2.782087	0.774567	-1.242260
7	1	0	0.195037	0.971922	2.606391
8	7	0	-1.442293	1.957290	-0.178250
9	6	0	-1.716249	3.159151	-0.960116
10	1	0	-0.859926	3.427691	-1.588818
11	1	0	-2.579931	2.982997	-1.602628
12	1	0	-1.939410	3.998830	-0.295208
13	6	0	-2.360386	-1.637143	-0.344085
14	8	0	-3.240481	-1.605966	-1.216023
15	6	0	-2.078601	-2.934735	0.391843
16	1	0	-2.369603	-2.839833	1.445531
17	1	0	-2.661907	-3.737054	-0.063074
18	1	0	-1.011921	-3.174832	0.372740
19	6	0	-0.429658	-0.401928	1.015911
20	1	0	-0.437729	-1.280138	1.663407
21	6	0	3.739173	-0.147102	-0.383708
22	6	0	2.979314	-1.422781	-0.754219
23	6	0	2.846007	1.080609	-0.576740
24	1	0	2.749504	-1.419878	-1.829984
25	1	0	3.564580	-2.324731	-0.556202
26	1	0	4.055215	-0.202300	0.664834
27	1	0	4.646233	-0.050933	-0.987793
28	1	0	2.700900	1.285172	-1.647381
29	1	0	3.298690	1.981244	-0.149827
30	6	0	1.494771	0.885391	0.050788
31	6	0	1.654886	-1.552754	-0.011684
32	8	0	1.209556	-2.672194	0.262484
33	8	0	0.895757	2.072061	0.309182
34	6	0	0.933433	-0.326005	0.328622
35	1	0	-1.559658	0.928250	2.338318

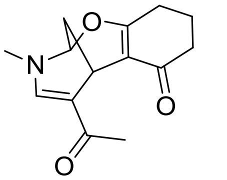

E(RB3LYP/6-31G(d,p)) = -824.183242168 Ha

ΔG (298.15 K, 1 atm, B3LYP/6-31G(d,p)) = -823.945751 Ha

6a (12-Acetyl-10-methyl-8-oxa-10-aza-tricyclo[7.3.1.0^{2,7}]trideca-2(7),11-dien-3-one)– conformation 4

Center Number	Atomic Number	Atomic Type	Coordinates (Angstroms) X	Y	Z
1	6	0	0.646930	-1.983863	0.916700
2	6	0	2.030834	-0.594340	-0.514184
3	6	0	1.539419	0.570256	0.001305
4	6	0	0.687432	-0.799235	1.869642
5	1	0	0.808239	-2.939101	1.417936
6	1	0	2.818648	-0.560875	-1.259499
7	1	0	-0.070378	-0.938934	2.644898
8	7	0	1.627718	-1.839398	-0.131032
9	6	0	2.001488	-3.029703	-0.889037
10	1	0	1.174183	-3.378406	-1.517478
11	1	0	2.853450	-2.797239	-1.529319
12	1	0	2.286213	-3.835368	-0.206087
13	6	0	2.164612	1.822947	-0.437688
14	8	0	3.031046	1.849394	-1.323261
15	6	0	1.751150	3.114766	0.244692
16	1	0	0.663464	3.225174	0.270594
17	1	0	2.097996	3.113968	1.285905
18	1	0	2.211445	3.955889	-0.276472
19	6	0	0.410053	0.453257	1.021951
20	1	0	0.365562	1.341096	1.654560
21	6	0	-3.337220	-0.123143	-1.226596
22	6	0	-2.757452	-1.324994	-0.476491
23	6	0	-3.255273	1.135306	-0.361205
24	1	0	-3.426383	-1.627440	0.342119
25	1	0	-2.655488	-2.196757	-1.130529
26	1	0	-2.769994	0.031498	-2.152076
27	1	0	-4.373190	-0.324647	-1.514707
28	1	0	-3.920873	1.033886	0.508717
29	1	0	-3.578325	2.029702	-0.901189
30	6	0	-1.854528	1.397803	0.180880
31	6	0	-1.409335	-1.004515	0.104591
32	8	0	-0.693461	-2.127943	0.343032
33	8	0	-1.520274	2.549204	0.480718
34	6	0	-0.965952	0.254206	0.386874
35	1	0	1.669976	-0.754391	2.347328

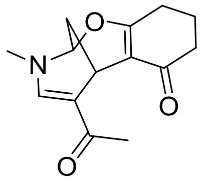

E(RB3LYP/6-31G(d,p)) = -824.183085531 Ha

ΔG (298.15 K, 1 atm, B3LYP/6-31G(d,p)) = -823.945874 Ha

6aa (10-Acetyl-12-methyl-8-oxa-12-aza-tricyclo[7.3.1.0 2,7]trideca-2(7),10-dien-3-one) – conformation 1

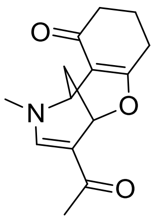

Center Number	Atomic Number	Atomic Type	X	Y	Z
1	6	0	0.425597	1.134242	1.015610
2	6	0	-1.569657	1.232527	-0.349790
3	6	0	-1.967235	-0.016547	0.085024
4	6	0	-0.447724	0.374245	2.017627
5	1	0	1.053776	1.881901	1.502404
6	1	0	-2.155517	1.782096	-1.080007
7	1	0	0.175607	-0.101700	2.778991
8	7	0	-0.465617	1.859930	0.080425
9	6	0	-0.106965	3.210518	-0.337001
10	1	0	-0.233799	3.921052	0.488454
11	1	0	0.937249	3.226809	-0.656115
12	1	0	-0.748262	3.518604	-1.164527
13	6	0	-3.135540	-0.699966	-0.441252
14	8	0	-3.487707	-1.803038	0.002824
15	6	0	-3.948833	-0.055167	-1.559810
16	1	0	-4.362904	0.909849	-1.248496
17	1	0	-3.337360	0.120684	-2.450731
18	1	0	-4.771123	-0.723210	-1.819418
19	6	0	-1.203009	-0.667876	1.203128
20	1	0	-1.871696	-1.282936	1.803590
21	6	0	3.267084	-1.855624	-0.427675
22	6	0	1.790777	-2.260238	-0.386532
23	6	0	3.424476	-0.450686	-1.016149
24	1	0	1.416517	-2.452625	-1.402243
25	1	0	1.638993	-3.185686	0.177967
26	1	0	3.673784	-1.871690	0.590442
27	1	0	3.838315	-2.582535	-1.012662
28	1	0	3.123619	-0.453201	-2.074229
29	1	0	4.460794	-0.103107	-0.983543
30	6	0	2.559895	0.583985	-0.306718
31	6	0	0.938278	-1.184681	0.227281
32	8	0	-0.222524	-1.659139	0.694556
33	8	0	2.904100	1.771780	-0.276974
34	6	0	1.310227	0.131056	0.294337
35	1	0	-1.153859	1.044633	2.515432

E(RB3LYP/6-31G(d,p)) = -824.186446616 Ha

ΔG (298.15 K, 1 atm, B3LYP/6-31G(d,p)) = -823.948765 Ha

6aa (10-Acetyl-12-methyl-8-oxa-12-aza-tricyclo[7.3.1.0 2,7]trideca-2(7),10-dien-3-one) – conformation 2

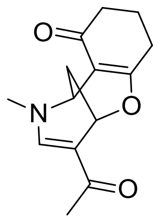

Center Number	Atomic Number	Atomic Type	Coordinates (Angstroms) X	Y	Z
1	6	0	0.448195	1.176667	0.973380
2	6	0	-1.525754	1.236265	-0.428678
3	6	0	-1.972021	0.035215	0.086311
4	6	0	-0.458287	0.498095	2.003763
5	1	0	1.080444	1.942708	1.425170
6	1	0	-2.082932	1.751431	-1.204862
7	1	0	0.143989	0.056523	2.801918
8	7	0	-0.407491	1.856712	-0.025763
9	6	0	0.032683	3.134413	-0.573281
10	1	0	-0.017126	3.920862	0.188710
11	1	0	1.066050	3.051134	-0.918493
12	1	0	-0.610513	3.415232	-1.408728
13	6	0	-3.163069	-0.637915	-0.400262
14	8	0	-3.555359	-1.697760	0.110520
15	6	0	-3.951585	-0.036085	-1.559839
16	1	0	-4.328320	0.962222	-1.313085
17	1	0	-3.334014	0.057752	-2.459003
18	1	0	-4.798531	-0.688105	-1.777382
19	6	0	-1.227755	-0.572051	1.241816
20	1	0	-1.911207	-1.142212	1.869209
21	6	0	2.806933	-1.829490	-1.193065
22	6	0	3.571933	-0.621465	-0.644813
23	6	0	1.771231	-2.324160	-0.178121
24	1	0	4.193457	-0.928882	0.209175
25	1	0	4.249217	-0.191758	-1.388194
26	1	0	2.294383	-1.546802	-2.120243
27	1	0	3.499231	-2.638508	-1.444125
28	1	0	2.271458	-2.790602	0.682499
29	1	0	1.117272	-3.087442	-0.610256
30	6	0	0.919803	-1.192774	0.328608
31	6	0	2.652799	0.488018	-0.148318
32	8	0	3.041944	1.662026	-0.134144
33	8	0	-0.268059	-1.608646	0.782745
34	6	0	1.332891	0.112429	0.346632
35	1	0	-1.156610	1.211523	2.450262

E(RB3LYP/6-31G(d,p)) = 824.186098441 Ha

ΔG (298.15 K, 1 atm, B3LYP/6-31G(d,p)) = -823.949242 Ha

6aa (10-Acetyl-12-methyl-8-oxa-12-aza-tricyclo[7.3.1.0 2,7]trideca-2(7),10-dien-3-one) – conformation 3

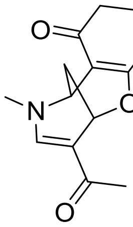

Center Number	Atomic Number	Atomic Type	X	Y	Z
1	6	0	0.452012	1.156691	1.000274
2	6	0	-1.497538	1.340979	-0.409864
3	6	0	-1.969663	0.114466	0.018125
4	6	0	-0.483358	0.452611	1.985717
5	1	0	1.108078	1.872465	1.498010
6	1	0	-2.058659	1.880752	-1.166947
7	1	0	0.094893	-0.048334	2.766172
8	7	0	-0.380405	1.921141	0.041600
9	6	0	0.056906	3.246182	-0.382946
10	1	0	-0.037909	3.968377	0.436458
11	1	0	1.103415	3.202311	-0.691903
12	1	0	-0.560272	3.581237	-1.218062
13	6	0	-3.151517	-0.433542	-0.633756
14	8	0	-3.734567	0.149225	-1.560342
15	6	0	-3.679833	-1.777504	-0.147541
16	1	0	-2.883303	-2.525880	-0.088202
17	1	0	-4.114353	-1.684041	0.854991
18	1	0	-4.456133	-2.122411	-0.832050
19	6	0	-1.272361	-0.561844	1.164159
20	1	0	-1.962468	-1.131199	1.786504
21	6	0	3.168013	-1.984549	-0.366872
22	6	0	1.673385	-2.317194	-0.344213
23	6	0	3.402119	-0.594353	-0.964811
24	1	0	1.305007	-2.503736	-1.363192
25	1	0	1.469154	-3.227633	0.228192
26	1	0	3.558649	-2.011078	0.657284
27	1	0	3.711625	-2.743577	-0.937130
28	1	0	3.115984	-0.591655	-2.026955
29	1	0	4.453533	-0.297215	-0.920229
30	6	0	2.579075	0.486846	-0.276303
31	6	0	0.863839	-1.195300	0.243855
32	8	0	-0.327444	-1.609908	0.696300
33	8	0	2.981312	1.655889	-0.245924
34	6	0	1.296947	0.101465	0.305159
35	1	0	-1.166979	1.162265	2.459693

E(RB3LYP/6-31G(d,p)) = -824.185658689 Ha

ΔG (298.15 K, 1 atm, B3LYP/6-31G(d,p)) = -823.947804 Ha

6aa (10-Acetyl-12-methyl-8-oxa-12-aza-tricyclo[7.3.1.0 2,7]trideca-2(7),10-dien-3-one) – conformation 4

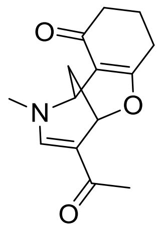

Center Number	Atomic Number	Atomic Type	Coordinates (Angstroms) X	Y	Z
1	6	0	0.475017	1.196869	0.957743
2	6	0	-1.454861	1.340437	-0.487653
3	6	0	-1.971871	0.161417	0.015056
4	6	0	-0.488830	0.571803	1.968232
5	1	0	1.135294	1.930947	1.422216
6	1	0	-1.990264	1.845394	-1.286112
7	1	0	0.069540	0.105961	2.784108
8	7	0	-0.324519	1.914656	-0.061899
9	6	0	0.185313	3.168678	-0.604032
10	1	0	0.162990	3.956849	0.157296
11	1	0	1.218145	3.034821	-0.934533
12	1	0	-0.433224	3.478144	-1.447742
13	6	0	-3.169382	-0.386161	-0.607638
14	8	0	-3.734747	0.161626	-1.566051
15	6	0	-3.736861	-1.685296	-0.049201
16	1	0	-2.964227	-2.455379	0.041797
17	1	0	-4.158438	-1.527336	0.950683
18	1	0	-4.529963	-2.039002	-0.709483
19	6	0	-1.292257	-0.469538	1.196825
20	1	0	-1.994397	-0.994478	1.844033
21	6	0	2.732580	-1.938301	-1.136468
22	6	0	3.541306	-0.763893	-0.578395
23	6	0	1.651803	-2.374655	-0.141898
24	1	0	4.123088	-1.093513	0.295093
25	1	0	4.258379	-0.374599	-1.306454
26	1	0	2.255267	-1.638860	-2.077029
27	1	0	3.390835	-2.781464	-1.365703
28	1	0	2.109415	-2.859361	0.732230
29	1	0	0.970758	-3.107878	-0.584209
30	6	0	0.845431	-1.200313	0.339877
31	6	0	2.664104	0.392807	-0.115542
32	8	0	3.105909	1.547649	-0.105125
33	8	0	-0.368908	-1.558542	0.777596
34	6	0	1.317361	0.084431	0.355411
35	1	0	-1.162708	1.321583	2.391836

E(RB3LYP/6-31G(d,p)) = -824.185262970 Ha

ΔG (298.15 K, 1 atm, B3LYP/6-31G(d,p)) = -823.947919 Ha

6a-K – conformation 1

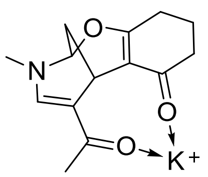

1	6	0	-2.429709	-0.689960	1.050365
2	6	0	-1.108912	-2.081986	-0.448362
3	6	0	0.074661	-1.524374	-0.042716
4	6	0	-1.165343	-0.641046	1.894033
5	1	0	-3.326899	-0.890634	1.636762
6	1	0	-1.142005	-2.906757	-1.151431
7	1	0	-1.274155	0.142797	2.647956
8	7	0	-2.327516	-1.688127	0.008328
9	6	0	-3.566977	-2.148008	-0.614109
10	1	0	-4.044406	-1.338598	-1.176215
11	1	0	-3.346780	-2.967741	-1.298630
12	1	0	-4.263206	-2.505201	0.150130
13	6	0	1.349420	-2.091219	-0.437710
14	8	0	2.421238	-1.668718	0.031462
15	6	0	1.399149	-3.252050	-1.423912
16	1	0	1.033719	-4.173197	-0.957536
17	1	0	0.792498	-3.066167	-2.314595
18	1	0	2.436693	-3.404414	-1.723997
19	6	0	-0.012286	-0.348211	0.923484
20	1	0	0.938343	-0.248160	1.448611
21	6	0	-0.961679	3.705692	-0.526330
22	6	0	0.358648	3.101734	-1.011220
23	6	0	-2.073548	2.654872	-0.557753
24	1	0	0.276377	2.828319	-2.073707
25	1	0	1.189765	3.806817	-0.926072
26	1	0	-0.839069	4.071990	0.499835
27	1	0	-1.239379	4.565069	-1.143267
28	1	0	-2.372397	2.442167	-1.594358
29	1	0	-2.974494	3.002830	-0.042839
30	6	0	-1.634752	1.364107	0.072276
31	6	0	0.735557	1.839572	-0.250563
32	8	0	1.929540	1.560822	-0.074956
33	8	0	-2.694447	0.624057	0.468926
34	6	0	-0.335641	0.972467	0.228614
35	1	0	-1.031135	-1.598673	2.404117
36	19	0	4.129314	0.250400	0.474574

E(RB3LYP/6-31G(d,p)) = -1424.05229111 Ha

ΔG (298.15 K, 1 atm, B3LYP/6-31G(d,p)) = -1423.818632 Ha

E(RB3LYP/6-311++G(d,p)//B3LYP/6-31G(d,p)) = -1424.28665584 Ha

ΔG (298.15 K, 1 atm, B3LYP/6-311++G(d,p)//B3LYP/6-31G(d,p)) = -1424.052997 Ha

6a-K – conformation 2

Center Number	Atomic Number	Atomic Type	Coordinates (Angstroms) X	Y	Z
1	6	0	-2.482405	-0.518039	1.071602
2	6	0	-1.283149	-1.995463	-0.439550
3	6	0	-0.059573	-1.523671	-0.044193
4	6	0	-1.219179	-0.576664	1.915451
5	1	0	-3.394140	-0.632367	1.658425
6	1	0	-1.379397	-2.803680	-1.155820
7	1	0	-1.262775	0.205641	2.677458
8	7	0	-2.467613	-1.530681	0.040485
9	6	0	-3.741040	-1.889290	-0.580024
10	1	0	-4.144222	-1.051557	-1.158909
11	1	0	-3.592377	-2.738249	-1.248165
12	1	0	-4.467299	-2.169657	0.187996
13	6	0	1.168998	-2.152523	-0.489625
14	8	0	2.276705	-1.790242	-0.054769
15	6	0	1.124748	-3.299900	-1.491481
16	1	0	0.685841	-4.195906	-1.040062
17	1	0	0.534148	-3.050315	-2.377635
18	1	0	2.146434	-3.529228	-1.796833
19	6	0	-0.044189	-0.366813	0.948733
20	1	0	0.911385	-0.361545	1.474202
21	6	0	-0.696746	3.329976	-1.354328
22	6	0	-1.842070	2.821701	-0.475509
23	6	0	0.632143	3.231804	-0.604328
24	1	0	-2.036601	3.523521	0.348138
25	1	0	-2.776547	2.736716	-1.038568
26	1	0	-0.647897	2.725516	-2.267767
27	1	0	-0.888068	4.362101	-1.661312
28	1	0	0.641979	3.941404	0.236288
29	1	0	1.484351	3.488414	-1.240066
30	6	0	0.890201	1.852292	-0.014518
31	6	0	-1.515767	1.478681	0.112046
32	8	0	-2.632195	0.806809	0.467597
33	8	0	2.053710	1.507507	0.236865
34	6	0	-0.249927	0.998640	0.297584
35	1	0	-1.165490	-1.547205	2.416005
36	19	0	4.155049	-0.038200	0.389186

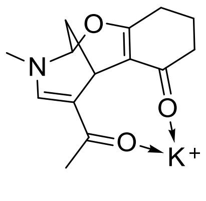

E(RB3LYP/6-31G(d,p)) = -1424.05232348 Ha

ΔG (298.15 K, 1 atm, B3LYP/6-31G(d,p)) = -1423.818707 Ha

E(RB3LYP/6-311++G(d,p)//B3LYP/6-31G(d,p)) = -1424.28677180 Ha

ΔG (298.15 K, 1 atm, B3LYP/6-311++G(d,p)//B3LYP/6-31G(d,p)) = -1424.053156 Ha

6a-K – conformation 3

Center Number	Atomic Number	Atomic Type	X	Y	Z
1	6	0	-2.755491	0.413767	0.866345
2	6	0	-2.129232	-1.478565	-0.521684
3	6	0	-0.882106	-1.575084	0.025288
4	6	0	-1.732423	-0.143155	1.843776
5	1	0	-3.689573	0.706414	1.346912
6	1	0	-2.454337	-2.212567	-1.251606
7	1	0	-1.523469	0.612207	2.605632
8	7	0	-3.047027	-0.528544	-0.184402
9	6	0	-4.265704	-0.330157	-0.964054
10	1	0	-4.190606	0.560092	-1.598187
11	1	0	-4.432549	-1.200147	-1.600272
12	1	0	-5.124506	-0.214019	-0.296620
13	6	0	-0.069373	-2.739755	-0.342691
14	8	0	-0.419502	-3.531514	-1.228595
15	6	0	1.219776	-2.987742	0.420397
16	1	0	0.995255	-3.249080	1.462097
17	1	0	1.756947	-3.817134	-0.042698
18	1	0	1.840379	-2.088328	0.439013
19	6	0	-0.481966	-0.485950	1.018492
20	1	0	0.322159	-0.829659	1.670698
21	6	0	0.857437	3.477494	-0.375106
22	6	0	1.757993	2.293330	-0.733262
23	6	0	-0.613058	3.108567	-0.577859
24	1	0	1.690996	2.084334	-1.811313
25	1	0	2.809369	2.499989	-0.514044
26	1	0	1.019572	3.754514	0.673244
27	1	0	1.113852	4.350282	-0.982299
28	1	0	-0.849253	3.042148	-1.649727
29	1	0	-1.282832	3.866967	-0.160963
30	6	0	-0.943430	1.785974	0.052651
31	6	0	1.367042	1.015527	-0.006661
32	8	0	2.235799	0.165106	0.253446
33	8	0	-2.263626	1.676571	0.303123
34	6	0	-0.031018	0.806628	0.335951
35	1	0	-2.143517	-1.029078	2.334820
36	19	0	4.803118	-0.451774	-0.010903

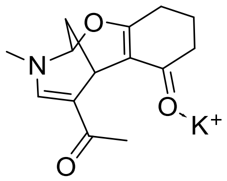

E(RB3LYP/6-31G(d,p)) = -1424.04202442 Ha

ΔG (298.15 K, 1 atm, B3LYP/6-31G(d,p)) = -1423.808622 Ha

E(RB3LYP/6-311++G(d,p)//B3LYP/6-31G(d,p)) = -1424.27796125 Ha

ΔG (298.15 K, 1 atm, B3LYP/6-311++G(d,p)//B3LYP/6-31G(d,p)) = -1424.044559 Ha

6a-K – conformation 4

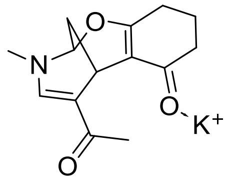

Center Number	Atomic Number	Atomic Type	Coordinates (Angstroms) X	Y	Z
1	6	0	2.620105	-0.529302	1.091518
2	6	0	2.322563	1.341464	-0.420049
3	6	0	1.021406	1.535314	-0.055398
4	6	0	1.530393	0.137719	1.915364
5	1	0	3.452611	-0.890275	1.696046
6	1	0	2.785272	2.015327	-1.133312
7	1	0	1.169377	-0.566838	2.668926
8	7	0	3.128223	0.362173	0.081369
9	6	0	4.419256	0.049953	-0.525701
10	1	0	4.358622	-0.848106	-1.150566
11	1	0	4.737459	0.888244	-1.146743
12	1	0	5.169646	-0.113719	0.252882
13	6	0	0.331283	2.706182	-0.608953
14	8	0	0.853638	3.432944	-1.465033
15	6	0	-1.053549	3.043340	-0.084040
16	1	0	-1.715009	2.173331	-0.116534
17	1	0	-0.989945	3.359064	0.964921
18	1	0	-1.468236	3.862779	-0.673356
19	6	0	0.418964	0.537076	0.930776
20	1	0	-0.422987	0.978924	1.465418
21	6	0	-0.791927	-3.005822	-1.394293
22	6	0	0.442037	-3.124437	-0.497264
23	6	0	-1.911677	-2.264634	-0.662602
24	1	0	0.255004	-3.829851	0.325029
25	1	0	1.305836	-3.510199	-1.047117
26	1	0	-0.524092	-2.457670	-2.305110
27	1	0	-1.129218	-3.999191	-1.703286
28	1	0	-2.286269	-2.877394	0.170803
29	1	0	-2.766602	-2.067188	-1.316153
30	6	0	-1.464493	-0.938359	-0.065606
31	6	0	0.810017	-1.794292	0.095610
32	8	0	2.103438	-1.757934	0.469810
33	8	0	-2.311370	-0.059195	0.171998
34	6	0	-0.062196	-0.754274	0.268971
35	1	0	1.949052	1.009151	2.425811
36	19	0	-4.880886	0.514333	0.353082

E(RB3LYP/6-31G(d,p)) = -1424.04202776 Ha

ΔG (298.15 K, 1 atm, B3LYP/6-31G(d,p)) = -1423.809590 Ha

E(RB3LYP/6-311++G(d,p)//B3LYP/6-31G(d,p)) = -1424.27808357 Ha

ΔG (298.15 K, 1 atm, B3LYP/6-311++G(d,p)//B3LYP/6-31G(d,p)) = -1424.045646 Ha

6a-K – conformation 5

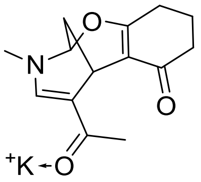

Center Number	Atomic Number	Atomic Type	Coordinates (Angstroms) X	Y	Z
1	6	0	1.511590	2.156163	0.686975
2	6	0	-0.742717	1.618160	-0.043754
3	6	0	-0.761284	0.423037	0.626216
4	6	0	1.184917	1.274303	1.881412
5	1	0	1.993436	3.092933	0.968639
6	1	0	-1.628199	1.952013	-0.574066
7	1	0	2.108599	1.049794	2.420623
8	7	0	0.318486	2.463173	-0.073520
9	6	0	0.357299	3.622955	-0.960828
10	1	0	1.080065	3.470055	-1.769184
11	1	0	-0.629847	3.777624	-1.397411
12	1	0	0.639644	4.519151	-0.400435
13	6	0	-2.011544	-0.320389	0.653195
14	8	0	-3.009399	0.044590	-0.002150
15	6	0	-2.109237	-1.553284	1.528155
16	1	0	-1.264288	-2.227175	1.361936
17	1	0	-2.084388	-1.259635	2.585073
18	1	0	-3.053049	-2.064863	1.330770
19	6	0	0.532597	0.001685	1.317991
20	1	0	0.330713	-0.711233	2.118559
21	6	0	3.152698	-1.726471	-1.777233
22	6	0	3.519070	-0.351659	-1.211887
23	6	0	2.720614	-2.668632	-0.652204
24	1	0	4.462642	-0.405826	-0.650198
25	1	0	3.673820	0.382980	-2.008272
26	1	0	2.331963	-1.613296	-2.495523
27	1	0	4.002373	-2.145702	-2.323947
28	1	0	3.576845	-2.883688	0.004047
29	1	0	2.366706	-3.630836	-1.032592
30	6	0	1.623795	-2.082592	0.228963
31	6	0	2.447400	0.159871	-0.291798
32	8	0	2.499276	1.507945	-0.170381
33	8	0	0.860559	-2.836589	0.842213
34	6	0	1.548304	-0.627478	0.364974
35	1	0	0.510531	1.810875	2.554225
36	19	0	-5.331129	-0.507208	-1.033479

E(RB3LYP/6-31G(d,p)) = -1424.04363714 Ha

ΔG (298.15 K, 1 atm, B3LYP/6-31G(d,p)) = -1423.811672 Ha

E(RB3LYP/6-311++G(d,p)//B3LYP/6-31G(d,p)) = -1424.27925201 Ha

ΔG (298.15 K, 1 atm, B3LYP/6-311++G(d,p)//B3LYP/6-31G(d,p)) = -1424.047287 Ha

6a-K – conformation 6

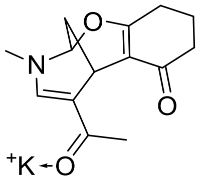

Center Number	Atomic Number	Atomic Type	Coordinates (Angstroms) X	Y	Z
1	6	0	1.505699	2.147988	0.676612
2	6	0	-0.736697	1.605250	-0.099565
3	6	0	-0.776523	0.419839	0.586506
4	6	0	1.153132	1.266421	1.864513
5	1	0	1.964955	3.092712	0.969790
6	1	0	-1.612059	1.944217	-0.643295
7	1	0	2.067094	1.043819	2.420928
8	7	0	0.335832	2.435935	-0.127931
9	6	0	0.397388	3.588036	-1.024095
10	1	0	1.153792	3.434418	-1.800672
11	1	0	-0.572593	3.726969	-1.502362
12	1	0	0.647679	4.493395	-0.462851
13	6	0	-2.040108	-0.299585	0.629137
14	8	0	-3.029112	0.066616	-0.039028
15	6	0	-2.161812	-1.506584	1.536473
16	1	0	-2.095500	-1.192971	2.585598
17	1	0	-3.128355	-1.987387	1.375143
18	1	0	-1.346870	-2.213093	1.357110
19	6	0	0.512279	-0.004657	1.286058
20	1	0	0.306248	-0.725250	2.078692
21	6	0	3.817752	-1.832121	-0.977881
22	6	0	2.506117	-2.620071	-0.944895
23	6	0	3.540892	-0.343442	-1.200525
24	1	0	2.022415	-2.579932	-1.932140
25	1	0	2.664453	-3.676331	-0.711409
26	1	0	4.345601	-1.962645	-0.025765
27	1	0	4.475248	-2.213566	-1.764612
28	1	0	3.220442	-0.162328	-2.236684
29	1	0	4.439376	0.262578	-1.046118
30	6	0	2.464752	0.161449	-0.282321
31	6	0	1.511724	-2.062171	0.065524
32	8	0	0.702308	-2.816154	0.615864
33	8	0	2.534537	1.506678	-0.132978
34	6	0	1.529727	-0.622027	0.326736
35	1	0	0.464308	1.800172	2.524685
36	19	0	-5.388150	-0.504390	-0.973424

E(RB3LYP/6-31G(d,p)) = -1424.04376653 Ha

ΔG (298.15 K, 1 atm, B3LYP/6-31G(d,p)) = -1423.811755 Ha

E(RB3LYP/6-311++G(d,p)//B3LYP/6-31G(d,p)) = -1424.27934186 Ha

ΔG (298.15 K, 1 atm, B3LYP/6-311++G(d,p)//B3LYP/6-31G(d,p)) = -1424.047331 Ha

6aa-K – conformation 1

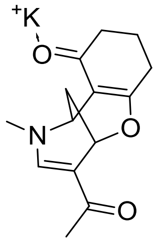

Center Number	Atomic Number	Atomic Type	Coordinates (Angstroms) X	Y	Z
1	6	0	-0.011858	-0.672217	1.130968
2	6	0	1.734823	-1.570069	-0.286024
3	6	0	2.586336	-0.537021	0.049607
4	6	0	1.132792	-0.229039	2.046482
5	1	0	-0.836706	-1.112196	1.693472
6	1	0	2.038362	-2.335038	-0.993716
7	1	0	0.768680	0.485190	2.789382
8	7	0	0.501577	-1.721639	0.220206
9	6	0	-0.353286	-2.856665	-0.107006
10	1	0	-0.515143	-3.487035	0.774977
11	1	0	-1.322229	-2.500029	-0.464078
12	1	0	0.122360	-3.458191	-0.883125
13	6	0	3.897108	-0.365778	-0.554210
14	8	0	4.652460	0.549102	-0.195189
15	6	0	4.354885	-1.324576	-1.648745
16	1	0	4.392508	-2.357422	-1.286466
17	1	0	3.678521	-1.301869	-2.509365
18	1	0	5.353111	-1.028866	-1.973868
19	6	0	2.171255	0.411844	1.136104
20	1	0	3.046790	0.779880	1.668454
21	6	0	-1.638463	3.086890	-0.411080
22	6	0	-0.119971	2.905388	-0.471408
23	6	0	-2.351401	1.811267	-0.868005
24	1	0	0.222933	2.875650	-1.515501
25	1	0	0.408609	3.737453	0.003511
26	1	0	-1.933897	3.319943	0.618739
27	1	0	-1.939733	3.935415	-1.031848
28	1	0	-2.159860	1.636719	-1.937135
29	1	0	-3.436106	1.885690	-0.748910
30	6	0	-1.876380	0.573732	-0.123008
31	6	0	0.313000	1.627921	0.191531
32	8	0	1.588654	1.661452	0.575627
33	8	0	-2.639324	-0.398554	0.023452
34	6	0	-0.514740	0.547120	0.374078
35	1	0	1.572327	-1.082090	2.570465
36	19	0	-5.143710	-1.204764	-0.226487

E(RB3LYP/6-31G(d,p)) = -1424.04503397 Ha

ΔG (298.15 K, 1 atm, B3LYP/6-31G(d,p)) =-1423.812410 Ha

E(RB3LYP/6-311++G(d,p)//B3LYP/6-31G(d,p)) = -1424.28223246 Ha

ΔG (298.15 K, 1 atm, B3LYP/6-311++G(d,p)//B3LYP/6-31G(d,p)) = -1424.049608 Ha

6aa-K – conformation 2

Center Number	Atomic Number	Atomic Type	Coordinates (Angstroms) X	Y	Z
1	6	0	-0.029504	-0.773511	1.043219
2	6	0	1.771604	-1.531277	-0.390760
3	6	0	2.608652	-0.534643	0.067851
4	6	0	1.082724	-0.397126	2.026014
5	1	0	-0.861849	-1.272436	1.541876
6	1	0	2.100843	-2.227304	-1.155557
7	1	0	0.685918	0.248157	2.813933
8	7	0	0.522422	-1.730150	0.057478
9	6	0	-0.340891	-2.787321	-0.454487
10	1	0	-0.606345	-3.488516	0.344471
11	1	0	-1.261473	-2.358915	-0.859982
12	1	0	0.180499	-3.334337	-1.241311
13	6	0	3.942079	-0.313998	-0.466221
14	8	0	4.677296	0.571616	-0.006264
15	6	0	4.449047	-1.182342	-1.613363
16	1	0	4.469802	-2.241660	-1.336476
17	1	0	3.812616	-1.085691	-2.499067
18	1	0	5.460963	-0.864426	-1.867576
19	6	0	2.140936	0.325543	1.205137
20	1	0	2.990290	0.666089	1.794950
21	6	0	-1.330593	2.778367	-1.194670
22	6	0	-2.432755	1.918699	-0.571015
23	6	0	-0.152997	2.931165	-0.227688
24	1	0	-2.891460	2.452469	0.274484
25	1	0	-3.235609	1.706829	-1.283296
26	1	0	-0.978485	2.306749	-2.119582
27	1	0	-1.722716	3.762842	-1.465024
28	1	0	-0.438353	3.561664	0.626340
29	1	0	0.701726	3.418831	-0.705077
30	6	0	0.291604	1.597131	0.303945
31	6	0	-1.920504	0.593617	-0.027347
32	8	0	-2.696441	-0.372357	0.089906
33	8	0	1.561023	1.604375	0.707619
34	6	0	-0.535758	0.503789	0.392061
35	1	0	1.518046	-1.285621	2.491387
36	19	0	-5.219908	-1.107963	-0.139105

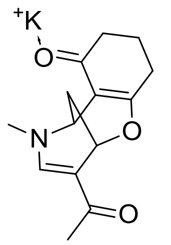

E(RB3LYP/6-31G(d,p)) = -1424.04484735 Ha

ΔG (298.15 K, 1 atm, B3LYP/6-31G(d,p)) = -1423.812539 Ha

E(RB3LYP/6-311++G(d,p)//B3LYP/6-31G(d,p)) = -1424.28209468 Ha

ΔG (298.15 K, 1 atm, B3LYP/6-311++G(d,p)//B3LYP/6-31G(d,p)) = -1424.049787 Ha

6aa-K – conformation 3

Center Number	Atomic Number	Atomic Type	X	Y	Z
1	6	0	-0.026995	-0.719634	1.121389
2	6	0	1.651311	-1.651050	-0.342499
3	6	0	2.548070	-0.652630	-0.015057
4	6	0	1.151143	-0.340817	2.021735
5	1	0	-0.859280	-1.133061	1.692929
6	1	0	1.942970	-2.391671	-1.081056
7	1	0	0.831619	0.372078	2.785923
8	7	0	0.428735	-1.774894	0.186861
9	6	0	-0.468707	-2.877702	-0.136796
10	1	0	-0.638699	-3.508761	0.743011
11	1	0	-1.430038	-2.485744	-0.476914
12	1	0	-0.022776	-3.487535	-0.923775
13	6	0	3.808358	-0.591455	-0.745436
14	8	0	4.093006	-1.388746	-1.650924
15	6	0	4.801630	0.501143	-0.371143
16	1	0	4.324015	1.485542	-0.339275
17	1	0	5.229070	0.314166	0.621193
18	1	0	5.610877	0.510412	-1.102644
19	6	0	2.198500	0.282436	1.105526
20	1	0	3.077143	0.608539	1.661027
21	6	0	-1.536026	3.123847	-0.332620
22	6	0	-0.025038	2.893112	-0.409789
23	6	0	-2.294501	1.882259	-0.809377
24	1	0	0.308724	2.873926	-1.457108
25	1	0	0.534801	3.696892	0.077938
26	1	0	-1.815361	3.345442	0.704195
27	1	0	-1.813561	3.994421	-0.933549
28	1	0	-2.116386	1.723116	-1.883210
29	1	0	-3.375270	1.989935	-0.680250
30	6	0	-1.855317	0.615061	-0.093180
31	6	0	0.370574	1.588363	0.222282
32	8	0	1.651856	1.571995	0.594188
33	8	0	-2.648101	-0.334373	0.040540
34	6	0	-0.490691	0.532466	0.392133
35	1	0	1.567651	-1.220981	2.518763
36	19	0	-5.164453	-1.082414	-0.249930

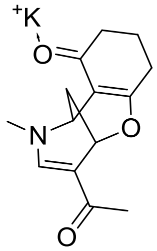

E(RB3LYP/6-31G(d,p)) = -1424.04422593 Ha

ΔG (298.15 K, 1 atm, B3LYP/6-31G(d,p)) = -1423.811279 Ha

E(RB3LYP/6-311++G(d,p)//B3LYP/6-31G(d,p)) = -1424.28063465 Ha

ΔG (298.15 K, 1 atm, B3LYP/6-311++G(d,p)//B3LYP/6-31G(d,p)) = -1424.047688 Ha

6aa-K – conformation 4

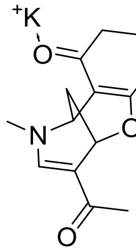

Center Number	Atomic Number	Atomic Type	X	Y	Z
1	6	0	-0.050014	-0.811456	1.025963
2	6	0	1.687458	-1.607489	-0.452388
3	6	0	2.572162	-0.647317	-0.002057
4	6	0	1.094810	-0.497943	1.991867
5	1	0	-0.892981	-1.282154	1.534083
6	1	0	2.007029	-2.279124	-1.243422
7	1	0	0.740703	0.147596	2.799458
8	7	0	0.445519	-1.775177	0.017028
9	6	0	-0.462221	-2.796634	-0.490864
10	1	0	-0.740246	-3.494630	0.306455
11	1	0	-1.372736	-2.332327	-0.878995
12	1	0	0.029913	-3.353766	-1.289113
13	6	0	3.865139	-0.535121	-0.666102
14	8	0	4.194492	-1.266097	-1.611447
15	6	0	4.836122	0.528053	-0.168783
16	1	0	4.354580	1.507951	-0.090446
17	1	0	5.216692	0.271295	0.827019
18	1	0	5.679055	0.588727	-0.858475
19	6	0	2.166550	0.203040	1.165488
20	1	0	3.017510	0.501286	1.776704
21	6	0	-1.247271	2.826082	-1.133695
22	6	0	-2.371787	1.993544	-0.513181
23	6	0	-0.054353	2.919879	-0.177821
24	1	0	-2.801873	2.526603	0.347652
25	1	0	-3.189959	1.822750	-1.219086
26	1	0	-0.921905	2.360100	-2.071142
27	1	0	-1.607642	3.828523	-1.381114
28	1	0	-0.307707	3.544295	0.690670
29	1	0	0.811438	3.386034	-0.656769
30	6	0	0.349508	1.561804	0.324510
31	6	0	-1.899420	0.642059	-0.000122
32	8	0	-2.705613	-0.299293	0.107984
33	8	0	1.624198	1.518472	0.714388
34	6	0	-0.513726	0.496331	0.403000
35	1	0	1.504972	-1.411217	2.431094
36	19	0	-5.230940	-1.009439	-0.140318

E(RB3LYP/6-31G(d,p)) = -1424.04396223 Ha

ΔG (298.15 K, 1 atm, B3LYP/6-31G(d,p)) = -1423.811618 Ha

E(RB3LYP/6-311++G(d,p)//B3LYP/6-31G(d,p)) = -1424.28045092 Ha

ΔG (298.15 K, 1 atm, B3LYP/6-311++G(d,p)//B3LYP/6-31G(d,p)) = -1424.048107 Ha

6aa-K – isomer 5

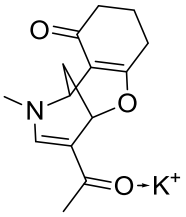

Center Number	Atomic Number	Atomic Type	Coordinates (Angstroms) X	Y	Z
1	6	0	1.395134	1.096242	1.024757
2	6	0	-0.253828	2.178211	-0.376727
3	6	0	-1.236816	1.303280	0.057042
4	6	0	0.246261	0.896930	2.013412
5	1	0	2.309209	1.431454	1.516324
6	1	0	-0.471175	2.934912	-1.123721
7	1	0	0.532434	0.181539	2.788336
8	7	0	1.005715	2.159429	0.066919
9	6	0	2.021852	3.106321	-0.382454
10	1	0	2.308124	3.779050	0.433514
11	1	0	2.907804	2.559272	-0.711986
12	1	0	1.627637	3.700773	-1.207792
13	6	0	-2.555017	1.257482	-0.526770
14	8	0	-3.386778	0.400011	-0.160393
15	6	0	-2.952781	2.243148	-1.615184
16	1	0	-2.771249	3.279546	-1.315077
17	1	0	-2.383915	2.059107	-2.532930
18	1	0	-4.014144	2.114747	-1.830829
19	6	0	-0.921184	0.372074	1.191398
20	1	0	-1.810161	0.190618	1.794232
21	6	0	2.084863	-2.574192	-1.292529
22	6	0	3.313680	-1.909087	-0.667850
23	6	0	0.897238	-2.549661	-0.323789
24	1	0	3.670401	-2.507639	0.183261
25	1	0	4.145408	-1.835407	-1.373622
26	1	0	1.809251	-2.042390	-2.210907
27	1	0	2.310802	-3.606311	-1.575199
28	1	0	1.067518	-3.245454	0.510210
29	1	0	-0.019825	-2.877009	-0.822947
30	6	0	0.679617	-1.176083	0.247324
31	6	0	3.019691	-0.513870	-0.135830
32	8	0	3.914336	0.334714	-0.066378
33	8	0	-0.577863	-0.988662	0.690926
34	6	0	1.663411	-0.231201	0.335654
35	1	0	-0.033164	1.837972	2.495095
36	19	0	-3.275540	-2.212466	0.184823

E(RB3LYP/6-31G(d,p)) = -1424.04860572 Ha

ΔG (298.15 K, 1 atm, B3LYP/6-31G(d,p)) = -1423.815305 Ha

E(RB3LYP/6-311++G(d,p)//B3LYP/6-31G(d,p)) = -1424.28380961 Ha

ΔG (298.15 K, 1 atm, B3LYP/6-311++G(d,p)//B3LYP/6-31G(d,p)) = -1424.050509 Ha

6aa-K – isomer 6

Center Number	Atomic Number	Atomic Type	Coordinates (Angstroms) X	Y	Z
1	6	0	1.512109	1.164902	1.016421
2	6	0	-0.759034	1.299874	0.205665
3	6	0	-1.080322	0.084920	0.792306
4	6	0	0.900461	0.483530	2.241696
5	1	0	2.264914	1.905240	1.290696
6	1	0	-1.515022	1.817102	-0.377177
7	1	0	1.683071	0.011416	2.840560
8	7	0	0.432217	1.889445	0.302368
9	6	0	0.733606	3.182780	-0.303008
10	1	0	0.897629	3.940414	0.471277
11	1	0	1.639568	3.099529	-0.907426
12	1	0	-0.102475	3.494318	-0.930588
13	6	0	-2.381153	-0.485960	0.528064
14	8	0	-3.221124	0.072904	-0.208647
15	6	0	-2.735044	-1.815609	1.174855
16	1	0	-1.932730	-2.549247	1.053482
17	1	0	-2.900565	-1.686432	2.251060
18	1	0	-3.652266	-2.201584	0.727280
19	6	0	-0.071180	-0.559971	1.702128
20	1	0	-0.544849	-1.121131	2.507014
21	6	0	3.105559	-1.835036	-1.769030
22	6	0	4.018806	-0.652370	-1.434622
23	6	0	2.379907	-2.333942	-0.514825
24	1	0	4.842085	-0.988033	-0.786954
25	1	0	4.476753	-0.220552	-2.328741
26	1	0	2.363255	-1.524156	-2.513750
27	1	0	3.683460	-2.650242	-2.214037
28	1	0	3.087606	-2.828819	0.165341
29	1	0	1.613727	-3.075027	-0.761313
30	6	0	1.729495	-1.199791	0.227557
31	6	0	3.295557	0.462221	-0.689789
32	8	0	3.692413	1.630260	-0.769093
33	8	0	0.706465	-1.606799	0.992136
34	6	0	2.157789	0.097510	0.150039
35	1	0	0.365666	1.200284	2.870728
36	19	0	-5.555616	0.106396	-1.344945

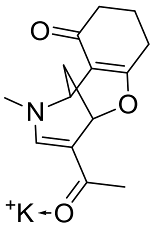

E(RB3LYP/6-31G(d,p)) = -1424.04579369 Ha

ΔG (298.15 K, 1 atm, B3LYP/6-31G(d,p)) = -1423.813818 Ha

E(RB3LYP/6-311++G(d,p)//B3LYP/6-31G(d,p)) = -1424.28180373 Ha

ΔG (298.15 K, 1 atm, B3LYP/6-311++G(d,p)//B3LYP/6-31G(d,p)) = -1424.049829 Ha

6aa-K – isomer 7

Center Number	Atomic Number	Atomic Type	X	Y	Z
1	6	0	1.378932	1.088902	1.039502
2	6	0	-0.280543	2.199055	-0.322276
3	6	0	-1.248949	1.292379	0.078353
4	6	0	0.237240	0.855413	2.029519
5	1	0	2.296534	1.408110	1.535111
6	1	0	-0.509788	2.978814	-1.041614
7	1	0	0.529504	0.120237	2.783361
8	7	0	0.978159	2.185251	0.123626
9	6	0	1.967958	3.189596	-0.255164
10	1	0	2.212643	3.829984	0.599569
11	1	0	2.877826	2.690136	-0.593523
12	1	0	1.567096	3.811390	-1.056977
13	6	0	-2.562532	1.240099	-0.515808
14	8	0	-3.383922	0.362769	-0.174099
15	6	0	-2.967374	2.243633	-1.585110
16	1	0	-2.817743	3.275653	-1.253581
17	1	0	-2.378736	2.100015	-2.497481
18	1	0	-4.021519	2.094586	-1.821808
19	6	0	-0.930491	0.343937	1.198120
20	1	0	-1.819887	0.150140	1.796581
21	6	0	2.366426	-2.887758	-0.478849
22	6	0	0.881309	-2.516310	-0.420076
23	6	0	3.191591	-1.732493	-1.052125
24	1	0	0.450375	-2.490238	-1.431427
25	1	0	0.306844	-3.259050	0.143531
26	1	0	2.718996	-3.120594	0.532848
27	1	0	2.500983	-3.790554	-1.081429
28	1	0	2.921552	-1.565946	-2.105322
29	1	0	4.264649	-1.940949	-1.030056
30	6	0	2.952424	-0.421801	-0.316286
31	6	0	0.670588	-1.169192	0.211641
32	8	0	-0.579328	-1.009464	0.686734
33	8	0	3.833036	0.442895	-0.267778
34	6	0	1.639419	-0.209040	0.292955
35	1	0	-0.043572	1.782124	2.537362
36	19	0	-3.191429	-2.254671	0.091276

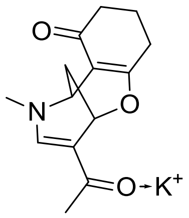

E(RB3LYP/6-31G(d,p)) = -1424.04928822 Ha

ΔG (298.15 K, 1 atm, B3LYP/6-31G(d,p)) = -1423.814817 Ha

E(RB3LYP/6-311++G(d,p)//B3LYP/6-31G(d,p)) = -1424.28443192 Ha

ΔG (298.15 K, 1 atm, B3LYP/6-311++G(d,p)//B3LYP/6-31G(d,p)) = -1424.049961 Ha

6aa-K – isomer 8

Center Number	Atomic Number	Atomic Type	X	Y	Z
1	6	0	1.493057	1.124894	1.062733
2	6	0	-0.782708	1.292416	0.277749
3	6	0	-1.081847	0.031741	0.772820
4	6	0	0.896178	0.362064	2.246654
5	1	0	2.246217	1.848508	1.377674
6	1	0	-1.549687	1.840052	-0.261332
7	1	0	1.685383	-0.141183	2.810469
8	7	0	0.400486	1.890627	0.412352
9	6	0	0.671017	3.241352	-0.070711
10	1	0	0.812543	3.929371	0.770275
11	1	0	1.580115	3.233628	-0.675314
12	1	0	-0.171449	3.587463	-0.671236
13	6	0	-2.371116	-0.542488	0.462491
14	8	0	-3.222016	0.055171	-0.229947
15	6	0	-2.699203	-1.925270	1.002640
16	1	0	-1.867180	-2.621903	0.867020
17	1	0	-2.913516	-1.874087	2.076913
18	1	0	-3.583495	-2.310329	0.492111
19	6	0	-0.068618	-0.655551	1.646817
20	1	0	-0.540084	-1.263502	2.418522
21	6	0	3.780627	-1.879998	-1.134024
22	6	0	2.361360	-2.263108	-0.704564
23	6	0	3.804207	-0.466028	-1.721591
24	1	0	1.724401	-2.430885	-1.584852
25	1	0	2.348787	-3.196437	-0.132849
26	1	0	4.444521	-1.923801	-0.262593
27	1	0	4.159092	-2.603581	-1.861916
28	1	0	3.233057	-0.442255	-2.661367
29	1	0	4.818673	-0.135135	-1.960411
30	6	0	3.180202	0.567196	-0.792673
31	6	0	1.726036	-1.187061	0.130472
32	8	0	0.724400	-1.654793	0.889117
33	8	0	3.540648	1.748959	-0.836214
34	6	0	2.126066	0.121202	0.114103
35	1	0	0.359913	1.034159	2.921970
36	19	0	-5.586658	0.111193	-1.310037

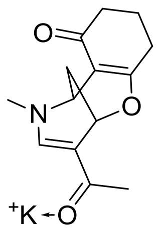

E(RB3LYP/6-31G(d,p)) = -1424.04613951 Ha

ΔG (298.15 K, 1 atm, B3LYP/6-31G(d,p)) = -1423.813133 Ha

E(RB3LYP/6-311++G(d,p)//B3LYP/6-31G(d,p)) = -1424.28211114 Ha

ΔG (298.15 K, 1 atm, B3LYP/6-311++G(d,p)//B3LYP/6-31G(d,p)) = -1424.049105 Ha

6a-KI – conformation 1

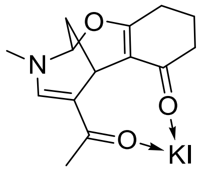

Center Number	Atomic Number	Atomic Type	Coordinates (Angstroms) X	Y	Z
1	6	0	3.574740	-0.368908	-1.756789
2	6	0	3.151909	-1.967252	0.031293
3	6	0	1.857698	-1.551102	0.197516
4	6	0	2.075366	-0.453893	-1.997646
5	1	0	4.158831	-0.434355	-2.675608
6	1	0	3.560011	-2.804584	0.586563
7	1	0	1.777121	0.361989	-2.661171
8	7	0	4.020653	-1.407036	-0.853396
9	6	0	5.447691	-1.720058	-0.831481
10	1	0	6.026407	-0.878582	-0.435657
11	1	0	5.618806	-2.591631	-0.198441
12	1	0	5.799956	-1.944908	-1.842452
13	6	0	0.932588	-2.286664	1.039451
14	8	0	-0.272910	-1.987690	1.095689
15	6	0	1.424304	-3.472677	1.861763
16	1	0	1.678498	-4.317875	1.213258
17	1	0	2.312082	-3.226485	2.451641
18	1	0	0.622563	-3.780569	2.534410
19	6	0	1.410890	-0.342845	-0.617889
20	1	0	0.323031	-0.346632	-0.696134
21	6	0	2.460013	3.749882	0.639685
22	6	0	1.530679	2.970151	1.573750
23	6	0	3.585517	2.846854	0.129875
24	1	0	2.074278	2.680742	2.485485
25	1	0	0.670941	3.566617	1.890838
26	1	0	1.884629	4.127221	-0.214051
27	1	0	2.879285	4.619409	1.154258
28	1	0	4.306141	2.639396	0.934273
29	1	0	4.152632	3.322234	-0.676704
30	6	0	3.057487	1.532486	-0.370753
31	6	0	1.004618	1.691029	0.939092
32	8	0	-0.114240	1.263264	1.253580
33	8	0	3.923188	0.947478	-1.228783
34	6	0	1.859390	0.984554	-0.010446
35	1	0	1.838219	-1.403382	-2.485249
36	19	0	-2.228751	-0.285488	1.488740
37	53	0	-4.981271	0.079833	-0.709547

E(RB3LYP/6-31G(d,p):LANL2DZ) = -1435.61625085 Ha

ΔG (298.15 K, 1 atm, B3LYP/6-31G(d,p):LANL2DZ) = -1435.391367 Ha

6a-KI – conformation 2

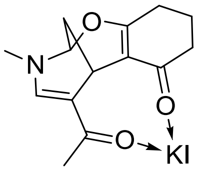

Center Number	Atomic Number	Atomic Type	Coordinates (Angstroms) X	Y	Z
1	6	0	3.613645	-0.542661	-1.742997
2	6	0	3.126586	-2.004153	0.137517
3	6	0	1.853143	-1.520058	0.276126
4	6	0	2.115056	-0.591237	-1.994083
5	1	0	4.203692	-0.667744	-2.651565
6	1	0	3.501829	-2.811396	0.756706
7	1	0	1.847646	0.188122	-2.712305
8	7	0	4.014209	-1.552993	-0.790399
9	6	0	5.427642	-1.920294	-0.741596
10	1	0	6.037939	-1.084116	-0.383434
11	1	0	5.559959	-2.765880	-0.065413
12	1	0	5.775471	-2.209616	-1.737470
13	6	0	0.912209	-2.126614	1.199543
14	8	0	-0.270080	-1.747236	1.261022
15	6	0	1.356344	-3.268105	2.106995
16	1	0	1.568564	-4.170870	1.524362
17	1	0	2.258869	-3.017166	2.672251
18	1	0	0.547579	-3.486919	2.805566
19	6	0	1.441477	-0.366843	-0.632162
20	1	0	0.354609	-0.353443	-0.722066
21	6	0	3.017099	3.320630	1.187638
22	6	0	3.691242	2.805614	-0.086824
23	6	0	1.495696	3.234947	1.056134
24	1	0	3.531198	3.507416	-0.917957
25	1	0	4.774665	2.713846	0.038028
26	1	0	3.346613	2.714216	2.039737
27	1	0	3.327148	4.350529	1.386799
28	1	0	1.144411	3.947378	0.294935
29	1	0	0.985940	3.496445	1.987924
30	6	0	1.004381	1.858864	0.626401
31	6	0	3.141451	1.465035	-0.483233
32	8	0	4.008075	0.782062	-1.262598
33	8	0	-0.160482	1.522305	0.880557
34	6	0	1.907480	0.997378	-0.129286
35	1	0	1.852520	-1.562840	-2.421448
36	19	0	-2.229854	-0.015285	1.416939
37	53	0	-5.086636	-0.002616	-0.660313

E(RB3LYP/6-31G(d,p):LANL2DZ) = -1435.61630369 Ha

ΔG (298.15 K, 1 atm, B3LYP/6-31G(d,p):LANL2DZ) = -1435.391310 Ha

6a-KI – conformation 3

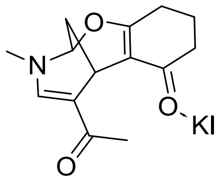

Center Number	Atomic Number	Atomic Type	X	Y	Z
1	6	0	4.288598	-0.558159	1.392555
2	6	0	4.192014	1.388210	-0.058744
3	6	0	2.845460	1.547046	0.102392
4	6	0	3.052799	0.042295	2.044930
5	1	0	5.025118	-0.909311	2.116450
6	1	0	4.753260	2.113780	-0.638461
7	1	0	2.591107	-0.710699	2.688931
8	7	0	4.922042	0.382139	0.502318
9	6	0	6.304641	0.127606	0.107326
10	1	0	6.376152	-0.750254	-0.544584
11	1	0	6.692161	0.994553	-0.429482
12	1	0	6.924351	-0.041840	0.992906
13	6	0	2.237010	2.762662	-0.450481
14	8	0	2.873246	3.547811	-1.166974
15	6	0	0.793428	3.071649	-0.094621
16	1	0	0.709599	3.296950	0.976048
17	1	0	0.462161	3.941352	-0.664790
18	1	0	0.149722	2.211368	-0.294983
19	6	0	2.118073	0.467355	0.901041
20	1	0	1.175573	0.845595	1.299926
21	6	0	1.046263	-3.399083	-0.924160
22	6	0	0.345985	-2.164802	-1.497151
23	6	0	2.529830	-3.108469	-0.689861
24	1	0	0.727175	-1.952559	-2.507196
25	1	0	-0.733300	-2.315524	-1.590161
26	1	0	0.576972	-3.675024	0.027551
27	1	0	0.931300	-4.252047	-1.599289
28	1	0	3.065169	-3.042162	-1.648099
29	1	0	3.014888	-3.907143	-0.120066
30	6	0	2.728824	-1.813468	0.045291
31	6	0	0.576619	-0.914320	-0.661342
32	8	0	-0.283386	-0.017315	-0.647521
33	8	0	3.921734	-1.782262	0.673195
34	6	0	1.823860	-0.788206	0.078255
35	1	0	3.345290	0.896130	2.662033
36	19	0	-2.719848	0.704571	-1.470455
37	53	0	-5.470888	-0.051939	0.585915

E(RB3LYP/6-31G(d,p):LANL2DZ) = -1435.60670776 Ha

ΔG (298.15 K, 1 atm, B3LYP/6-31G(d,p):LANL2DZ) = -1435.382360 Ha

6a-KI – conformation 4

Center Number	Atomic Number	Atomic Type	X	Y	Z
1	6	0	4.650449	-0.782398	0.724706
2	6	0	4.255262	1.062312	-0.799220
3	6	0	3.100953	1.438772	-0.174981
4	6	0	3.855643	0.048700	1.719800
5	1	0	5.548194	-1.224457	1.158460
6	1	0	4.629866	1.647208	-1.632844
7	1	0	3.586430	-0.577901	2.574106
8	7	0	5.024056	-0.000714	-0.426210
9	6	0	6.099325	-0.501980	-1.277773
10	1	0	5.795260	-1.411732	-1.807729
11	1	0	6.361569	0.260208	-2.012828
12	1	0	6.983629	-0.725920	-0.673895
13	6	0	2.458605	2.677110	-0.631461
14	8	0	2.865540	3.302870	-1.619871
15	6	0	1.279264	3.213426	0.161621
16	1	0	0.525115	2.438922	0.325230
17	1	0	1.614432	3.549152	1.151246
18	1	0	0.848184	4.063074	-0.370710
19	6	0	2.612057	0.556678	0.971354
20	1	0	1.964201	1.119434	1.645068
21	6	0	0.517976	-2.869287	-0.847335
22	6	0	1.885970	-3.121945	-0.209482
23	6	0	-0.326346	-1.961506	0.048327
24	1	0	1.784823	-3.766099	0.675891
25	1	0	2.562990	-3.640177	-0.895491
26	1	0	0.658220	-2.391982	-1.824337
27	1	0	0.003408	-3.818335	-1.023049
28	1	0	-0.591328	-2.490218	0.976072
29	1	0	-1.268137	-1.676988	-0.430194
30	6	0	0.394733	-0.686133	0.461045
31	6	0	2.531015	-1.832764	0.213931
32	8	0	3.869681	-1.955028	0.309549
33	8	0	-0.268848	0.307788	0.803532
34	6	0	1.848940	-0.681786	0.500152
35	1	0	4.477101	0.874789	2.076003
36	19	0	-2.710860	1.300640	1.158163
37	53	0	-5.506794	-0.179931	-0.378159

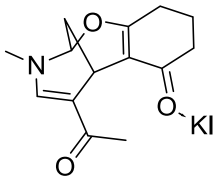

E(RB3LYP/6-31G(d,p):LANL2DZ) = -1435.60661316 Ha

ΔG (298.15 K, 1 atm, B3LYP/6-31G(d,p):LANL2DZ) = -1435.382155 Ha

6a-KI – conformation 5

Center Number	Atomic Number	Atomic Type	Coordinates (Angstroms) X	Y	Z
1	6	0	1.779562	-1.963108	-1.405343
2	6	0	0.113218	-0.329093	-0.724826
3	6	0	0.955349	0.733518	-0.928278
4	6	0	2.354107	-0.824727	-2.233944
5	1	0	1.712303	-2.900461	-1.958951
6	1	0	-0.916769	-0.159924	-0.425977
7	1	0	3.352409	-1.104948	-2.579939
8	7	0	0.463838	-1.628130	-0.904958
9	6	0	-0.404113	-2.724018	-0.478369
10	1	0	0.010352	-3.227022	0.402221
11	1	0	-1.390642	-2.328664	-0.229331
12	1	0	-0.509097	-3.456888	-1.284253
13	6	0	0.405144	2.074820	-0.801568
14	8	0	-0.761171	2.279550	-0.408876
15	6	0	1.267554	3.259459	-1.188432
16	1	0	2.253163	3.205118	-0.717559
17	1	0	1.428232	3.262401	-2.274020
18	1	0	0.756694	4.182302	-0.907950
19	6	0	2.395993	0.405631	-1.313458
20	1	0	2.863308	1.249471	-1.823059
21	6	0	4.522434	-0.628346	2.405897
22	6	0	4.208055	-1.758457	1.421700
23	6	0	5.029809	0.605334	1.656514
24	1	0	5.136370	-2.169204	0.999160
25	1	0	3.696279	-2.591278	1.914284
26	1	0	3.612624	-0.371195	2.961506
27	1	0	5.262368	-0.963871	3.138612
28	1	0	6.006190	0.388123	1.198547
29	1	0	5.178279	1.460635	2.321662
30	6	0	4.100492	1.044915	0.531051
31	6	0	3.346674	-1.269386	0.291372
32	8	0	2.672067	-2.285759	-0.295627
33	8	0	4.101955	2.223776	0.159755
34	6	0	3.269432	0.029740	-0.117222
35	1	0	1.717677	-0.655281	-3.106848
36	19	0	-3.100279	2.520538	0.663723
37	53	0	-4.390120	-0.754544	0.335156

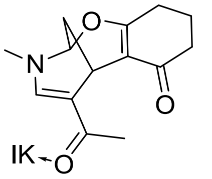

E(RB3LYP/6-31G(d,p):LANL2DZ) = -1435.60833633 Ha

ΔG (298.15 K, 1 atm, B3LYP/6-31G(d,p):LANL2DZ) = -1435.382365 Ha

6a-KI – conformation 6

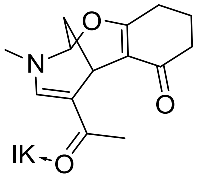

Center Number	Atomic Number	Atomic Type	X	Y	Z
1	6	0	3.037405	2.274299	0.710260
2	6	0	1.029725	1.227625	-0.182628
3	6	0	1.239244	0.055568	0.495364
4	6	0	2.843932	1.316998	1.876911
5	1	0	3.246005	3.296118	1.029966
6	1	0	0.127314	1.356803	-0.770997
7	1	0	3.754949	1.309070	2.480900
8	7	0	1.873473	2.290421	-0.151828
9	6	0	1.699851	3.440295	-1.036205
10	1	0	2.510856	3.490178	-1.770338
11	1	0	0.751725	3.348554	-1.567126
12	1	0	1.691225	4.368889	-0.456978
13	6	0	0.182554	-0.945296	0.465285
14	8	0	-0.833435	-0.810136	-0.246359
15	6	0	0.309139	-2.169127	1.349721
16	1	0	0.229336	-1.876020	2.404125
17	1	0	-0.497513	-2.867270	1.118411
18	1	0	1.283042	-2.648739	1.218757
19	6	0	2.553599	-0.061699	1.263165
20	1	0	2.481594	-0.821711	2.042602
21	6	0	6.317539	-1.017498	-0.813319
22	6	0	5.234532	-2.098781	-0.848886
23	6	0	5.701521	0.362521	-1.056816
24	1	0	4.808896	-2.166576	-1.861143
25	1	0	5.630204	-3.088201	-0.603955
26	1	0	6.808895	-1.025506	0.166901
27	1	0	7.089412	-1.221536	-1.561396
28	1	0	5.401348	0.469353	-2.109317
29	1	0	6.417635	1.166411	-0.858015
30	6	0	4.488505	0.584803	-0.198379
31	6	0	4.082546	-1.806795	0.104792
32	8	0	3.452037	-2.739133	0.614600
33	8	0	4.227218	1.905396	-0.046312
34	6	0	3.738464	-0.407208	0.361087
35	1	0	2.015108	1.662869	2.500591
36	19	0	-3.147912	-1.269240	-1.329736
37	53	0	-5.973055	0.225409	0.139759

E(RB3LYP/6-31G(d,p):LANL2DZ) = -1435.60820060 Ha

ΔG (298.15 K, 1 atm, B3LYP/6-31G(d,p):LANL2DZ) = -1435.385112 Ha

6aa-KI – conformation 1

Center Number	Atomic Number	Atomic Type	Coordinates (Angstroms) X	Y	Z
1	6	0	-1.491850	-0.674691	-0.582818
2	6	0	-3.404496	-1.646886	0.541817
3	6	0	-4.279448	-0.967306	-0.281774
4	6	0	-2.354816	-0.752847	-1.845520
5	1	0	-0.468746	-1.006148	-0.767315
6	1	0	-3.771420	-2.286568	1.338360
7	1	0	-1.910331	-0.154459	-2.645036
8	7	0	-2.068486	-1.587799	0.431022
9	6	0	-1.170168	-2.360146	1.281688
10	1	0	-0.624648	-3.106655	0.692638
11	1	0	-0.445975	-1.694624	1.757661
12	1	0	-1.749575	-2.876309	2.048938
13	6	0	-5.721674	-1.008583	-0.107538
14	8	0	-6.479304	-0.420465	-0.892955
15	6	0	-6.318211	-1.787008	1.061490
16	1	0	-6.052481	-2.848475	1.013514
17	1	0	-5.962353	-1.401788	2.022724
18	1	0	-7.404401	-1.693953	1.022131
19	6	0	-3.721735	-0.208903	-1.451353
20	1	0	-4.438394	-0.202871	-2.271239
21	6	0	-1.113294	3.620951	0.154633
22	6	0	-2.520791	3.079091	-0.108263
23	6	0	-0.357416	2.707481	1.123945
24	1	0	-3.152089	3.200513	0.783700
25	1	0	-3.021347	3.620277	-0.917044
26	1	0	-0.565316	3.679210	-0.793340
27	1	0	-1.171429	4.637201	0.555038
28	1	0	-0.830792	2.740134	2.116495
29	1	0	0.681383	3.022923	1.258022
30	6	0	-0.355775	1.252997	0.679902
31	6	0	-2.495035	1.617475	-0.459795
32	8	0	-3.574654	1.238610	-1.143214
33	8	0	0.574175	0.499109	1.017921
34	6	0	-1.472676	0.770072	-0.109309
35	1	0	-2.450777	-1.784339	-2.195584
36	19	0	3.059586	0.057112	1.765856
37	53	0	5.588463	-0.456657	-0.619335

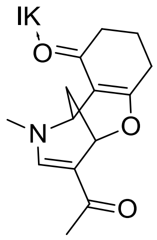

E(RB3LYP/6-31G(d,p):LANL2DZ) = -1435.60976062 Ha

ΔG (298.15 K, 1 atm, B3LYP/6-31G(d,p):LANL2DZ) = -1435.387573 Ha

6aa-KI – conformation 2

Center Number	Atomic Number	Atomic Type	X	Y	Z
1	6	0	-1.345060	-0.699325	-0.313397
2	6	0	-3.305873	-1.525344	0.846939
3	6	0	-4.133715	-1.111781	-0.177334
4	6	0	-2.088092	-1.101891	-1.590304
5	1	0	-0.296672	-1.000981	-0.340141
6	1	0	-3.712488	-1.993146	1.738032
7	1	0	-1.599395	-0.662715	-2.463926
8	7	0	-1.969559	-1.400662	0.830929
9	6	0	-1.122776	-1.833195	1.936187
10	1	0	-0.422437	-2.607711	1.603981
11	1	0	-0.546734	-0.988211	2.323481
12	1	0	-1.744776	-2.242876	2.733618
13	6	0	-5.581403	-1.224751	-0.117514
14	8	0	-6.291357	-0.876914	-1.072283
15	6	0	-6.243769	-1.779300	1.140226
16	1	0	-5.921582	-2.806212	1.343499
17	1	0	-5.999062	-1.176685	2.021083
18	1	0	-7.324842	-1.771998	0.994544
19	6	0	-3.507755	-0.576556	-1.432647
20	1	0	-4.145904	-0.784497	-2.290118
21	6	0	-1.806205	3.610897	0.409724
22	6	0	-0.384541	3.048925	0.494193
23	6	0	-2.560003	2.997379	-0.774487
24	1	0	0.206892	3.393565	-0.367106
25	1	0	0.138126	3.390618	1.392327
26	1	0	-2.345335	3.383356	1.337011
27	1	0	-1.780101	4.700016	0.312422
28	1	0	-2.143357	3.363416	-1.723701
29	1	0	-3.617929	3.275623	-0.768979
30	6	0	-2.458373	1.496955	-0.766797
31	6	0	-0.342849	1.528850	0.473708
32	8	0	0.624571	0.927650	0.973608
33	8	0	-3.462362	0.911518	-1.418070
34	6	0	-1.422365	0.812964	-0.178634
35	1	0	-2.106454	-2.188404	-1.712423
36	19	0	3.124734	0.613787	1.708871
37	53	0	5.445828	-0.654745	-0.603469

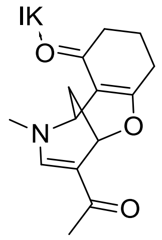

E(RB3LYP/6-31G(d,p):LANL2DZ) = -1435.60957662 Ha

ΔG (298.15 K, 1 atm, B3LYP/6-31G(d,p):LANL2DZ) = -1435.386392 Ha

6aa-KI – conformation 3

Center Number	Atomic Number	Atomic Type	Coordinates (Angstroms) X	Y	Z
1	6	0	-1.512542	-0.724831	-0.631821
2	6	0	-3.450903	-1.687802	0.437420
3	6	0	-4.303503	-0.891588	-0.303141
4	6	0	-2.393185	-0.657102	-1.881644
5	1	0	-0.507442	-1.082282	-0.861185
6	1	0	-3.875910	-2.354632	1.181982
7	1	0	-1.931104	-0.017175	-2.637603
8	7	0	-2.119038	-1.696626	0.307587
9	6	0	-1.252557	-2.595748	1.060977
10	1	0	-0.769037	-3.318023	0.392931
11	1	0	-0.478112	-2.019123	1.572155
12	1	0	-1.847451	-3.141829	1.794839
13	6	0	-5.725945	-0.923117	0.014810
14	8	0	-6.190570	-1.622068	0.927168
15	6	0	-6.664287	-0.055427	-0.814826
16	1	0	-6.303390	0.975827	-0.883363
17	1	0	-6.746017	-0.440462	-1.838428
18	1	0	-7.655179	-0.067319	-0.358403
19	6	0	-3.728222	-0.077860	-1.425988
20	1	0	-4.424816	0.029714	-2.256962
21	6	0	-0.920777	3.465426	0.483511
22	6	0	-2.352326	3.023333	0.169017
23	6	0	-0.220938	2.431844	1.370622
24	1	0	-2.985067	3.100549	1.064873
25	1	0	-2.816393	3.658196	-0.592104
26	1	0	-0.363070	3.577826	-0.453815
27	1	0	-0.929407	4.444136	0.971793
28	1	0	-0.699937	2.400841	2.360568
29	1	0	0.831633	2.679132	1.536002
30	6	0	-0.290800	1.024901	0.798170
31	6	0	-2.401567	1.598441	-0.307123
32	8	0	-3.500147	1.336977	-1.018569
33	8	0	0.595266	0.196101	1.070581
34	6	0	-1.425631	0.671877	-0.035277
35	1	0	-2.545568	-1.650570	-2.312071
36	19	0	3.044335	-0.469016	1.793120
37	53	0	5.540752	-0.243194	-0.685760

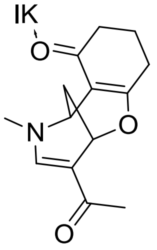

E(RB3LYP/6-31G(d,p):LANL2DZ) = -1435.60894670 Ha

ΔG (298.15 K, 1 atm, B3LYP/6-31G(d,p):LANL2DZ) = -1435.384897 Ha

6aa-KI – conformation 4

Center Number	Atomic Number	Atomic Type	Coordinates (Angstroms) X	Y	Z
1	6	0	-1.254149	-0.607785	-0.375577
2	6	0	-2.995587	-1.610470	0.965874
3	6	0	-3.955534	-1.320659	0.015899
4	6	0	-2.051517	-1.132311	-1.572073
5	1	0	-0.183728	-0.784802	-0.492166
6	1	0	-3.307300	-2.085445	1.891430
7	1	0	-1.695910	-0.667104	-2.494951
8	7	0	-1.690389	-1.345580	0.831742
9	6	0	-0.709340	-1.653183	1.865486
10	1	0	0.052562	-2.339128	1.479059
11	1	0	-0.213901	-0.737952	2.201440
12	1	0	-1.210404	-2.125842	2.711478
13	6	0	-5.349796	-1.599583	0.337962
14	8	0	-5.699884	-2.081659	1.425066
15	6	0	-6.405073	-1.277479	-0.712852
16	1	0	-6.296204	-0.256326	-1.092330
17	1	0	-6.317898	-1.954702	-1.570957
18	1	0	-7.395200	-1.400022	-0.271361
19	6	0	-3.507047	-0.766259	-1.304352
20	1	0	-4.156956	-1.070484	-2.124256
21	6	0	-2.164358	3.637810	0.308428
22	6	0	-0.684051	3.248287	0.289374
23	6	0	-2.928384	2.911714	-0.803163
24	1	0	-0.203760	3.640206	-0.619488
25	1	0	-0.138984	3.668732	1.139500
26	1	0	-2.600049	3.370704	1.278386
27	1	0	-2.275282	4.719806	0.193278
28	1	0	-2.631250	3.299088	-1.788193
29	1	0	-4.008124	3.065441	-0.718901
30	6	0	-2.651561	1.433999	-0.777755
31	6	0	-0.464294	1.743234	0.286734
32	8	0	0.604187	1.271745	0.713102
33	8	0	-3.629967	0.719777	-1.336179
34	6	0	-1.500683	0.888989	-0.263340
35	1	0	-1.954102	-2.216999	-1.669456
36	19	0	3.131332	1.125613	1.404716
37	53	0	5.403914	-0.768858	-0.500832

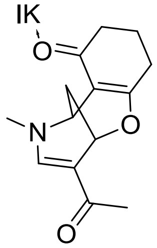

E(RB3LYP/6-31G(d,p):LANL2DZ) = -1435.60871710 Ha

ΔG (298.15 K, 1 atm, B3LYP/6-31G(d,p):LANL2DZ) = -1435.385550 Ha

6aa-KI – isomer 5

Center Number	Atomic Number	Atomic Type	X	Y	Z
1	6	0	-2.952361	-0.025004	-1.417078
2	6	0	-2.816259	2.061154	-0.199087
3	6	0	-1.440342	1.939568	-0.090662
4	6	0	-1.609850	0.383023	-2.025397
5	1	0	-3.639609	-0.423738	-2.164490
6	1	0	-3.340074	2.896063	0.255827
7	1	0	-1.149591	-0.467202	-2.535077
8	7	0	-3.591033	1.185293	-0.845827
9	6	0	-5.035963	1.345955	-0.977917
10	1	0	-5.306785	1.559072	-2.018207
11	1	0	-5.532958	0.423646	-0.668902
12	1	0	-5.370644	2.173361	-0.350265
13	6	0	-0.639099	2.825310	0.720049
14	8	0	0.587948	2.635252	0.853396
15	6	0	-1.280841	4.000740	1.443100
16	1	0	-1.843187	4.642199	0.757475
17	1	0	-1.974903	3.652810	2.215784
18	1	0	-0.494622	4.589206	1.917802
19	6	0	-0.751173	0.848520	-0.858836
20	1	0	0.241875	1.170236	-1.170926
21	6	0	-2.343541	-2.872021	1.876839
22	6	0	-3.403486	-3.235624	0.833407
23	6	0	-1.101923	-2.272400	1.207230
24	1	0	-3.035768	-4.055111	0.198253
25	1	0	-4.332021	-3.584150	1.293781
26	1	0	-2.759633	-2.141577	2.581049
27	1	0	-2.061252	-3.754041	2.458964
28	1	0	-0.550991	-3.047814	0.656006
29	1	0	-0.411078	-1.868113	1.953447
30	6	0	-1.470396	-1.181071	0.240396
31	6	0	-3.740230	-2.074882	-0.092135
32	8	0	-4.852214	-1.996454	-0.624539
33	8	0	-0.459678	-0.322522	0.013550
34	6	0	-2.692465	-1.091233	-0.366209
35	1	0	-1.729295	1.196988	-2.745871
36	19	0	2.045742	0.485359	1.422123
37	53	0	4.811657	-0.507984	-0.51765

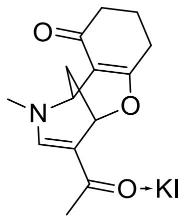

E(RB3LYP/6-31G(d,p):LANL2DZ) = -1435.61319384 Ha

ΔG (298.15 K, 1 atm, B3LYP/6-31G(d,p):LANL2DZ) = -1435.388504 Ha

6aa-KI – isomer 6

Center Number	Atomic Number	Atomic Type	Coordinates (Angstroms) X	Y	Z
1	6	0	3.318644	1.179129	1.144852
2	6	0	1.099561	0.822191	0.262390
3	6	0	1.085231	-0.484791	0.725691
4	6	0	2.878823	0.254323	2.281612
5	1	0	3.858901	2.053038	1.512012
6	1	0	0.246621	1.188226	-0.301322
7	1	0	3.745617	-0.063367	2.866526
8	7	0	2.102826	1.678261	0.457285
9	6	0	2.076873	3.059281	-0.014196
10	1	0	2.018715	3.754000	0.831402
11	1	0	2.990149	3.268231	-0.575927
12	1	0	1.206106	3.209019	-0.654279
13	6	0	-0.027755	-1.332807	0.360725
14	8	0	-0.967731	-0.934674	-0.358163
15	6	0	-0.049347	-2.763980	0.873337
16	1	0	0.906046	-3.267999	0.698166
17	1	0	-0.235443	-2.783012	1.953842
18	1	0	-0.849048	-3.312224	0.372912
19	6	0	2.208420	-0.942155	1.615392
20	1	0	1.876347	-1.676282	2.349072
21	6	0	5.650392	-1.056561	-1.832793
22	6	0	6.231368	0.281657	-1.366321
23	6	0	5.060280	-1.834580	-0.651365
24	1	0	7.104896	0.104694	-0.721436
25	1	0	6.576446	0.894880	-2.203451
26	1	0	4.862015	-0.874318	-2.572828
27	1	0	6.420716	-1.656367	-2.326423
28	1	0	5.863060	-2.194226	0.007988
29	1	0	4.511044	-2.719633	-0.986202
30	6	0	4.134062	-0.973022	0.161817
31	6	0	5.240517	1.106149	-0.553886
32	8	0	5.328344	2.338895	-0.520910
33	8	0	3.235685	-1.691342	0.849387
34	6	0	4.222976	0.391743	0.212246
35	1	0	2.172005	0.753686	2.949990
36	19	0	-3.209506	-0.571290	-1.599369
37	53	0	-6.161585	0.348789	0.092594

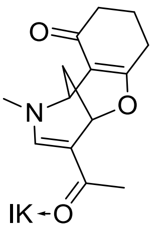

E(RB3LYP/6-31G(d,p):LANL2DZ) = -1435.61017151 Ha

ΔG (298.15 K, 1 atm, B3LYP/6-31G(d,p):LANL2DZ) = -1435.383663 Ha

6aa-KI – isomer 7

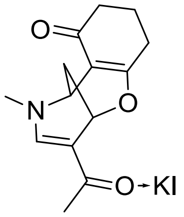

Center Number	Atomic Number	Atomic Type	Coordinates (Angstroms) X	Y	Z
1	6	0	3.116705	-0.031727	1.349739
2	6	0	2.934341	2.030762	0.102228
3	6	0	1.552072	1.934304	0.098201
4	6	0	1.840132	0.424103	2.057769
5	1	0	3.851261	-0.439338	2.045706
6	1	0	3.438490	2.845149	-0.408762
7	1	0	1.400887	-0.402286	2.622093
8	7	0	3.738325	1.153466	0.709933
9	6	0	5.190060	1.303315	0.754338
10	1	0	5.523053	1.536751	1.772046
11	1	0	5.659063	0.370137	0.435824
12	1	0	5.492706	2.114962	0.090736
13	6	0	0.710991	2.816263	-0.675696
14	8	0	-0.526307	2.650542	-0.711678
15	6	0	1.320621	3.956317	-1.478828
16	1	0	1.949351	4.601639	-0.857502
17	1	0	1.943818	3.571094	-2.293056
18	1	0	0.512964	4.551228	-1.907348
19	6	0	0.898733	0.887172	0.954445
20	1	0	-0.054126	1.249406	1.339009
21	6	0	1.895635	-3.507044	-1.001773
22	6	0	0.994832	-2.269732	-1.071722
23	6	0	3.370296	-3.110542	-1.116870
24	1	0	0.975464	-1.864270	-2.093829
25	1	0	-0.040802	-2.516756	-0.815083
26	1	0	1.729126	-4.019453	-0.046815
27	1	0	1.624591	-4.210038	-1.794772
28	1	0	3.569418	-2.696602	-2.116465
29	1	0	4.039012	-3.966364	-0.990210
30	6	0	3.770855	-2.046500	-0.104170
31	6	0	1.468824	-1.183680	-0.146954
32	8	0	0.497673	-0.302736	0.155620
33	8	0	4.934534	-1.972600	0.304290
34	6	0	2.745722	-1.099096	0.333141
35	1	0	2.041501	1.248675	2.747128
36	19	0	-1.986087	0.492289	-1.246500
37	53	0	-4.947066	-0.434468	0.427855

E(RB3LYP/6-31G(d,p):LANL2DZ) = -1435.61382599 Ha

ΔG (298.15 K, 1 atm, B3LYP/6-31G(d,p):LANL2DZ) = -1435.387419 Ha

6aa-KI – isomer 8

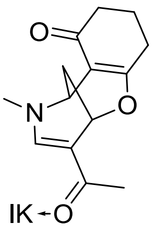

Center Number	Atomic Number	Atomic Type	Coordinates (Angstroms) X	Y	Z
1	6	0	3.199783	1.191052	1.141601
2	6	0	0.992515	0.713161	0.291317
3	6	0	1.081213	-0.606920	0.706863
4	6	0	2.844668	0.210197	2.260770
5	1	0	3.693538	2.086608	1.521768
6	1	0	0.103955	1.036925	-0.242287
7	1	0	3.741194	-0.065731	2.821573
8	7	0	1.936971	1.631105	0.498060
9	6	0	1.793840	3.027488	0.097180
10	1	0	1.718860	3.674680	0.978442
11	1	0	2.666675	3.328322	-0.486186
12	1	0	0.888838	3.142324	-0.501502
13	6	0	0.027336	-1.521609	0.327484
14	8	0	-0.952188	-1.169628	-0.362045
15	6	0	0.122177	-2.967690	0.787778
16	1	0	1.115435	-3.385371	0.597885
17	1	0	-0.062478	-3.039928	1.866340
18	1	0	-0.630442	-3.561096	0.266193
19	6	0	2.242808	-1.011111	1.573002
20	1	0	1.965821	-1.780772	2.293089
21	6	0	6.323793	-0.837741	-1.140223
22	6	0	5.081253	-1.658609	-0.781242
23	6	0	5.925368	0.550868	-1.648951
24	1	0	4.539038	-1.955168	-1.690621
25	1	0	5.343908	-2.586452	-0.263055
26	1	0	6.955381	-0.732778	-0.250107
27	1	0	6.916124	-1.365889	-1.893305
28	1	0	5.387917	0.458388	-2.604249
29	1	0	6.794803	1.187102	-1.836567
30	6	0	5.003456	1.287389	-0.685573
31	6	0	4.136338	-0.879458	0.090614
32	8	0	3.311757	-1.674082	0.787338
33	8	0	4.987186	2.523277	-0.652517
34	6	0	4.122394	0.487135	0.160746
35	1	0	2.119980	0.645096	2.954545
36	19	0	-3.285871	-1.046456	-1.479870
37	53	0	-6.047953	0.491268	0.072240

E(RB3LYP/6-31G(d,p):LANL2DZ) = -1435.61056053 Ha

ΔG (298.15 K, 1 atm, B3LYP/6-31G(d,p):LANL2DZ) = -1435.387155 Ha

6a-KHCO$_3$ – conformation 1

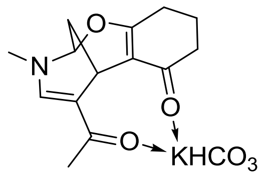

Center Number	Atomic Number	Atomic Type	X	Y	Z
1	6	0	-2.369683	-0.991250	1.614990
2	6	0	-1.793520	-2.163579	-0.442176
3	6	0	-0.621091	-1.474070	-0.597565
4	6	0	-0.868646	-0.802252	1.759935
5	1	0	-2.851126	-1.323014	2.535685
6	1	0	-2.078127	-2.967950	-1.111352
7	1	0	-0.679913	-0.056631	2.536609
8	7	0	-2.679683	-1.937846	0.566369
9	6	0	-4.015836	-2.528023	0.559257
10	1	0	-4.777220	-1.771204	0.342395
11	1	0	-4.065875	-3.304157	-0.205227
12	1	0	-4.234480	-2.978542	1.531812
13	6	0	0.354284	-1.865697	-1.599353
14	8	0	1.473933	-1.331601	-1.663315
15	6	0	0.025713	-2.977638	-2.589963
16	1	0	-0.007924	-3.950043	-2.087261
17	1	0	-0.939553	-2.822474	-3.080441
18	1	0	0.810674	-3.005840	-3.346791
19	6	0	-0.354175	-0.341690	0.388415
20	1	0	0.714861	-0.130094	0.432003
21	6	0	-2.298764	3.580458	-0.138032
22	6	0	-1.342091	3.144309	-1.249969
23	6	0	-3.170352	2.404043	0.305430
24	1	0	-1.913970	2.874438	-2.150248
25	1	0	-0.652915	3.942956	-1.537247
26	1	0	-1.719266	3.944671	0.718659
27	1	0	-2.928202	4.408870	-0.475799
28	1	0	-3.921534	2.170803	-0.463055
29	1	0	-3.727065	2.634079	1.219477
30	6	0	-2.352233	1.167712	0.547103
31	6	0	-0.512018	1.928707	-0.861749
32	8	0	0.625915	1.789556	-1.327690
33	8	0	-3.008377	0.294628	1.346377
34	6	0	-1.109068	0.938186	0.031156
35	1	0	-0.409005	-1.746149	2.065332
36	19	0	3.084951	0.737723	-1.288310
37	6	0	3.966244	-0.053717	1.491044
38	8	0	4.632469	-0.546859	0.552782
39	8	0	4.316394	-0.511338	2.763329
40	1	0	3.721987	-0.044238	3.368937
41	8	0	3.030887	0.790580	1.442103

E(RB3LYP/6-31G(d,p)) = -1688.61527173 Ha

ΔG (298.15 K, 1 atm, B3LYP/6-31G(d,p)) = -1688.364669 Ha

E(RB3LYP/6-311++G(d,p)//B3LYP/6-31G(d,p)) = -1688.95122507 Ha

ΔG (298.15 K, 1 atm, B3LYP/6-311++G(d,p)//B3LYP/6-31G(d,p)) = -1688.700622 Ha

6a-$KHCO_3$ – conformation 2

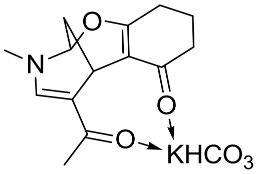

Center Number	Atomic Number	Atomic Type	Coordinates (Angstroms) X	Y	Z
1	6	0	2.265612	-1.268897	-1.516556
2	6	0	1.662246	-2.143585	0.670123
3	6	0	0.554582	-1.345281	0.766007
4	6	0	0.778499	-0.996840	-1.666280
5	1	0	2.711364	-1.719294	-2.403963
6	1	0	1.910152	-2.871290	1.434692
7	1	0	0.619904	-0.339336	-2.524856
8	7	0	2.523958	-2.128436	-0.384300
9	6	0	3.815011	-2.809261	-0.326135
10	1	0	4.631417	-2.094211	-0.177056
11	1	0	3.814321	-3.518484	0.502495
12	1	0	3.992326	-3.357052	-1.255931
13	6	0	-0.387849	-1.496447	1.860863
14	8	0	-1.442990	-0.842654	1.905780
15	6	0	-0.103576	-2.489690	2.982343
16	1	0	-0.156474	-3.520223	2.615759
17	1	0	0.888162	-2.342604	3.420048
18	1	0	-0.858947	-2.356911	3.757815
19	6	0	0.320341	-0.339276	-0.355982
20	1	0	-0.738209	-0.083410	-0.409465
21	6	0	2.900543	3.126567	0.515750
22	6	0	3.287186	2.219131	-0.654388
23	6	0	1.395732	3.396575	0.502736
24	1	0	3.199467	2.761015	-1.607017
25	1	0	4.327051	1.886237	-0.580653
26	1	0	3.177271	2.637629	1.457383
27	1	0	3.459481	4.065415	0.464523
28	1	0	1.132337	4.006871	-0.373966
29	1	0	1.069573	3.956869	1.383576
30	6	0	0.555165	2.128709	0.420970
31	6	0	2.401593	1.006980	-0.712223
32	8	0	2.999961	-0.011346	-1.370659
33	8	0	-0.612912	2.149824	0.830146
34	6	0	1.139523	0.940080	-0.194164
35	1	0	0.255568	-1.939461	-1.849692
36	19	0	-3.059064	1.108783	1.112785
37	6	0	-3.986672	-0.305833	-1.391886
38	8	0	-4.629927	-0.573214	-0.351606
39	8	0	-4.349974	-1.052261	-2.515144
40	1	0	-3.772095	-0.733105	-3.223989
41	8	0	-3.063630	0.536644	-1.559715

E(RB3LYP/6-31G(d,p)) = -1688.61517233 Ha

ΔG (298.15 K, 1 atm, B3LYP/6-31G(d,p)) = -1688.364792 Ha

E(RB3LYP/6-311++G(d,p)//B3LYP/6-31G(d,p)) = -1688.95123994 Ha

ΔG (298.15 K, 1 atm, B3LYP/6-311++G(d,p)//B3LYP/6-31G(d,p)) = -1688.700860 Ha

6a-KHCO$_3$ – conformation 3

Center Number	Atomic Number	Atomic Type	X	Y	Z
1	6	0	-3.668000	0.222852	1.315332
2	6	0	-3.256124	-1.577220	-0.265180
3	6	0	-1.914262	-1.603574	-0.015707
4	6	0	-2.416813	-0.290914	2.010351
5	1	0	-4.485863	0.429219	2.006584
6	1	0	-3.693603	-2.309735	-0.935383
7	1	0	-2.086961	0.453138	2.739937
8	7	0	-4.129731	-0.702975	0.311161
9	6	0	-5.503596	-0.567984	-0.164623
10	1	0	-5.631895	0.347537	-0.752609
11	1	0	-5.754270	-1.424472	-0.791715
12	1	0	-6.194598	-0.540104	0.682980
13	6	0	-1.135448	-2.699058	-0.604643
14	8	0	-1.626162	-3.483673	-1.428210
15	6	0	0.302257	-2.885314	-0.153001
16	1	0	0.330272	-3.174746	0.904940
17	1	0	0.764960	-3.675391	-0.746857
18	1	0	0.865276	-1.953273	-0.247721
19	6	0	-1.368779	-0.517923	0.909354
20	1	0	-0.415951	-0.820543	1.346016
21	6	0	-0.629805	3.567133	-0.570309
22	6	0	0.229577	2.459924	-1.184229
23	6	0	-2.083305	3.106475	-0.446853
24	1	0	-0.074680	2.284004	-2.226763
25	1	0	1.289383	2.729345	-1.203231
26	1	0	-0.244149	3.817745	0.424953
27	1	0	-0.572959	4.475998	-1.176154
28	1	0	-2.557986	3.059833	-1.437651
29	1	0	-2.680565	3.803857	0.148712
30	6	0	-2.179826	1.744701	0.179679
31	6	0	0.093839	1.134160	-0.450423
32	8	0	1.043125	0.333284	-0.444663
33	8	0	-3.398265	1.536105	0.720370
34	6	0	-1.170282	0.822786	0.200555
35	1	0	-2.649283	-1.218766	2.539627
36	19	0	3.530679	-0.046762	-1.392808
37	6	0	5.571085	-0.183194	0.797640
38	8	0	5.080206	-1.280184	0.442883
39	8	0	6.537848	-0.269231	1.800340
40	1	0	6.814550	0.645304	1.959898
41	8	0	5.310802	0.974105	0.372029

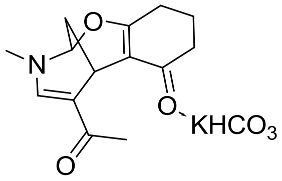

E(RB3LYP/6-31G(d,p)) = -1688.60603962 Ha

ΔG (298.15 K, 1 atm, B3LYP/6-31G(d,p)) = -1688.358868 Ha

E(RB3LYP/6-311++G(d,p)//B3LYP/6-31G(d,p)) = -1688.94485751 Ha

ΔG (298.15 K, 1 atm, B3LYP/6-311++G(d,p)//B3LYP/6-31G(d,p)) = -1688.697687 Ha

6a-KHCO$_3$ – conformation 4

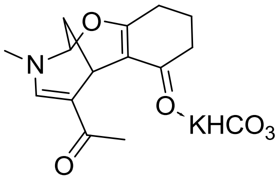

Center Number	Atomic Number	Atomic Type	X	Y	Z
1	6	0	-3.761050	-0.705448	-0.450553
2	6	0	-3.053091	1.137776	0.959250
3	6	0	-2.020580	1.480293	0.134536
4	6	0	-3.136724	0.098756	-1.579633
5	1	0	-4.733772	-1.123871	-0.711296
6	1	0	-3.257256	1.736762	1.840583
7	1	0	-3.040277	-0.539553	-2.461667
8	7	0	-3.902029	0.092998	0.740812
9	6	0	-4.817720	-0.375933	1.776916
10	1	0	-4.439515	-1.282842	2.261902
11	1	0	-4.936155	0.401821	2.532401
12	1	0	-5.797305	-0.593153	1.341489
13	6	0	-1.275940	2.703856	0.453776
14	8	0	-1.482356	3.346090	1.492738
15	6	0	-0.248270	3.204832	-0.546008
16	1	0	0.431295	2.405976	-0.854375
17	1	0	-0.753823	3.564051	-1.451476
18	1	0	0.307368	4.033111	-0.103012
19	6	0	-1.767187	0.577375	-1.070469
20	1	0	-1.236446	1.117454	-1.855746
21	6	0	0.533302	-2.892856	0.374418
22	6	0	-0.933530	-3.111766	-0.002367
23	6	0	1.228266	-2.008566	-0.662466
24	1	0	-1.011178	-3.763511	-0.884548
25	1	0	-1.488538	-3.607482	0.800044
26	1	0	0.584131	-2.411527	1.358425
27	1	0	1.046439	-3.855018	0.460932
28	1	0	1.299240	-2.544954	-1.621139
29	1	0	2.251142	-1.753939	-0.366244
30	6	0	0.476180	-0.716743	-0.944396
31	6	0	-1.611331	-1.808657	-0.316278
32	8	0	-2.949877	-1.896914	-0.170883
33	8	0	1.088541	0.264808	-1.399404
34	6	0	-0.963791	-0.677824	-0.729308
35	1	0	-3.790652	0.939090	-1.827815
36	19	0	3.643662	1.132376	-1.372237
37	6	0	4.537307	-0.280736	1.122462
38	8	0	4.540074	-0.976958	0.077596
39	8	0	4.862604	-0.972497	2.287788
40	1	0	4.820945	-0.310068	2.993437
41	8	0	4.280253	0.944891	1.251619

E(RB3LYP/6-31G(d,p)) = -1688.60782859 Ha

ΔG (298.15 K, 1 atm, B3LYP/6-31G(d,p)) = -1688.357983 Ha

E(RB3LYP/6-311++G(d,p)//B3LYP/6-31G(d,p)) = -1688.94463786 Ha

ΔG (298.15 K, 1 atm, B3LYP/6-311++G(d,p)//B3LYP/6-31G(d,p)) = -1688.694791 Ha

6a-$KHCO_3$ – conformation 5 (related to conformation 1)

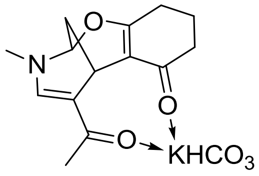

Center Number	Atomic Number	Atomic Type	X	Y	Z
1	6	0	-3.734946	-0.760082	0.666236
2	6	0	-2.097698	-2.099269	-0.540741
3	6	0	-1.039192	-1.526014	0.111088
4	6	0	-2.675076	-0.698679	1.755712
5	1	0	-4.729121	-0.990155	1.050933
6	1	0	-1.964289	-2.914053	-1.243681
7	1	0	-2.957544	0.070566	2.479078
8	7	0	-3.394777	-1.737539	-0.343865
9	6	0	-4.463244	-2.202003	-1.225381
10	1	0	-4.810072	-1.395001	-1.879509
11	1	0	-4.093631	-3.020316	-1.844366
12	1	0	-5.309053	-2.563721	-0.633597
13	6	0	0.301684	-2.069641	-0.005057
14	8	0	1.235505	-1.647472	0.698201
15	6	0	0.583839	-3.204061	-0.983437
16	1	0	0.122315	-4.136820	-0.642275
17	1	0	0.200677	-2.989725	-1.985199
18	1	0	1.663316	-3.350731	-1.037594
19	6	0	-1.352478	-0.366538	1.050260
20	1	0	-0.532329	-0.253701	1.760425
21	6	0	-2.074887	3.689269	-0.521284
22	6	0	-0.669141	3.119962	-0.728343
23	6	0	-3.131812	2.616720	-0.793583
24	1	0	-0.523123	2.860681	-1.787446
25	1	0	0.109776	3.840708	-0.465646
26	1	0	-2.175869	4.042158	0.511977
27	1	0	-2.238834	4.552405	-1.172878
28	1	0	-3.205577	2.414566	-1.871939
29	1	0	-4.127330	2.938688	-0.472036
30	6	0	-2.803123	1.325333	-0.100563
31	6	0	-0.428450	1.854603	0.081723
32	8	0	0.706796	1.598664	0.502570
33	8	0	-3.905808	0.558162	0.060102
34	6	0	-1.556453	0.957312	0.316777
35	1	0	-2.627570	-1.661301	2.271905
36	19	0	2.931276	0.320623	1.178244
37	6	0	5.419653	0.049849	-0.511387
38	8	0	4.435855	0.584915	-1.072458
39	8	0	6.548203	-0.096974	-1.322110
40	1	0	7.209689	-0.518448	-0.753739
41	8	0	5.527664	-0.369223	0.671682

E(RB3LYP/6-31G(d,p)) = -1688.61452800 Ha

ΔG (298.15 K, 1 atm, B3LYP/6-31G(d,p)) = -1688.366743 Ha

E(RB3LYP/6-311++G(d,p)//B3LYP/6-31G(d,p)) = -1688.95137477 Ha

ΔG (298.15 K, 1 atm, B3LYP/6-311++G(d,p)//B3LYP/6-31G(d,p)) = -1688.703590 Ha

6a-KHCO$_3$ – conformation 6 (related to conformation 1)

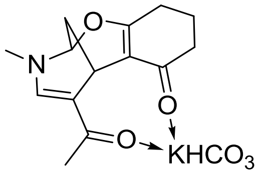

Center Number	Atomic Number	Atomic Type	Coordinates (Angstroms) X	Y	Z
1	6	0	-3.811871	-0.329082	0.705946
2	6	0	-2.469179	-1.900240	-0.583593
3	6	0	-1.309068	-1.541973	0.048714
4	6	0	-2.728566	-0.486315	1.762177
5	1	0	-4.820418	-0.393967	1.115735
6	1	0	-2.501637	-2.706224	-1.308276
7	1	0	-2.851029	0.299482	2.512066
8	7	0	-3.675340	-1.319410	-0.339246
9	6	0	-4.833374	-1.564318	-1.195484
10	1	0	-5.062481	-0.683100	-1.803967
11	1	0	-4.622972	-2.403143	-1.859817
12	1	0	-5.708380	-1.808940	-0.586267
13	6	0	-0.089751	-2.311694	-0.118290
14	8	0	0.920502	-2.083839	0.568822
15	6	0	-0.037875	-3.449348	-1.131746
16	1	0	-0.638730	-4.299838	-0.792370
17	1	0	-0.413472	-3.146410	-2.113201
18	1	0	0.998405	-3.775165	-1.230611
19	6	0	-1.388021	-0.371590	1.022481
20	1	0	-0.542108	-0.423652	1.709030
21	6	0	-1.424337	3.788782	-0.435947
22	6	0	-0.150539	2.984540	-0.707211
23	6	0	-2.663909	2.929348	-0.693337
24	1	0	-0.087989	2.732887	-1.776337
25	1	0	0.752899	3.547421	-0.458053
26	1	0	-1.427361	4.124214	0.608001
27	1	0	-1.452144	4.685604	-1.061514
28	1	0	-2.807162	2.774224	-1.772480
29	1	0	-3.575217	3.413625	-0.328471
30	6	0	-2.549141	1.581014	-0.041546
31	6	0	-0.113795	1.674615	0.065850
32	8	0	0.970882	1.208126	0.436544
33	8	0	-3.765168	1.016647	0.139579
34	6	0	-1.375743	0.986814	0.324560
35	1	0	-2.837038	-1.456778	2.253908
36	19	0	2.920970	-0.440831	1.128282
37	6	0	5.461456	0.117096	-0.407739
38	8	0	5.471103	0.476182	0.792214
39	8	0	6.641575	0.381459	-1.108008
40	1	0	6.478532	0.050746	-2.003749
41	8	0	4.542298	-0.444225	-1.061059

E(RB3LYP/6-31G(d,p)) = -1688.61461078 Ha

ΔG (298.15 K, 1 atm, B3LYP/6-31G(d,p)) = -1688.367247 Ha

E(RB3LYP/6-311++G(d,p)//B3LYP/6-31G(d,p)) = -1688.95148722 Ha

ΔG (298.15 K, 1 atm, B3LYP/6-311++G(d,p)//B3LYP/6-31G(d,p)) = -1688.704123 Ha

6a-KHCO$_3$ – conformation 7 (related to conformation 1)

Center Number	Atomic Number	Atomic Type	Coordinates (Angstroms) X	Y	Z
1	6	0	-2.540508	0.141710	1.702763
2	6	0	-2.452806	-1.737849	0.154349
3	6	0	-1.119692	-1.563569	-0.103536
4	6	0	-1.061900	-0.143230	1.908921
5	1	0	-3.081936	0.295466	2.636950
6	1	0	-3.012948	-2.568191	-0.260978
7	1	0	-0.609795	0.701015	2.435794
8	7	0	-3.182255	-0.926400	0.968900
9	6	0	-4.640029	-1.003961	1.020177
10	1	0	-5.096912	-0.154100	0.501865
11	1	0	-4.971910	-1.925964	0.541344
12	1	0	-4.980590	-1.006096	2.059592
13	6	0	-0.364067	-2.551740	-0.853369
14	8	0	0.868377	-2.470137	-0.984151
15	6	0	-1.079743	-3.743392	-1.481112
16	1	0	-1.427706	-4.439417	-0.710433
17	1	0	-1.948254	-3.440232	-2.072851
18	1	0	-0.372816	-4.266264	-2.126575
19	6	0	-0.457036	-0.334664	0.510616
20	1	0	0.620824	-0.487087	0.577117
21	6	0	-0.956403	3.633660	-1.342167
22	6	0	-0.233614	2.579298	-2.183676
23	6	0	-2.167099	3.015899	-0.639845
24	1	0	-0.884697	2.247372	-3.006014
25	1	0	0.680367	2.970866	-2.638201
26	1	0	-0.266787	4.033942	-0.589442
27	1	0	-1.272282	4.473668	-1.967608
28	1	0	-2.970648	2.815625	-1.363362
29	1	0	-2.589803	3.692714	0.109495
30	6	0	-1.815304	1.721018	0.035096
31	6	0	0.138196	1.343699	-1.376252
32	8	0	1.147357	0.693958	-1.678120
33	8	0	-2.708844	1.413616	1.003789
34	6	0	-0.739535	0.941696	-0.280093
35	1	0	-0.944645	-1.039569	2.523923
36	19	0	3.101521	-1.036462	-1.121359
37	6	0	3.803560	0.344345	1.470916
38	8	0	3.088883	-0.679699	1.582859
39	8	0	3.995589	1.056636	2.656883
40	1	0	4.560110	1.804158	2.410253
41	8	0	4.362785	0.812148	0.444479

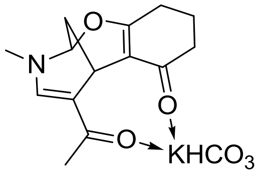

E(RB3LYP/6-31G(d,p)) = -1688.61530216 Ha

ΔG (298.15 K, 1 atm, B3LYP/6-31G(d,p)) = -1688.365117 Ha

E(RB3LYP/6-311++G(d,p)//B3LYP/6-31G(d,p)) = -1688.95119437 Ha

ΔG (298.15 K, 1 atm, B3LYP/6-311++G(d,p)//B3LYP/6-31G(d,p)) = -1688.701009 Ha

6a-KHCO$_3$ – conformation 8 (related to conformation 2)

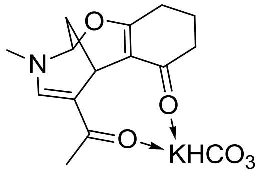

Center Number	Atomic Number	Atomic Type	X	Y	Z
1	6	0	-3.743868	-0.658363	0.736396
2	6	0	-2.216277	-2.055572	-0.536038
3	6	0	-1.110561	-1.519764	0.067003
4	6	0	-2.647141	-0.652264	1.789790
5	1	0	-4.736840	-0.825992	1.154628
6	1	0	-2.139629	-2.863351	-1.255249
7	1	0	-2.867795	0.121319	2.529737
8	7	0	-3.492473	-1.662955	-0.270953
9	6	0	-4.610833	-2.076272	-1.115266
10	1	0	-4.922780	-1.264787	-1.781665
11	1	0	-4.311493	-2.932575	-1.720830
12	1	0	-5.461320	-2.367491	-0.492747
13	6	0	0.211113	-2.080454	-0.150461
14	8	0	1.198832	-1.673692	0.484450
15	6	0	0.407698	-3.207823	-1.157343
16	1	0	-0.073538	-4.129311	-0.813164
17	1	0	-0.010879	-2.959638	-2.137124
18	1	0	1.477533	-3.391981	-1.262972
19	6	0	-1.333877	-0.371066	1.044475
20	1	0	-0.486028	-0.316041	1.728201
21	6	0	-1.773535	3.294880	-1.353689
22	6	0	-0.595882	3.269669	-0.378835
23	6	0	-3.026598	2.720369	-0.688515
24	1	0	0.340359	3.574165	-0.855316
25	1	0	-0.774657	3.977772	0.443913
26	1	0	-1.964731	4.315383	-1.697662
27	1	0	-1.529363	2.696460	-2.239354
28	1	0	-3.840408	2.585983	-1.407771
29	1	0	-3.402546	3.407114	0.083401
30	6	0	-2.735193	1.394606	-0.045734
31	6	0	-0.370212	1.905770	0.260096
32	8	0	0.743181	1.629980	0.726457
33	8	0	-3.858662	0.659740	0.110199
34	6	0	-1.497943	0.985051	0.362814
35	1	0	-2.628525	-1.621684	2.295164
36	19	0	2.923069	0.193917	1.197631
37	6	0	5.419801	0.002394	-0.491474
38	8	0	4.432226	0.544251	-1.039173
39	8	0	6.549744	-0.115330	-1.305098
40	1	0	7.214027	-0.546462	-0.747334
41	8	0	5.530611	-0.446149	0.680478

E(RB3LYP/6-31G(d,p)) = -1688.61457604 Ha

ΔG (298.15 K, 1 atm, B3LYP/6-31G(d,p)) = -1688.366497 Ha

E(RB3LYP/6-311++G(d,p)//B3LYP/6-31G(d,p)) = -1688.95150002 Ha

ΔG (298.15 K, 1 atm, B3LYP/6-311++G(d,p)//B3LYP/6-31G(d,p)) = -1688.703421 Ha

6a-KHCO$_3$ – conformation 9 (related to conformation 2)

Center Number	Atomic Number	Atomic Type	Coordinates (Angstroms) X	Y	Z
1	6	0	-3.805913	-0.261540	0.773874
2	6	0	-2.558650	-1.854121	-0.573765
3	6	0	-1.366342	-1.525435	0.012622
4	6	0	-2.699037	-0.465790	1.796344
5	1	0	-4.803203	-0.278180	1.214473
6	1	0	-2.633386	-2.643835	-1.312946
7	1	0	-2.772210	0.312007	2.560781
8	7	0	-3.745193	-1.262107	-0.266854
9	6	0	-4.937754	-1.464961	-1.085954
10	1	0	-5.149080	-0.580776	-1.696889
11	1	0	-4.783156	-2.318518	-1.746954
12	1	0	-5.802332	-1.667284	-0.447459
13	6	0	-0.161391	-2.291554	-0.250489
14	8	0	0.893197	-2.072287	0.369071
15	6	0	-0.176276	-3.409211	-1.286901
16	1	0	-0.784300	-4.252719	-0.943276
17	1	0	-0.582304	-3.075158	-2.246109
18	1	0	0.847587	-3.755400	-1.433719
19	6	0	-1.375225	-0.382579	1.021733
20	1	0	-0.514142	-0.485667	1.683016
21	6	0	-1.264525	3.369662	-1.279502
22	6	0	-0.081089	3.122950	-0.343354
23	6	0	-2.576800	2.992869	-0.587782
24	1	0	0.879250	3.280486	-0.842531
25	1	0	-0.116348	3.828448	0.500003
26	1	0	-1.292490	4.416784	-1.594220
27	1	0	-1.148140	2.763512	-2.185703
28	1	0	-3.421334	3.015717	-1.283384
29	1	0	-2.811138	3.710587	0.211461
30	6	0	-2.492590	1.619555	0.014418
31	6	0	-0.068319	1.724003	0.258290
32	8	0	0.996012	1.255177	0.683443
33	8	0	-3.717918	1.076122	0.186664
34	6	0	-1.330181	1.000187	0.375996
35	1	0	-2.828076	-1.439263	2.277218
36	19	0	2.909232	-0.523736	1.105128
37	6	0	5.466049	0.074794	-0.388493
38	8	0	5.493077	0.323445	0.838815
39	8	0	6.658340	0.341597	-1.066651
40	1	0	6.479340	0.101986	-1.987980
41	8	0	4.520375	-0.379050	-1.086026

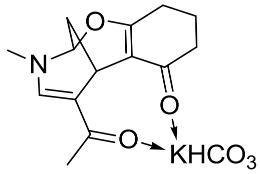

E(RB3LYP/6-31G(d,p)) = -1688.61463046 Ha

ΔG (298.15 K, 1 atm, B3LYP/6-31G(d,p)) = -1688.366939 Ha

E(RB3LYP/6-311++G(d,p)//B3LYP/6-31G(d,p)) = -1688.95159980 Ha

ΔG (298.15 K, 1 atm, B3LYP/6-311++G(d,p)//B3LYP/6-31G(d,p)) = -1688.703909 Ha

6a-KHCO$_3$ – conformation 10 (related to conformation 2), **23** (this conformation was shown by IRC, starting from TS **22a**).

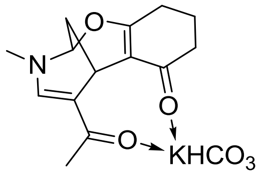

Center Number	Atomic Number	Atomic Type	Coordinates (Angstroms) X	Y	Z
1	6	0	-2.422954	-0.229522	1.798829
2	6	0	-2.286499	-1.884471	0.025005
3	6	0	-1.006256	-1.531936	-0.307141
4	6	0	-0.914641	-0.391685	1.879991
5	1	0	-2.906723	-0.216174	2.776035
6	1	0	-2.799588	-2.701543	-0.469590
7	1	0	-0.498936	0.419819	2.482724
8	7	0	-3.014337	-1.281911	1.005636
9	6	0	-4.447617	-1.522627	1.152322
10	1	0	-5.030543	-0.678269	0.768491
11	1	0	-4.723987	-2.420397	0.598075
12	1	0	-4.696406	-1.671725	2.206764
13	6	0	-0.253060	-2.288983	-1.292140
14	8	0	0.934887	-2.029091	-1.544556
15	6	0	-0.913862	-3.445732	-2.034736
16	1	0	-1.141359	-4.271917	-1.353084
17	1	0	-1.849252	-3.143577	-2.515091
18	1	0	-0.221335	-3.803500	-2.797626
19	6	0	-0.391383	-0.350035	0.436399
20	1	0	0.696034	-0.430169	0.425566
21	6	0	-1.883056	3.287853	-1.550838
22	6	0	-0.368866	3.078547	-1.584975
23	6	0	-2.434506	2.950450	-0.163574
24	1	0	0.043251	3.210564	-2.589462
25	1	0	0.126487	3.820813	-0.941506
26	1	0	-2.133968	4.319278	-1.814824
27	1	0	-2.359891	2.639397	-2.295468
28	1	0	-3.528818	2.943954	-0.154661
29	1	0	-2.120780	3.707138	0.569762
30	6	0	-1.945898	1.607237	0.298625
31	6	0	0.062861	1.707152	-1.081735
32	8	0	1.155594	1.247608	-1.438867
33	8	0	-2.765520	1.077357	1.234241
34	6	0	-0.803635	1.001801	-0.142133
35	1	0	-0.679920	-1.341533	2.368062
36	19	0	3.130251	-0.540715	-1.347874
37	6	0	3.862562	0.091877	1.515541
38	8	0	3.123094	-0.907978	1.356766
39	8	0	4.089180	0.446563	2.847363
40	1	0	4.670037	1.220546	2.803922
41	8	0	4.420534	0.811129	0.645733

E(RB3LYP/6-31G(d,p)) = -1688.61521665 Ha

ΔG (298.15 K, 1 atm, B3LYP/6-31G(d,p)) = -1688.365380 Ha

E(RB3LYP/6-311++G(d,p)//B3LYP/6-31G(d,p)) = -1688.95123968 Ha

ΔG (298.15 K, 1 atm, B3LYP/6-311++G(d,p)//B3LYP/6-31G(d,p)) = -1688.701404 Ha

6a-KHCO$_3$ – conformation 11 (related to conformation 3)

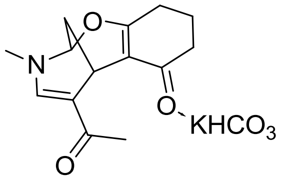

Center Number	Atomic Number	Atomic Type	Coordinates (Angstroms) X	Y	Z
1	6	0	-3.698813	-1.102443	-0.672593
2	6	0	-3.504939	0.838019	0.779701
3	6	0	-2.440723	1.350478	0.095303
4	6	0	-3.045181	-0.209215	-1.715698
5	1	0	-4.556909	-1.652902	-1.060158
6	1	0	-3.939662	1.400679	1.599152
7	1	0	-2.718983	-0.827948	-2.555671
8	7	0	-4.115222	-0.344438	0.481701
9	6	0	-5.085281	-0.953703	1.387001
10	1	0	-4.646902	-1.800277	1.926921
11	1	0	-5.417864	-0.210451	2.112731
12	1	0	-5.954215	-1.308566	0.825082
13	6	0	-1.992841	2.702061	0.449482
14	8	0	-2.447792	3.308071	1.429636
15	6	0	-0.966405	3.379183	-0.441559
16	1	0	-1.390322	3.557692	-1.437613
17	1	0	-0.687191	4.337217	0.000240
18	1	0	-0.085814	2.744834	-0.573797
19	6	0	-1.861969	0.480782	-1.018244
20	1	0	-1.297780	1.084718	-1.730395
21	6	0	0.822858	-2.889616	-0.103373
22	6	0	1.345026	-1.496106	0.254497
23	6	0	-0.649762	-3.027755	0.286929
24	1	0	1.346795	-1.370199	1.348129
25	1	0	2.373484	-1.341007	-0.086305
26	1	0	0.926840	-3.051379	-1.183063
27	1	0	1.417954	-3.660286	0.395219
28	1	0	-0.753861	-3.089885	1.379915
29	1	0	-1.093886	-3.942859	-0.117363
30	6	0	-1.459493	-1.856710	-0.191468
31	6	0	0.478710	-0.383501	-0.314291
32	8	0	0.985475	0.717632	-0.586872
33	8	0	-2.770472	-2.167376	-0.282835
34	6	0	-0.946646	-0.626527	-0.493538
35	1	0	-3.774752	0.518278	-2.081316
36	19	0	3.458416	1.794824	-0.584611
37	6	0	5.250416	-0.404208	0.406360
38	8	0	4.672321	-0.629096	-0.685004
39	8	0	6.027440	-1.457866	0.884746
40	1	0	6.402843	-1.135665	1.717649
41	8	0	5.217670	0.635257	1.115990

E(RB3LYP/6-31G(d,p)) = -1688.60773041 Ha

ΔG (298.15 K, 1 atm, B3LYP/6-31G(d,p)) = -1688.359171 Ha

E(RB3LYP/6-311++G(d,p)//B3LYP/6-31G(d,p)) = -1688.94478021 Ha

ΔG (298.15 K, 1 atm, B3LYP/6-311++G(d,p)//B3LYP/6-31G(d,p)) = -1688.696220 Ha

$6a\text{-}KHCO_3$ – conformation 12 (related to conformation 3)

Center Number	Atomic Number	Atomic Type	Coordinates (Angstroms) X	Y	Z
1	6	0	2.288533	2.266643	0.722939
2	6	0	0.272132	1.300525	-0.237520
3	6	0	0.420528	0.115871	0.433525
4	6	0	2.029176	1.305275	1.872570
5	1	0	2.524793	3.276961	1.058940
6	1	0	-0.608112	1.467385	-0.849315
7	1	0	2.921916	1.259359	2.501499
8	7	0	1.151573	2.332851	-0.170921
9	6	0	1.048883	3.491943	-1.053882
10	1	0	1.873218	3.505561	-1.774554
11	1	0	0.106436	3.447938	-1.600448
12	1	0	1.075524	4.417657	-0.471049
13	6	0	-0.671269	-0.845345	0.363897
14	8	0	-1.658171	-0.666383	-0.377293
15	6	0	-0.615740	-2.078599	1.242575
16	1	0	-0.709555	-1.789505	2.296747
17	1	0	-1.443567	-2.742577	0.986828
18	1	0	0.340807	-2.596433	1.131635
19	6	0	1.707910	-0.055892	1.236028
20	1	0	1.588349	-0.820360	2.005047
21	6	0	5.492288	-1.125826	-0.745425
22	6	0	4.372994	-2.166853	-0.821712
23	6	0	4.933506	0.277950	-0.989608
24	1	0	3.972877	-2.209510	-1.845570
25	1	0	4.726348	-3.171995	-0.576424
26	1	0	5.956605	-1.161775	0.247226
27	1	0	6.276060	-1.350231	-1.474912
28	1	0	4.667745	0.407272	-2.048714
29	1	0	5.672296	1.052740	-0.761037
30	6	0	3.705534	0.534945	-0.163374
31	6	0	3.206753	-1.842855	0.103580
32	8	0	2.530950	-2.756808	0.587808
33	8	0	3.486641	1.862815	-0.004856
34	6	0	2.904673	-0.434560	0.363738
35	1	0	1.195823	1.674172	2.476454
36	19	0	-4.069817	-1.420776	-1.136046
37	6	0	-6.111535	0.387527	0.112308
38	8	0	-5.692972	0.739062	-1.015191
39	8	0	-7.060245	1.240788	0.678150
40	1	0	-7.279497	0.839169	1.531973
41	8	0	-5.793549	-0.622666	0.795027

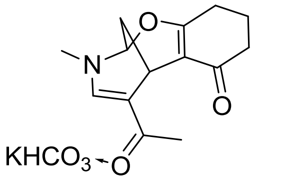

E(RB3LYP/6-31G(d,p)) = -1688.60726163 Ha

ΔG (298.15 K, 1 atm, B3LYP/6-31G(d,p)) = -1688.361254 Ha

E(RB3LYP/6-311++G(d,p)//B3LYP/6-31G(d,p)) = -1688.94597816 Ha

ΔG (298.15 K, 1 atm, B3LYP/6-311++G(d,p)//B3LYP/6-31G(d,p)) = -1688.699970 Ha

6a-$KHCO_3$ – conformation 13 (related to conformation 3)

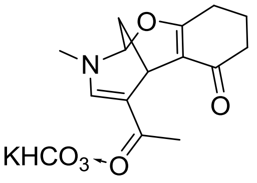

Center Number	Atomic Number	Atomic Type	X	Y	Z
1	6	0	-3.014154	-1.941553	-0.526697
2	6	0	-0.593431	-1.831133	-0.308424
3	6	0	-0.647192	-1.048898	0.814456
4	6	0	-3.017024	-1.705748	0.975935
5	1	0	-3.683924	-2.746488	-0.831692
6	1	0	0.364970	-2.183078	-0.675076
7	1	0	-4.031778	-1.448544	1.290113
8	7	0	-1.682153	-2.244143	-1.006400
9	6	0	-1.561339	-2.904434	-2.303728
10	1	0	-1.923179	-2.253224	-3.106269
11	1	0	-0.514171	-3.143259	-2.491669
12	1	0	-2.142106	-3.831736	-2.313815
13	6	0	0.598760	-0.794119	1.523981
14	8	0	1.699932	-1.170221	1.074814
15	6	0	0.546511	-0.081691	2.860135
16	1	0	0.058852	-0.722610	3.605256
17	1	0	1.562868	0.133991	3.194867
18	1	0	-0.039209	0.838880	2.791088
19	6	0	-2.029676	-0.557571	1.237236
20	1	0	-2.037567	-0.276780	2.291383
21	6	0	-3.935736	2.856084	-0.792735
22	6	0	-2.642445	3.158034	-0.031912
23	6	0	-3.803165	1.546135	-1.573173
24	1	0	-1.826604	3.348562	-0.744827
25	1	0	-2.730654	4.048652	0.596019
26	1	0	-4.765635	2.769960	-0.081315
27	1	0	-4.180834	3.675980	-1.474275
28	1	0	-3.138479	1.679766	-2.438869
29	1	0	-4.766685	1.214678	-1.973383
30	6	0	-3.241252	0.447004	-0.717255
31	6	0	-2.201753	2.001659	0.856502
32	8	0	-1.579752	2.226993	1.899908
33	8	0	-3.551160	-0.774191	-1.216819
34	6	0	-2.509880	0.639704	0.417142
35	1	0	-2.714607	-2.622056	1.489962
36	19	0	4.316916	-0.929698	0.956895
37	6	0	5.188865	1.026197	-1.143091
38	8	0	5.064028	1.491345	0.013584
39	8	0	5.587387	1.952672	-2.108033
40	1	0	5.638813	1.447796	-2.933097
41	8	0	5.004696	-0.153503	-1.546058

E(RB3LYP/6-31G(d,p)) = -1688.60714841 Ha

ΔG (298.15 K, 1 atm, B3LYP/6-31G(d,p)) = -1688.361098 Ha

E(RB3LYP/6-311++G(d,p)//B3LYP/6-31G(d,p)) = -1688.94583479 Ha

ΔG (298.15 K, 1 atm, B3LYP/6-311++G(d,p)//B3LYP/6-31G(d,p)) = -1688.699784 Ha

6a-KHCO$_3$ – conformation 14 (related to conformation 4)

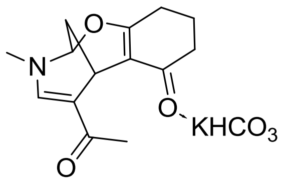

Center Number	Atomic Number	Atomic Type	Coordinates (Angstroms) X	Y	Z
1	6	0	3.362333	-0.707246	1.482796
2	6	0	3.544371	1.222927	0.025207
3	6	0	2.206954	1.491225	0.076046
4	6	0	2.142608	0.004541	2.045694
5	1	0	4.003691	-1.132284	2.255510
6	1	0	4.204413	1.886581	-0.523324
7	1	0	1.570315	-0.697066	2.657931
8	7	0	4.147154	0.176515	0.659225
9	6	0	5.525585	-0.203251	0.362712
10	1	0	5.565184	-1.072450	-0.303403
11	1	0	6.033858	0.632116	-0.120686
12	1	0	6.054673	-0.446303	1.288529
13	6	0	1.738563	2.725278	-0.565687
14	8	0	2.492311	3.442944	-1.237365
15	6	0	0.291292	3.139447	-0.364701
16	1	0	-0.393670	2.318397	-0.593061
17	1	0	0.122832	3.415198	0.683966
18	1	0	0.077183	4.004756	-0.994261
19	6	0	1.329773	0.501169	0.838681
20	1	0	0.405771	0.976240	1.171145
21	6	0	0.544341	-2.890911	-1.866764
22	6	0	1.503619	-3.113774	-0.695516
23	6	0	-0.677681	-2.093153	-1.409362
24	1	0	1.074521	-3.826136	0.023630
25	1	0	2.454094	-3.542156	-1.027921
26	1	0	1.065004	-2.340887	-2.659501
27	1	0	0.237464	-3.851816	-2.289449
28	1	0	-1.282975	-2.696812	-0.716978
29	1	0	-1.329663	-1.827203	-2.246589
30	6	0	-0.315680	-0.811594	-0.672811
31	6	0	1.786785	-1.826900	0.025813
32	8	0	2.946726	-1.886199	0.710290
33	8	0	-1.132170	0.124028	-0.634982
34	6	0	0.960913	-0.736872	0.020964
35	1	0	2.469714	0.833165	2.679476
36	19	0	-3.631122	0.867124	-1.238050
37	6	0	-5.414798	-0.065045	0.982309
38	8	0	-4.832170	1.027354	1.175534
39	8	0	-6.231088	-0.488495	2.031707
40	1	0	-6.603492	-1.331861	1.734465
41	8	0	-5.361305	-0.823066	-0.023108

E(RB3LYP/6-31G(d,p)) = -1688.60607990 Ha

ΔG (298.15 K, 1 atm, B3LYP/6-31G(d,p)) = -1688.359622 Ha

E(RB3LYP/6-311++G(d,p)//B3LYP/6-31G(d,p)) = -1688.94508551 Ha

ΔG (298.15 K, 1 atm, B3LYP/6-311++G(d,p)//B3LYP/6-31G(d,p)) = -1688.698628 Ha

6a-KHCO$_3$ – conformation 15 (related to conformation 4)

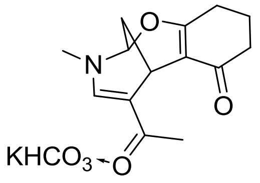

Center Number	Atomic Number	Atomic Type	X	Y	Z
1	6	0	1.122884	-2.093228	-1.120023
2	6	0	-0.538468	-0.453365	-0.440209
3	6	0	0.226815	0.608205	-0.853343
4	6	0	1.548307	-1.040897	-2.131341
5	1	0	1.044232	-3.091616	-1.551778
6	1	0	-1.537621	-0.302824	-0.040726
7	1	0	2.515071	-1.323339	-2.556019
8	7	0	-0.143478	-1.750115	-0.515106
9	6	0	-0.922268	-2.821251	0.106864
10	1	0	-0.369792	-3.256410	0.947119
11	1	0	-1.865990	-2.408337	0.469234
12	1	0	-1.125815	-3.612102	-0.622517
13	6	0	-0.363431	1.937147	-0.807254
14	8	0	-1.483429	2.154958	-0.302272
15	6	0	0.396114	3.096290	-1.423252
16	1	0	1.430383	3.128661	-1.069779
17	1	0	0.431669	2.980486	-2.513866
18	1	0	-0.120184	4.028417	-1.187436
19	6	0	1.630049	0.289624	-1.366094
20	1	0	1.994729	1.083692	-2.019086
21	6	0	4.216859	-0.224084	2.165214
22	6	0	3.840112	-1.473262	1.364635
23	6	0	4.582528	0.922752	1.221620
24	1	0	4.730500	-1.906735	0.886918
25	1	0	3.422702	-2.253663	2.008608
26	1	0	3.366361	0.072925	2.790421
27	1	0	5.048861	-0.447031	2.839650
28	1	0	5.508651	0.679553	0.680085
29	1	0	4.769315	1.856578	1.759180
30	6	0	3.513033	1.195833	0.169795
31	6	0	2.835744	-1.149488	0.295069
32	8	0	2.147590	-2.250104	-0.088213
33	8	0	3.428698	2.318997	-0.339337
34	6	0	2.652800	0.088599	-0.247900
35	1	0	0.812354	-0.997948	-2.938931
36	19	0	-3.618287	1.904685	1.197071
37	6	0	-4.512704	-0.836593	0.290442
38	8	0	-3.503090	-0.827566	1.042571
39	8	0	-4.868007	-2.092002	-0.190848
40	1	0	-5.648901	-1.940375	-0.743990
41	8	0	-5.224882	0.132487	-0.074529

E(RB3LYP/6-31G(d,p)) = -1688.61243558 Ha

ΔG (298.15 K, 1 atm, B3LYP/6-31G(d,p)) = -1688.360565 Ha

E(RB3LYP/6-311++G(d,p)//B3LYP/6-31G(d,p)) = -1688.94704653 Ha

ΔG (298.15 K, 1 atm, B3LYP/6-311++G(d,p)//B3LYP/6-31G(d,p)) = -1688.695177 Ha

6a-$KHCO_3$ – conformation 16 (related to conformation 4)

Center Number	Atomic Number	Atomic Type	Coordinates (Angstroms) X	Y	Z
1	6	0	1.280945	-1.608156	-1.797730
2	6	0	-0.510583	-0.180012	-0.980768
3	6	0	0.297216	0.926879	-0.913063
4	6	0	1.870713	-0.320367	-2.349829
5	1	0	1.302945	-2.428930	-2.515641
6	1	0	-1.568198	-0.116490	-0.740360
7	1	0	2.906126	-0.501835	-2.649394
8	7	0	-0.083465	-1.410646	-1.363648
9	6	0	-0.942981	-2.587467	-1.242601
10	1	0	-0.562355	-3.264120	-0.469581
11	1	0	-1.949535	-2.262418	-0.973314
12	1	0	-0.978132	-3.128446	-2.193677
13	6	0	-0.318924	2.206516	-0.597277
14	8	0	-1.522875	2.309816	-0.287477
15	6	0	0.523020	3.464866	-0.680588
16	1	0	1.467735	3.350814	-0.142096
17	1	0	0.772277	3.676132	-1.728217
18	1	0	-0.048487	4.304603	-0.281534
19	6	0	1.778662	0.718863	-1.221123
20	1	0	2.247694	1.655516	-1.525438
21	6	0	3.639886	-0.923050	2.421481
22	6	0	3.455781	-1.860484	1.225343
23	6	0	4.154100	0.440056	1.955930
24	1	0	4.431944	-2.162868	0.819675
25	1	0	2.939924	-2.782252	1.511916
26	1	0	2.677304	-0.796838	2.931226
27	1	0	4.330264	-1.367677	3.144305
28	1	0	5.174031	0.337961	1.556590
29	1	0	4.208723	1.162399	2.775154
30	6	0	3.304591	1.053950	0.848663
31	6	0	2.670567	-1.194971	0.130873
32	8	0	2.093842	-2.104807	-0.689020
33	8	0	3.294559	2.280157	0.694012
34	6	0	2.568918	0.154502	-0.040486
35	1	0	1.302265	-0.011103	-3.231238
36	19	0	-3.994455	1.777181	0.370300
37	6	0	-4.043792	-1.194635	0.906034
38	8	0	-4.170247	-0.439968	1.894484
39	8	0	-4.213418	-2.550469	1.171601
40	1	0	-4.120780	-2.994702	0.316006
41	8	0	-3.784807	-0.887303	-0.294062

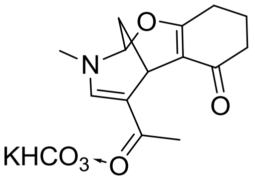

E(RB3LYP/6-31G(d,p)) = -1688.61179423 Ha

ΔG (298.15 K, 1 atm, B3LYP/6-31G(d,p)) = -1688.360876 Ha

E(RB3LYP/6-311++G(d,p)//B3LYP/6-31G(d,p)) = -1688.94671263 Ha

ΔG (298.15 K, 1 atm, B3LYP/6-311++G(d,p)//B3LYP/6-31G(d,p)) = -1688.695795 Ha

6aa-$KHCO_3$ – conformation 1

Center Number	Atomic Number	Atomic Type	Coordinates (Angstroms) X	Y	Z
1	6	0	0.763956	-0.700929	0.671870
2	6	0	2.644675	-1.610126	-0.552267
3	6	0	3.536124	-0.874494	0.202830
4	6	0	1.704436	-0.716967	1.880159
5	1	0	-0.230572	-1.071862	0.924296
6	1	0	2.991443	-2.248934	-1.358338
7	1	0	1.282850	-0.123710	2.695594
8	7	0	1.317239	-1.608814	-0.359823
9	6	0	0.404749	-2.444362	-1.132006
10	1	0	-0.054747	-3.208545	-0.494517
11	1	0	-0.386881	-1.826791	-1.562330
12	1	0	0.955692	-2.941807	-1.931747
13	6	0	4.964917	-0.855265	-0.060341
14	8	0	5.742333	-0.221128	0.668018
15	6	0	5.523600	-1.626849	-1.252006
16	1	0	5.312629	-2.698410	-1.170005
17	1	0	5.090432	-1.277069	-2.194701
18	1	0	6.604257	-1.482321	-1.282537
19	6	0	3.018606	-0.121266	1.393980
20	1	0	3.783692	-0.069942	2.166980
21	6	0	0.148409	3.555798	-0.120717
22	6	0	1.591903	3.084320	0.073168
23	6	0	-0.615750	2.590589	-1.031085
24	1	0	2.168092	3.217498	-0.853651
25	1	0	2.108957	3.663636	0.844191
26	1	0	-0.348951	3.606505	0.855088
27	1	0	0.137042	4.565855	-0.540146
28	1	0	-0.201788	2.627927	-2.049636
29	1	0	-1.674524	2.854021	-1.110343
30	6	0	-0.523141	1.145692	-0.565714
31	6	0	1.653685	1.630680	0.450508
32	8	0	2.788078	1.313830	1.075892
33	8	0	-1.428038	0.340641	-0.846404
34	6	0	0.653741	0.731656	0.174675
35	1	0	1.867685	-1.736381	2.240268
36	19	0	-3.956109	-0.008871	-1.597489
37	6	0	-5.520518	-0.736039	0.852640
38	8	0	-5.473809	0.486454	0.581481
39	8	0	-6.241938	-1.054693	2.003809
40	1	0	-6.186005	-2.019137	2.074871
41	8	0	-5.007910	-1.704469	0.230147

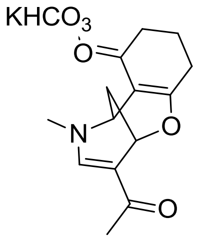

E(RB3LYP/6-31G(d,p)) = -1688.60918823 Ha

ΔG (298.15 K, 1 atm, B3LYP/6-31G(d,p)) = -1688.363301 Ha

E(RB3LYP/6-311++G(d,p)//B3LYP/6-31G(d,p)) = -1688.94945267 Ha

ΔG (298.15 K, 1 atm, B3LYP/6-311++G(d,p)//B3LYP/6-31G(d,p)) = -1688.703566 Ha

6aa-KHCO$_3$ – conformation 2

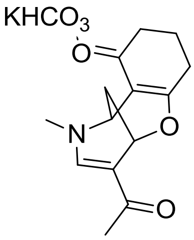

Center Number	Atomic Number	Atomic Type	Coordinates (Angstroms) X	Y	Z
1	6	0	0.974694	-0.804447	0.777994
2	6	0	3.036035	-1.507734	-0.284068
3	6	0	3.751161	-0.521720	0.365180
4	6	0	1.860196	-0.458000	1.978106
5	1	0	0.062724	-1.321850	1.079746
6	1	0	3.521794	-2.176807	-0.987143
7	1	0	1.305016	0.158368	2.689823
8	7	0	1.724505	-1.726391	-0.105158
9	6	0	0.995283	-2.778158	-0.803412
10	1	0	0.599380	-3.510875	-0.091296
11	1	0	0.158205	-2.348322	-1.359738
12	1	0	1.666185	-3.289477	-1.495261
13	6	0	5.162124	-0.277261	0.120012
14	8	0	5.778572	0.598072	0.745139
15	6	0	5.901992	-1.108224	-0.923753
16	1	0	5.874024	-2.175629	-0.680693
17	1	0	5.460959	-0.985245	-1.918355
18	1	0	6.941892	-0.780618	-0.954027
19	6	0	3.054655	0.296889	1.412727
20	1	0	3.764939	0.618283	2.172704
21	6	0	0.114782	2.818609	-1.558132
22	6	0	-1.079522	1.928478	-1.205000
23	6	0	1.069750	2.941591	-0.366792
24	1	0	-1.708570	2.425171	-0.451572
25	1	0	-1.718188	1.739054	-2.072777
26	1	0	0.654359	2.385545	-2.408628
27	1	0	-0.227509	3.810519	-1.866511
28	1	0	0.609567	3.539767	0.432450
29	1	0	1.997638	3.450351	-0.643634
30	6	0	1.413171	1.591596	0.198724
31	6	0	-0.669271	0.586208	-0.618621
32	8	0	-1.433179	-0.391010	-0.705361
33	8	0	2.574450	1.590536	0.853522
34	6	0	0.599630	0.492131	0.078503
35	1	0	2.201568	-1.360410	2.492613
36	19	0	-3.940622	-1.081780	-1.311522
37	6	0	-5.758427	-0.060694	0.841666
38	8	0	-5.675377	0.656507	-0.182484
39	8	0	-6.602384	0.433389	1.837031
40	1	0	-6.560776	-0.222545	2.548624
41	8	0	-5.187137	-1.156531	1.088387

E(RB3LYP/6-31G(d,p)) = -1688.60891131 Ha

ΔG (298.15 K, 1 atm, B3LYP/6-31G(d,p)) = -1688.362519 Ha

E(RB3LYP/6-311++G(d,p)//B3LYP/6-31G(d,p)) = -1688.94923475 Ha

ΔG (298.15 K, 1 atm, B3LYP/6-311++G(d,p)//B3LYP/6-31G(d,p)) = -1688.702843 Ha

6aa-KHCO$_3$ – conformation 3

Center Number	Atomic Number	Atomic Type	Coordinates (Angstroms) X	Y	Z
1	6	0	0.661552	-0.708629	0.581031
2	6	0	2.451110	-1.653192	-0.734693
3	6	0	3.406729	-1.043330	0.055347
4	6	0	1.607987	-0.894575	1.769090
5	1	0	-0.357422	-1.016306	0.820465
6	1	0	2.777265	-2.235469	-1.591286
7	1	0	1.242578	-0.338715	2.636151
8	7	0	1.132440	-1.576731	-0.523466
9	6	0	0.154076	-2.284668	-1.341352
10	1	0	-0.357138	-3.055149	-0.752891
11	1	0	-0.591537	-1.580726	-1.717905
12	1	0	0.662276	-2.763721	-2.179541
13	6	0	4.802494	-1.138290	-0.353293
14	8	0	5.157665	-1.729234	-1.383725
15	6	0	5.853701	-0.478803	0.530477
16	1	0	5.588627	0.556681	0.766455
17	1	0	5.952561	-1.014649	1.482074
18	1	0	6.815348	-0.502301	0.016045
19	6	0	2.962635	-0.360866	1.316344
20	1	0	3.708228	-0.430568	2.107737
21	6	0	0.376231	3.632934	0.115509
22	6	0	1.778786	3.039081	0.268956
23	6	0	-0.457600	2.799385	-0.861317
24	1	0	2.366703	3.197846	-0.646414
25	1	0	2.336077	3.516908	1.080498
26	1	0	-0.117926	3.649203	1.094101
27	1	0	0.443544	4.669160	-0.227877
28	1	0	-0.039150	2.879604	-1.875526
29	1	0	-1.492837	3.147987	-0.917669
30	6	0	-0.477436	1.321612	-0.504001
31	6	0	1.729341	1.560554	0.533945
32	8	0	2.837214	1.112732	1.129593
33	8	0	-1.442264	0.612215	-0.836165
34	6	0	0.665096	0.762876	0.195916
35	1	0	1.695982	-1.949265	2.043219
36	19	0	-4.011043	0.401887	-1.489698
37	6	0	-5.360708	-0.905116	0.845422
38	8	0	-5.280598	0.344385	0.896114
39	8	0	-5.967322	-1.500144	1.952226
40	1	0	-5.953871	-2.450241	1.763883
41	8	0	-4.969762	-1.686558	-0.062701

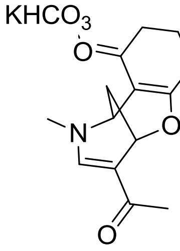

E(RB3LYP/6-31G(d,p)) = -1688.60835074 Ha

ΔG (298.15 K, 1 atm, B3LYP/6-31G(d,p)) = -1688.361835 Ha

E(RB3LYP/6-311++G(d,p)//B3LYP/6-31G(d,p)) = -1688.94779883 Ha

ΔG (298.15 K, 1 atm, B3LYP/6-311++G(d,p)//B3LYP/6-31G(d,p)) = -1688.701283 Ha

6aa-KHCO$_3$ – conformation 4

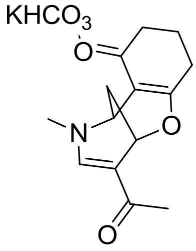

Center Number	Atomic Number	Atomic Type	Coordinates (Angstroms) X	Y	Z
1	6	0	0.762306	-0.854010	0.482963
2	6	0	2.725148	-1.558214	-0.736059
3	6	0	3.560937	-0.895906	0.142377
4	6	0	1.645697	-0.982559	1.725775
5	1	0	-0.229380	-1.279749	0.642907
6	1	0	3.164268	-2.067298	-1.588772
7	1	0	1.166537	-0.499353	2.580906
8	7	0	1.394120	-1.618930	-0.616332
9	6	0	0.543883	-2.341410	-1.554985
10	1	0	0.037504	-3.174439	-1.054584
11	1	0	-0.214797	-1.669677	-1.964582
12	1	0	1.155421	-2.738611	-2.366308
13	6	0	4.984571	-0.845851	-0.165291
14	8	0	5.466169	-1.366839	-1.182032
15	6	0	5.903388	-0.119786	0.809190
16	1	0	5.526482	0.879507	1.049236
17	1	0	5.984366	-0.672869	1.752575
18	1	0	6.897025	-0.039405	0.366277
19	6	0	2.962520	-0.296746	1.381384
20	1	0	3.655187	-0.306856	2.222168
21	6	0	0.560731	3.345462	-0.804650
22	6	0	-0.767292	2.590558	-0.705984
23	6	0	1.467957	3.001163	0.380525
24	1	0	-1.346727	2.960910	0.152656
25	1	0	-1.389107	2.738830	-1.593629
26	1	0	1.067887	3.072311	-1.737474
27	1	0	0.384202	4.424239	-0.838901
28	1	0	1.066151	3.432830	1.308201
29	1	0	2.472890	3.413822	0.252956
30	6	0	1.580911	1.513741	0.567516
31	6	0	-0.586526	1.094579	-0.500470
32	8	0	-1.479255	0.304731	-0.852620
33	8	0	2.699012	1.161051	1.205409
34	6	0	0.620536	0.624122	0.154671
35	1	0	1.828479	-2.031029	1.975975
36	19	0	-4.032578	0.031765	-1.560206
37	6	0	-5.540942	-0.658264	0.935747
38	8	0	-5.480255	0.562222	0.658311
39	8	0	-6.225376	-0.958964	2.114142
40	1	0	-6.185514	-1.924059	2.187052
41	8	0	-5.070755	-1.638624	0.298706

E(RB3LYP/6-31G(d,p)) = -1688.60805948 Ha

ΔG (298.15 K, 1 atm, B3LYP/6-31G(d,p)) = -1688.362265 Ha

E(RB3LYP/6-311++G(d,p)//B3LYP/6-31G(d,p)) = -1688.94755392 Ha

ΔG (298.15 K, 1 atm, B3LYP/6-311++G(d,p)//B3LYP/6-31G(d,p)) = -1688.701760 Ha

6aa-$KHCO_3$ – conformation 5 (related to conformation 1)

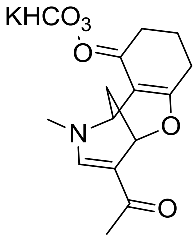

Center Number	Atomic Number	Atomic Type	Coordinates (Angstroms) X	Y	Z
1	6	0	1.364172	0.033969	1.531795
2	6	0	2.400388	-1.733204	0.241070
3	6	0	3.475335	-0.924075	-0.068166
4	6	0	2.795435	0.425761	1.909350
5	1	0	0.694868	0.054793	2.393190
6	1	0	2.333517	-2.744382	-0.147704
7	1	0	2.808223	1.419361	2.364615
8	7	0	1.381106	-1.359342	1.028801
9	6	0	0.290112	-2.257197	1.390245
10	1	0	0.320434	-2.493830	2.460212
11	1	0	-0.667790	-1.783615	1.163867
12	1	0	0.380718	-3.185774	0.824538
13	6	0	4.539225	-1.333824	-0.968813
14	8	0	5.511177	-0.596335	-1.188977
15	6	0	4.463877	-2.692396	-1.658544
16	1	0	4.433697	-3.510464	-0.931072
17	1	0	3.568901	-2.774573	-2.283841
18	1	0	5.347888	-2.811783	-2.286260
19	6	0	3.588641	0.410563	0.609728
20	1	0	4.636101	0.678447	0.738460
21	6	0	-0.005948	3.346217	-0.976784
22	6	0	1.363713	2.744625	-1.301267
23	6	0	-1.031447	2.240735	-0.710697
24	1	0	1.351170	2.271575	-2.293592
25	1	0	2.146380	3.508739	-1.333404
26	1	0	0.081097	3.983255	-0.088668
27	1	0	-0.339087	3.985484	-1.799350
28	1	0	-1.223071	1.674935	-1.634403
29	1	0	-1.993183	2.646479	-0.383663
30	6	0	-0.561836	1.240467	0.334122
31	6	0	1.761743	1.699321	-0.297279
32	8	0	3.081070	1.508265	-0.256692
33	8	0	-1.391241	0.614898	1.017392
34	6	0	0.863764	1.015419	0.483839
35	1	0	3.226511	-0.286942	2.617709
36	19	0	-4.016078	0.238090	1.385901
37	6	0	-5.408530	-1.036265	-0.941215
38	8	0	-4.761509	-1.815431	-0.203328
39	8	0	-6.063044	-1.648496	-2.010783
40	1	0	-6.509321	-0.926345	-2.477312
41	8	0	-5.545352	0.212803	-0.845281

E(RB3LYP/6-31G(d,p)) = -1688.60924048 Ha

ΔG (298.15 K, 1 atm, B3LYP/6-31G(d,p)) = -1688.364026 Ha

E(RB3LYP/6-311++G(d,p)//B3LYP/6-31G(d,p)) = -1688.94944807 Ha

ΔG (298.15 K, 1 atm, B3LYP/6-311++G(d,p)//B3LYP/6-31G(d,p)) = -1688.704233 Ha

6aa-KHCO$_3$ – isomer 6 (related to conformation 1)

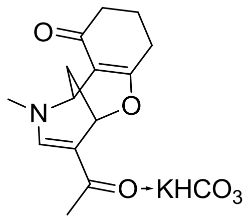

Center Number	Atomic Number	Atomic Type	Coordinates (Angstroms) X	Y	Z
1	6	0	2.030745	0.630370	1.355846
2	6	0	1.233839	2.330844	-0.165357
3	6	0	0.029712	1.664573	-0.319021
4	6	0	0.583735	0.561576	1.845909
5	1	0	2.747095	0.614841	2.178362
6	1	0	1.444630	3.239160	-0.721000
7	1	0	0.428874	-0.330136	2.458583
8	7	0	2.213371	1.920234	0.646934
9	6	0	3.447084	2.673085	0.848789
10	1	0	3.482492	3.093548	1.860282
11	1	0	4.302727	2.008382	0.713662
12	1	0	3.496478	3.490436	0.127587
13	6	0	-0.940542	2.036700	-1.322612
14	8	0	-1.975433	1.362814	-1.496457
15	6	0	-0.704259	3.251697	-2.209515
16	1	0	-0.469479	4.147060	-1.626134
17	1	0	0.130234	3.075445	-2.896851
18	1	0	-1.606153	3.433671	-2.795360
19	6	0	-0.279808	0.510940	0.593070
20	1	0	-1.343649	0.453858	0.823532
21	6	0	2.700159	-3.184100	-0.672803
22	6	0	1.401135	-2.441187	-0.999426
23	6	0	3.887621	-2.217381	-0.662301
24	1	0	1.383598	-2.142609	-2.057633
25	1	0	0.524085	-3.077940	-0.842736
26	1	0	2.606217	-3.656853	0.312024
27	1	0	2.864805	-3.985405	-1.398978
28	1	0	4.058769	-1.824580	-1.675437
29	1	0	4.815180	-2.706488	-0.352441
30	6	0	3.660888	-1.018937	0.249689
31	6	0	1.245118	-1.202153	-0.163532
32	8	0	-0.030923	-0.792320	-0.084380
33	8	0	4.618176	-0.440371	0.775429
34	6	0	2.287555	-0.560009	0.445903
35	1	0	0.325948	1.441434	2.442037
36	19	0	-2.670736	-1.232178	-1.293159
37	6	0	-4.411233	-0.571259	1.072776
38	8	0	-4.971872	-0.515843	-0.044807
39	8	0	-5.185544	-0.124407	2.142898
40	1	0	-4.620586	-0.229288	2.922746
41	8	0	-3.248324	-0.972791	1.355412

E(RB3LYP/6-31G(d,p)) = -1688.61440676 Ha

ΔG (298.15 K, 1 atm, B3LYP/6-31G(d,p)) = -1688.363561 Ha

E(RB3LYP/6-311++G(d,p)//B3LYP/6-31G(d,p)) = -1688.95073758 Ha

ΔG (298.15 K, 1 atm, B3LYP/6-311++G(d,p)//B3LYP/6-31G(d,p)) = -1688.699892 Ha

$\mathbf{6aa\text{-}KHCO_3}$ – isomer 7 (related to conformation 1)

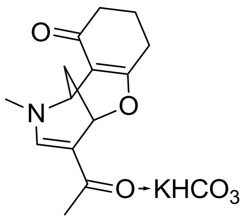

Center Number	Atomic Number	Atomic Type	Coordinates (Angstroms) X	Y	Z
1	6	0	2.834749	0.094443	-0.629317
2	6	0	2.135707	-1.693904	0.836976
3	6	0	1.057713	-1.978733	0.014817
4	6	0	2.349599	-0.809316	-1.764333
5	1	0	3.792574	0.564213	-0.857170
6	1	0	2.291020	-2.239887	1.762058
7	1	0	2.278004	-0.244960	-2.697476
8	7	0	3.042490	-0.749169	0.573001
9	6	0	4.208287	-0.507217	1.417460
10	1	0	5.127008	-0.809439	0.902135
11	1	0	4.269524	0.557184	1.652308
12	1	0	4.116209	-1.084475	2.338694
13	6	0	0.024353	-2.916333	0.386296
14	8	0	-0.967476	-3.100696	-0.349560
15	6	0	0.114827	-3.684548	1.697478
16	1	0	1.072783	-4.202128	1.805325
17	1	0	0.004832	-3.008252	2.552030
18	1	0	-0.692170	-4.417745	1.726581
19	6	0	0.980227	-1.315726	-1.331738
20	1	0	0.526131	-1.987241	-2.059450
21	6	0	0.027931	3.447832	-0.568074
22	6	0	-0.566582	2.044110	-0.720667
23	6	0	1.118176	3.466541	0.507118
24	1	0	-1.229688	1.788231	0.119102
25	1	0	-1.182657	1.975101	-1.624685
26	1	0	0.459530	3.764161	-1.525370
27	1	0	-0.763120	4.162370	-0.322401
28	1	0	0.676375	3.254826	1.491931
29	1	0	1.607886	4.441344	0.581600
30	6	0	2.192820	2.416504	0.263010
31	6	0	0.500179	0.991319	-0.803911
32	8	0	0.043320	-0.160748	-1.329502
33	8	0	3.353385	2.600997	0.645928
34	6	0	1.791659	1.174252	-0.394040
35	1	0	3.029438	-1.651810	-1.918079
36	19	0	-2.707418	-1.176736	-1.053407
37	6	0	-4.030385	0.800265	0.803813
38	8	0	-2.892046	0.405955	1.158124
39	8	0	-4.605451	1.754377	1.640922
40	1	0	-5.466199	1.952487	1.242949
41	8	0	-4.707923	0.451520	-0.197873

E(RB3LYP/6-31G(d,p)) = -1688.61534010 Ha

ΔG (298.15 K, 1 atm, B3LYP/6-31G(d,p)) = -1688.364229 Ha

E(RB3LYP/6-311++G(d,p)//B3LYP/6-31G(d,p)) = -1688.95072049 Ha

ΔG (298.15 K, 1 atm, B3LYP/6-311++G(d,p)//B3LYP/6-31G(d,p)) = -1688.699609 Ha

6aa-$KHCO_3$ – conformation 8 (related to conformation 2)

Center Number	Atomic Number	Atomic Type	Coordinates (Angstroms) X	Y	Z
1	6	0	-1.334384	-0.839110	-1.375917
2	6	0	-2.613959	-1.477876	0.580528
3	6	0	-3.512390	-0.450139	0.376281
4	6	0	-2.685333	-0.428167	-1.967866
5	1	0	-0.724291	-1.388659	-2.094474
6	1	0	-2.710896	-2.140823	1.434283
7	1	0	-2.533727	0.180149	-2.863249
8	7	0	-1.582208	-1.747825	-0.233435
9	6	0	-0.644278	-2.835982	0.014500
10	1	0	-0.676821	-3.565678	-0.802246
11	1	0	0.374031	-2.445345	0.089663
12	1	0	-0.908723	-3.340180	0.945087
13	6	0	-4.600110	-0.158864	1.294169
14	8	0	-5.410640	0.749683	1.060238
15	6	0	-4.753555	-0.982722	2.569128
16	1	0	-4.912089	-2.042868	2.344567
17	1	0	-3.864060	-0.909651	3.203305
18	1	0	-5.615567	-0.607122	3.121946
19	6	0	-3.396034	0.363916	-0.879622
20	1	0	-4.375168	0.732629	-1.180866
21	6	0	0.759040	2.684240	0.204480
22	6	0	1.570820	1.756647	-0.703821
23	6	0	-0.659052	2.877923	-0.340838
24	1	0	1.746528	2.247162	-1.673514
25	1	0	2.555112	1.526271	-0.283307
26	1	0	0.701284	2.251399	1.210411
27	1	0	1.257135	3.653323	0.301299
28	1	0	-0.635843	3.476999	-1.262381
29	1	0	-1.295533	3.417200	0.366808
30	6	0	-1.305338	1.558003	-0.657345
31	6	0	0.855851	0.448661	-1.004219
32	8	0	1.506787	-0.563264	-1.319972
33	8	0	-2.637856	1.621677	-0.638063
34	6	0	-0.595895	0.423915	-0.963819
35	1	0	-3.282418	-1.303013	-2.239491
36	19	0	4.061194	-1.376879	-1.106361
37	6	0	4.770040	0.119716	1.398432
38	8	0	4.532822	-1.101109	1.542174
39	8	0	4.996723	0.819669	2.581987
40	1	0	5.160890	1.733620	2.306000
41	8	0	4.827212	0.793231	0.331965

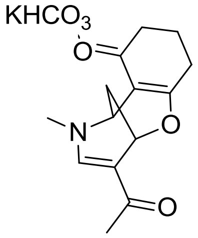

E(RB3LYP/6-31G(d,p)) = -1688.61067237 Ha

ΔG (298.15 K, 1 atm, B3LYP/6-31G(d,p)) = -1688.363445 Ha

E(RB3LYP/6-311++G(d,p)//B3LYP/6-31G(d,p)) = -1688.94893626 Ha

ΔG (298.15 K, 1 atm, B3LYP/6-311++G(d,p)//B3LYP/6-31G(d,p)) = -1688.701709 Ha

6aa-KHCO$_3$ – isomer 9 (related to conformation 2)

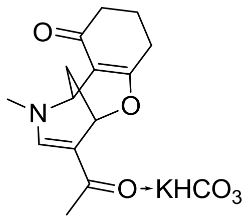

Center Number	Atomic Number	Atomic Type	X	Y	Z
1	6	0	2.829005	-0.229287	-0.600791
2	6	0	1.868118	-1.903986	0.857577
3	6	0	0.765329	-2.033645	0.029754
4	6	0	2.204018	-1.038173	-1.738176
5	1	0	3.849784	0.080949	-0.828080
6	1	0	1.941720	-2.469749	1.780818
7	1	0	2.216550	-0.459661	-2.665274
8	7	0	2.899721	-1.094525	0.601321
9	6	0	4.065771	-0.992455	1.472987
10	1	0	4.953918	-1.400194	0.977403
11	1	0	4.250565	0.056943	1.712783
12	1	0	3.885751	-1.554146	2.390736
13	6	0	-0.382061	-2.837451	0.379155
14	8	0	-1.380668	-2.886623	-0.368511
15	6	0	-0.400565	-3.631251	1.677798
16	1	0	0.472105	-4.285013	1.769301
17	1	0	-0.405815	-2.961552	2.544419
18	1	0	-1.305546	-4.239545	1.700502
19	6	0	0.777598	-1.335455	-1.299948
20	1	0	0.220606	-1.912913	-2.036704
21	6	0	0.295691	3.221209	0.389378
22	6	0	1.791360	3.491747	0.209155
23	6	0	-0.215819	2.236822	-0.668479
24	1	0	1.961114	4.030919	-0.734702
25	1	0	2.198309	4.117959	1.007970
26	1	0	0.118144	2.800424	1.386566
27	1	0	-0.271140	4.155030	0.331303
28	1	0	-0.214630	2.714019	-1.659992
29	1	0	-1.250362	1.938525	-0.467524
30	6	0	0.659337	1.017708	-0.744182
31	6	0	2.618357	2.215287	0.142528
32	8	0	3.807691	2.219634	0.479036
33	8	0	0.020598	-0.054333	-1.247874
34	6	0	1.976867	1.008116	-0.375359
35	1	0	2.743545	-1.974512	-1.905394
36	19	0	-2.864439	-0.856619	-1.294849
37	6	0	-3.952204	1.091885	0.725240
38	8	0	-3.581790	1.592221	-0.365659
39	8	0	-4.387266	2.014529	1.674314
40	1	0	-4.638965	1.483605	2.444566
41	8	0	-3.979088	-0.119188	1.066740

E(RB3LYP/6-31G(d,p)) = -1688.61509778 Ha

ΔG (298.15 K, 1 atm, B3LYP/6-31G(d,p)) = -1688.364951 Ha

E(RB3LYP/6-311++G(d,p)//B3LYP/6-31G(d,p)) = -1688.95068868 Ha

ΔG (298.15 K, 1 atm, B3LYP/6-311++G(d,p)//B3LYP/6-31G(d,p)) = -1688.700541 Ha

6aa-KHCO$_3$ – isomer 10 (related to conformation 2)

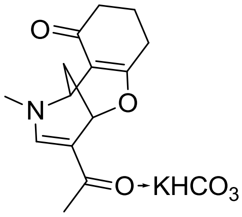

Center Number	Atomic Number	Atomic Type	Coordinates (Angstroms) X	Y	Z
1	6	0	2.017526	0.633565	1.367290
2	6	0	1.229711	2.322960	-0.174115
3	6	0	0.019383	1.666478	-0.320551
4	6	0	0.566590	0.568512	1.845643
5	1	0	2.726538	0.631428	2.196292
6	1	0	1.447551	3.224364	-0.738065
7	1	0	0.409222	-0.318437	2.464562
8	7	0	2.206979	1.911557	0.640396
9	6	0	3.461722	2.637293	0.806973
10	1	0	3.530050	3.064474	1.813828
11	1	0	4.300367	1.953082	0.659976
12	1	0	3.514587	3.446943	0.077552
13	6	0	-0.954074	2.047487	-1.317381
14	8	0	-1.994713	1.381212	-1.487070
15	6	0	-0.715635	3.264085	-2.201587
16	1	0	-0.473507	4.156365	-1.616550
17	1	0	0.114605	3.085615	-2.893507
18	1	0	-1.619301	3.452470	-2.782641
19	6	0	-0.287765	0.509569	0.587810
20	1	0	-1.352336	0.443327	0.811652
21	6	0	2.843365	-2.623681	-1.468645
22	6	0	3.861796	-2.409200	-0.345809
23	6	0	1.411905	-2.475260	-0.941565
24	1	0	3.781501	-3.222069	0.390943
25	1	0	4.890536	-2.418970	-0.716121
26	1	0	3.012371	-1.884799	-2.261032
27	1	0	2.973217	-3.612008	-1.919011
28	1	0	1.152714	-3.324448	-0.293127
29	1	0	0.685488	-2.473546	-1.760258
30	6	0	1.249075	-1.210065	-0.145704
31	6	0	3.644006	-1.104530	0.409032
32	8	0	4.589150	-0.539607	0.970211
33	8	0	-0.024203	-0.787839	-0.098151
34	6	0	2.283452	-0.576655	0.487084
35	1	0	0.302154	1.452416	2.432868
36	19	0	-2.696125	-1.208315	-1.307896
37	6	0	-4.401031	-0.614705	1.100261
38	8	0	-4.980579	-0.538559	-0.006422
39	8	0	-5.161804	-0.201092	2.193086
40	1	0	-4.583180	-0.318917	2.960986
41	8	0	-3.229752	-1.011033	1.354156

E(RB3LYP/6-31G(d,p)) = -1688.61401496 Ha

ΔG (298.15 K, 1 atm, B3LYP/6-31G(d,p)) = -1688.363750 Ha

E(RB3LYP/6-311++G(d,p)//B3LYP/6-31G(d,p)) = -1688.95037792 Ha

ΔG (298.15 K, 1 atm, B3LYP/6-311++G(d,p)//B3LYP/6-31G(d,p)) = -1688.700113 Ha

6aa-$KHCO_3$ – conformation 11 (related to conformation 3)

Center Number	Atomic Number	Atomic Type	Coordinates (Angstroms) X	Y	Z
1	6	0	0.857692	0.122571	1.472430
2	6	0	1.620302	-1.801003	0.232214
3	6	0	2.888538	-1.265757	0.106004
4	6	0	2.268967	0.140400	2.064616
5	1	0	0.093538	0.289406	2.232927
6	1	0	1.408589	-2.755908	-0.239642
7	1	0	2.453603	1.086559	2.579560
8	7	0	0.615603	-1.221987	0.898079
9	6	0	-0.677948	-1.867657	1.098933
10	1	0	-0.819368	-2.124513	2.155531
11	1	0	-1.493532	-1.211750	0.790191
12	1	0	-0.714988	-2.786114	0.510966
13	6	0	3.850524	-1.961842	-0.738072
14	8	0	3.569364	-2.999826	-1.355559
15	6	0	5.252966	-1.377989	-0.858004
16	1	0	5.225422	-0.309248	-1.092732
17	1	0	5.802571	-1.490741	0.084216
18	1	0	5.793449	-1.910965	-1.641535
19	6	0	3.222207	-0.027768	0.886056
20	1	0	4.263857	-0.009478	1.205274
21	6	0	0.691435	3.725435	-1.011339
22	6	0	1.922567	2.828494	-1.162711
23	6	0	-0.586061	2.882578	-0.963715
24	1	0	1.961884	2.396251	-2.172801
25	1	0	2.852880	3.389492	-1.030757
26	1	0	0.779109	4.306089	-0.085439
27	1	0	0.645901	4.441706	-1.836645
28	1	0	-0.746976	2.390025	-1.934107
29	1	0	-1.472303	3.492443	-0.766530
30	6	0	-0.525169	1.786305	0.088344
31	6	0	1.908953	1.693426	-0.177629
32	8	0	3.128951	1.196038	0.041108
33	8	0	-1.567723	1.372762	0.621863
34	6	0	0.769830	1.220684	0.423387
35	1	0	2.409623	-0.677053	2.776875
36	19	0	-4.219946	1.148490	0.479007
37	6	0	-4.330049	-1.656936	-0.578099
38	8	0	-4.054893	-1.549188	0.641090
39	8	0	-4.322209	-2.962680	-1.067384
40	1	0	-4.550773	-2.880661	-2.005183
41	8	0	-4.607400	-0.750679	-1.407660

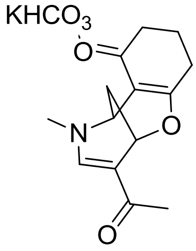

E(RB3LYP/6-31G(d,p)) = -1688.60939123 Ha

ΔG (298.15 K, 1 atm, B3LYP/6-31G(d,p)) = -1688.361166 Ha

E(RB3LYP/6-311++G(d,p)//B3LYP/6-31G(d,p)) = -1688.94720706 Ha

ΔG (298.15 K, 1 atm, B3LYP/6-311++G(d,p)//B3LYP/6-31G(d,p)) = -1688.698982 Ha

6aa-KHCO$_3$ – isomer 12 (related to conformation 3)

Center Number	Atomic Number	Atomic Type	X	Y	Z
1	6	0	2.872743	-0.813267	1.110181
2	6	0	0.475097	-1.035065	1.270601
3	6	0	0.407435	-1.594390	0.003995
4	6	0	2.816415	-2.126947	0.328300
5	1	0	3.687833	-0.801205	1.835171
6	1	0	-0.446799	-0.865505	1.818425
7	1	0	3.768320	-2.308856	-0.176578
8	7	0	1.608607	-0.679597	1.875831
9	6	0	1.653666	-0.143629	3.232778
10	1	0	2.127823	-0.859386	3.913785
11	1	0	2.232570	0.782137	3.237434
12	1	0	0.637893	0.052426	3.579408
13	6	0	-0.896783	-1.838855	-0.572030
14	8	0	-1.955859	-1.559082	0.025852
15	6	0	-0.972970	-2.460944	-1.957406
16	1	0	-0.304235	-1.957256	-2.661643
17	1	0	-0.676143	-3.515947	-1.921708
18	1	0	-1.999625	-2.400980	-2.322018
19	6	0	1.690725	-1.962888	-0.687704
20	1	0	1.579376	-2.850635	-1.309683
21	6	0	4.055534	2.173106	-1.852393
22	6	0	2.937636	1.204302	-2.248719
23	6	0	3.837299	2.696870	-0.430027
24	1	0	1.994954	1.747577	-2.406506
25	1	0	3.159553	0.692405	-3.190447
26	1	0	5.018984	1.652348	-1.905409
27	1	0	4.100378	3.003196	-2.563466
28	1	0	2.930129	3.317986	-0.395638
29	1	0	4.664154	3.326843	-0.090706
30	6	0	3.652166	1.577888	0.586504
31	6	0	2.702967	0.165628	-1.188015
32	8	0	2.101641	-0.930532	-1.671736
33	8	0	3.966795	1.747210	1.770143
34	6	0	3.061897	0.325632	0.122612
35	1	0	2.604677	-2.972686	0.988085
36	19	0	-4.537685	-1.133275	0.205112
37	6	0	-5.222427	1.776409	-0.039932
38	8	0	-5.190934	1.133182	-1.114729
39	8	0	-5.545263	3.127681	-0.174267
40	1	0	-5.528522	3.479534	0.728084
41	8	0	-5.004372	1.369579	1.132613

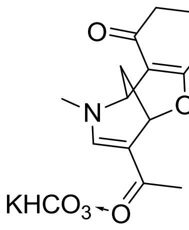

E(RB3LYP/6-31G(d,p)) = -1688.60965227 Ha

ΔG (298.15 K, 1 atm, B3LYP/6-31G(d,p)) = -1688.360819 Ha

E(RB3LYP/6-311++G(d,p)//B3LYP/6-31G(d,p)) = -1688.94864138 Ha

ΔG (298.15 K, 1 atm, B3LYP/6-311++G(d,p)//B3LYP/6-31G(d,p)) = -1688.699808 Ha

6aa-$KHCO_3$ – isomer 13 (related to conformation 3)

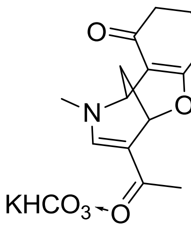

Center Number	Atomic Number	Atomic Type	X	Y	Z
1	6	0	2.568872	1.229886	1.060222
2	6	0	0.346016	1.020544	0.144808
3	6	0	0.217436	-0.259891	0.660478
4	6	0	2.029254	0.406419	2.231095
5	1	0	3.180285	2.068943	1.395077
6	1	0	-0.463914	1.428804	-0.452084
7	1	0	2.852680	0.044849	2.851845
8	7	0	1.411449	1.800906	0.327846
9	6	0	1.492713	3.169814	-0.172025
10	1	0	1.473820	3.884680	0.658379
11	1	0	2.425108	3.298566	-0.725430
12	1	0	0.642494	3.368166	-0.826195
13	6	0	-0.951218	-1.032588	0.300086
14	8	0	-1.832217	-0.594330	-0.467589
15	6	0	-1.106551	-2.428835	0.882097
16	1	0	-0.185079	-3.010737	0.786578
17	1	0	-1.350312	-2.374022	1.949797
18	1	0	-1.919072	-2.944064	0.367271
19	6	0	1.271303	-0.761530	1.608577
20	1	0	0.855814	-1.421199	2.369787
21	6	0	5.421510	-1.421389	-0.920660
22	6	0	4.056255	-2.013825	-0.559306
23	6	0	5.259551	-0.032681	-1.545366
24	1	0	3.505924	-2.295747	-1.468360
25	1	0	4.155366	-2.926746	0.036417
26	1	0	6.031928	-1.343918	-0.013184
27	1	0	5.949797	-2.090530	-1.606130
28	1	0	4.747374	-0.116540	-2.515215
29	1	0	6.222471	0.448525	-1.737635
30	6	0	4.429578	0.907507	-0.681078
31	6	0	3.213506	-1.034938	0.209259
32	8	0	2.253891	-1.639080	0.924323
33	8	0	4.604996	2.130057	-0.737144
34	6	0	3.405933	0.319339	0.178036
35	1	0	1.355750	0.999086	2.856006
36	19	0	-4.209228	-0.766797	-1.578157
37	6	0	-6.335709	0.525693	0.095739
38	8	0	-5.732415	1.284321	-0.698305
39	8	0	-7.324594	1.145126	0.861663
40	1	0	-7.690839	0.439361	1.414906
41	8	0	-6.170300	-0.708075	0.291612

E(RB3LYP/6-31G(d,p)) = -1688.60970454 Ha

ΔG (298.15 K, 1 atm, B3LYP/6-31G(d,p)) = -1688.363036 Ha

E(RB3LYP/6-311++G(d,p)//B3LYP/6-31G(d,p)) = -1688.94869112 Ha

ΔG (298.15 K, 1 atm, B3LYP/6-311++G(d,p)//B3LYP/6-31G(d,p)) = -1688.702022 Ha

6aa-$KHCO_3$ – conformation 14 (related to conformation 4)

Center Number	Atomic Number	Atomic Type	X	Y	Z
1	6	0	-1.323690	-1.010258	-1.261703
2	6	0	-2.583942	-1.489347	0.743498
3	6	0	-3.502212	-0.497976	0.456491
4	6	0	-2.683645	-0.684986	-1.883051
5	1	0	-0.702311	-1.606741	-1.931364
6	1	0	-2.692561	-2.047081	1.668741
7	1	0	-2.548317	-0.158625	-2.831206
8	7	0	-1.552589	-1.819155	-0.042439
9	6	0	-0.597142	-2.865668	0.300546
10	1	0	-0.619927	-3.666985	-0.446300
11	1	0	0.414846	-2.453762	0.336347
12	1	0	-0.853304	-3.285426	1.274229
13	6	0	-4.527847	-0.199890	1.447918
14	8	0	-4.605649	-0.801201	2.529390
15	6	0	-5.535134	0.897826	1.129051
16	1	0	-5.038666	1.816421	0.800462
17	1	0	-6.208541	0.584989	0.322164
18	1	0	-6.130581	1.102161	2.019980
19	6	0	-3.411914	0.193545	-0.872392
20	1	0	-4.387066	0.509548	-1.241432
21	6	0	0.692890	2.682374	0.001522
22	6	0	1.528132	1.691536	-0.813736
23	6	0	-0.723764	2.800201	-0.568741
24	1	0	1.706288	2.096632	-1.821656
25	1	0	2.511652	1.515440	-0.366048
26	1	0	0.633987	2.341122	1.041985
27	1	0	1.173115	3.665110	0.013545
28	1	0	-0.703591	3.313092	-1.540954
29	1	0	-1.375054	3.390188	0.082750
30	6	0	-1.344635	1.445520	-0.766566
31	6	0	0.837920	0.350131	-1.003007
32	8	0	1.507971	-0.672631	-1.230015
33	8	0	-2.679682	1.487702	-0.761416
34	6	0	-0.614024	0.301127	-0.964059
35	1	0	-3.263132	-1.593278	-2.069034
36	19	0	4.105320	-1.356826	-1.061061
37	6	0	4.812530	0.265225	1.364360
38	8	0	4.627632	-0.957578	1.559441
39	8	0	5.035612	1.018394	2.515590
40	1	0	5.157822	1.926786	2.202093
41	8	0	4.820523	0.898815	0.272277

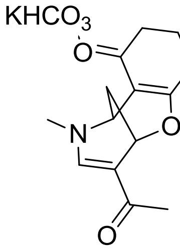

E(RB3LYP/6-31G(d,p)) = -1688.60976617 Ha

ΔG (298.15 K, 1 atm, B3LYP/6-31G(d,p)) = -1688.360303 Ha

E(RB3LYP/6-311++G(d,p)//B3LYP/6-31G(d,p)) = -1688.94723992 Ha

ΔG (298.15 K, 1 atm, B3LYP/6-311++G(d,p)//B3LYP/6-31G(d,p)) = -1688.697777 Ha

6aa-KHCO$_3$ – isomer 15 (related to conformation 4)

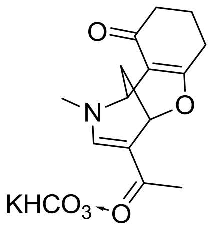

Center Number	Atomic Number	Atomic Type	Coordinates (Angstroms) X	Y	Z
1	6	0	2.994614	-1.078781	0.726853
2	6	0	0.609967	-1.328021	1.013429
3	6	0	0.429388	-1.528382	-0.346256
4	6	0	2.843176	-2.122948	-0.380867
5	1	0	3.860572	-1.275181	1.360588
6	1	0	-0.261909	-1.308618	1.660123
7	1	0	3.749107	-2.159970	-0.990807
8	7	0	1.795495	-1.149797	1.596882
9	6	0	1.956935	-0.966909	3.035641
10	1	0	2.475599	-1.825466	3.476714
11	1	0	2.547370	-0.067627	3.224684
12	1	0	0.975780	-0.869457	3.502466
13	6	0	-0.921652	-1.613673	-0.856074
14	8	0	-1.922967	-1.513695	-0.118070
15	6	0	-1.122611	-1.834755	-2.346931
16	1	0	-0.507730	-1.152482	-2.941370
17	1	0	-0.839873	-2.857020	-2.625267
18	1	0	-2.175157	-1.686226	-2.592996
19	6	0	1.646559	-1.680925	-1.215740
20	1	0	1.470194	-2.355119	-2.053321
21	6	0	3.100237	2.965807	-0.984971
22	6	0	4.176168	2.628724	0.050993
23	6	0	2.876485	1.788097	-1.939475
24	1	0	5.151848	2.521820	-0.445627
25	1	0	4.288120	3.417693	0.799756
26	1	0	2.158886	3.196315	-0.472276
27	1	0	3.382251	3.857248	-1.552791
28	1	0	3.744807	1.662006	-2.601755
29	1	0	2.010852	1.954390	-2.587507
30	6	0	2.670963	0.504487	-1.184020
31	6	0	3.905712	1.319898	0.782088
32	8	0	4.348893	1.135353	1.921370
33	8	0	1.991036	-0.405695	-1.894910
34	6	0	3.153604	0.285303	0.077667
35	1	0	2.661133	-3.118583	0.032729
36	19	0	-4.461759	-1.199181	0.456202
37	6	0	-5.378887	1.646730	0.215528
38	8	0	-5.604566	0.916254	-0.777151
39	8	0	-5.814052	2.966423	0.083592
40	1	0	-5.571594	3.394004	0.918299
41	8	0	-4.818251	1.351035	1.304564

E(RB3LYP/6-31G(d,p)) = -1688.60928039 Ha

ΔG (298.15 K, 1 atm, B3LYP/6-31G(d,p)) = -1688.363711 Ha

E(RB3LYP/6-311++G(d,p)//B3LYP/6-31G(d,p)) = -1688.94833638 Ha

ΔG (298.15 K, 1 atm, B3LYP/6-311++G(d,p)//B3LYP/6-31G(d,p)) = -1688.702766 Ha

$6aa\text{-}KHCO_3$ – isomer 16 (related to conformation 4)

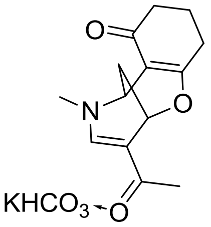

Center Number	Atomic Number	Atomic Type	X	Y	Z
1	6	0	2.513976	1.279572	1.011816
2	6	0	0.321490	0.971669	0.046892
3	6	0	0.189761	-0.270494	0.647869
4	6	0	1.952867	0.517164	2.213502
5	1	0	3.094310	2.152533	1.313951
6	1	0	-0.474202	1.326490	-0.601017
7	1	0	2.764523	0.210898	2.877980
8	7	0	1.373083	1.776034	0.204374
9	6	0	1.471269	3.091505	-0.419718
10	1	0	1.432458	3.881219	0.338850
11	1	0	2.418962	3.169978	-0.957094
12	1	0	0.640639	3.227809	-1.113613
13	6	0	-0.963854	-1.077061	0.314884
14	8	0	-1.836271	-0.695899	-0.492082
15	6	0	-1.113588	-2.438361	0.975732
16	1	0	-0.185063	-3.014864	0.926722
17	1	0	-1.372966	-2.324214	2.034995
18	1	0	-1.913390	-2.991366	0.480626
19	6	0	1.232868	-0.699142	1.642126
20	1	0	0.815544	-1.325529	2.429901
21	6	0	4.833852	-1.417886	-1.565425
22	6	0	5.485969	-0.080101	-1.205532
23	6	0	4.124443	-2.021539	-0.348621
24	1	0	6.307670	-0.245260	-0.493089
25	1	0	5.920069	0.414424	-2.078861
26	1	0	4.102572	-1.263647	-2.367699
27	1	0	5.583095	-2.118491	-1.945438
28	1	0	4.860673	-2.362976	0.392874
29	1	0	3.528856	-2.897197	-0.623376
30	6	0	3.222977	-1.016191	0.312671
31	6	0	4.516835	0.892715	-0.545967
32	8	0	4.696674	2.112669	-0.633524
33	8	0	2.241008	-1.594153	1.018192
34	6	0	3.408023	0.336890	0.224740
35	1	0	1.250324	1.132048	2.782530
36	19	0	-4.226152	-0.864482	-1.573075
37	6	0	-6.231630	0.602681	0.106777
38	8	0	-5.690390	1.275052	-0.801528
39	8	0	-7.158232	1.301755	0.882065
40	1	0	-7.481195	0.655778	1.527628
41	8	0	-6.052679	-0.607330	0.409561

E(RB3LYP/6-31G(d,p)) = -1688.60932225 Ha

ΔG (298.15 K, 1 atm, B3LYP/6-31G(d,p)) = -1688.363395 Ha

E(RB3LYP/6-311++G(d,p)//B3LYP/6-31G(d,p)) = -1688.94835763 Ha

ΔG (298.15 K, 1 atm, B3LYP/6-311++G(d,p)//B3LYP/6-31G(d,p)) = -1688.702431 Ha

Comparison of conformational energies and Boltzmann weighted average Gibbs free energies

2a+

Conformation	Free energy, Ha	Relative energy, kcal/mol
1	-440.584093	0.15
2	-440.584330	0.00

Boltzmann average free energy = -440.584226 Ha

⇨ Conformation 2 has lower energy, however the difference is negligible. Because conformation 1 is very important for further research, the barrier for interconversion (Scheme S1) was calculated to be only 6.04 kcal/mol (2a+-TS- energy)

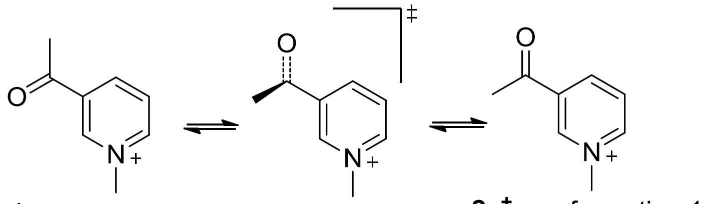

Scheme S1

2a+-TS (transition state)

Center Number	Atomic Number	Atomic Type	Coordinates (Angstroms) X	Y	Z
1	6	0	2.092379	0.837902	0.062605
2	6	0	0.400230	-0.796137	-0.043201
3	6	0	-0.587699	0.178091	-0.049493
4	6	0	-0.205179	1.521906	0.002922
5	6	0	1.149261	1.848771	0.058733
6	1	0	3.157632	1.024155	0.104665
7	1	0	0.178389	-1.854535	-0.084131
8	1	0	-0.954193	2.306548	-0.001175
9	1	0	1.478826	2.879248	0.098997
10	7	0	1.707669	-0.458693	0.012883
11	6	0	2.750045	-1.514540	0.014156
12	1	0	3.364048	-1.404140	-0.879569
13	1	0	3.362164	-1.404182	0.908956
14	1	0	2.267740	-2.488884	0.014631
15	6	0	-2.053351	-0.225873	-0.178322
16	8	0	-2.533203	-0.326444	-1.290059
17	6	0	-2.810319	-0.463024	1.098282
18	1	0	-2.336557	-1.269355	1.669945
19	1	0	-2.772311	0.432727	1.728347
20	1	0	-3.846002	-0.721747	0.875530

E(RB3LYP/6-31G(d,p)) = -440.700737162 Ha

ΔG (298.15 K, 1 atm, B3LYP/6-31G(d,p)) = -440.574704 Ha

2a-I

Conformation	Free energy, Ha	Relative energy, kcal/mol
1	-452.159073	0.09
2	-452.159213	0.00

Boltzmann average free energy (298.15 K) = -452.159148 Ha

⇨ Conformation 2 has lower energy, however the difference is negligible.

2a-HCO_3

Conformation	Free energy, Ha	Relative energy, kcal/mol
1	-705.123579	0.00
2	-705.119678	2.45

Boltzmann average free energy (298.15 K) = -705.123517 Ha

⇨ Conformation 1 has significantly lower energy

6a

Conformation	Free energy, Ha	Relative energy, kcal/mol
1	-823.945084	0.50
2	-823.944761	0.70
3	-823.945751	0.08
4	-823.945874	0.00

Boltzmann average free energy (298.15 K) = -823.945571 Ha

⇨ Conformation 4 has the lowest energy, however the difference is negligible. To build these structures, the conformation of acetyl group and that of cyclohexanone ring were changed. Interconversion energies are estimated to be ≈ 6 kcal/mol for acetyl group inversion and 3 kcal/mol for cyclohexane ring inversion (like that for normal cyclohexane).

6aa

Conformation	Free energy, Ha	Relative energy, kcal/mol
1	-823.948765	0.30
2	-823.949242	0.00
3	-823.947804	0.90
4	-823.947919	0.83

Boltzmann average free energy (298.15 K) = -823.948794 Ha

⇨ Conformation 2 has the lowest energy, however the difference is negligible. To build these structures, the conformation of the acetyl group and that of the cyclohexanone ring were changed.

6a-K, B3LYP/6-31G(d,p) level

Conformation	Free energy, Ha	Relative energy, kcal/mol
1	-1423.818632	0.05
2	-1423.818707	0.00
3	-1423.808622	6.33
4	-1423.80959	5.72
5	-1423.811672	4.41
6	-1423.811755	4.36

Boltzmann average free energy (298.15 K) = -1423.818666 Ha

⇨ Conformation 2 has the lowest energy and exists in equilibrium with conformation 1.

6aa-K, B3LYP/6-31G(d,p) level

Conformation/Isomer	Free energy, Ha	Relative energy, kcal/mol
1	-1423.81241	1.82
2	-1423.812539	1.74
3	-1423.811279	2.53
4	-1423.811618	2.31
5	-1423.815305	0.00
6	-1423.813818	0.93
7	-1423.814817	0.31
8	-1423.813133	1.36

Boltzmann average free energy (298.15 K) = -1423.814701 Ha

⇨ Conformation 5 has the lowest energy and exists in equilibrium with conformations 6 and 7

6a-K, B3LYP/6-311++G(d,p)//B3LYP/6-31G(d,p) level

Conformation	Free energy, Ha	Relative energy, kcal/mol
1	-1424.052997	0.10
2	-1424.053156	0.00
3	-1424.044559	5.39
4	-1424.045646	4.71
5	-1424.047287	3.68
6	-1424.047331	3.66

Boltzmann average free energy (298.15 K) = -1424.053068 Ha

⇨ Minimal SCF energy was computed for conformation 2: -1424.28677180 Ha

6aa-K, B3LYP/6-311++G(d,p)//B3LYP/6-31G(d,p) level

Conformation/Isomer	Free energy, Ha	Relative energy, kcal/mol
1	-1424.049608	0.57
2	-1424.049787	0.45
3	-1424.047688	1.77
4	-1424.048107	1.51
5	-1424.050509	0.00
6	-1424.049829	0.43
7	-1424.049961	0.34
8	-1424.049105	0.88

Boltzmann average free energy (298.15 K) = -1424.049904 Ha

⇨ Minimal SCF energy was computed for conformation 7: -1424.28443192 Ha

6a-KI

Conformation	Free energy, Ha	Relative energy, kcal/mol
1	-1435.391367	0.00
2	-1435.39131	0.04
3	-1435.38236	5.65
4	-1435.382155	5.78
5	-1435.382365	5.65
6	-1435.385112	3.93

Boltzmann average free energy = -1435.391334 Ha

⇨ Conformation 1 has the lowest energy and exists in equilibrium with conformation 2.

6aa-KI

Conformation/Isomer	Free energy, Ha	Relative energy, kcal/mol
1	-1435.387573	0.58
2	-1435.386392	1.33
3	-1435.384897	2.26
4	-1435.38555	1.85
5	-1435.388504	0.00
6	-1435.383663	3.04
7	-1435.387419	0.68
8	-1435.387155	0.85

Boltzmann average free energy (298.15 K) = -1435.387803 Ha

⇨ Conformation 5 has the lowest energy and exists in equilibrium with conformations 1, 7 and 8.

6a-KHCO$_3$, B3LYP/6-31G(d,p) level

Conformation	Free energy, Ha	Relative energy, kcal/mol
1	-1688.364669	1.62
2	-1688.364792	1.54
3	-1688.358868	5.26
4	-1688.357983	5.81
**5**	**-1688.366743**	**0.32**
**6**	**-1688.367247**	**0.00**
7	-1688.365117	1.34
**8**	**-1688.366497**	**0.47**
**9**	**-1688.366939**	**0.19**
10	-1688.36538	1.17
11	-1688.359171	5.07
12	-1688.361254	3.76
13	-1688.361098	3.86
14	-1688.359622	4.78
15	-1688.360565	4.19
16	-1688.360876	4.00

Boltzmann average free energy (298.15 K) = -1688.366698 Ha

⇨ Conformation 6 has the lowest energy; most-populated conformations are in bold. To build these structures, the conformation of the acetyl group, that of the cyclohexanone ring, and the HCO_3^--coordination were changed. **Most important is conformation 5, which was given by IRC calculation starting from TS 22a.**

⇨ Minimal SCF energy was computed for conformation 7: -1688.61530216 Ha

6a-KHCO$_3$, B3LYP/6-311++G(d,p)//B3LYP/6-31G(d,p) level

Conformation	Free energy, Ha	Relative energy, kcal/mol
1	-1688.700622	2.20
2	-1688.70086	2.05
3	-1688.697687	4.04
4	-1688.694791	5.86
**5**	**-1688.70359**	**0.33**
**6**	**-1688.704123**	**0.00**
7	-1688.701009	1.95
**8**	**-1688.703421**	**0.44**
**9**	**-1688.703909**	**0.13**
10	-1688.701404	1.71
11	-1688.69622	4.96
12	-1688.69997	2.61
13	-1688.699784	2.72
14	-1688.698628	3.45
15	-1688.695177	5.61
16	-1688.695795	5.23

Boltzmann average free energy (298.15 K) = -1688.703664 Ha

⇨ Minimal SCF energy was computed for conformation 9: -1688.95159980 Ha

6aa-KHCO$_3$, B3LYP/6-31G(d,p) level

Conformation/Isomer	Free energy, Ha	Relative energy, kcal/mol
1	-1688.363301	1.04
2	-1688.362519	1.53
3	-1688.361835	1.96
4	-1688.362265	1.69
**5**	**-1688.364026**	**0.58**
**6**	**-1688.363561**	**0.87**
**7**	**-1688.364229**	**0.45**
**8**	**-1688.363445**	**0.95**
**9**	**-1688.364951**	**0.00**
**10**	**-1688.36375**	**0.75**
11	-1688.361166	2.38
12	-1688.360819	2.59
13	-1688.363036	1.20
14	-1688.360303	2.92
**15**	**-1688.363711**	**0.78**
**16**	**-1688.363395**	**0.98**

Boltzmann average free energy (298.15 K) = -1688.363980 Ha

⇨ Conformation 9 has the lowest energy; most-populated conformations are in bold. To build these structures, the conformation of the acetyl group, that of the cyclohexanone ring, and the HCO_3^--coordination were changed.

⇨ Minimal SCF energy was computed for conformation 7: -1688.6153401 Ha

6aa-KHCO$_3$, B3LYP/6-311++G(d,p)//B3LYP/6-31G(d,p) level

Conformation/Isomer	Free energy, Ha	Relative energy, kcal/mol
**1**	**-1688.703566**	**0.42**
**2**	**-1688.702843**	**0.87**
3	-1688.701283	1.85
4	-1688.70176	1.55
**5**	**-1688.704233**	**0.00**
6	-1688.699892	2.72
7	-1688.699609	2.90
8	-1688.701709	1.58
9	-1688.700541	2.32
10	-1688.700113	2.59
11	-1688.698982	3.30
12	-1688.699808	2.78
13	-1688.702022	1.39
14	-1688.697777	4.05
**15**	**-1688.702766**	**0.92**
16	-1688.702431	1.13

Boltzmann average free energy (298.15 K) = -1688.703329 Ha

⇨ Minimal SCF energy was computed for conformation 6: -1688.95073758 Ha

State of ionizable compounds

KI

K^+ + I^- —(ΔG = -1.77 kcal/mol)→ KI

ΔG = -599.863105 Ha ΔG = -11.5880406 Ha ΔG = -611.453959 Ha

⇨ **KI** is mainly associated

$KHCO_3$

K^+ + HCO_3^- —(ΔG = -12.08 kcal/mol)→ $KHCO_3$

ΔG = -599.863105 Ha ΔG = -264.534460 Ha ΔG = -864.416808 Ha

⇨ **$KHCO_3$** is mainly associated

2a⁺I⁻ (Boltzmann average free energies are given)

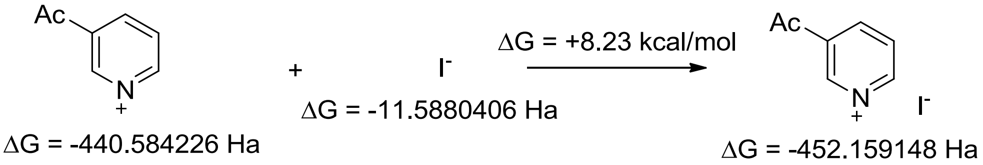

⇨ **$2a^+I^-$** is mainly dissociated

DIPEA-HI

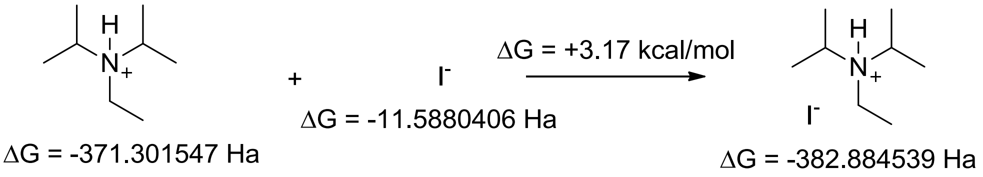

⇨ $[DIPEA\text{-}H]^+I^-$ is mainly dissociated

Potassium 3-oxocyclohex-1-enolate

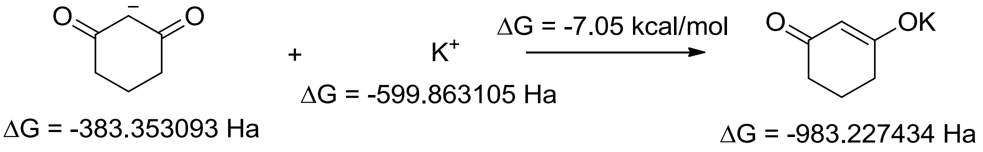

⇨ Potassium 3-oxocyclohex-1-enolate is mainly associated

6a-KI (Boltzmann average free energies are given)

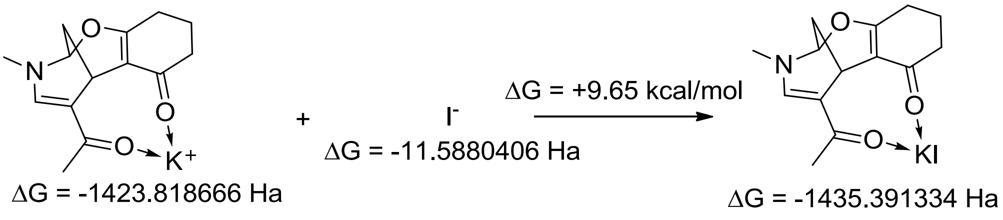

⇨ **6a-KI** is mainly dissociated

6aa-KI (Boltzmann average free energies are given)

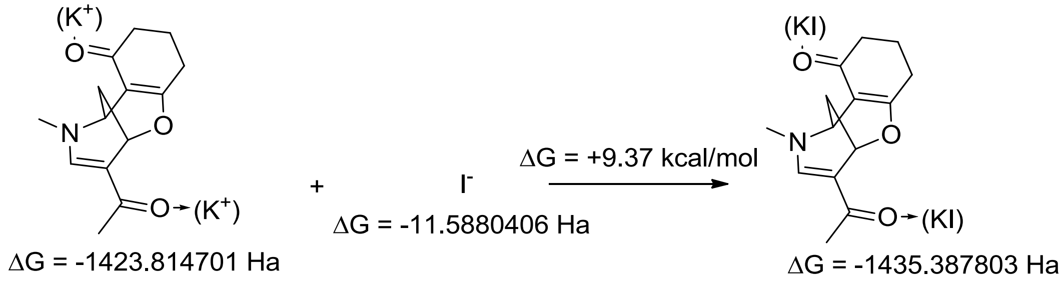

⇨ **6aa-KI** is mainly dissociated

6a-KHCO₃ (Boltzmann average free energies are given)

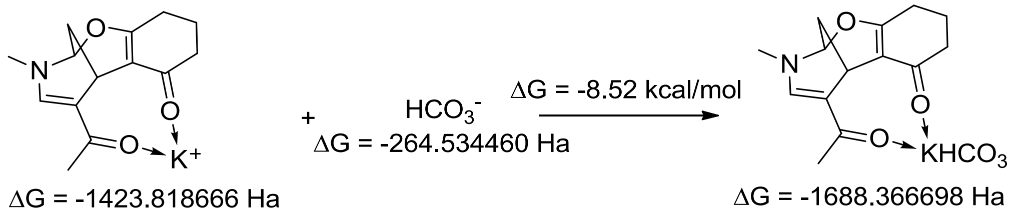

⇨ **6a-KHCO₃** is mainly associated

6aa-KHCO₃ (Boltzmann average free energies are given)

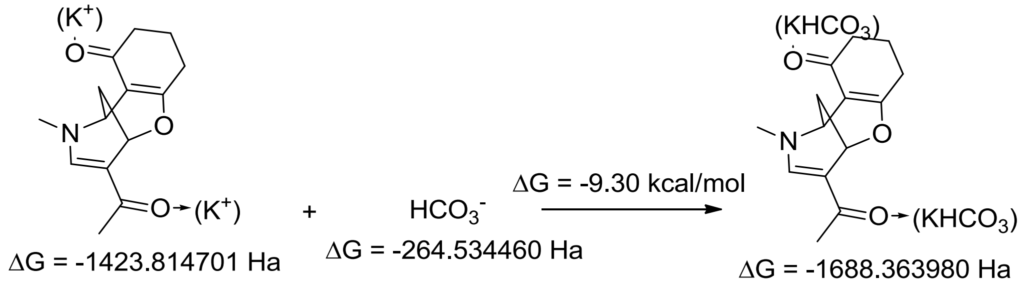

⇨ **6aa-KHCO₃** is mainly associated

Formation of 6a and 6aa, thermodynamic analysis

Reaction in absence of metal cation

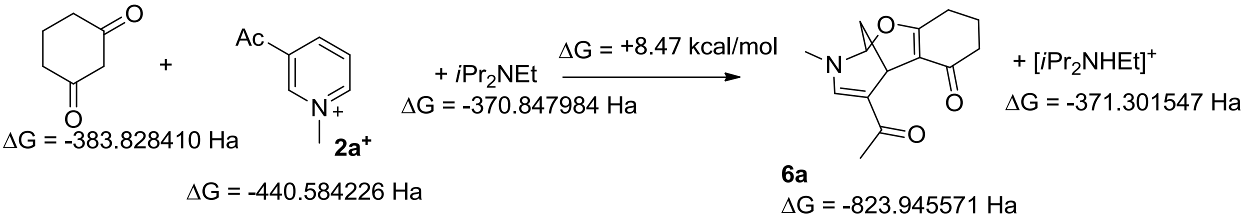

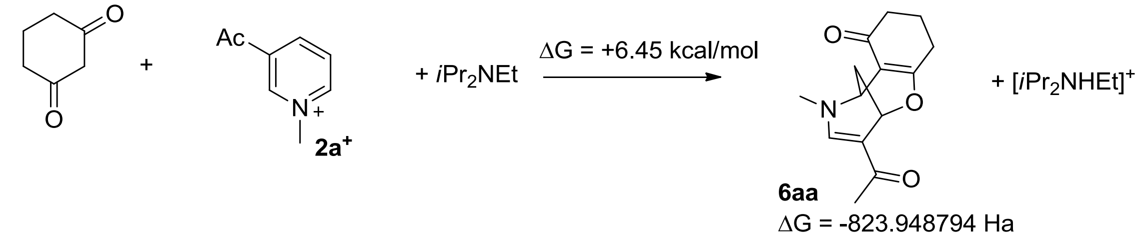

For conformationally not rigid molecules, Boltzmann average Gibbs free energies are given. In the experiment no reaction is observed.

Reaction in presence of KI

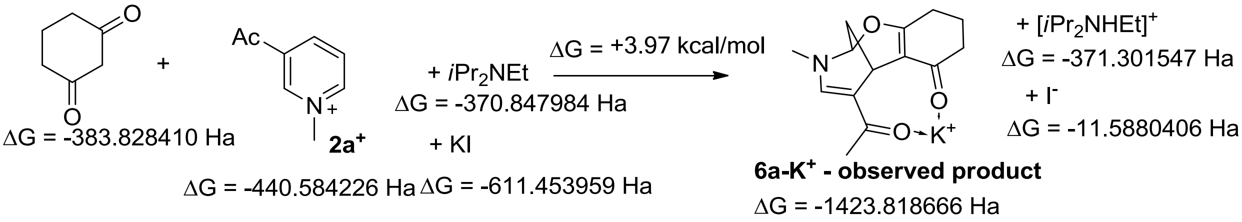

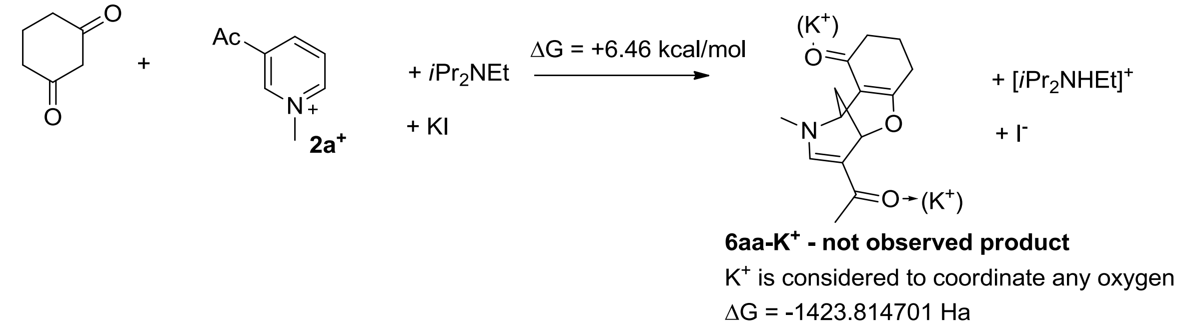

For conformationally not rigid molecules, Boltzmann average Gibbs free energies are given. As long as regioselective formation of **6a** is observed in experiment, the driving force must be the observed formation of precipitated material of polymeric nature, from which **6a** is liberated under action of silica gel.

Reaction in the presence of K_2CO_3

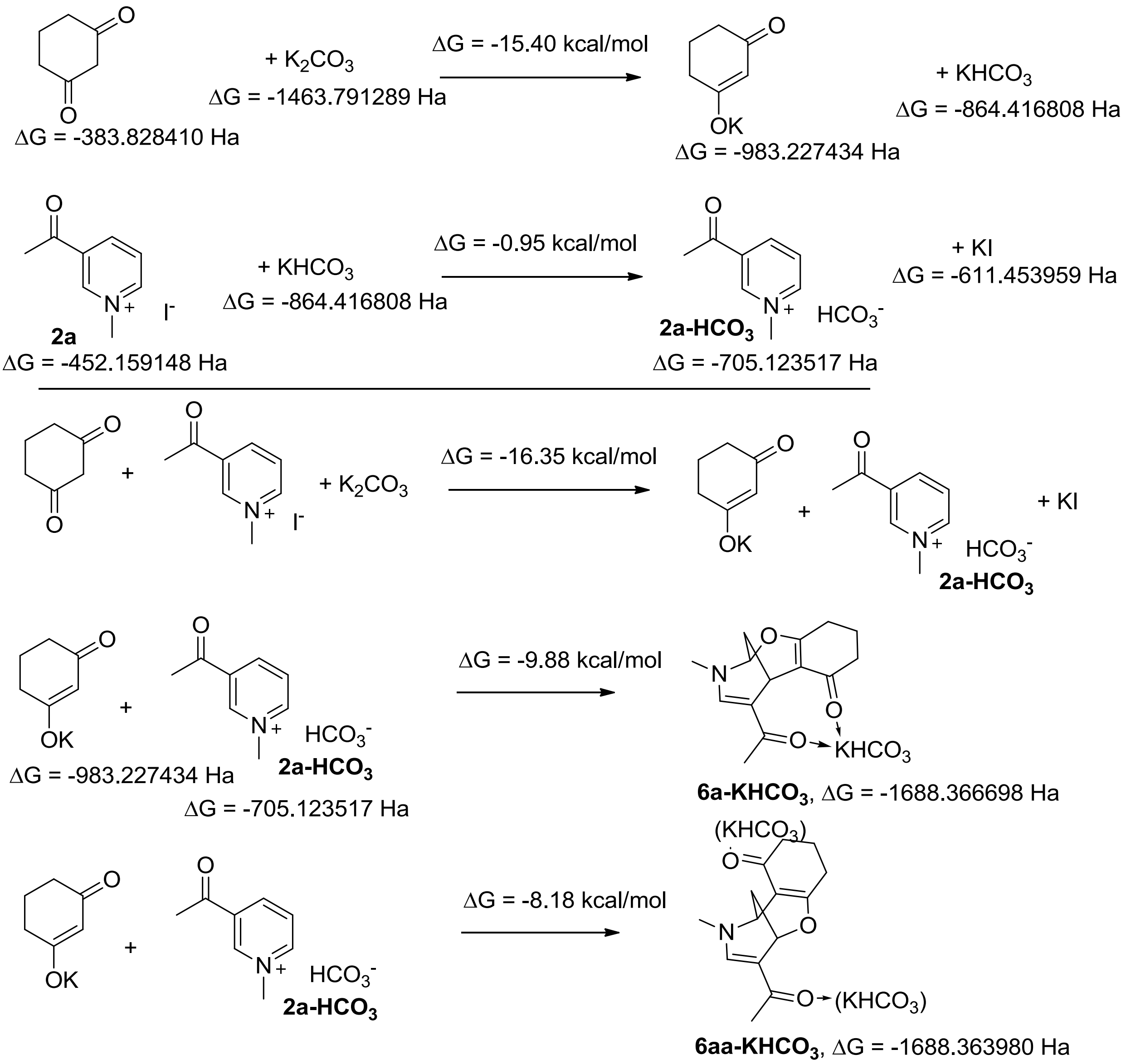

For conformationally not rigid molecules, Boltzmann average Gibbs free energies are given. In experiment the formation of only **6a** as product was observed. Given Gibbs free energy changes are relevant for acetonitrile solution phase; however in reality K_2CO_3 is hardly soluble in MeCN, and as well the reaction product is insoluble in MeCN (see main document).

Reaction of 3-oxocyclohex-1-enolate with 3-acetyl-1-methyl pyridinium. Impossibility of initial C-O bond formation

The reaction of **2a** with 1,3-cyclohexanedione leading to either observed product **6a** or not observed product **6aa** (see scheme below) in essence must include the following stages: formation of a new C–C bond, formation of a new C–O bond and proton migration. Starting with **2a**$^+$ and deprotonated 1,3-cyclohexanedione one awaits the reaction of nucleophilic addition, hence formation of a C–C or C–O bond. By computations of 3-oxocyclohex-1-enolate reacting with **2a**$^+$ cation (potassium and base were not included for simplicity) we have proven the impossibility of initial C–O bond formation. The latter bond must be broken to form a new C–C bond in intermediate **S2**, i.e., any TS directly connecting intermediates **S3** and **S2** was not located (Scheme S2). On the other hand TSs **S1** were located in reactions of 3-oxocyclohex-1-enolate with **2a**$^+$ forming a new C–C bond. In addition, intermediates **S3** do not contain any active hydrogen suspected to migration. Contrary the intermediates **S2** have an acidic proton prone to migration with the assistance of base (Scheme S2). In summary, the C-C bond formation, hence formation of TS **S1**, is a key stage (although not rate limiting!) in defining the reaction pathway as well as the product.

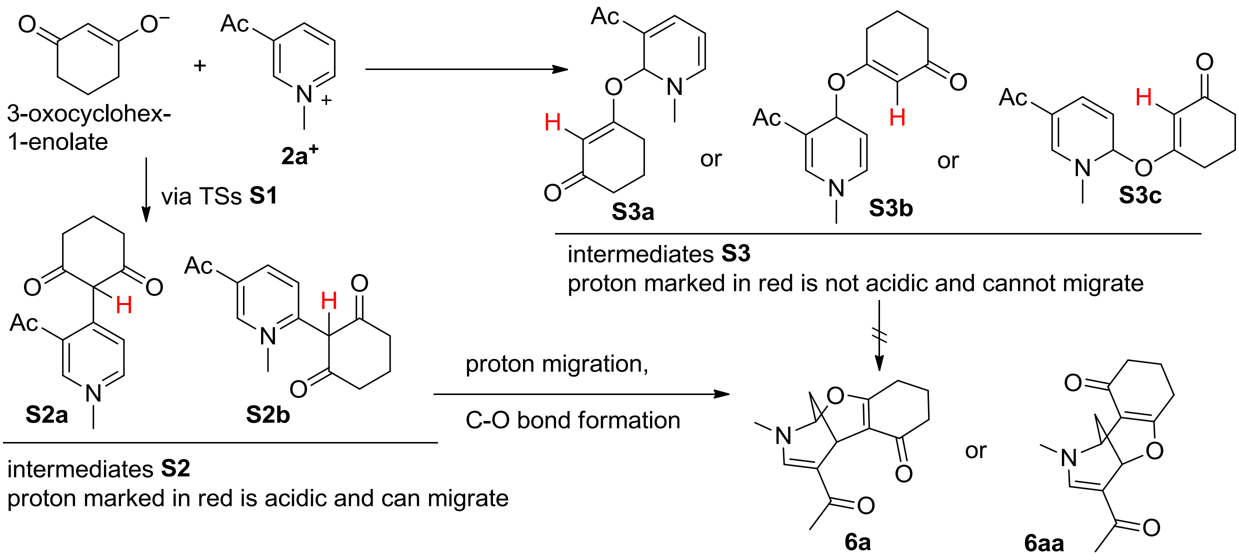

Scheme S2

The TSs **S1a** (4C-attack), **S1b** (6C-attack) and **S1c** (2C-attack) were located and computed first without potassium. Thereafter potassium cation was added rendering new TSs **K-S1a-b** (TSs **K-S1c** corresponding to 2C-attack were ignored as formation of the corresponding products was not observed with any pyridinium salt and cyclic diketone reported herein). The addition of K$^+$ lowers the energy of TSs **S1**, compared with free **S1** and free K$^+$. On the other hand TSs **K-S1a** and **K-S1b** have similar energy, hence nucleophilic attack is not responsible for regiospecificity in product formation. Among TSs from the **K-S1a** family, the three most important are given with their relative energies in the figure below.

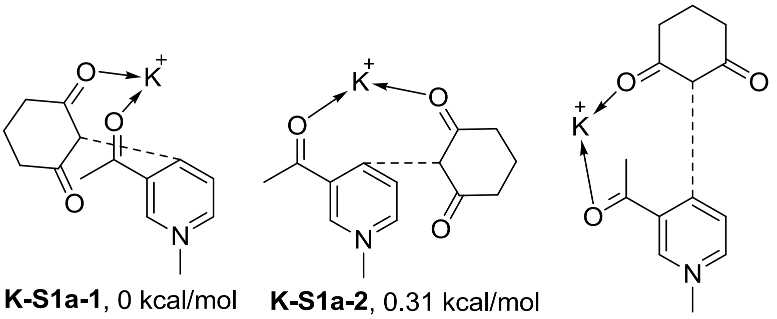

K-S1a-1, 0 kcal/mol **K-S1a-2**, 0.31 kcal/mol **K-S1a-3**, 1.57 kcal/mol

S3a (conformation 1)

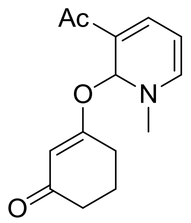

Center Number	Atomic Number	Atomic Type	Coordinates (Angstroms) X	Y	Z
1	6	0	1.202532	1.913881	1.494990
2	6	0	1.470748	0.620828	-0.551930
3	6	0	1.707102	-0.554018	0.324875
4	6	0	1.137906	0.786746	2.275511
5	1	0	1.071423	2.909165	1.905109
6	1	0	2.161124	0.689037	-1.391780
7	1	0	0.927259	0.872539	3.333264
8	7	0	1.495070	1.859477	0.170266
9	6	0	1.659624	3.081204	-0.619602
10	1	0	2.592792	3.026704	-1.187977
11	1	0	0.827808	3.205116	-1.317545
12	1	0	1.704731	3.942049	0.047031
13	6	0	2.154632	-1.842307	-0.242848
14	8	0	2.261303	-2.848409	0.461447
15	6	0	2.492788	-1.908557	-1.722224
16	1	0	1.665497	-1.539828	-2.336129
17	1	0	3.366327	-1.283871	-1.944723
18	1	0	2.720625	-2.940842	-1.989718
19	6	0	1.464626	-0.449512	1.674276
20	1	0	1.576607	-1.344159	2.279163
21	6	0	-3.433555	1.013812	-0.570397
22	6	0	-3.592948	-0.074710	0.496384
23	6	0	-1.968647	1.446489	-0.700999
24	1	0	-3.342104	0.333320	1.486815
25	1	0	-4.619075	-0.447984	0.556349
26	1	0	-3.778454	0.625160	-1.536361
27	1	0	-4.061156	1.877895	-0.332142
28	1	0	-1.670910	2.061662	0.161039
29	1	0	-1.817303	2.071694	-1.587553
30	6	0	-1.030996	0.268544	-0.791622
31	6	0	-2.666756	-1.259496	0.250548
32	8	0	-2.974035	-2.396956	0.613574
33	8	0	0.168262	0.541969	-1.367464
34	6	0	-1.384410	-0.980739	-0.395581
35	1	0	-0.710800	-1.819807	-0.525457

E(RB3LYP/6-31G(d,p)) = -824.150162227 Ha

S3a (conformation 2)

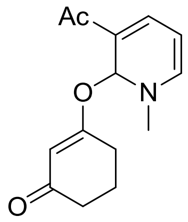

Center Number	Atomic Number	Atomic Type	Coordinates (Angstroms) X	Y	Z
1	6	0	-2.535416	-2.179069	0.222643
2	6	0	-1.087562	-0.329694	-0.392535
3	6	0	-2.218996	0.570042	-0.021548
4	6	0	-3.421952	-1.352464	0.863309
5	1	0	-2.649443	-3.257421	0.221720
6	1	0	-0.570653	-0.027005	-1.301201
7	1	0	-4.252134	-1.773581	1.414808
8	7	0	-1.495080	-1.700402	-0.510028
9	6	0	-0.623668	-2.612382	-1.253410
10	1	0	-0.479128	-2.239148	-2.271056
11	1	0	0.352275	-2.710735	-0.768888
12	1	0	-1.094161	-3.593839	-1.309170
13	6	0	-2.172997	2.016458	-0.306222
14	8	0	-3.056924	2.775814	0.097589
15	6	0	-1.019139	2.568163	-1.126869
16	1	0	-0.050655	2.335566	-0.672506
17	1	0	-1.016120	2.135750	-2.134430
18	1	0	-1.128937	3.650052	-1.207686
19	6	0	-3.285005	0.041216	0.664126
20	1	0	-4.056054	0.724747	1.006220
21	6	0	3.452621	-0.111164	1.744876
22	6	0	4.086334	0.477942	0.481334
23	6	0	1.960616	0.228249	1.815248
24	1	0	4.073083	1.576557	0.534377
25	1	0	5.131815	0.178922	0.365327
26	1	0	3.573153	-1.201156	1.738292
27	1	0	3.961254	0.260958	2.639214
28	1	0	1.817995	1.296559	2.031847
29	1	0	1.463156	-0.318117	2.622637
30	6	0	1.245956	-0.083029	0.523688
31	6	0	3.334890	0.078717	-0.782915
32	8	0	3.915709	0.006395	-1.869447
33	8	0	-0.074748	-0.223414	0.729674
34	6	0	1.903600	-0.176303	-0.666412
35	1	0	1.402128	-0.425749	-1.593711

E(RB3LYP/6-31G(d,p)) = -824.157322950 Ha

S3a (conformation 3)

Center Number	Atomic Number	Atomic Type	Coordinates (Angstroms) X	Y	Z
1	6	0	2.875890	-1.917276	-0.004456
2	6	0	1.088061	-0.360486	0.473502
3	6	0	1.942189	0.691516	-0.157286
4	6	0	3.453384	-1.060025	-0.908031
5	1	0	3.256816	-2.918859	0.162870
6	1	0	0.572858	0.001720	1.361923
7	1	0	4.295726	-1.388739	-1.501978
8	7	0	1.839082	-1.542183	0.786815
9	6	0	1.266658	-2.461989	1.770033
10	1	0	1.066205	-1.924231	2.700275
11	1	0	0.331705	-2.896300	1.402337
12	1	0	1.977670	-3.262441	1.975016
13	6	0	1.609951	2.094428	0.099965
14	8	0	0.733592	2.405554	0.915827
15	6	0	2.382003	3.185517	-0.624553
16	1	0	3.434817	3.186829	-0.323651
17	1	0	2.350003	3.046463	-1.709558
18	1	0	1.941306	4.150032	-0.370340
19	6	0	3.023533	0.289151	-0.907017
20	1	0	3.610703	1.022969	-1.450262
21	6	0	-3.570440	-1.402779	-0.810342
22	6	0	-4.115460	-0.005223	-0.503676
23	6	0	-2.120654	-1.329037	-1.301884
24	1	0	-4.210708	0.571367	-1.435839
25	1	0	-5.111872	-0.041307	-0.053814
26	1	0	-3.608804	-2.015832	0.098232
27	1	0	-4.193137	-1.902361	-1.558927
28	1	0	-2.081133	-0.888766	-2.308398
29	1	0	-1.674865	-2.325180	-1.380946
30	6	0	-1.264377	-0.491102	-0.384424
31	6	0	-3.204146	0.794085	0.422347
32	8	0	-3.666113	1.697256	1.127568
33	8	0	0.031556	-0.832196	-0.488443
34	6	0	-1.780145	0.487924	0.408834
35	1	0	-1.141836	1.126764	1.007675

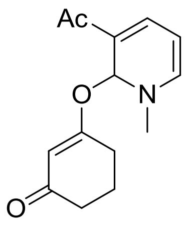

E(RB3LYP/6-31G(d,p)) = -824.160990179 Ha

S3a (conformation 4)

Center Number	Atomic Number	Atomic Type	X	Y	Z
1	6	0	-2.513499	-2.198017	0.336754
2	6	0	-1.043511	-0.403362	-0.373371
3	6	0	-2.174067	0.525052	-0.073591
4	6	0	-3.406973	-1.326556	0.904703
5	1	0	-2.636166	-3.273214	0.407039
6	1	0	-0.535656	-0.129570	-1.295904
7	1	0	-4.248847	-1.706690	1.467772
8	7	0	-1.456459	-1.776851	-0.406146
9	6	0	-0.577703	-2.739363	-1.071444
10	1	0	-0.416297	-2.436335	-2.109807
11	1	0	0.391175	-2.805120	-0.567092
12	1	0	-1.049048	-3.722176	-1.066327
13	6	0	-2.004357	1.913611	-0.511314
14	8	0	-0.996928	2.255178	-1.140280
15	6	0	-3.086973	2.931980	-0.193906
16	1	0	-4.041604	2.648967	-0.648974
17	1	0	-3.249942	3.012729	0.885711
18	1	0	-2.779107	3.903194	-0.582819
19	6	0	-3.261583	0.054040	0.619578
20	1	0	-4.052034	0.736362	0.916404
21	6	0	3.474361	0.122181	1.730016
22	6	0	4.085958	0.614601	0.415310
23	6	0	1.970443	0.408503	1.771444
24	1	0	4.028312	1.711929	0.364868
25	1	0	5.142828	0.348228	0.325701
26	1	0	3.636745	-0.958305	1.824485
27	1	0	3.967420	0.595290	2.584573
28	1	0	1.785945	1.486229	1.887255
29	1	0	1.493047	-0.078421	2.627620
30	6	0	1.270410	-0.047353	0.515738
31	6	0	3.352760	0.067469	-0.803908
32	8	0	3.940221	-0.087823	-1.878712
33	8	0	-0.042072	-0.227629	0.737824
34	6	0	1.933074	-0.228392	-0.660942
35	1	0	1.438633	-0.579318	-1.558530

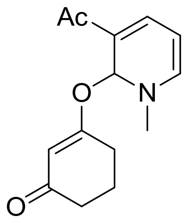

E(RB3LYP/6-31G(d,p)) = -824.160779850 Ha

S3b (conformation 1)

Center Number	Atomic Number	Atomic Type	Coordinates (Angstroms) X	Y	Z
1	6	0	-1.570189	1.712828	1.497676
2	6	0	-2.113479	0.576579	-0.508054
3	6	0	-1.704437	-0.628230	-0.002095
4	6	0	-1.132717	0.582721	2.078833
5	1	0	-1.624716	2.659266	2.022772
6	1	0	-2.568411	0.657906	-1.487919
7	1	0	-0.832855	0.605167	3.120418
8	7	0	-2.014186	1.746097	0.177775
9	6	0	-2.482378	3.014611	-0.387841
10	1	0	-3.318267	3.407447	0.197648
11	1	0	-1.672510	3.748129	-0.394578
12	1	0	-2.814254	2.848799	-1.412115
13	6	0	-1.967737	-1.888003	-0.708942
14	8	0	-1.675153	-2.967314	-0.187895
15	6	0	-2.622851	-1.864191	-2.082632
16	1	0	-3.635613	-1.450868	-2.032579
17	1	0	-2.050764	-1.253965	-2.788865
18	1	0	-2.677627	-2.886782	-2.457451
19	6	0	-1.030974	-0.705307	1.334585
20	1	0	-1.428728	-1.543547	1.911205
21	6	0	3.900827	-0.576341	0.390259
22	6	0	2.646351	-1.412101	0.658888
23	6	0	3.739674	0.246346	-0.890517
24	1	0	2.566358	-2.228772	-0.072982
25	1	0	2.685168	-1.885680	1.644743
26	1	0	4.071072	0.099140	1.237529
27	1	0	4.776600	-1.229156	0.321828
28	1	0	3.690455	-0.424678	-1.760917
29	1	0	4.584698	0.920197	-1.058758
30	6	0	2.461453	1.077467	-0.887018
31	6	0	1.381918	-0.587972	0.578655
32	8	0	0.398782	-1.182771	1.263433
33	8	0	2.393063	2.125810	-1.539314
34	6	0	1.325510	0.576885	-0.128560
35	1	0	0.429343	1.177170	-0.183620

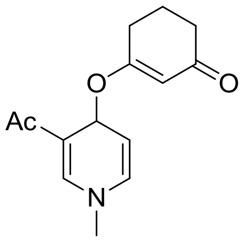

E(RB3LYP/6-31G(d,p)) = -824.155814282 Ha

S3b (conformation 2)

Center Number	Atomic Number	Atomic Type	Coordinates (Angstroms) X	Y	Z
1	6	0	-2.035012	-2.174483	-0.327384
2	6	0	-3.033332	-0.094582	0.191823
3	6	0	-1.941356	0.598331	-0.258012
4	6	0	-0.914893	-1.582941	-0.781097
5	1	0	-2.200274	-3.243411	-0.396150
6	1	0	-3.935118	0.409316	0.518777
7	1	0	-0.149054	-2.194675	-1.243011
8	7	0	-3.090746	-1.451511	0.217252
9	6	0	-4.247386	-2.163054	0.765573
10	1	0	-4.579743	-2.931154	0.063225
11	1	0	-3.996392	-2.635484	1.719777
12	1	0	-5.062099	-1.456715	0.924261
13	6	0	-1.954699	2.057248	-0.415411
14	8	0	-0.965534	2.644724	-0.862335
15	6	0	-3.200435	2.847735	-0.042690
16	1	0	-4.056985	2.551651	-0.656989
17	1	0	-3.474860	2.689235	1.005361
18	1	0	-2.999658	3.907772	-0.201333
19	6	0	-0.676307	-0.123971	-0.594605
20	1	0	-0.175512	0.353425	-1.438402
21	6	0	3.743279	0.706400	1.489496
22	6	0	2.297426	0.256586	1.723348
23	6	0	4.460340	-0.242801	0.525090
24	1	0	2.275516	-0.665138	2.322234
25	1	0	1.731280	1.005007	2.286323
26	1	0	3.739447	1.718816	1.067994
27	1	0	4.276223	0.759771	2.443801
28	1	0	4.581437	-1.230217	0.994682
29	1	0	5.463673	0.109796	0.269456
30	6	0	3.686587	-0.454503	-0.772236
31	6	0	1.574521	-0.009828	0.425973
32	8	0	0.249836	0.088410	0.577757
33	8	0	4.277967	-0.758361	-1.813973
34	6	0	2.238109	-0.328892	-0.723310
35	1	0	1.718134	-0.533060	-1.651717

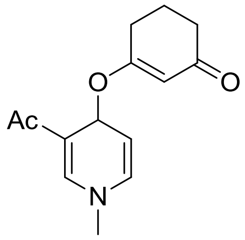

E(RB3LYP/6-31G(d,p)) = -824.160733426 Ha

S3b (conformation 3)

Center Number	Atomic Number	Atomic Type	X	Y	Z
1	6	0	-2.731997	-1.707777	-0.946107
2	6	0	-2.921864	0.257632	0.355587
3	6	0	-1.700902	0.716916	-0.069385
4	6	0	-1.517170	-1.346802	-1.398463
5	1	0	-3.261811	-2.579538	-1.311974
6	1	0	-3.579342	0.860775	0.970093
7	1	0	-1.039406	-1.937252	-2.171700
8	7	0	-3.423956	-0.950279	-0.007151
9	6	0	-4.704870	-1.437492	0.509832
10	1	0	-5.341613	-1.762031	-0.316653
11	1	0	-4.551521	-2.276737	1.194233
12	1	0	-5.206618	-0.631838	1.045518
13	6	0	-1.290683	2.107812	0.150287
14	8	0	-0.251179	2.546831	-0.351953
15	6	0	-2.168049	3.034002	0.980322
16	1	0	-3.137578	3.197315	0.498502
17	1	0	-2.358405	2.622084	1.976532
18	1	0	-1.659199	3.993213	1.080683
19	6	0	-0.774677	-0.206997	-0.792606
20	1	0	-0.151662	0.324100	-1.510057
21	6	0	3.632648	-1.273795	1.365802
22	6	0	2.230723	-1.717999	0.939503
23	6	0	4.389170	-0.658286	0.184914
24	1	0	2.288419	-2.608529	0.296807
25	1	0	1.620291	-2.003384	1.802444
26	1	0	3.546153	-0.530715	2.167980
27	1	0	4.185681	-2.124043	1.776848
28	1	0	4.577227	-1.427076	-0.579250
29	1	0	5.364405	-0.261478	0.481189
30	6	0	3.601369	0.465963	-0.480548
31	6	0	1.500280	-0.640203	0.175941
32	8	0	0.175605	-0.846924	0.201262
33	8	0	4.189251	1.393130	-1.049296
34	6	0	2.149563	0.383979	-0.447227
35	1	0	1.602007	1.191071	-0.918348

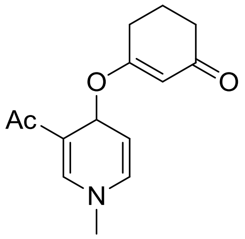

E(RB3LYP/6-31G(d,p)) = -824.159118263 Ha

S3b (conformation 4)

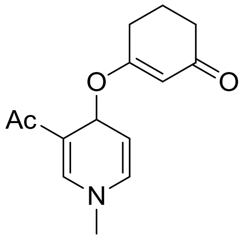

Center Number	Atomic Number	Atomic Type	X	Y	Z
1	6	0	-1.630327	1.681129	1.484094
2	6	0	-2.133454	0.604358	-0.558361
3	6	0	-1.730905	-0.615938	-0.086015
4	6	0	-1.194692	0.535140	2.035331
5	1	0	-1.693252	2.610093	2.038519
6	1	0	-2.563717	0.678168	-1.550566
7	1	0	-0.904753	0.526748	3.079757
8	7	0	-2.058909	1.752015	0.161090
9	6	0	-2.540101	3.030347	-0.370892
10	1	0	-3.405774	3.379886	0.198697
11	1	0	-1.748844	3.782065	-0.319525
12	1	0	-2.830356	2.895586	-1.412138
13	6	0	-1.977009	-1.788058	-0.950033
14	8	0	-2.445658	-1.670295	-2.085221
15	6	0	-1.658514	-3.165017	-0.396537
16	1	0	-0.624172	-3.222996	-0.047364
17	1	0	-2.300606	-3.385159	0.465225
18	1	0	-1.836273	-3.912171	-1.170742
19	6	0	-1.081251	-0.733035	1.257709
20	1	0	-1.473690	-1.576596	1.831852
21	6	0	3.878266	-0.553569	0.432096
22	6	0	2.621928	-1.406455	0.630554
23	6	0	3.745049	0.325527	-0.813966
24	1	0	2.565311	-2.188393	-0.140461
25	1	0	2.639051	-1.924629	1.594318
26	1	0	4.023068	0.083291	1.313264
27	1	0	4.758988	-1.198818	0.356445
28	1	0	3.721746	-0.305544	-1.714778
29	1	0	4.590189	1.010143	-0.929655
30	6	0	2.462426	1.149345	-0.806723
31	6	0	1.356405	-0.583876	0.556364
32	8	0	0.357389	-1.211786	1.192514
33	8	0	2.403098	2.224563	-1.413636
34	6	0	1.309773	0.609738	-0.099886
35	1	0	0.411260	1.206550	-0.152141

E(RB3LYP/6-31G(d,p)) = -824.155573855 Ha

S3b (conformation 5)

Center Number	Atomic Number	Atomic Type	Coordinates (Angstroms) X	Y	Z
1	6	0	-2.055872	-2.138336	-0.344448
2	6	0	-3.068627	-0.079244	0.199642
3	6	0	-1.988662	0.636134	-0.245367
4	6	0	-0.943278	-1.528280	-0.792019
5	1	0	-2.205609	-3.208827	-0.423331
6	1	0	-3.956861	0.444828	0.533459
7	1	0	-0.169411	-2.127570	-1.256090
8	7	0	-3.119958	-1.432990	0.205669
9	6	0	-4.281897	-2.160975	0.720844
10	1	0	-4.637305	-2.876014	-0.025276
11	1	0	-4.024921	-2.698584	1.637924
12	1	0	-5.080149	-1.451878	0.937914
13	6	0	-2.122576	2.104064	-0.292924
14	8	0	-3.152095	2.674906	0.077869
15	6	0	-0.953782	2.915272	-0.822862
16	1	0	-0.050173	2.730088	-0.234327
17	1	0	-0.726943	2.640945	-1.859730
18	1	0	-1.207580	3.975020	-0.782034
19	6	0	-0.717483	-0.066341	-0.600174
20	1	0	-0.210218	0.392125	-1.452002
21	6	0	3.738261	0.624633	1.493339
22	6	0	2.273464	0.237429	1.721536
23	6	0	4.418615	-0.356570	0.534330
24	1	0	2.208845	-0.680342	2.323325
25	1	0	1.737017	1.011060	2.279538
26	1	0	3.779708	1.635371	1.069802
27	1	0	4.268555	0.657227	2.449968
28	1	0	4.493234	-1.347680	1.005763
29	1	0	5.437856	-0.048818	0.283948
30	6	0	3.643491	-0.535352	-0.766766
31	6	0	1.546222	-0.003839	0.421866
32	8	0	0.223934	0.152335	0.567527
33	8	0	4.225479	-0.860763	-1.806715
34	6	0	2.200087	-0.350900	-0.724096
35	1	0	1.677326	-0.534009	-1.655379

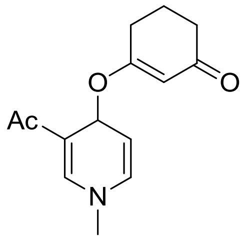

E(RB3LYP/6-31G(d,p)) = -824.159769809 Ha

S3b (conformation 6)

Center Number	Atomic Number	Atomic Type	X	Y	Z
1	6	0	-2.055866	-2.138335	-0.344459
2	6	0	-3.068624	-0.079249	0.199646
3	6	0	-1.988664	0.636135	-0.245367
4	6	0	-0.943275	-1.528273	-0.792033
5	1	0	-2.205601	-3.208825	-0.423350
6	1	0	-3.956860	0.444818	0.533467
7	1	0	-0.169410	-2.127558	-1.256114
8	7	0	-3.119949	-1.432995	0.205670
9	6	0	-4.281883	-2.160987	0.720845
10	1	0	-4.637302	-2.876012	-0.025283
11	1	0	-4.024897	-2.698613	1.637912
12	1	0	-5.080131	-1.451892	0.937938
13	6	0	-2.122583	2.104063	-0.292920
14	8	0	-3.152105	2.674901	0.077874
15	6	0	-0.953792	2.915277	-0.822854
16	1	0	-0.050181	2.730090	-0.234323
17	1	0	-0.726955	2.640958	-1.859726
18	1	0	-1.207591	3.975025	-0.782018
19	6	0	-0.717482	-0.066335	-0.600175
20	1	0	-0.210219	0.392137	-1.452001
21	6	0	3.738259	0.624626	1.493344
22	6	0	2.273463	0.237419	1.721539
23	6	0	4.418614	-0.356571	0.534330
24	1	0	2.208845	-0.680357	2.323321
25	1	0	1.737015	1.011046	2.279547
26	1	0	3.779706	1.635367	1.069813
27	1	0	4.268553	0.657215	2.449973
28	1	0	4.493233	-1.347684	1.005757
29	1	0	5.437855	-0.048817	0.283950
30	6	0	3.643491	-0.535344	-0.766768
31	6	0	1.546222	-0.003839	0.421866
32	8	0	0.223933	0.152335	0.567528
33	8	0	4.225480	-0.860748	-1.806719
34	6	0	2.200087	-0.350893	-0.724098
35	1	0	1.677326	-0.533995	-1.655382

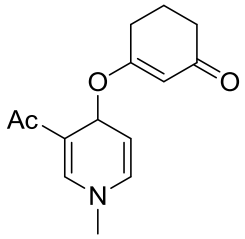

E(RB3LYP/6-31G(d,p)) = -824.159769810 Ha

S3c (conformation 1)

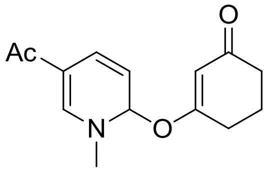

Center Number	Atomic Number	Atomic Type	X	Y	Z
1	6	0	0.242083	-1.954368	0.618694
2	6	0	1.845750	-0.723417	-0.779367
3	6	0	2.297860	0.049396	0.265225
4	6	0	0.821682	-1.187852	1.756119
5	1	0	0.232232	-3.033156	0.808322
6	1	0	2.216454	-0.600972	-1.790282
7	1	0	0.439049	-1.437793	2.739696
8	7	0	0.930439	-1.708756	-0.628591
9	6	0	0.537811	-2.551255	-1.759599
10	1	0	-0.476650	-2.313959	-2.090536
11	1	0	1.230807	-2.397589	-2.586444
12	1	0	0.572171	-3.602747	-1.458829
13	6	0	3.313329	1.096935	0.098569
14	8	0	3.721370	1.736797	1.071111
15	6	0	3.861740	1.392792	-1.289323
16	1	0	4.323668	0.504925	-1.733502
17	1	0	3.067436	1.725173	-1.965737
18	1	0	4.611555	2.180246	-1.207209
19	6	0	1.768117	-0.246885	1.578343
20	1	0	2.173876	0.304515	2.419881
21	6	0	-4.095989	0.354019	-0.383291
22	6	0	-3.340515	-0.740233	0.376797
23	6	0	-3.544094	1.738765	-0.033729
24	1	0	-3.598598	-0.714000	1.445389
25	1	0	-3.615050	-1.737198	0.018411
26	1	0	-3.989000	0.181251	-1.461035
27	1	0	-5.164603	0.296422	-0.155064
28	1	0	-3.753585	1.968429	1.021521
29	1	0	-4.007516	2.531176	-0.628133
30	6	0	-2.034225	1.823972	-0.221875
31	6	0	-1.844585	-0.585194	0.253981
32	8	0	-1.234547	-1.766184	0.480610
33	8	0	-1.490857	2.901239	-0.483205
34	6	0	-1.255484	0.602932	-0.044459
35	1	0	-0.183928	0.714121	-0.140945

E(RB3LYP/6-31G(d,p)) = -824.158622846 Ha

S3c (conformation 2)

Center Number	Atomic Number	Atomic Type	Coordinates (Angstroms) X	Y	Z
1	6	0	0.272398	0.252031	-0.844066
2	6	0	2.547225	0.917737	-0.269664
3	6	0	2.958061	-0.377379	-0.058700
4	6	0	0.791245	-1.142069	-0.865756
5	1	0	-0.375257	0.496215	-1.690425
6	1	0	3.199178	1.766553	-0.100317
7	1	0	0.107936	-1.919524	-1.187531
8	7	0	1.322251	1.228636	-0.757226
9	6	0	0.945159	2.618680	-1.021744
10	1	0	0.216741	2.967727	-0.285084
11	1	0	1.831882	3.250740	-0.979705
12	1	0	0.505076	2.695261	-2.020268
13	6	0	4.293690	-0.715970	0.447064
14	8	0	4.637159	-1.893681	0.579283
15	6	0	5.259492	0.401270	0.813706
16	1	0	5.487645	1.028495	-0.054656
17	1	0	4.841301	1.051253	1.589074
18	1	0	6.184494	-0.044037	1.181410
19	6	0	2.036878	-1.425311	-0.436484
20	1	0	2.396713	-2.448062	-0.397916
21	6	0	-3.898233	-0.039349	1.859983
22	6	0	-2.574204	0.689603	1.610282
23	6	0	-4.761135	-0.049713	0.594903
24	1	0	-2.743628	1.771041	1.507774
25	1	0	-1.882835	0.564192	2.449513
26	1	0	-3.687771	-1.071295	2.165384
27	1	0	-4.434386	0.435731	2.686982
28	1	0	-5.070260	0.976126	0.345661
29	1	0	-5.676525	-0.633720	0.724743
30	6	0	-4.012740	-0.601675	-0.613057
31	6	0	-1.891861	0.202846	0.356821
32	8	0	-0.578379	0.477291	0.377244
33	8	0	-4.619328	-1.151132	-1.536990
34	6	0	-2.566434	-0.421457	-0.649389
35	1	0	-2.068914	-0.802427	-1.533762

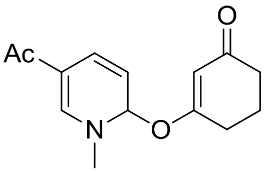

E(RB3LYP/6-31G(d,p)) = -824.162245306 Ha

S3c (conformation 3)

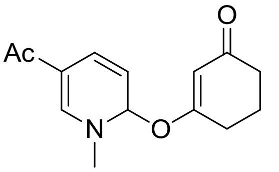

Center Number	Atomic Number	Atomic Type	Coordinates (Angstroms) X	Y	Z
1	6	0	0.272398	0.252003	-0.844048
2	6	0	2.547228	0.917731	-0.269677
3	6	0	2.958071	-0.377379	-0.058686
4	6	0	0.791249	-1.142095	-0.865700
5	1	0	-0.375252	0.496163	-1.690417
6	1	0	3.199181	1.766553	-0.100356
7	1	0	0.107939	-1.919560	-1.187450
8	7	0	1.322247	1.228615	-0.757225
9	6	0	0.945153	2.618649	-1.021795
10	1	0	0.216717	2.967713	-0.285162
11	1	0	1.831871	3.250716	-0.979754
12	1	0	0.505092	2.695199	-2.020331
13	6	0	4.293707	-0.715952	0.447069
14	8	0	4.637180	-1.893659	0.579320
15	6	0	5.259516	0.401300	0.813657
16	1	0	5.487659	1.028492	-0.054733
17	1	0	4.841334	1.051313	1.589005
18	1	0	6.184521	-0.043994	1.181366
19	6	0	2.036887	-1.425323	-0.436431
20	1	0	2.396724	-2.448072	-0.397839
21	6	0	-3.898260	-0.039293	1.859969
22	6	0	-2.574226	0.689648	1.610261
23	6	0	-4.761148	-0.049693	0.594880
24	1	0	-2.743646	1.771084	1.507718
25	1	0	-1.882866	0.564260	2.449503
26	1	0	-3.687805	-1.071230	2.165405
27	1	0	-4.434421	0.435815	2.686947
28	1	0	-5.070269	0.976139	0.345603
29	1	0	-5.676540	-0.633695	0.724727
30	6	0	-4.012741	-0.601694	-0.613055
31	6	0	-1.891872	0.202851	0.356822
32	8	0	-0.578389	0.477292	0.377250
33	8	0	-4.619320	-1.151179	-1.536977
34	6	0	-2.566435	-0.421479	-0.649377
35	1	0	-2.068906	-0.802480	-1.533733

E(RB3LYP/6-31G(d,p)) = -824.162245296 Ha

S3c (conformation 4)

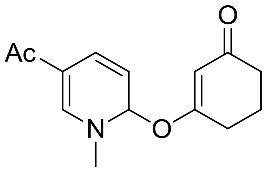

Center Number	Atomic Number	Atomic Type	Coordinates (Angstroms) X	Y	Z
1	6	0	0.241655	-1.929229	0.687658
2	6	0	1.820065	-0.809976	-0.819098
3	6	0	2.297234	0.036922	0.156907
4	6	0	0.851869	-1.093471	1.756963
5	1	0	0.226544	-2.992475	0.950190
6	1	0	2.196219	-0.733932	-1.833179
7	1	0	0.494954	-1.277508	2.764159
8	7	0	0.907434	-1.775291	-0.587774
9	6	0	0.502691	-2.704165	-1.644764
10	1	0	-0.537587	-2.534219	-1.932819
11	1	0	1.144636	-2.566275	-2.514151
12	1	0	0.604429	-3.732906	-1.285269
13	6	0	3.308027	1.035651	-0.224316
14	8	0	3.700376	1.150262	-1.388983
15	6	0	3.868963	1.937697	0.861281
16	1	0	3.072883	2.518542	1.339558
17	1	0	4.358018	1.350750	1.646509
18	1	0	4.595236	2.619212	0.417241
19	6	0	1.798157	-0.167652	1.498395
20	1	0	2.204793	0.417242	2.317012
21	6	0	-4.103709	0.369285	-0.319358
22	6	0	-3.334304	-0.694533	0.469285
23	6	0	-3.525823	1.763040	-0.058921
24	1	0	-3.557120	-0.613298	1.543060
25	1	0	-3.630165	-1.704815	0.169852
26	1	0	-4.035368	0.141585	-1.390006
27	1	0	-5.164476	0.336310	-0.052857
28	1	0	-3.696637	2.048076	0.989782
29	1	0	-4.001095	2.530137	-0.676723
30	6	0	-2.022556	1.820419	-0.302133
31	6	0	-1.841746	-0.562960	0.291321
32	8	0	-1.235929	-1.737080	0.562639
33	8	0	-1.477271	2.876664	-0.635332
34	6	0	-1.250929	0.600445	-0.089177
35	1	0	-0.182287	0.693002	-0.228787

E(RB3LYP/6-31G(d,p)) = -824.158187051 Ha

S3c (conformation 5)

Center Number	Atomic Number	Atomic Type	Coordinates (Angstroms) X	Y	Z
1	6	0	0.274713	0.346643	-0.822156
2	6	0	2.523171	1.030668	-0.191770
3	6	0	2.973680	-0.263742	-0.063637
4	6	0	0.834082	-1.025944	-0.939420
5	1	0	-0.378061	0.630318	-1.652063
6	1	0	3.177846	1.860755	0.048288
7	1	0	0.176721	-1.797446	-1.322808
8	7	0	1.297911	1.344150	-0.663724
9	6	0	0.887686	2.739182	-0.839955
10	1	0	0.154838	3.024007	-0.080515
11	1	0	1.760577	3.386571	-0.761152
12	1	0	0.440914	2.866461	-1.830223
13	6	0	4.336881	-0.477681	0.444688
14	8	0	5.047997	0.462179	0.812265
15	6	0	4.862004	-1.901487	0.512708
16	1	0	4.223112	-2.526917	1.145593
17	1	0	4.881430	-2.361274	-0.481529
18	1	0	5.872883	-1.888688	0.921634
19	6	0	2.086863	-1.308077	-0.522233
20	1	0	2.444944	-2.331368	-0.572269
21	6	0	-3.895717	-0.203753	1.844935
22	6	0	-2.585699	0.564795	1.643725
23	6	0	-4.754624	-0.157787	0.577766
24	1	0	-2.776737	1.646781	1.604939
25	1	0	-1.894292	0.403617	2.476776
26	1	0	-3.665560	-1.247316	2.090901
27	1	0	-4.443793	0.211790	2.695799
28	1	0	-5.086131	0.873870	0.388567
29	1	0	-5.657059	-0.768734	0.668617
30	6	0	-3.991409	-0.619727	-0.658373
31	6	0	-1.890256	0.167676	0.366424
32	8	0	-0.582690	0.466889	0.409110
33	8	0	-4.584008	-1.124306	-1.616323
34	6	0	-2.549009	-0.408173	-0.678194
35	1	0	-2.041580	-0.725134	-1.581935

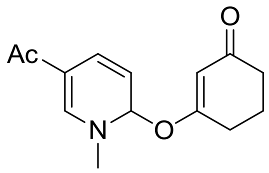

E(RB3LYP/6-31G(d,p)) = -824.161877508 Ha

S3c (conformation 6)

Center Number	Atomic Number	Atomic Type	Coordinates (Angstroms) X	Y	Z
1	6	0	0.274714	0.346644	-0.822168
2	6	0	2.523168	1.030668	-0.191773
3	6	0	2.973676	-0.263743	-0.063639
4	6	0	0.834083	-1.025942	-0.939437
5	1	0	-0.378066	0.630322	-1.652069
6	1	0	3.177842	1.860754	0.048290
7	1	0	0.176724	-1.797443	-1.322832
8	7	0	1.297912	1.344151	-0.663735
9	6	0	0.887685	2.739185	-0.839953
10	1	0	0.154873	3.024017	-0.080481
11	1	0	1.760582	3.386570	-0.761195
12	1	0	0.440866	2.866459	-1.830201
13	6	0	4.336873	-0.477682	0.444694
14	8	0	5.047988	0.462177	0.812275
15	6	0	4.861995	-1.901489	0.512718
16	1	0	4.223102	-2.526917	1.145603
17	1	0	4.881423	-2.361277	-0.481517
18	1	0	5.872873	-1.888690	0.921647
19	6	0	2.086862	-1.308076	-0.522243
20	1	0	2.444944	-2.331367	-0.572281
21	6	0	-3.895708	-0.203747	1.844940
22	6	0	-2.585691	0.564799	1.643723
23	6	0	-4.754620	-0.157787	0.577773
24	1	0	-2.776728	1.646785	1.604932
25	1	0	-1.894281	0.403626	2.476773
26	1	0	-3.665552	-1.247309	2.090911
27	1	0	-4.443782	0.211801	2.695804
28	1	0	-5.086128	0.873869	0.388571
29	1	0	-5.657053	-0.768734	0.668629
30	6	0	-3.991408	-0.619729	-0.658367
31	6	0	-1.890251	0.167674	0.366422
32	8	0	-0.582685	0.466886	0.409103
33	8	0	-4.584011	-1.124309	-1.616314
34	6	0	-2.549008	-0.408178	-0.678192
35	1	0	-2.041581	-0.725143	-1.581933

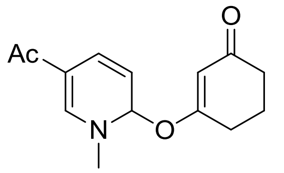

E(RB3LYP/6-31G(d,p)) = -824.161877508 Ha

S2a (conformation 1)

Center Number	Atomic Number	Atomic Type	Coordinates (Angstroms) X	Y	Z
1	6	0	-2.006019	-1.981280	-0.565690
2	6	0	-2.444686	0.225701	0.167697
3	6	0	-1.248968	0.676349	-0.324949
4	6	0	-0.804587	-1.632982	-1.049970
5	1	0	-2.460016	-2.948206	-0.749630
6	1	0	-3.202459	0.903638	0.543021
7	1	0	-0.264329	-2.342652	-1.666111
8	7	0	-2.798057	-1.087395	0.163620
9	6	0	-4.053432	-1.553342	0.749959
10	1	0	-3.861038	-2.232520	1.585783
11	1	0	-4.618451	-0.696983	1.118257
12	1	0	-4.653390	-2.076055	-0.000634
13	6	0	-1.007525	2.099675	-0.541913
14	8	0	0.038754	2.485334	-1.080962
15	6	0	-2.055097	3.125927	-0.130741
16	1	0	-2.980507	3.003696	-0.702527
17	1	0	-2.306698	3.037849	0.931074
18	1	0	-1.653433	4.122275	-0.318832
19	6	0	-0.164586	-0.314536	-0.698286
20	1	0	0.402447	0.082523	-1.545880
21	6	0	3.757596	0.115237	-0.091509
22	6	0	3.088375	-1.120077	-0.720406
23	6	0	2.742673	1.248361	0.134348
24	1	0	2.717090	-0.857776	-1.721782
25	1	0	3.786468	-1.953039	-0.831522
26	1	0	4.220472	-0.165881	0.861930
27	1	0	4.562840	0.467220	-0.742550
28	1	0	2.362091	1.605531	-0.830799
29	1	0	3.191117	2.099676	0.652544
30	6	0	1.539428	0.783665	0.932775
31	6	0	1.910329	-1.587509	0.113171
32	8	0	1.765553	-2.752611	0.450588
33	8	0	1.094871	1.408802	1.883675
34	6	0	0.886675	-0.528577	0.507518
35	1	0	0.309709	-0.909404	1.353885

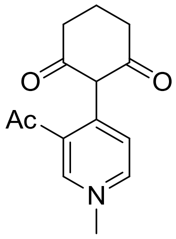

E(RB3LYP/6-31G(d,p)) = -824.164123160 Ha

ΔG (298.15 K, 1 atm, B3LYP/6-31G(d,p)) = -823.933216 Ha

S2a (conformation 2)

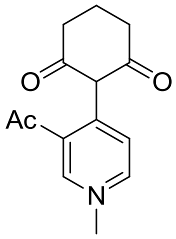

Center Number	Atomic Number	Atomic Type	Coordinates (Angstroms) X	Y	Z
1	6	0	2.531691	-1.024265	1.055068
2	6	0	1.972545	0.758051	-0.397359
3	6	0	0.777010	0.974353	0.241026
4	6	0	1.376681	-0.903811	1.723354
5	1	0	3.329768	-1.686273	1.370070
6	1	0	2.331494	1.427237	-1.170992
7	1	0	1.233039	-1.490143	2.625258
8	7	0	2.817244	-0.253905	-0.079109
9	6	0	4.037637	-0.514640	-0.839331
10	1	0	3.948341	-1.441819	-1.414260
11	1	0	4.217964	0.310765	-1.528413
12	1	0	4.890906	-0.599392	-0.161171
13	6	0	0.045373	2.220004	0.043616
14	8	0	-0.996393	2.439172	0.679141
15	6	0	0.560887	3.275619	-0.926460
16	1	0	1.518834	3.688103	-0.593925
17	1	0	0.707800	2.865927	-1.931050
18	1	0	-0.171404	4.082283	-0.976665
19	6	0	0.245406	-0.032528	1.246774
20	1	0	-0.201934	0.504603	2.090963
21	6	0	-2.696799	-0.783164	-1.681194
22	6	0	-2.955918	0.187948	-0.520866
23	6	0	-1.195515	-1.088335	-1.827808
24	1	0	-2.527739	1.169285	-0.759852
25	1	0	-4.023859	0.336955	-0.340150
26	1	0	-3.246293	-1.718187	-1.518474
27	1	0	-3.076567	-0.353043	-2.612384
28	1	0	-0.653118	-0.152758	-2.026172
29	1	0	-0.993602	-1.778216	-2.650794
30	6	0	-0.642372	-1.685825	-0.550973
31	6	0	-2.317665	-0.248636	0.786701
32	8	0	-2.861350	-0.061772	1.864422
33	8	0	0.033805	-2.704294	-0.544845
34	6	0	-0.969415	-0.973370	0.752564
35	1	0	-1.030107	-1.741250	1.531363

E(RB3LYP/6-31G(d,p)) = -824.161967225 Ha

ΔG (298.15 K, 1 atm, B3LYP/6-31G(d,p)) = -823.930615 Ha

S2a (conformation 3)

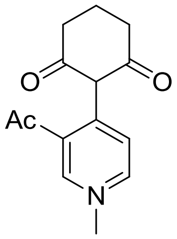

Center Number	Atomic Number	Atomic Type	Coordinates (Angstroms) X	Y	Z
1	6	0	0.817181	1.980995	-1.228677
2	6	0	2.256704	0.558077	-0.000336
3	6	0	1.489891	-0.542213	-0.268798
4	6	0	-0.009501	0.967046	-1.528285
5	1	0	0.704271	2.973539	-1.649457
6	1	0	3.203355	0.476677	0.520654
7	1	0	-0.806279	1.148834	-2.241997
8	7	0	1.926505	1.817474	-0.394222
9	6	0	2.730942	2.981197	-0.027901
10	1	0	2.190142	3.625308	0.672670
11	1	0	3.654538	2.646665	0.444838
12	1	0	2.983272	3.561385	-0.919761
13	6	0	1.965634	-1.886992	0.048518
14	8	0	1.275433	-2.875888	-0.232218
15	6	0	3.319107	-2.086291	0.718465
16	1	0	4.135890	-1.717483	0.089740
17	1	0	3.372390	-1.557530	1.675649
18	1	0	3.462326	-3.152998	0.894702
19	6	0	0.128313	-0.401729	-0.916667
20	1	0	0.009859	-1.176380	-1.683486
21	6	0	-3.422151	0.860131	0.904706
22	6	0	-2.015894	1.261567	1.380140
23	6	0	-3.376224	0.275163	-0.513432
24	1	0	-1.613472	2.030562	0.703996
25	1	0	-2.022989	1.673293	2.392312
26	1	0	-3.851074	0.125916	1.596959
27	1	0	-4.081364	1.732625	0.921850
28	1	0	-3.065726	1.064099	-1.215049
29	1	0	-4.352937	-0.083400	-0.848623
30	6	0	-2.376965	-0.857855	-0.666391
31	6	0	-1.064317	0.083043	1.344899
32	8	0	-0.301599	-0.165751	2.268647
33	8	0	-2.581990	-1.794922	-1.425055
34	6	0	-1.065413	-0.790714	0.105204
35	1	0	-0.806345	-1.809497	0.404895

E(RB3LYP/6-31G(d,p)) = -824.163446334 Ha

ΔG (298.15 K, 1 atm, B3LYP/6-31G(d,p)) = -823.932617 Ha

S2a (conformation 4)

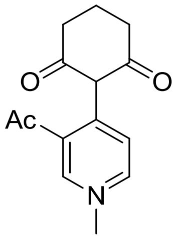

Center Number	Atomic Number	Atomic Type	Coordinates (Angstroms) X	Y	Z
1	6	0	-1.943144	-1.913842	-0.702340
2	6	0	-2.437277	0.193220	0.241377
3	6	0	-1.298169	0.753460	-0.275725
4	6	0	-0.785311	-1.460533	-1.206465
5	1	0	-2.369920	-2.874395	-0.967065
6	1	0	-3.195343	0.826387	0.688191
7	1	0	-0.250721	-2.068590	-1.927203
8	7	0	-2.724799	-1.131363	0.152721
9	6	0	-3.935662	-1.699637	0.743968
10	1	0	-3.680112	-2.444918	1.502676
11	1	0	-4.511767	-0.903224	1.214726
12	1	0	-4.550075	-2.174063	-0.026729
13	6	0	-1.273952	2.225268	-0.339373
14	8	0	-2.145400	2.912634	0.205890
15	6	0	-0.176419	2.927742	-1.124322
16	1	0	0.702512	3.073265	-0.488084
17	1	0	0.136684	2.376499	-2.015033
18	1	0	-0.542787	3.913037	-1.417742
19	6	0	-0.177654	-0.166725	-0.725709
20	1	0	0.410960	0.283686	-1.530398
21	6	0	3.784030	-0.121551	-0.040906
22	6	0	3.017908	-1.300429	-0.666773
23	6	0	2.876829	1.109138	0.120269
24	1	0	2.704146	-1.028332	-1.685253
25	1	0	3.635420	-2.198720	-0.737684
26	1	0	4.180194	-0.419673	0.936757
27	1	0	4.643001	0.135035	-0.666965
28	1	0	2.588986	1.471539	-0.877285
29	1	0	3.384753	1.930819	0.630827
30	6	0	1.601705	0.791439	0.883770
31	6	0	1.779188	-1.630603	0.142507
32	8	0	1.514679	-2.767152	0.501929
33	8	0	1.162342	1.529411	1.753785
34	6	0	0.852103	-0.472811	0.494319
35	1	0	0.229648	-0.782760	1.336788

E(RB3LYP/6-31G(d,p)) = -824.157045218 Ha

ΔG (298.15 K, 1 atm, B3LYP/6-31G(d,p)) = -823.925419 Ha

S2a (conformation 5)

Center Number	Atomic Number	Atomic Type	Coordinates (Angstroms) X	Y	Z
1	6	0	2.534878	-0.512481	1.155109
2	6	0	1.749021	0.842996	-0.616138
3	6	0	0.593093	1.142501	0.056865
4	6	0	1.417580	-0.304674	1.867348
5	1	0	3.412497	-1.002633	1.560043
6	1	0	2.003213	1.379027	-1.523942
7	1	0	1.381056	-0.629507	2.901617
8	7	0	2.670255	-0.043153	-0.156159
9	6	0	3.865582	-0.384515	-0.925310
10	1	0	3.829875	-1.427716	-1.253960
11	1	0	3.924627	0.258961	-1.803268
12	1	0	4.761758	-0.235307	-0.316953
13	6	0	-0.129363	2.347929	-0.392558
14	8	0	0.097353	2.873115	-1.488245
15	6	0	-1.127380	2.999010	0.553921
16	1	0	-1.966643	2.343247	0.798185
17	1	0	-0.639741	3.257090	1.501286
18	1	0	-1.506913	3.908281	0.086196
19	6	0	0.184403	0.293236	1.245829
20	1	0	-0.349928	0.893177	1.989618
21	6	0	-2.252521	-1.490364	-1.654506
22	6	0	-2.668886	-0.248990	-0.851908
23	6	0	-0.745396	-1.759868	-1.506696
24	1	0	-2.173867	0.633936	-1.283725
25	1	0	-3.745946	-0.068299	-0.891914
26	1	0	-2.821524	-2.362977	-1.312888
27	1	0	-2.499366	-1.344257	-2.709683
28	1	0	-0.188969	-0.902462	-1.909825
29	1	0	-0.431600	-2.653805	-2.050789
30	6	0	-0.359155	-1.925840	-0.050264
31	6	0	-2.252251	-0.320747	0.604940
32	8	0	-2.966149	0.101978	1.504078
33	8	0	0.336672	-2.852730	0.338058
34	6	0	-0.883253	-0.898599	0.942096
35	1	0	-0.981435	-1.401149	1.909619

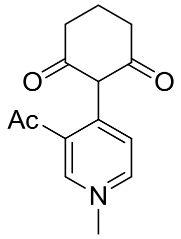

E(RB3LYP/6-31G(d,p)) = -824.156296650 Ha

ΔG (298.15 K, 1 atm, B3LYP/6-31G(d,p)) = -823.925409 Ha

S2a (conformation 6)

Center Number	Atomic Number	Atomic Type	X	Y	Z
1	6	0	-0.828365	-1.832811	-1.371997
2	6	0	-2.251511	-0.597577	0.054508
3	6	0	-1.547651	0.556566	-0.149376
4	6	0	-0.053765	-0.760627	-1.603412
5	1	0	-0.717014	-2.765310	-1.913521
6	1	0	-3.186535	-0.567671	0.602498
7	1	0	0.696429	-0.822528	-2.384497
8	7	0	-1.885931	-1.801659	-0.459549
9	6	0	-2.637113	-3.023642	-0.179470
10	1	0	-2.051111	-3.708533	0.441337
11	1	0	-3.554595	-2.767219	0.350524
12	1	0	-2.899255	-3.528419	-1.113391
13	6	0	-2.184549	1.810369	0.288451
14	8	0	-3.221632	1.819539	0.960854
15	6	0	-1.572929	3.129622	-0.159139
16	1	0	-0.515235	3.221461	0.104328
17	1	0	-1.635053	3.218289	-1.250712
18	1	0	-2.130951	3.949619	0.294515
19	6	0	-0.181793	0.510808	-0.802411
20	1	0	-0.039480	1.373772	-1.462614
21	6	0	3.483669	-0.930208	0.615143
22	6	0	2.122146	-1.521625	1.017883
23	6	0	3.352985	-0.030217	-0.622280
24	1	0	1.743480	-2.147027	0.196769
25	1	0	2.192450	-2.147058	1.911140
26	1	0	3.899517	-0.354851	1.450627
27	1	0	4.189116	-1.739569	0.407378
28	1	0	3.048827	-0.645687	-1.482412
29	1	0	4.297613	0.450039	-0.889968
30	6	0	2.299220	1.048783	-0.462932
31	6	0	1.095552	-0.436583	1.273564
32	8	0	0.350003	-0.458971	2.242113
33	8	0	2.434220	2.163740	-0.946114
34	6	0	1.010318	0.709864	0.277621
35	1	0	0.725288	1.601499	0.843117

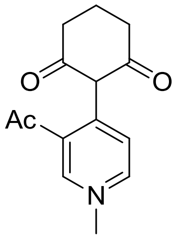

E(RB3LYP/6-31G(d,p)) = -824.158816951 Ha

ΔG (298.15 K, 1 atm, B3LYP/6-31G(d,p)) = -823.928788 Ha

S2b (conformation 1)

Center Number	Atomic Number	Atomic Type	Coordinates (Angstroms) X	Y	Z
1	6	0	0.306655	-0.004986	0.951394
2	6	0	-1.819517	1.014479	0.318061
3	6	0	-2.430225	-0.215194	0.182927
4	6	0	-0.504106	-1.233978	1.270572
5	1	0	1.024500	0.207183	1.749371
6	1	0	-2.325594	1.941009	0.068684
7	1	0	-0.018565	-2.034546	1.817348
8	7	0	-0.581929	1.156756	0.839192
9	6	0	-0.101967	2.456954	1.304040
10	1	0	0.148845	2.403092	2.368507
11	1	0	0.779282	2.783880	0.748180
12	1	0	-0.890190	3.198639	1.171757
13	6	0	-3.759650	-0.378664	-0.404549
14	8	0	-4.323520	-1.478704	-0.404529
15	6	0	-4.453014	0.823937	-1.031309
16	1	0	-4.629144	1.611841	-0.290808
17	1	0	-3.850908	1.257872	-1.835790
18	1	0	-5.413123	0.501198	-1.435682
19	6	0	-1.766609	-1.339718	0.820360
20	1	0	-2.349365	-2.241882	0.974867
21	6	0	4.122568	-0.138194	-0.234831
22	6	0	3.304897	1.163826	-0.163450
23	6	0	3.341991	-1.326532	0.355059
24	1	0	3.156447	1.434465	0.892031
25	1	0	3.819169	1.997161	-0.647498
26	1	0	4.382280	-0.352259	-1.277903
27	1	0	5.063005	-0.010354	0.307791
28	1	0	3.179589	-1.155496	1.429600
29	1	0	3.887795	-2.266634	0.248780
30	6	0	1.987310	-1.481563	-0.308997
31	6	0	1.940069	1.004779	-0.808686
32	8	0	1.489452	1.814294	-1.604838
33	8	0	1.580635	-2.549037	-0.736281
34	6	0	1.126126	-0.223870	-0.418747
35	1	0	0.369772	-0.392527	-1.189464

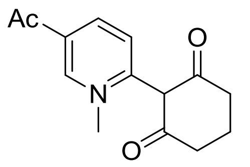

E(RB3LYP/6-31G(d,p)) = -824.159824700 Ha

ΔG (298.15 K, 1 atm, B3LYP/6-31G(d,p)) = -823.928928 Ha

S2b (conformation 2)

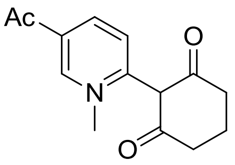

Center Number	Atomic Number	Atomic Type	X	Y	Z
1	6	0	-0.455559	1.021858	-0.829496
2	6	0	1.827279	1.033783	0.097519
3	6	0	2.157626	-0.183737	-0.454400
4	6	0	0.016463	-0.168490	-1.619976
5	1	0	-0.918921	1.759509	-1.497271
6	1	0	2.513973	1.574783	0.738881
7	1	0	-0.660636	-0.547553	-2.378742
8	7	0	0.664987	1.673589	-0.148380
9	6	0	0.417114	2.995136	0.423627
10	1	0	-0.124478	3.610219	-0.300528
11	1	0	-0.171245	2.920687	1.343566
12	1	0	1.368196	3.479095	0.648223
13	6	0	3.430000	-0.852226	-0.197841
14	8	0	3.711726	-1.919385	-0.756683
15	6	0	4.422223	-0.228157	0.775820
16	1	0	4.726633	0.772559	0.451210
17	1	0	3.991662	-0.132174	1.777945
18	1	0	5.305276	-0.866064	0.828118
19	6	0	1.212757	-0.742328	-1.406352
20	1	0	1.525769	-1.613331	-1.972489
21	6	0	-2.923622	-1.927059	0.817960
22	6	0	-3.179997	-1.291623	-0.555698
23	6	0	-1.488184	-1.650732	1.297237
24	1	0	-2.537419	-1.782517	-1.302219
25	1	0	-4.212962	-1.420513	-0.888832
26	1	0	-3.640297	-1.536397	1.549796
27	1	0	-3.089291	-3.006166	0.758339
28	1	0	-0.778245	-2.087652	0.579847
29	1	0	-1.287039	-2.088214	2.277988
30	6	0	-1.203445	-0.166454	1.365142
31	6	0	-2.847298	0.186911	-0.607591
32	8	0	-3.471755	0.970710	-1.306339
33	8	0	-0.625809	0.352119	2.310387
34	6	0	-1.656812	0.698312	0.200243
35	1	0	-1.950451	1.669880	0.608748

E(RB3LYP/6-31G(d,p)) = -824.162486 Ha

ΔG (298.15 K, 1 atm, B3LYP/6-31G(d,p)) = -823.931438491 Ha

S2b (conformation 3)

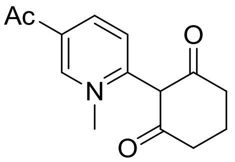

Center Number	Atomic Number	Atomic Type	X	Y	Z
1	6	0	-0.445850	-1.161032	0.891702
2	6	0	1.314643	-0.856144	-0.798522
3	6	0	2.180845	-0.289170	0.108954
4	6	0	0.645536	-0.914274	1.894644
5	1	0	-1.021539	-2.054121	1.158691
6	1	0	1.563156	-0.953175	-1.850221
7	1	0	0.401862	-1.088694	2.937795
8	7	0	0.120160	-1.385196	-0.445492
9	6	0	-0.534739	-2.366942	-1.310985
10	1	0	-0.385247	-3.379947	-0.919675
11	1	0	-1.607744	-2.182623	-1.377542
12	1	0	-0.105986	-2.311982	-2.312502
13	6	0	3.453872	0.314770	-0.283601
14	8	0	4.254106	0.718558	0.567603
15	6	0	3.793993	0.450898	-1.762187
16	1	0	3.835874	-0.527289	-2.253604
17	1	0	3.046969	1.052146	-2.290144
18	1	0	4.768897	0.931469	-1.852022
19	6	0	1.846048	-0.446566	1.515379
20	1	0	2.619348	-0.219851	2.241653
21	6	0	-2.672983	1.848090	-1.121507
22	6	0	-1.194513	1.972857	-0.711509
23	6	0	-3.113786	0.378769	-1.138387
24	1	0	-0.575341	1.413202	-1.425695
25	1	0	-0.855171	3.011067	-0.707899
26	1	0	-3.298259	2.419922	-0.425770
27	1	0	-2.819836	2.285754	-2.112683
28	1	0	-2.582522	-0.140386	-1.950058
29	1	0	-4.182076	0.264369	-1.339808
30	6	0	-2.795600	-0.377384	0.141132
31	6	0	-0.990169	1.396258	0.674512
32	8	0	-0.437967	2.017991	1.568931
33	8	0	-3.489037	-1.306805	0.526699
34	6	0	-1.554656	0.007972	0.948743
35	1	0	-1.856179	-0.010065	2.001855

E(RB3LYP/6-31G(d,p)) = -824.157679 Ha

ΔG (298.15 K, 1 atm, B3LYP/6-31G(d,p)) = -823.926667682 Ha

S2b (conformation 4)

Center Number	Atomic Number	Atomic Type	Coordinates (Angstroms) X	Y	Z
1	6	0	0.303011	0.045643	0.944547
2	6	0	-1.768526	1.130706	0.264557
3	6	0	-2.430960	-0.075935	0.149134
4	6	0	-0.561808	-1.137101	1.291547
5	1	0	1.024574	0.247953	1.741625
6	1	0	-2.263100	2.052175	-0.025109
7	1	0	-0.118403	-1.938429	1.871490
8	7	0	-0.537049	1.240458	0.796261
9	6	0	-0.007847	2.533931	1.226243
10	1	0	0.248998	2.494878	2.289758
11	1	0	0.880252	2.815791	0.656532
12	1	0	-0.771651	3.298270	1.081313
13	6	0	-3.756269	-0.080173	-0.475022
14	8	0	-4.227699	0.927196	-1.016283
15	6	0	-4.559905	-1.370578	-0.445053
16	1	0	-4.004201	-2.193185	-0.907883
17	1	0	-4.782353	-1.670708	0.585112
18	1	0	-5.496882	-1.219283	-0.982444
19	6	0	-1.825186	-1.206920	0.830166
20	1	0	-2.423823	-2.089869	1.031327
21	6	0	4.115591	-0.270776	-0.206988
22	6	0	3.350117	1.064282	-0.179155
23	6	0	3.284187	-1.411056	0.407394
24	1	0	3.204761	1.371656	0.866718
25	1	0	3.900977	1.862058	-0.682467
26	1	0	4.375204	-0.523513	-1.241399
27	1	0	5.055920	-0.165507	0.340650
28	1	0	3.121610	-1.206283	1.476050
29	1	0	3.792910	-2.374521	0.329849
30	6	0	1.927984	-1.530664	-0.260947
31	6	0	1.985279	0.940765	-0.831849
32	8	0	1.573902	1.743703	-1.655415
33	8	0	1.478794	-2.593397	-0.656264
34	6	0	1.119785	-0.242603	-0.413923
35	1	0	0.361866	-0.401826	-1.185145

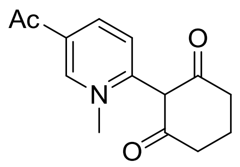

E(RB3LYP/6-31G(d,p)) = -824.159506 Ha

ΔG (298.15 K, 1 atm, B3LYP/6-31G(d,p)) = -823.928531242 Ha

S2b (conformation 5)

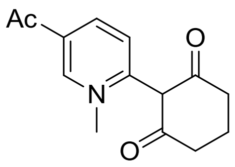

Center Number	Atomic Number	Atomic Type	Coordinates (Angstroms) X	Y	Z
1	6	0	-0.473048	1.016570	-0.863252
2	6	0	1.773284	1.135942	0.124466
3	6	0	2.163134	-0.090249	-0.371786
4	6	0	0.066483	-0.172600	-1.610008
5	1	0	-0.950633	1.714910	-1.561992
6	1	0	2.447308	1.692843	0.766690
7	1	0	-0.567043	-0.588126	-2.386544
8	7	0	0.606859	1.732600	-0.178435
9	6	0	0.305329	3.069502	0.329591
10	1	0	-0.214956	3.644354	-0.441655
11	1	0	-0.323067	3.015702	1.223934
12	1	0	1.235623	3.580661	0.578831
13	6	0	3.468019	-0.618225	0.027140
14	8	0	4.190642	-0.036920	0.846780
15	6	0	3.932633	-1.925699	-0.595982
16	1	0	3.221625	-2.734445	-0.394595
17	1	0	4.018764	-1.835204	-1.684574
18	1	0	4.906149	-2.190579	-0.181679
19	6	0	1.274934	-0.702865	-1.343546
20	1	0	1.609805	-1.571158	-1.902002
21	6	0	-2.806671	-1.992690	0.852627
22	6	0	-3.062916	-1.427931	-0.552088
23	6	0	-1.405340	-1.608825	1.357416
24	1	0	-2.367125	-1.903192	-1.259994
25	1	0	-4.075109	-1.636322	-0.907814
26	1	0	-3.566485	-1.619873	1.549378
27	1	0	-2.903979	-3.081560	0.832036
28	1	0	-0.649609	-2.031130	0.679240
29	1	0	-1.207486	-1.991624	2.361428
30	6	0	-1.213333	-0.108142	1.368795
31	6	0	-2.823728	0.065867	-0.653003
32	8	0	-3.483167	0.783786	-1.389265
33	8	0	-0.699116	0.484040	2.307377
34	6	0	-1.681322	0.677504	0.154476
35	1	0	-2.033428	1.650333	0.510142

E(RB3LYP/6-31G(d,p)) = -824.161924 Ha

ΔG (298.15 K, 1 atm, B3LYP/6-31G(d,p)) = -823.930825191 Ha

S2b (conformation 6)

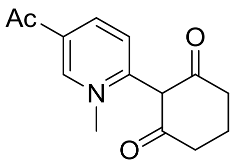

Center Number	Atomic Number	Atomic Type	Coordinates (Angstroms) X	Y	Z
1	6	0	-0.442280	-1.124089	0.943138
2	6	0	1.238734	-0.937046	-0.831313
3	6	0	2.148240	-0.318294	0.000141
4	6	0	0.696458	-0.846997	1.882435
5	1	0	-1.025811	-1.985272	1.285659
6	1	0	1.472202	-1.078485	-1.881902
7	1	0	0.503482	-0.974828	2.942419
8	7	0	0.065688	-1.441966	-0.399142
9	6	0	-0.639790	-2.457326	-1.182376
10	1	0	-0.501261	-3.446982	-0.732447
11	1	0	-1.709873	-2.250399	-1.227982
12	1	0	-0.239589	-2.473743	-2.196706
13	6	0	3.367312	0.232702	-0.597191
14	8	0	3.537615	0.269565	-1.821926
15	6	0	4.442228	0.769600	0.334122
16	1	0	4.045358	1.562746	0.977042
17	1	0	4.823140	-0.019032	0.992555
18	1	0	5.265098	1.165948	-0.262009
19	6	0	1.883946	-0.407977	1.426913
20	1	0	2.675569	-0.171884	2.130922
21	6	0	-2.665605	1.833283	-1.153898
22	6	0	-1.173196	1.946163	-0.795942
23	6	0	-3.134134	0.374062	-1.087896
24	1	0	-0.587565	1.340324	-1.500373
25	1	0	-0.813559	2.975972	-0.852970
26	1	0	-3.257720	2.449674	-0.467264
27	1	0	-2.835289	2.226896	-2.159751
28	1	0	-2.635072	-0.193676	-1.887460
29	1	0	-4.209566	0.271111	-1.254630
30	6	0	-2.794918	-0.324541	0.218449
31	6	0	-0.937346	1.433143	0.609733
32	8	0	-0.346276	2.085818	1.456136
33	8	0	-3.496525	-1.217043	0.670660
34	6	0	-1.522206	0.072864	0.970177
35	1	0	-1.792545	0.115393	2.030980

E(RB3LYP/6-31G(d,p)) = -824.157441095 Ha

ΔG (298.15 K, 1 atm, B3LYP/6-31G(d,p)) = -823.926235 Ha

S1a (conformation 1)

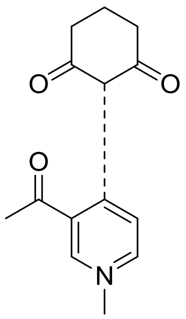

Center Number	Atomic Number	Atomic Type	X	Y	Z
1	6	0	-2.149757	-1.975547	-0.413389
2	6	0	-2.446190	0.303177	0.146167
3	6	0	-1.260154	0.646516	-0.465206
4	6	0	-0.959783	-1.710788	-1.014020
5	1	0	-2.606114	-2.957345	-0.409770
6	1	0	-3.117443	1.039716	0.568497
7	1	0	-0.427152	-2.513365	-1.506488
8	7	0	-2.873321	-0.981841	0.214022
9	6	0	-4.137178	-1.331599	0.879765
10	1	0	-3.937713	-1.967715	1.744875
11	1	0	-4.632144	-0.420237	1.210865
12	1	0	-4.788025	-1.860853	0.180914
13	6	0	-0.882494	2.062665	-0.696012
14	8	0	0.094066	2.324756	-1.395711
15	6	0	-1.729305	3.169741	-0.099866
16	1	0	-2.746774	3.153072	-0.504713
17	1	0	-1.796958	3.062759	0.986727
18	1	0	-1.270195	4.128840	-0.340946
19	6	0	-0.380314	-0.399431	-0.923771
20	1	0	0.376694	-0.109422	-1.640487
21	6	0	3.813400	0.178337	0.182417
22	6	0	3.253599	-1.026670	-0.585626
23	6	0	2.734796	1.249366	0.382848
24	1	0	2.978328	-0.715232	-1.605151
25	1	0	3.990281	-1.828723	-0.687614
26	1	0	4.183447	-0.156785	1.160014
27	1	0	4.671665	0.600338	-0.350878
28	1	0	2.454659	1.684071	-0.586239
29	1	0	3.087419	2.072995	1.011037
30	6	0	1.459182	0.692951	1.010401
31	6	0	2.010439	-1.609451	0.081283
32	8	0	1.784793	-2.828165	0.039249
33	8	0	0.734435	1.415443	1.714981
34	6	0	1.083569	-0.670835	0.692280
35	1	0	0.328070	-1.106096	1.339431

E(RB3LYP/6-31G(d,p)) = -824.150680032 Ha

ΔG (298.15 K, 1 atm, B3LYP/6-31G(d,p)) = -823.920473 Ha

S1a (conformation 2)

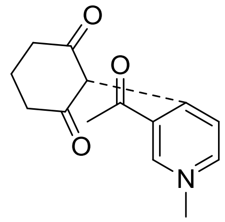

Center Number	Atomic Number	Atomic Type	Coordinates (Angstroms) X	Y	Z
1	6	0	-2.469146	-1.069326	-1.038735
2	6	0	-2.012682	0.738782	0.406600
3	6	0	-0.872669	1.064451	-0.298140
4	6	0	-1.358688	-0.804268	-1.775639
5	1	0	-3.178981	-1.843583	-1.300329
6	1	0	-2.373405	1.335796	1.234386
7	1	0	-1.172408	-1.394434	-2.664785
8	7	0	-2.785105	-0.323928	0.077892
9	6	0	-3.950616	-0.699776	0.889533
10	1	0	-4.801484	-0.897954	0.235857
11	1	0	-3.724960	-1.594174	1.475705
12	1	0	-4.199749	0.119939	1.562026
13	6	0	-0.146828	2.329707	-0.038076
14	8	0	0.849564	2.607363	-0.702911
15	6	0	-0.648973	3.281520	1.031666
16	1	0	-1.660899	3.633004	0.805695
17	1	0	-0.678160	2.796689	2.013086
18	1	0	0.023725	4.138165	1.077520
19	6	0	-0.411036	0.185264	-1.351641
20	1	0	0.275960	0.611113	-2.072134
21	6	0	2.611633	-0.813309	1.777781
22	6	0	1.140256	-1.249765	1.750806
23	6	0	1.268091	-1.115644	-0.779794
24	6	0	2.901260	0.210713	0.672960
25	1	0	0.498753	-0.385002	1.980924
26	1	0	0.925028	-2.016790	2.500253
27	1	0	3.252629	-1.693240	1.638676
28	1	0	2.863304	-0.392845	2.757071
29	1	0	1.165679	-1.650478	-1.720771
30	1	0	2.374705	1.149151	0.891052
31	1	0	3.966037	0.454146	0.608911
32	6	0	2.445714	-0.257446	-0.707489
33	6	0	0.729587	-1.791023	0.384851
34	8	0	-0.080202	-2.726598	0.289018
35	8	0	3.053706	0.099076	-1.725667

E(RB3LYP/6-31G(d,p)) = -824.147761040 Ha

ΔG (298.15 K, 1 atm, B3LYP/6-31G(d,p)) = -823.917881 Ha

S1a (conformation 3)

Center Number	Atomic Number	Atomic Type	Coordinates (Angstroms) X	Y	Z
1	6	0	-1.983996	-1.945317	0.618486
2	6	0	-2.482862	0.190700	-0.248680
3	6	0	-1.355004	0.739048	0.324111
4	6	0	-0.840260	-1.482871	1.182541
5	1	0	-2.351582	-2.953223	0.764197
6	1	0	-3.204815	0.824490	-0.748361
7	1	0	-0.260440	-2.147083	1.809295
8	7	0	-2.776781	-1.126860	-0.162194
9	6	0	-3.981932	-1.683878	-0.792834
10	1	0	-4.595786	-2.183074	-0.040299
11	1	0	-3.699392	-2.401531	-1.566116
12	1	0	-4.553346	-0.875097	-1.245260
13	6	0	-1.286247	2.227891	0.336334
14	8	0	-2.100271	2.895065	-0.299846
15	6	0	-0.248731	2.915553	1.201419
16	1	0	-0.107029	2.412521	2.162277
17	1	0	-0.568211	3.944874	1.371208
18	1	0	0.711307	2.932041	0.678695
19	6	0	-0.360340	-0.152573	0.886769
20	1	0	0.365156	0.265748	1.574095
21	6	0	3.885071	-0.123405	-0.058423
22	6	0	2.969310	1.102435	-0.158241
23	6	0	3.140569	-1.309525	0.567459
24	1	0	3.450234	1.935303	-0.679144
25	1	0	2.729733	1.460236	0.855066
26	1	0	4.776258	0.118317	0.529582
27	1	0	4.233482	-0.401167	-1.061171
28	1	0	3.753278	-2.215038	0.591190
29	1	0	2.882574	-1.070529	1.610903
30	6	0	1.847792	-1.632407	-0.174734
31	6	0	1.652960	0.801385	-0.871491
32	8	0	1.080675	1.682360	-1.531505
33	8	0	1.456536	-2.801780	-0.283053
34	6	0	1.059846	-0.511201	-0.674382
35	1	0	0.283475	-0.778945	-1.385570

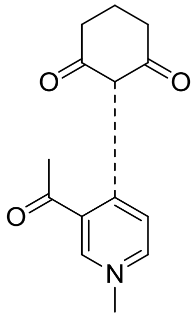

E(RB3LYP/6-31G(d,p)) = -824.146919812 Ha

ΔG (298.15 K, 1 atm, B3LYP/6-31G(d,p)) = -823.917038 Ha

⇨ Conformation of the acetyl group was not extensively studied since its variation does not change the energy significantly, e.g., if we compare **104a** (conformation 3) and **104a** (conformation 1).

S1a (conformation 4)

Center Number	Atomic Number	Atomic Type	Coordinates (Angstroms) X	Y	Z
1	6	0	0.754200	2.067842	-1.203039
2	6	0	2.179267	0.629810	0.014762
3	6	0	1.502820	-0.485733	-0.427074
4	6	0	0.030254	1.013618	-1.661032
5	1	0	0.571436	3.089573	-1.511120
6	1	0	3.056579	0.561210	0.644246
7	1	0	-0.762810	1.196011	-2.376362
8	7	0	1.815100	1.886500	-0.337678
9	6	0	2.534958	3.060880	0.174662
10	1	0	1.924294	3.582680	0.915659
11	1	0	3.465625	2.737230	0.638499
12	1	0	2.763295	3.738005	-0.650188
13	6	0	1.967018	-1.852939	-0.093085
14	8	0	1.365045	-2.825586	-0.543940
15	6	0	3.173964	-2.039688	0.804806
16	1	0	4.059838	-1.537077	0.404093
17	1	0	2.972558	-1.625811	1.798171
18	1	0	3.379759	-3.106386	0.896729
19	6	0	0.293529	-0.323693	-1.198265
20	1	0	-0.016346	-1.150265	-1.824821
21	6	0	-3.228396	0.868085	1.181267
22	6	0	-1.777317	0.948082	1.671925
23	6	0	-3.276845	0.527527	-0.313340
24	1	0	-1.272540	1.791226	1.174767
25	1	0	-1.714336	1.130877	2.748631
26	1	0	-3.764796	0.098099	1.749932
27	1	0	-3.744522	1.815325	1.369012
28	1	0	-2.843882	1.360988	-0.888319
29	1	0	-4.300447	0.394018	-0.675384
30	6	0	-2.481850	-0.726428	-0.664195
31	6	0	-0.983526	-0.314483	1.354351
32	8	0	-0.048812	-0.674766	2.089592
33	8	0	-2.811618	-1.430236	-1.629697
34	6	0	-1.290829	-1.009159	0.122121
35	1	0	-0.899806	-2.018818	0.039381

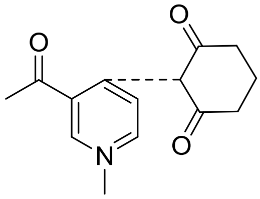

E(RB3LYP/6-31G(d,p)) = -824.151015942 Ha

ΔG (298.15 K, 1 atm, B3LYP/6-31G(d,p)) = -823.920991 Ha

S1b (conformation 1)

Center Number	Atomic Number	Atomic Type	Coordinates (Angstroms) X	Y	Z
1	6	0	-0.305448	1.107810	-1.031301
2	6	0	1.816007	1.038972	0.079240
3	6	0	2.143600	-0.177741	-0.475220
4	6	0	0.078048	-0.083871	-1.740427
5	1	0	-0.981213	1.800857	-1.518337
6	1	0	2.458557	1.554315	0.781368
7	1	0	-0.600434	-0.458508	-2.497423
8	7	0	0.679531	1.702437	-0.252997
9	6	0	0.400279	3.014519	0.349937
10	1	0	-0.137927	3.631933	-0.370440
11	1	0	-0.200236	2.871169	1.250817
12	1	0	1.342237	3.500577	0.601761
13	6	0	3.397531	-0.895531	-0.147134
14	8	0	3.654241	-1.962758	-0.700160
15	6	0	4.350103	-0.301737	0.872895
16	1	0	4.711832	0.680286	0.549957
17	1	0	3.859657	-0.170197	1.843012
18	1	0	5.200757	-0.973453	0.989780
19	6	0	1.244964	-0.721180	-1.451134
20	1	0	1.538194	-1.628132	-1.967471
21	6	0	-2.747338	-2.012327	0.904269
22	6	0	-2.988721	-1.471012	-0.510674
23	6	0	-1.417923	-1.492203	1.467129
24	1	0	-2.234686	-1.892439	-1.193347
25	1	0	-3.966452	-1.762068	-0.905426
26	1	0	-3.569043	-1.701642	1.561577
27	1	0	-2.749305	-3.106953	0.893095
28	1	0	-0.587486	-1.885299	0.861016
29	1	0	-1.246296	-1.821598	2.495800
30	6	0	-1.329009	0.028674	1.433085
31	6	0	-2.878523	0.047494	-0.595397
32	8	0	-3.505255	0.680702	-1.456065
33	8	0	-0.694477	0.648182	2.301335
34	6	0	-1.946337	0.709007	0.307906
35	1	0	-2.134341	1.769835	0.455321

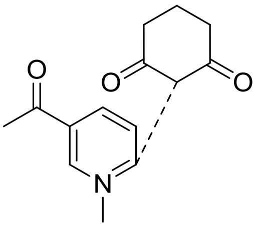

E(RB3LYP/6-31G(d,p)) = -824.151149141 Ha

ΔG (298.15 K, 1 atm, B3LYP/6-31G(d,p)) = -823.920837 Ha

S1b (conformation 2)

Center Number	Atomic Number	Atomic Type	Coordinates (Angstroms) X	Y	Z
1	6	0	-0.311379	1.086849	-1.059299
2	6	0	1.774657	1.131866	0.109090
3	6	0	2.164975	-0.092704	-0.389901
4	6	0	0.138025	-0.109954	-1.717052
5	1	0	-0.993601	1.739037	-1.591789
6	1	0	2.403110	1.666871	0.810294
7	1	0	-0.501353	-0.533112	-2.482163
8	7	0	0.632241	1.744126	-0.274821
9	6	0	0.294173	3.067999	0.269357
10	1	0	-0.230420	3.645604	-0.492928
11	1	0	-0.338340	2.938394	1.150089
12	1	0	1.212587	3.586445	0.542097
13	6	0	3.453680	-0.656829	0.081637
14	8	0	4.128468	-0.068409	0.924587
15	6	0	3.913855	-1.977426	-0.500396
16	1	0	3.175796	-2.765270	-0.314441
17	1	0	4.042955	-1.903021	-1.585758
18	1	0	4.863509	-2.256235	-0.043372
19	6	0	1.318847	-0.699088	-1.373800
20	1	0	1.630391	-1.608885	-1.874373
21	6	0	-2.686287	-2.043409	0.936847
22	6	0	-2.903258	-1.571315	-0.506442
23	6	0	-1.394522	-1.449830	1.514133
24	1	0	-2.111010	-1.989455	-1.146737
25	1	0	-3.855436	-1.917784	-0.918485
26	1	0	-3.538347	-1.736484	1.556124
27	1	0	-2.647869	-3.136827	0.972839
28	1	0	-0.532928	-1.834088	0.947162
29	1	0	-1.240047	-1.730227	2.559833
30	6	0	-1.363319	0.070634	1.419607
31	6	0	-2.849271	-0.054372	-0.652143
32	8	0	-3.475569	0.517928	-1.554455
33	8	0	-0.785153	0.749680	2.282316
34	6	0	-1.967402	0.680167	0.246611
35	1	0	-2.203296	1.737028	0.346276

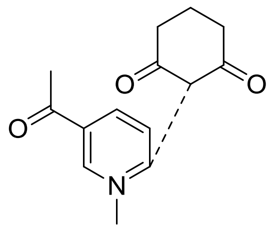

E(RB3LYP/6-31G(d,p)) = -824.150847097 Ha

ΔG (298.15 K, 1 atm, B3LYP/6-31G(d,p)) =-823.920285 Ha

S1c (conformation 1)

Center Number	Atomic Number	Atomic Type	Coordinates (Angstroms) X	Y	Z
1	6	0	1.201668	2.595260	-0.307016
2	6	0	0.610358	0.478470	0.605946
3	6	0	1.810784	-0.090360	0.027641
4	6	0	2.126698	2.010576	-1.143605
5	1	0	0.990992	3.657660	-0.320818
6	1	0	0.191698	0.021571	1.493288
7	1	0	2.634212	2.614571	-1.884504
8	7	0	0.551143	1.871481	0.634005
9	6	0	-0.238673	2.533741	1.680773
10	1	0	0.331570	2.553834	2.613633
11	1	0	-0.467091	3.551927	1.369704
12	1	0	-1.172092	1.996325	1.839132
13	6	0	2.337851	-1.435211	0.388269
14	8	0	3.291703	-1.909552	-0.224110
15	6	0	1.738063	-2.173255	1.569086
16	1	0	1.724361	-1.542947	2.464777
17	1	0	0.710349	-2.478662	1.357087
18	1	0	2.336441	-3.063803	1.764790
19	6	0	2.496768	0.673285	-0.896180
20	1	0	3.359125	0.236767	-1.388687
21	6	0	-3.418716	-1.325397	-0.006711
22	6	0	-2.108732	-2.113798	0.112596
23	6	0	-3.203081	0.145800	0.368209
24	1	0	-1.801054	-2.150170	1.169026
25	1	0	-2.219002	-3.149949	-0.219294
26	1	0	-3.789174	-1.387985	-1.037716
27	1	0	-4.187566	-1.770252	0.632888
28	1	0	-2.943851	0.212919	1.435763
29	1	0	-4.105546	0.746053	0.222968
30	6	0	-2.076386	0.789513	-0.436270
31	6	0	-0.972769	-1.484195	-0.690739
32	8	0	-0.094880	-2.196523	-1.199599
33	8	0	-2.128359	1.987290	-0.748097
34	6	0	-0.916585	-0.031182	-0.769441
35	1	0	-0.322564	0.362575	-1.590973

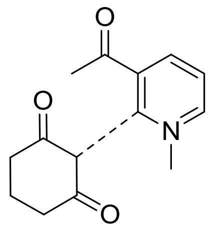

E(RB3LYP/6-31G(d,p)) = -824.145510180 Ha

ΔG (298.15 K, 1 atm, B3LYP/6-31G(d,p)) = -823.914571 Ha

S1c (conformation 2)

Center Number	Atomic Number	Atomic Type	X	Y	Z
1	6	0	1.566212	2.497433	-0.455396
2	6	0	0.599136	0.611813	0.619555
3	6	0	1.708150	-0.195783	0.173941
4	6	0	2.452231	1.696004	-1.138663
5	1	0	1.522205	3.570442	-0.597840
6	1	0	0.081206	0.289832	1.512558
7	1	0	3.103174	2.134189	-1.883867
8	7	0	0.734754	1.985641	0.486294
9	6	0	-0.003917	2.878722	1.393760
10	1	0	0.528047	2.951414	2.346404
11	1	0	-0.076922	3.867460	0.942615
12	1	0	-1.006787	2.492719	1.556610
13	6	0	1.823087	-1.560337	0.741556
14	8	0	1.068071	-1.910784	1.648484
15	6	0	2.894465	-2.489586	0.212108
16	1	0	2.760506	-2.651096	-0.861299
17	1	0	3.894103	-2.070775	0.367390
18	1	0	2.822631	-3.443819	0.734788
19	6	0	2.579422	0.344301	-0.749621
20	1	0	3.391556	-0.248155	-1.156605
21	6	0	-3.333481	-1.335571	0.052751
22	6	0	-1.987662	-2.070240	0.019620
23	6	0	-3.151053	0.124806	0.486729
24	1	0	-1.584598	-2.159573	1.038339
25	1	0	-2.085126	-3.085971	-0.374787
26	1	0	-3.788267	-1.362259	-0.945830
27	1	0	-4.027504	-1.845926	0.728522
28	1	0	-2.806518	0.153131	1.531633
29	1	0	-4.088527	0.686535	0.445525
30	6	0	-2.123393	0.860246	-0.370324
31	6	0	-0.941617	-1.339208	-0.816561
32	8	0	-0.070208	-1.973483	-1.432504
33	8	0	-2.269748	2.062342	-0.638108
34	6	0	-0.952077	0.113360	-0.803787
35	1	0	-0.384396	0.581992	-1.605196

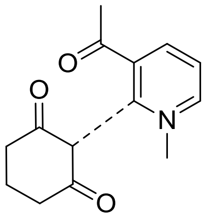

E(RB3LYP/6-31G(d,p)) = -824.150110214 Ha

ΔG (298.15 K, 1 atm, B3LYP/6-31G(d,p)) = -823.919229214 Ha

Reaction intermediates and transition states

K-S1a (conformation 1)

Center Number	Atomic Number	Atomic Type	X	Y	Z
1	6	0	-0.202490	2.527079	-0.848920
2	6	0	1.845663	1.890695	0.146060
3	6	0	1.762385	0.629126	-0.398109
4	6	0	-0.361227	1.293571	-1.396319
5	1	0	-0.872624	3.354060	-1.049361
6	1	0	2.711896	2.229958	0.699290
7	1	0	-1.193935	1.117186	-2.064644
8	7	0	0.865606	2.813941	-0.023234
9	6	0	0.964885	4.150354	0.582036
10	1	0	0.884782	4.914042	-0.194065
11	1	0	0.165529	4.288461	1.313495
12	1	0	1.927593	4.245756	1.081579
13	6	0	2.911076	-0.308997	-0.376967
14	8	0	2.849736	-1.357525	-1.016449
15	6	0	4.158499	0.050804	0.405041
16	1	0	4.623175	0.963388	0.017629
17	1	0	3.917284	0.217317	1.459243
18	1	0	4.870726	-0.770584	0.323358
19	6	0	0.532501	0.219485	-1.040297
20	1	0	0.610512	-0.629172	-1.708295
21	6	0	-0.917824	-3.706378	0.062244
22	6	0	0.466828	-3.183679	0.463722
23	6	0	-0.559819	-0.840385	0.456761
24	6	0	-1.630291	-2.721218	-0.876378
25	1	0	1.129314	-3.166544	-0.412347
26	1	0	0.943912	-3.822134	1.212800
27	1	0	-1.529860	-3.854164	0.961132
28	1	0	-0.827188	-4.683838	-0.421673
29	1	0	-0.724193	0.054733	1.050411
30	1	0	-1.077387	-2.652464	-1.825783
31	1	0	-2.644711	-3.047572	-1.121130
32	6	0	-1.712010	-1.326124	-0.277052
33	6	0	0.424530	-1.762868	1.014960
34	8	0	1.242542	-1.382694	1.863158
35	8	0	-2.718524	-0.617170	-0.471392
36	19	0	-4.003175	1.417595	0.544093

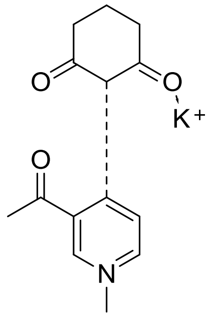

E(RB3LYP/6-31G(d,p)) = -1424.01184673 Ha

ΔG (298.15 K, 1 atm, B3LYP/6-31G(d,p)) = -1423.785917 Ha

K-S1a (conformation 2)

Center Number	Atomic Number	Atomic Type	X	Y	Z
1	6	0	2.090249	-0.856967	-1.811242
2	6	0	2.795739	0.761505	-0.250976
3	6	0	1.523213	1.277332	-0.140505
4	6	0	0.807789	-0.425874	-1.725575
5	1	0	2.409047	-1.621880	-2.508261
6	1	0	3.621501	1.241530	0.259303
7	1	0	0.069672	-0.855040	-2.392382
8	7	0	3.089779	-0.300510	-1.033533
9	6	0	4.453494	-0.840951	-1.112830
10	1	0	5.134760	-0.166939	-0.595728
11	1	0	4.494570	-1.826008	-0.641791
12	1	0	4.756618	-0.924807	-2.158260
13	6	0	1.387181	2.554136	0.611800
14	8	0	2.336209	3.022000	1.237078
15	6	0	0.069569	3.299616	0.540794
16	1	0	-0.183490	3.529964	-0.500346
17	1	0	-0.756498	2.709196	0.949170
18	1	0	0.158176	4.228135	1.105350
19	6	0	0.411983	0.569607	-0.747801
20	1	0	-0.487273	1.131907	-0.978106
21	6	0	-1.094247	-3.218359	0.826708
22	6	0	0.328632	-2.691210	1.058511
23	6	0	-0.632937	-0.341820	0.807052
24	6	0	-1.840456	-2.352831	-0.198128
25	1	0	0.908801	-2.791750	0.128572
26	1	0	0.857566	-3.256810	1.830278
27	1	0	-1.644840	-3.217224	1.775325
28	1	0	-1.059920	-4.256444	0.482373
29	1	0	-0.797177	0.597563	1.327305
30	1	0	-1.363167	-2.463598	-1.183969
31	1	0	-2.883496	-2.659700	-0.316964
32	6	0	-1.816658	-0.874484	0.152570
33	6	0	0.343819	-1.220130	1.451300
34	8	0	1.189613	-0.771096	2.231589
35	8	0	-2.758935	-0.132308	-0.178612
36	19	0	-4.945401	1.153647	-0.654612

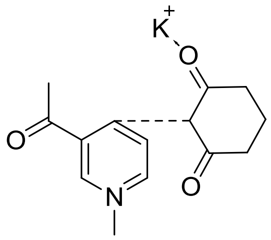

E(RB3LYP/6-31G(d,p)) = -1424.00775736 Ha

ΔG (298.15 K, 1 atm, B3LYP/6-31G(d,p)) = -1423.784717 Ha

K-S1a (conformation 3), **K-S1a-1**

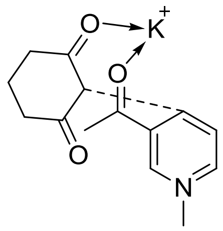

Center Number	Atomic Number	Atomic Type	X	Y	Z
1	6	0	2.880825	-0.418252	-1.457230
2	6	0	2.000072	-1.395321	0.501823
3	6	0	0.706234	-1.238101	0.043761
4	6	0	1.636650	-0.225167	-1.964560
5	1	0	3.784368	-0.193810	-2.009420
6	1	0	2.229100	-1.906697	1.427761
7	1	0	1.538829	0.174921	-2.966272
8	7	0	3.069224	-0.959769	-0.200613
9	6	0	4.434932	-1.076575	0.331485
10	1	0	5.067752	-1.595014	-0.390988
11	1	0	4.841728	-0.081727	0.526071
12	1	0	4.409983	-1.643504	1.260732
13	6	0	-0.424749	-1.889058	0.731922
14	8	0	-1.571140	-1.743463	0.296116
15	6	0	-0.180707	-2.751789	1.952547
16	1	0	0.460362	-3.605239	1.709856
17	1	0	0.311328	-2.182998	2.748107
18	1	0	-1.139630	-3.118787	2.318728
19	6	0	0.467167	-0.472695	-1.165421
20	1	0	-0.478881	-0.647752	-1.663552
21	6	0	-0.690263	2.597032	1.839014
22	6	0	0.742209	2.250623	1.411873
23	6	0	-0.226595	1.541231	-0.832958
24	6	0	-1.691538	1.589829	1.259021
25	1	0	1.023884	1.276550	1.840551
26	1	0	1.469040	2.983330	1.772751
27	1	0	-0.941402	3.605847	1.488685
28	1	0	-0.766580	2.613210	2.930733
29	1	0	-0.228168	1.721568	-1.905110
30	1	0	-1.533977	0.606526	1.724125
31	1	0	-2.726859	1.875092	1.468299
32	6	0	-1.549599	1.409607	-0.246644
33	6	0	0.876087	2.154853	-0.103409
34	8	0	1.906441	2.525882	-0.679235
35	8	0	-2.542189	1.126203	-0.943837
36	19	0	-3.949194	-1.099442	-0.740446

E(RB3LYP/6-31G(d,p)) = -1424.01549029 Ha

ΔG (298.15 K, 1 atm, B3LYP/6-31G(d,p)) = -1423.788817 Ha

K-S1a (conformation 4), **K-S1a-2**

Center Number	Atomic Number	Atomic Type	Coordinates (Angstroms) X	Y	Z
1	6	0	2.536870	0.590862	-1.382200
2	6	0	2.317227	-1.315928	-0.012274
3	6	0	0.950730	-1.348912	-0.210785
4	6	0	1.199205	0.629003	-1.605800
5	1	0	3.234840	1.265139	-1.862089
6	1	0	2.835509	-2.076184	0.557683
7	1	0	0.808783	1.358572	-2.304783
8	7	0	3.101819	-0.359409	-0.551477
9	6	0	4.548609	-0.318421	-0.293871
10	1	0	5.088428	-0.276597	-1.241603
11	1	0	4.794014	0.560484	0.306532
12	1	0	4.840762	-1.217071	0.246896
13	6	0	0.136969	-2.475070	0.282655
14	8	0	-1.088408	-2.457084	0.122229
15	6	0	0.794532	-3.647325	0.978312
16	1	0	1.544664	-4.120711	0.337458
17	1	0	1.298012	-3.323639	1.895556
18	1	0	0.027572	-4.378404	1.234320
19	6	0	0.303583	-0.259809	-0.910088
20	1	0	-0.660401	-0.469901	-1.357936
21	6	0	-0.366898	3.539289	-0.177785
22	6	0	-1.785405	2.969999	-0.305208
23	6	0	0.321906	3.062295	1.106613
24	1	0	-2.436825	3.414185	0.462272
25	1	0	-2.237574	3.213113	-1.271938
26	1	0	0.227308	3.235236	-1.046152
27	1	0	-0.402486	4.633078	-0.198338
28	1	0	-0.145320	3.542261	1.979034
29	1	0	1.379190	3.345782	1.131037
30	6	0	0.226879	1.558289	1.347904
31	6	0	-1.854778	1.463140	-0.106499
32	8	0	-2.819814	0.821995	-0.568468
33	8	0	0.994372	1.006207	2.145372
34	6	0	-0.782769	0.805419	0.613517
35	1	0	-1.051002	-0.151366	1.049322
36	19	0	-3.622494	-1.711792	-0.294576

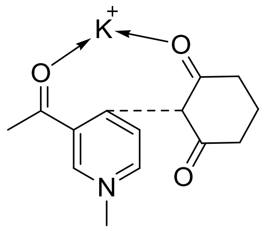

E(RB3LYP/6-31G(d,p)) = -1424.01559626 Ha

ΔG (298.15 K, 1 atm, B3LYP/6-31G(d,p)) = -1423.788319 Ha

K-S1a (conformation 5), **K-S1a-3**

Center Number	Atomic Number	Atomic Type	Coordinates (Angstroms) X	Y	Z
1	6	0	0.858327	3.021506	0.179859
2	6	0	2.135142	1.037695	0.154197
3	6	0	1.155908	0.347975	0.838862
4	6	0	-0.162944	2.398347	0.817408
5	1	0	0.867791	4.084686	-0.024092
6	1	0	3.091805	0.575399	-0.054942
7	1	0	-1.013741	2.982957	1.140479
8	7	0	1.985464	2.327734	-0.219758
9	6	0	3.049408	3.044668	-0.938809
10	1	0	3.372185	3.909645	-0.355747
11	1	0	2.678379	3.377732	-1.910312
12	1	0	3.894097	2.373921	-1.086857
13	6	0	1.529486	-0.984631	1.368272
14	8	0	2.513058	-1.588252	0.923107
15	6	0	0.727654	-1.576473	2.507632
16	1	0	0.413359	-0.819325	3.230814
17	1	0	1.335484	-2.333688	3.005095
18	1	0	-0.169959	-2.063452	2.114834
19	6	0	-0.150208	0.962362	0.998626
20	1	0	-0.799532	0.568648	1.772277
21	6	0	-3.903221	-0.933471	-0.096364
22	6	0	-2.634541	-1.694815	0.309720
23	6	0	-3.724152	0.575981	0.111001
24	1	0	-2.706725	-2.763604	0.090243
25	1	0	-2.490936	-1.604194	1.397308
26	1	0	-4.758062	-1.296414	0.482104
27	1	0	-4.126337	-1.132788	-1.151843
28	1	0	-4.592513	1.145108	-0.231249
29	1	0	-3.604384	0.787291	1.184722
30	6	0	-2.494344	1.110338	-0.612126
31	6	0	-1.385293	-1.157190	-0.374481
32	8	0	-0.439771	-1.924228	-0.640770
33	8	0	-2.490907	2.235493	-1.119872
34	6	0	-1.292867	0.269104	-0.619995
35	1	0	-0.544775	0.562513	-1.351271
36	19	0	1.912971	-2.787424	-1.467147

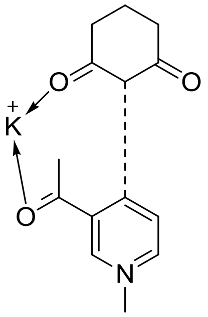

E(RB3LYP/6-31G(d,p)) = -1424.01289494 Ha

ΔG (298.15 K, 1 atm, B3LYP/6-31G(d,p)) = -1423.786309 Ha

K-S1b (conformation 1)

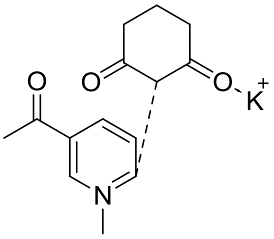

Center Number	Atomic Number	Atomic Type	Coordinates (Angstroms) X	Y	Z
1	6	0	-0.009190	-1.146608	-0.095843
2	6	0	-2.337107	-1.142746	0.494933
3	6	0	-2.704203	-0.509492	-0.670330
4	6	0	-0.393864	-0.642951	-1.395071
5	1	0	0.856056	-1.799754	-0.048261
6	1	0	-3.047760	-1.397682	1.270683
7	1	0	0.383668	-0.531808	-2.141620
8	7	0	-1.060350	-1.537773	0.733825
9	6	0	-0.740680	-2.247499	1.980850
10	1	0	0.063295	-2.960262	1.791349
11	1	0	-0.433212	-1.523617	2.738858
12	1	0	-1.622486	-2.789052	2.321649
13	6	0	-4.098554	-0.100782	-0.956075
14	8	0	-4.375921	0.417150	-2.035923
15	6	0	-5.168844	-0.320915	0.095841
16	1	0	-5.265549	-1.382457	0.346755
17	1	0	-4.928925	0.216094	1.019562
18	1	0	-6.121011	0.041445	-0.292160
19	6	0	-1.683726	-0.306693	-1.658628
20	1	0	-1.979228	0.087647	-2.624238
21	6	0	1.249952	3.034436	0.096497
22	6	0	-0.078565	2.516393	0.663346
23	6	0	1.080459	0.263642	0.977226
24	6	0	1.953792	1.948031	-0.728150
25	1	0	-0.767746	2.298186	-0.166560
26	1	0	-0.570911	3.252471	1.304420
27	1	0	1.902215	3.350355	0.919846
28	1	0	1.074659	3.917787	-0.524902
29	1	0	1.404160	-0.457213	1.724617
30	1	0	1.352187	1.725255	-1.623132
31	1	0	2.938634	2.267565	-1.080366
32	6	0	2.119566	0.644838	0.033903
33	6	0	0.092448	1.230224	1.458719
34	8	0	-0.621552	0.981205	2.437275
35	8	0	3.079580	-0.109803	-0.202665
36	19	0	5.359929	-1.219907	-0.707689

E(RB3LYP/6-31G(d,p)) = -1424.01116158 Ha

ΔG (298.15 K, 1 atm, B3LYP/6-31G(d,p)) = -1423.785542 Ha

K-S1b (conformation 2)

Center Number	Atomic Number	Atomic Type	Coordinates (Angstroms) X	Y	Z
1	6	0	0.041522	-1.178254	-0.185031
2	6	0	2.386741	-1.194552	0.318790
3	6	0	2.696208	-0.355844	-0.728833
4	6	0	0.367704	-0.452755	-1.393942
5	1	0	-0.795057	-1.867664	-0.237648
6	1	0	3.159149	-1.534264	0.998020
7	1	0	-0.441726	-0.237263	-2.081880
8	7	0	1.139121	-1.676011	0.525229
9	6	0	0.892894	-2.615109	1.628076
10	1	0	1.808104	-3.166997	1.839107
11	1	0	0.583367	-2.056834	2.514705
12	1	0	0.112462	-3.318651	1.333645
13	6	0	4.103522	0.085119	-0.874536
14	8	0	4.956990	-0.239948	-0.050280
15	6	0	4.463443	0.948849	-2.066153
16	1	0	4.255594	0.424578	-3.005419
17	1	0	3.874242	1.872205	-2.072959
18	1	0	5.524062	1.196759	-2.019391
19	6	0	1.636545	-0.031795	-1.641452
20	1	0	1.850382	0.523064	-2.548365
21	6	0	-0.785823	2.601405	0.070926
22	6	0	-0.156478	2.342679	1.444699
23	6	0	-1.043218	-0.048399	1.147458
24	6	0	-2.154729	1.921007	-0.057992
25	1	0	-0.740528	2.850321	2.226596
26	1	0	0.860156	2.741958	1.510494
27	1	0	-0.117369	2.226620	-0.711348
28	1	0	-0.890558	3.677697	-0.096474
29	1	0	-1.304387	-0.919948	1.743199
30	1	0	-2.884568	2.416486	0.599333
31	1	0	-2.554067	1.998406	-1.074820
32	6	0	-2.149253	0.454447	0.344094
33	6	0	-0.120187	0.867693	1.825241
34	8	0	0.665996	0.456620	2.685591
35	8	0	-3.062066	-0.302904	-0.028343
36	19	0	-5.359674	-0.761657	-1.180907

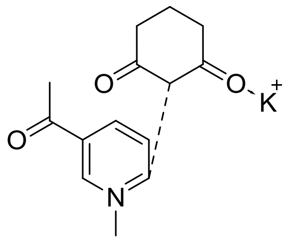

E(RB3LYP/6-31G(d,p)) = -1424.00844130 Ha

ΔG (298.15 K, 1 atm, B3LYP/6-31G(d,p)) = -1423.782991 Ha

K-S1b (conformation 3)

Center Number	Atomic Number	Atomic Type	X	Y	Z
1	6	0	0.542201	-1.372258	0.978860
2	6	0	-1.486159	-0.174051	1.226450
3	6	0	-2.107737	-0.944675	0.261260
4	6	0	-0.167794	-2.387036	0.242190
5	1	0	1.451116	-1.644256	1.500321
6	1	0	-1.995444	0.656894	1.698688
7	1	0	0.371354	-3.273597	-0.062483
8	7	0	-0.249445	-0.463773	1.675621
9	6	0	0.314679	0.220838	2.846303
10	1	0	0.411618	-0.488769	3.671244
11	1	0	1.296255	0.625899	2.602863
12	1	0	-0.346805	1.033509	3.141948
13	6	0	-3.406353	-0.466581	-0.261226
14	8	0	-3.709409	0.726245	-0.149812
15	6	0	-4.325882	-1.445065	-0.950477
16	1	0	-3.866793	-1.802018	-1.879700
17	1	0	-4.512200	-2.322169	-0.322816
18	1	0	-5.267736	-0.950217	-1.188022
19	6	0	-1.458916	-2.159920	-0.133186
20	1	0	-1.989833	-2.888536	-0.735560
21	6	0	4.163760	0.794678	-0.467462
22	6	0	3.941908	-0.723675	-0.480546
23	6	0	1.440385	-0.226965	-0.586163
24	6	0	3.077673	1.502483	0.353412
25	1	0	4.054932	-1.120368	0.539913
26	1	0	4.674608	-1.240980	-1.105660
27	1	0	4.147738	1.173730	-1.496949
28	1	0	5.151945	1.028600	-0.059825
29	1	0	0.539760	-0.320545	-1.186188
30	1	0	3.195034	1.235402	1.413699
31	1	0	3.160901	2.591293	0.294052
32	6	0	1.670190	1.115367	-0.085375
33	6	0	2.550685	-1.095062	-0.983225
34	8	0	2.367530	-2.118056	-1.651031
35	8	0	0.732859	1.929881	0.029676
36	19	0	-1.702632	2.674711	-0.850022

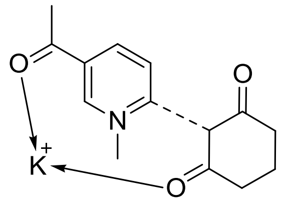

E(RB3LYP/6-31G(d,p)) = -1424.01026770 Ha

ΔG (298.15 K, 1 atm, B3LYP/6-31G(d,p)) = -1423.784129 Ha

K-S1b (conformation 4)

Center Number	Atomic Number	Atomic Type	X	Y	Z
1	6	0	-0.692728	1.236697	0.934809
2	6	0	1.361270	1.948954	-0.076582
3	6	0	2.048225	0.872767	0.447403
4	6	0	0.073249	0.314949	1.736488
5	1	0	-1.595659	1.658460	1.360412
6	1	0	1.819263	2.660996	-0.751772
7	1	0	-0.442292	-0.240342	2.511419
8	7	0	0.068150	2.188668	0.235880
9	6	0	-0.625160	3.372591	-0.295855
10	1	0	-1.434695	3.057388	-0.957578
11	1	0	-1.043079	3.946858	0.534807
12	1	0	0.089151	3.992831	-0.835153
13	6	0	3.397889	0.495195	-0.004609
14	8	0	3.828059	-0.635582	0.254394
15	6	0	4.232202	1.471493	-0.804716
16	1	0	3.774855	1.655188	-1.783260
17	1	0	4.316872	2.435138	-0.293628
18	1	0	5.224944	1.047533	-0.957239
19	6	0	1.383213	0.092826	1.451739
20	1	0	1.960637	-0.637080	2.008776
21	6	0	-3.870329	-1.714409	0.006976
22	6	0	-4.101537	-0.209305	0.184262
23	6	0	-1.666924	0.144107	-0.544529
24	6	0	-2.444904	-2.097329	0.422735
25	1	0	-4.052319	0.044354	1.254535
26	1	0	-5.086580	0.104356	-0.171074
27	1	0	-4.031232	-1.989562	-1.042545
28	1	0	-4.597805	-2.279345	0.597404
29	1	0	-1.054164	0.562298	-1.339805
30	1	0	-2.321343	-1.934515	1.504724
31	1	0	-2.228766	-3.152482	0.234379
32	6	0	-1.387057	-1.265384	-0.281750
33	6	0	-3.055451	0.636146	-0.529608
34	8	0	-3.362365	1.728400	-1.017162
35	8	0	-0.281119	-1.760707	-0.549201
36	19	0	2.149306	-2.773970	-0.468968

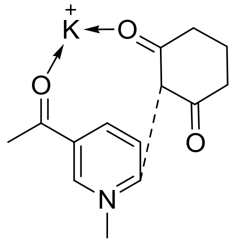

E(RB3LYP/6-31G(d,p)) = -1424.01281044 Ha

ΔG (298.15 K, 1 atm, B3LYP/6-31G(d,p)) = -1423.785902 Ha

K-S1b (conformation 5)

Center Number	Atomic Number	Atomic Type	Coordinates (Angstroms) X	Y	Z
1	6	0	-0.962043	1.122776	-1.339473
2	6	0	0.485097	1.949216	0.382416
3	6	0	1.575714	1.308619	-0.166733
4	6	0	0.205452	0.724769	-2.079375
5	1	0	-1.867029	1.385615	-1.875761
6	1	0	0.532622	2.497036	1.315778
7	1	0	0.068497	0.327467	-3.077966
8	7	0	-0.723927	1.939382	-0.229294
9	6	0	-1.815049	2.793953	0.270357
10	1	0	-1.827469	3.735743	-0.284687
11	1	0	-2.768689	2.287261	0.133965
12	1	0	-1.661191	3.000590	1.329103
13	6	0	2.848092	1.134237	0.559707
14	8	0	3.654328	0.284321	0.165208
15	6	0	3.140814	1.964747	1.789377
16	1	0	3.007192	3.032399	1.591393
17	1	0	2.462510	1.692037	2.605564
18	1	0	4.165404	1.775059	2.109919
19	6	0	1.427263	0.759966	-1.484675
20	1	0	2.313684	0.415662	-2.005822
21	6	0	-2.460624	-1.699105	1.833504
22	6	0	-3.302363	-0.548461	1.270628
23	6	0	-1.839946	-0.715455	-0.836879
24	6	0	-0.989552	-1.549690	1.420494
25	1	0	-3.006131	0.390691	1.760805
26	1	0	-4.369663	-0.679510	1.469753
27	1	0	-2.847594	-2.655476	1.460617
28	1	0	-2.543546	-1.727036	2.924203
29	1	0	-1.903533	-0.967595	-1.893056
30	1	0	-0.576812	-0.633520	1.865957
31	1	0	-0.379072	-2.384450	1.776334
32	6	0	-0.840168	-1.465665	-0.092988
33	6	0	-3.131796	-0.360953	-0.234664
34	8	0	-4.040396	0.126932	-0.916144
35	8	0	0.129666	-1.993044	-0.668075
36	19	0	2.751272	-2.364581	-0.315743

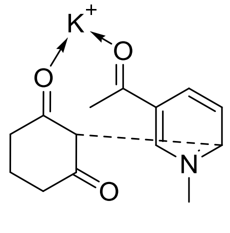

E(RB3LYP/6-31G(d,p)) = -1424.01111943 Ha

ΔG (298.15 K, 1 atm, B3LYP/6-31G(d,p)) = -1423.784631 Ha

K-S1b (conformation 6)

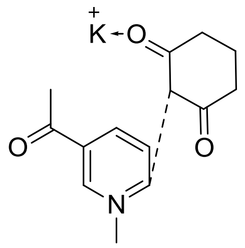

Center Number	Atomic Number	Atomic Type	X	Y	Z
1	6	0	-0.616826	-1.269629	0.695312
2	6	0	1.529110	-1.698379	-0.299923
3	6	0	2.173638	-0.802280	0.528639
4	6	0	0.081128	-0.519848	1.720116
5	1	0	-1.485924	-1.839933	1.005445
6	1	0	2.076518	-2.218855	-1.076425
7	1	0	-0.502344	-0.134260	2.548446
8	7	0	0.225558	-2.014345	-0.156063
9	6	0	-0.380486	-3.072349	-0.977630
10	1	0	-0.837537	-3.818238	-0.321659
11	1	0	-1.151760	-2.647233	-1.622904
12	1	0	0.396106	-3.549609	-1.573582
13	6	0	3.608091	-0.534587	0.283167
14	8	0	4.188383	-1.023232	-0.686971
15	6	0	4.343644	0.370860	1.251213
16	1	0	3.886230	1.366462	1.275104
17	1	0	4.305595	-0.027395	2.270755
18	1	0	5.383936	0.459261	0.936892
19	6	0	1.411547	-0.258520	1.616390
20	1	0	1.902338	0.333208	2.381264
21	6	0	-3.682359	0.760205	1.335982
22	6	0	-4.130710	-0.013433	0.092364
23	6	0	-1.628645	0.012043	-0.510459
24	6	0	-2.669401	1.846617	0.961493
25	1	0	-4.692058	0.652820	-0.579057
26	1	0	-4.802606	-0.841164	0.338715
27	1	0	-3.237049	0.068767	2.061894
28	1	0	-4.547583	1.210041	1.831793
29	1	0	-0.963414	-0.114323	-1.362375
30	1	0	-3.166974	2.644509	0.391521
31	1	0	-2.234674	2.328096	1.843875
32	6	0	-1.528004	1.342962	0.091844
33	6	0	-2.969534	-0.574167	-0.718956
34	8	0	-3.157755	-1.502627	-1.508373
35	8	0	-0.516944	2.044165	-0.056728
36	19	0	1.617666	2.660255	-1.455684

E(RB3LYP/6-31G(d,p)) = -1424.00790564 Ha

ΔG (298.15 K, 1 atm, B3LYP/6-31G(d,p)) = -1423.783622 Ha

K-S1b (conformation 7)

Center Number	Atomic Number	Atomic Type	Coordinates (Angstroms) X	Y	Z
1	6	0	-1.546365	-1.299290	-0.259378
2	6	0	-0.508625	-1.934753	-1.020058
3	6	0	0.743607	-2.038953	-0.495926
4	6	0	-1.274088	-1.010244	1.061134
5	1	0	-0.719389	-2.327954	-2.008398
6	1	0	1.553491	-2.500243	-1.048304
7	1	0	-2.046123	-0.642959	1.725949
8	7	0	-0.058824	-1.223617	1.611274
9	6	0	0.151222	-0.939825	3.037665
10	1	0	0.951538	-1.575526	3.416202
11	1	0	0.418503	0.112468	3.164785
12	1	0	-0.765897	-1.155274	3.584777
13	6	0	1.042353	-1.491108	0.804374
14	1	0	1.907423	-1.874198	1.333313
15	6	0	2.056518	0.371589	0.679091
16	1	0	2.169904	0.393630	1.759919
17	6	0	3.452348	0.548445	-1.459422
18	6	0	1.227708	1.759878	-1.301034
19	6	0	2.117520	0.845986	-2.153275
20	1	0	4.056106	-0.168942	-2.023383
21	1	0	4.048117	1.470422	-1.385724
22	1	0	0.225352	1.857120	-1.731531
23	1	0	1.650484	2.774977	-1.272481
24	1	0	2.297922	1.309551	-3.128096
25	1	0	1.590821	-0.094495	-2.347580
26	6	0	1.094191	1.318177	0.150352
27	6	0	3.284996	0.025308	-0.038612
28	8	0	4.154390	-0.677416	0.487690
29	8	0	0.167995	1.764131	0.858120
30	6	0	-2.875566	-0.926363	-0.783535
31	6	0	-3.366693	-1.566904	-2.059527
32	1	0	-2.756091	-1.232071	-2.906325
33	1	0	-3.288658	-2.657454	-2.013665
34	1	0	-4.402173	-1.274475	-2.234947
35	8	0	-3.556238	-0.078747	-0.193677
36	19	0	-2.431763	2.427123	0.507812

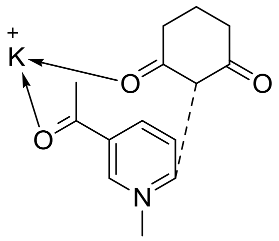

E(RB3LYP/6-31G(d,p)) = -1424.01098730 Ha

ΔG (298.15 K, 1 atm, B3LYP/6-31G(d,p)) = -1423.784445 Ha

K-S1b (conformation 8)

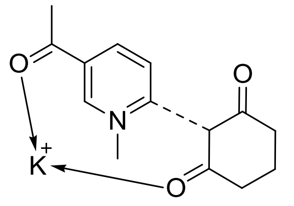

Center Number	Atomic Number	Atomic Type	X	Y	Z
1	6	0	-2.052962	-0.984265	0.322551
2	6	0	-1.393002	-2.193216	-0.076030
3	6	0	-0.079971	-2.378822	0.237986
4	6	0	-1.404873	-0.175459	1.237421
5	1	0	-1.935027	-2.950004	-0.632015
6	1	0	0.466672	-3.259241	-0.071861
7	1	0	-1.910647	0.656805	1.711402
8	7	0	-0.142614	-0.428009	1.636109
9	6	0	0.472262	0.309905	2.745354
10	1	0	0.672584	-0.374531	3.572776
11	1	0	1.410063	0.760050	2.416447
12	1	0	-0.205624	1.094404	3.077843
13	6	0	0.640015	-1.328482	0.916612
14	1	0	1.573704	-1.575150	1.405498
15	6	0	1.429450	-0.195044	-0.692672
16	1	0	0.518806	-0.346485	-1.264590
17	6	0	3.014541	1.647928	0.105514
18	6	0	3.968731	-0.542360	-0.724134
19	6	0	3.982602	0.496409	0.403659
20	1	0	2.949963	2.360040	0.934223
21	1	0	3.374901	2.219208	-0.762920
22	1	0	4.568826	-1.424118	-0.478593
23	1	0	4.415294	-0.108788	-1.630958
24	1	0	4.995383	0.887673	0.539836
25	1	0	3.708689	0.017319	1.352281
26	6	0	2.570432	-1.011648	-1.115762
27	6	0	1.610774	1.174790	-0.244526
28	8	0	0.643455	1.952468	-0.133237
29	8	0	2.417085	-2.056633	-1.755651
30	6	0	-3.387937	-0.552740	-0.143510
31	6	0	-4.312852	-1.570732	-0.765488
32	1	0	-3.890258	-1.936705	-1.708441
33	1	0	-4.444425	-2.438041	-0.110901
34	1	0	-5.278506	-1.106461	-0.966366
35	8	0	-3.719846	0.633586	-0.041248
36	19	0	-1.845311	2.637217	-0.875642

E(RB3LYP/6-31G(d,p)) = -1424.00978585 Ha

ΔG (298.15 K, 1 atm, B3LYP/6-31G(d,p)) = -1423.783236 Ha

9 (intermediate)

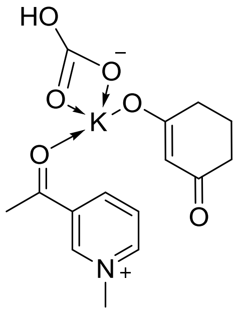

Center Number	Atomic Number	Atomic Type	Coordinates (Angstroms) X	Y	Z
1	6	0	1.333004	-2.401810	-1.467469
2	6	0	1.213115	-2.225085	0.875052
3	6	0	-0.080871	-1.724440	0.806016
4	6	0	0.031215	-1.950316	-1.591620
5	1	0	1.954999	-2.659507	-2.312521
6	1	0	1.744306	-2.364550	1.806664
7	1	0	-0.404128	-1.859242	-2.578852
8	7	0	1.894769	-2.543809	-0.244669
9	6	0	3.313233	-2.966349	-0.169656
10	1	0	3.434155	-3.896319	-0.724849
11	1	0	3.902867	-2.162467	-0.615783
12	1	0	3.583215	-3.121777	0.872612
13	6	0	-0.816159	-1.276070	2.041344
14	8	0	-1.772305	-0.522494	1.917747
15	6	0	-0.349769	-1.755562	3.391705
16	1	0	-0.254711	-2.845432	3.413299
17	1	0	0.634382	-1.333382	3.625547
18	1	0	-1.060357	-1.428792	4.150621
19	6	0	-0.682830	-1.586111	-0.453890
20	1	0	-1.688450	-1.177527	-0.550395
21	6	0	3.740583	2.773215	0.312986
22	6	0	4.032161	1.316891	-0.058750
23	6	0	1.703736	1.304020	-1.036399
24	6	0	2.298123	2.928228	0.802951
25	1	0	4.041223	0.694491	0.849220
26	1	0	5.019920	1.204069	-0.518501
27	1	0	3.888124	3.405820	-0.572061
28	1	0	4.446033	3.126145	1.074185
29	1	0	0.970025	0.880746	-1.718529
30	1	0	2.179107	2.433609	1.779350
31	1	0	2.032378	3.979809	0.956483
32	6	0	1.272810	2.319730	-0.156092
33	6	0	2.996672	0.727149	-1.020866
34	8	0	3.334613	-0.262593	-1.727632
35	8	0	0.080032	2.738100	-0.090103
36	19	0	-2.310589	1.870062	0.482183
37	6	0	-4.318233	0.109553	-0.957126
38	8	0	-3.144567	0.078745	-1.409073
39	8	0	-5.179123	-0.834147	-1.512193
40	1	0	-6.030360	-0.687860	-1.073345
41	8	0	-4.803108	0.868564	-0.081380

E(RB3LYP/6-31G(d,p)) = -1688.59355784 Ha

ΔG (298.15 K, 1 atm, B3LYP/6-31G(d,p)) = -1688.352488 Ha

10 (TS)

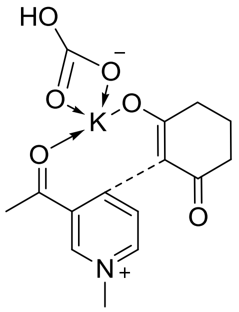

Center Number	Atomic Number	Atomic Type	Coordinates (Angstroms) X	Y	Z
1	6	0	1.914077	-2.396226	-1.087346
2	6	0	1.822947	-1.688729	1.162946
3	6	0	0.726264	-0.889554	0.908060
4	6	0	0.844850	-1.625509	-1.409090
5	1	0	2.388786	-3.070932	-1.788126
6	1	0	2.225117	-1.820399	2.159157
7	1	0	0.444291	-1.683611	-2.413752
8	7	0	2.438962	-2.399411	0.190703
9	6	0	3.640212	-3.197780	0.471426
10	1	0	3.513513	-4.203084	0.065977
11	1	0	4.515394	-2.728820	0.015358
12	1	0	3.785349	-3.261797	1.548776
13	6	0	-0.033422	-0.277070	2.015149
14	8	0	-1.018446	0.425202	1.769650
15	6	0	0.380402	-0.524006	3.452467
16	1	0	0.311161	-1.587239	3.704062
17	1	0	1.413268	-0.207442	3.629714
18	1	0	-0.282773	0.040800	4.107880
19	6	0	0.281157	-0.699410	-0.461675
20	1	0	-0.752647	-0.405155	-0.608745
21	6	0	2.514435	3.095615	0.161640
22	6	0	3.113188	1.739558	-0.232509
23	6	0	0.874836	1.183541	-1.317498
24	6	0	1.031204	2.948143	0.524474
25	1	0	3.093673	1.069051	0.640207
26	1	0	4.155913	1.825146	-0.550170
27	1	0	2.619033	3.798752	-0.674112
28	1	0	3.066004	3.522739	1.005208
29	1	0	0.372739	0.933810	-2.249328
30	1	0	0.937461	2.351388	1.442582
31	1	0	0.558368	3.913996	0.723991
32	6	0	0.225578	2.246262	-0.560973
33	6	0	2.327700	1.069429	-1.354670
34	8	0	2.904205	0.400819	-2.222345
35	8	0	-0.954617	2.575861	-0.774053
36	19	0	-3.105468	1.489833	0.374993
37	6	0	-3.703840	-1.261505	-0.720205
38	8	0	-2.934368	-0.559894	-1.421611
39	8	0	-3.884872	-2.566241	-1.178315
40	1	0	-4.496959	-2.971500	-0.546201
41	8	0	-4.323660	-0.946150	0.328493

E(RB3LYP/6-31G(d,p)) = -1688.58024711 Ha

ΔG (298.15 K, 1 atm, B3LYP/6-31G(d,p)) = -1688.335828 Ha

11 (intermediate)

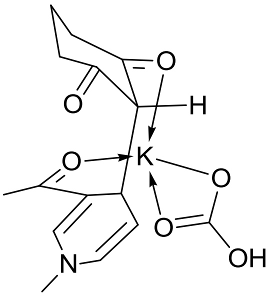

Center Number	Atomic Number	Atomic Type	X	Y	Z
1	6	0	-1.693912	2.527166	-1.122893
2	6	0	-1.658113	1.767992	1.118395
3	6	0	-0.733563	0.791167	0.827992
4	6	0	-0.818589	1.588712	-1.505831
5	1	0	-2.028890	3.325970	-1.773628
6	1	0	-1.966524	1.972304	2.137079
7	1	0	-0.419144	1.627490	-2.513943
8	7	0	-2.205435	2.575109	0.182129
9	6	0	-3.239172	3.555835	0.510377
10	1	0	-2.934818	4.550029	0.172301
11	1	0	-4.186471	3.291401	0.031159
12	1	0	-3.384372	3.578848	1.590303
13	6	0	0.008706	0.146385	1.896360
14	8	0	0.889331	-0.693547	1.629993
15	6	0	-0.253430	0.494292	3.353805
16	1	0	0.048497	1.523483	3.573036
17	1	0	-1.311955	0.395913	3.613393
18	1	0	0.328623	-0.183199	3.979596
19	6	0	-0.401250	0.450265	-0.614658
20	1	0	0.677747	0.289408	-0.716857
21	6	0	-2.587633	-3.063412	0.205042
22	6	0	-3.186603	-1.655401	0.047818
23	6	0	-1.028857	-0.925667	-1.195300
24	6	0	-1.068863	-2.996678	0.418828
25	1	0	-2.985306	-1.074240	0.959159
26	1	0	-4.267708	-1.682311	-0.106974
27	1	0	-2.814648	-3.661759	-0.685157
28	1	0	-3.054143	-3.570335	1.054281
29	1	0	-0.757573	-0.896754	-2.256052
30	1	0	-0.855913	-2.529379	1.388954
31	1	0	-0.610276	-3.989094	0.433749
32	6	0	-0.345869	-2.163551	-0.621641
33	6	0	-2.549182	-0.931690	-1.119668
34	8	0	-3.208077	-0.370558	-1.982308
35	8	0	0.768539	-2.473725	-1.028641
36	19	0	3.028705	-1.606180	0.280683
37	6	0	3.775357	1.169935	-0.620692
38	8	0	3.085268	0.505557	-1.430936
39	8	0	4.003406	2.494634	-0.994688
40	1	0	4.543519	2.866859	-0.281854
41	8	0	4.281265	0.803256	0.472326

SCF Done: E(RB3LYP/6-31G(d,p)) = -1688.59196355 Ha

ΔG (298.15 K, 1 atm, B3LYP/6-31G(d,p)) = -1688.347755 Ha

12 (TS)

Center Number	Atomic Number	Atomic Type	Coordinates (Angstroms) X	Y	Z
1	6	0	-1.451759	2.682437	-1.318016
2	6	0	-1.149898	2.094147	0.953372
3	6	0	-0.555326	0.896663	0.629155
4	6	0	-0.901137	1.536983	-1.737214
5	1	0	-1.759687	3.471259	-1.993693
6	1	0	-1.236082	2.416142	1.984306
7	1	0	-0.757841	1.393472	-2.803674
8	7	0	-1.637532	2.964406	0.043658
9	6	0	-2.294773	4.211497	0.431243
10	1	0	-1.782573	5.065007	-0.021820
11	1	0	-3.339866	4.211401	0.107948
12	1	0	-2.261234	4.314956	1.515598
13	6	0	0.052188	0.084623	1.664448
14	8	0	0.546323	-1.024405	1.377058
15	6	0	0.116287	0.560873	3.108168
16	1	0	0.755503	1.444814	3.200612
17	1	0	-0.871012	0.822569	3.501043
18	1	0	0.534268	-0.242183	3.716324
19	6	0	-0.493573	0.416689	-0.815802
20	1	0	0.532504	0.109829	-1.054103
21	6	0	-2.500568	-2.936537	0.816671
22	6	0	-2.982376	-1.467441	0.735268
23	6	0	-1.351144	-0.894805	-1.167706
24	6	0	-1.316306	-3.301251	-0.111895
25	1	0	-2.415168	-0.858508	1.449931
26	1	0	-4.038671	-1.382811	0.999977
27	1	0	-3.343003	-3.590652	0.577348
28	1	0	-2.228052	-3.151985	1.852742
29	1	0	-1.434346	-0.875950	-2.261177
30	1	0	-0.556562	-3.888018	0.410432
31	1	0	-1.680569	-3.947834	-0.923426
32	6	0	-0.601495	-2.185428	-0.850908
33	6	0	-2.764013	-0.828805	-0.615945
34	8	0	-3.647869	-0.244730	-1.225970
35	8	0	0.510013	-2.373669	-1.331082
36	19	0	2.785937	-1.883587	0.087102
37	6	0	3.651118	0.954258	-0.428710
38	8	0	3.111984	0.370640	-1.399076
39	8	0	3.921934	2.307538	-0.635676
40	1	0	4.333681	2.609139	0.187652
41	8	0	3.974526	0.486281	0.694829

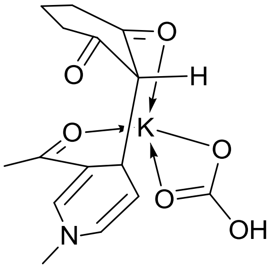

E(RB3LYP/6-31G(d,p)) = -1688.58424107 Ha

ΔG (298.15 K, 1 atm, B3LYP/6-31G(d,p)) = -1688.339803 Ha

13 (intermediate)

Center Number	Atomic Number	Atomic Type	Coordinates (Angstroms) X	Y	Z
1	6	0	-1.704804	2.545934	-1.184190
2	6	0	-1.583764	1.807452	1.060077
3	6	0	-0.737128	0.773934	0.732565
4	6	0	-0.917473	1.549036	-1.605932
5	1	0	-2.042380	3.346992	-1.830816
6	1	0	-1.833691	2.026756	2.091604
7	1	0	-0.599976	1.542139	-2.643926
8	7	0	-2.122024	2.649306	0.151237
9	6	0	-3.055221	3.709382	0.528328
10	1	0	-2.680292	4.679553	0.190362
11	1	0	-4.038602	3.533543	0.082192
12	1	0	-3.158971	3.729591	1.613071
13	6	0	-0.022323	0.056981	1.768491
14	8	0	0.761282	-0.864294	1.463009
15	6	0	-0.187328	0.418606	3.236964
16	1	0	0.236928	1.405952	3.446363
17	1	0	-1.238112	0.437428	3.541507
18	1	0	0.340548	-0.324243	3.836212
19	6	0	-0.473586	0.414403	-0.721504
20	1	0	0.599978	0.247285	-0.869697
21	6	0	-2.128183	-2.678484	0.941636
22	6	0	-3.179364	-2.043313	0.027949
23	6	0	-1.131010	-0.966535	-1.234536
24	6	0	-0.940235	-3.206061	0.131978
25	1	0	-3.981719	-1.558550	0.593021
26	1	0	-3.663184	-2.822111	-0.579677
27	1	0	-2.579887	-3.497649	1.508712
28	1	0	-1.778842	-1.947509	1.676129
29	1	0	-1.012482	-0.913608	-2.323172
30	1	0	-0.149013	-3.609442	0.769692
31	1	0	-1.271324	-4.034074	-0.513219
32	6	0	-0.319362	-2.183005	-0.801538
33	6	0	-2.633096	-1.031706	-0.969046
34	8	0	-3.390544	-0.299298	-1.586837
35	8	0	0.795918	-2.359768	-1.279871
36	19	0	3.003397	-1.607635	0.158861
37	6	0	3.690964	1.252746	-0.481338
38	8	0	3.087824	0.629878	-1.387570
39	8	0	3.883893	2.611651	-0.732696
40	1	0	4.357900	2.944806	0.043688
41	8	0	4.138403	0.818936	0.612904

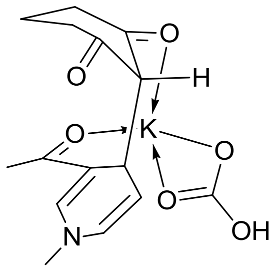

E(RB3LYP/6-31G(d,p)) = -1688.58926368 Ha

ΔG (298.15 K, 1 atm, B3LYP/6-31G(d,p)) = -1688.345392 Ha

14 (TS)

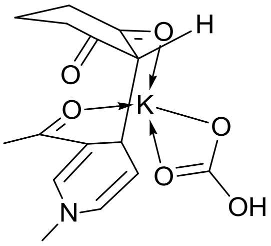

Center Number	Atomic Number	Atomic Type	X	Y	Z
1	6	0	-1.502931	2.652852	-1.268779
2	6	0	-1.372030	1.976143	0.995098
3	6	0	-0.672411	0.835944	0.674273
4	6	0	-0.865776	1.552928	-1.685495
5	1	0	-1.782562	3.461951	-1.932810
6	1	0	-1.560009	2.249128	2.027001
7	1	0	-0.613897	1.467840	-2.738254
8	7	0	-1.832152	2.855795	0.080626
9	6	0	-2.607950	4.036055	0.456137
10	1	0	-2.131877	4.939943	0.065759
11	1	0	-3.625593	3.971129	0.059406
12	1	0	-2.657192	4.105067	1.542752
13	6	0	-0.044431	0.049050	1.712734
14	8	0	0.606506	-0.974187	1.413609
15	6	0	-0.143597	0.448117	3.177498
16	1	0	0.375632	1.393629	3.364320
17	1	0	-1.182772	0.570823	3.498000
18	1	0	0.321061	-0.333319	3.779944
19	6	0	-0.489209	0.411973	-0.776244
20	1	0	0.562345	0.157231	-0.954578
21	6	0	-2.183177	-2.795432	0.912646
22	6	0	-3.260337	-2.044015	0.126755
23	6	0	-1.269711	-0.924771	-1.194351
24	6	0	-1.067951	-3.275748	-0.018837
25	1	0	-4.039308	-1.629287	0.773437
26	1	0	-3.767342	-2.740584	-0.558141
27	1	0	-2.631376	-3.652324	1.424111
28	1	0	-1.760008	-2.150551	1.688074
29	1	0	-1.294371	-0.887610	-2.291469
30	1	0	-0.258497	-3.774386	0.521368
31	1	0	-1.472698	-4.015900	-0.726105
32	6	0	-0.459171	-2.180819	-0.873827
33	6	0	-2.735166	-0.917521	-0.749192
34	8	0	-3.482648	-0.034143	-1.137730
35	8	0	0.644259	-2.325389	-1.388960
36	19	0	2.863130	-1.751174	0.123534
37	6	0	3.701366	1.090118	-0.407933
38	8	0	3.146327	0.503015	-1.367062
39	8	0	3.958055	2.445423	-0.619731
40	1	0	4.384355	2.749664	0.195179
41	8	0	4.051826	0.624036	0.708321

E(RB3LYP/6-31G(d,p)) = -1688.58913729 Ha

ΔG (298.15 K, 1 atm, B3LYP/6-31G(d,p)) = -1688.344590 Ha

15 (intermediate)

Center Number	Atomic Number	Atomic Type	Coordinates (Angstroms) X	Y	Z
1	6	0	-1.021059	2.882961	-1.417804
2	6	0	-0.724980	2.314445	0.862644
3	6	0	-0.417064	1.008850	0.563669
4	6	0	-0.762708	1.632604	-1.813921
5	1	0	-1.207344	3.695793	-2.109527
6	1	0	-0.700359	2.675762	1.884073
7	1	0	-0.735488	1.423655	-2.879477
8	7	0	-1.046134	3.244754	-0.060786
9	6	0	-1.398258	4.615046	0.303734
10	1	0	-0.744226	5.324365	-0.211725
11	1	0	-2.436823	4.831283	0.034991
12	1	0	-1.279355	4.744605	1.379374
13	6	0	-0.004186	0.104569	1.609828
14	8	0	0.254605	-1.090682	1.342204
15	6	0	0.136033	0.569565	3.052365
16	1	0	0.936676	1.309558	3.152157
17	1	0	-0.786070	1.023727	3.427634
18	1	0	0.378666	-0.296080	3.669961
19	6	0	-0.503554	0.486676	-0.866777
20	1	0	0.453494	0.026187	-1.141605
21	6	0	-2.681316	-2.706759	0.810419
22	6	0	-3.627032	-1.680432	0.173751
23	6	0	-1.552958	-0.682226	-1.073449
24	6	0	-1.645440	-3.181472	-0.208497
25	1	0	-4.398614	-1.333486	0.865747
26	1	0	-4.140413	-2.142515	-0.682999
27	1	0	-3.256652	-3.560647	1.179615
28	1	0	-2.172667	-2.262280	1.671912
29	1	0	-1.827035	-0.650884	-2.140727
30	1	0	-0.897781	-3.845571	0.234725
31	1	0	-2.145814	-3.762190	-0.998299
32	6	0	-0.907420	-2.067610	-0.927995
33	6	0	-2.885210	-0.463231	-0.344841
34	8	0	-3.347266	0.659174	-0.225142
35	8	0	0.156542	-2.295149	-1.491988
36	19	0	2.402897	-2.225119	0.097900
37	6	0	3.752165	0.430584	-0.335691
38	8	0	3.191138	-0.060095	-1.343734
39	8	0	4.254616	1.720544	-0.513879
40	1	0	4.653967	1.953241	0.337447
41	8	0	3.919322	-0.080289	0.803479

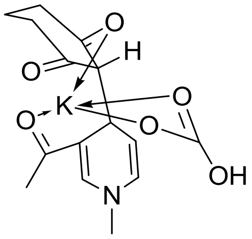

E(RB3LYP/6-31G(d,p)) = -1688.58989136 Ha

ΔG (298.15 K, 1 atm, B3LYP/6-31G(d,p)) = -1688.345857 Ha

16 (TS)

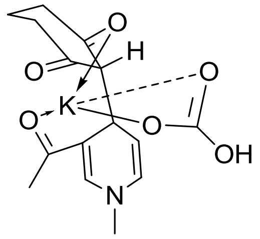

Center Number	Atomic Number	Atomic Type	X	Y	Z
1	6	0	1.520054	-2.149035	-1.485063
2	6	0	2.221657	-1.650021	0.721834
3	6	0	1.119718	-0.862757	0.957527
4	6	0	0.442797	-1.363480	-1.372692
5	1	0	1.692380	-2.804826	-2.331160
6	1	0	2.911737	-1.914824	1.515269
7	1	0	-0.321947	-1.374156	-2.142168
8	7	0	2.484816	-2.233440	-0.470288
9	6	0	3.728922	-2.953328	-0.718468
10	1	0	3.519756	-3.895266	-1.232530
11	1	0	4.410924	-2.358616	-1.335675
12	1	0	4.218043	-3.174177	0.231357
13	6	0	0.710397	-0.637167	2.333866
14	8	0	-0.430553	-0.233893	2.623083
15	6	0	1.667074	-0.939836	3.485137
16	1	0	1.681165	-2.012961	3.705411
17	1	0	2.693431	-0.624273	3.280629
18	1	0	1.302245	-0.418328	4.371538
19	6	0	0.242103	-0.413673	-0.210773
20	1	0	-0.807563	-0.466033	0.085962
21	6	0	1.398870	3.903988	-0.446807
22	6	0	2.065438	2.947722	-1.444369
23	6	0	0.427074	1.060422	-0.716450
24	6	0	-0.095712	3.593761	-0.319584
25	1	0	3.139702	3.126254	-1.530347
26	1	0	1.619691	3.089993	-2.439082
27	1	0	1.535063	4.938632	-0.773332
28	1	0	1.883508	3.813626	0.533020
29	1	0	-0.074927	1.082410	-1.703222
30	1	0	-0.586860	4.216424	0.432140
31	1	0	-0.591111	3.793212	-1.281579
32	6	0	-0.385703	2.141649	0.021464
33	6	0	1.865515	1.497349	-1.037898
34	8	0	2.801982	0.720361	-1.002045
35	8	0	-1.298787	1.859413	0.778401
36	19	0	-2.967154	0.104750	1.907248
37	6	0	-3.141864	-1.014402	-1.565172
38	8	0	-2.523683	-0.134388	-2.192247
39	8	0	-3.658044	-2.051583	-2.356861
40	1	0	-4.107142	-2.640502	-1.732946
41	8	0	-3.372656	-1.122398	-0.327118

E(RB3LYP/6-31G(d,p)) = -1688.58058399 Ha

ΔG (298.15 K, 1 atm, B3LYP/6-31G(d,p)) = -1688.332587 Ha

17 (intermediate)

Center Number	Atomic Number	Atomic Type	Coordinates (Angstroms) X	Y	Z
1	6	0	-1.942086	-0.949517	2.209013
2	6	0	-2.635240	-1.348324	-0.020987
3	6	0	-1.385793	-1.065703	-0.519396
4	6	0	-0.709942	-0.605126	1.815677
5	1	0	-2.218934	-1.084579	3.248646
6	1	0	-3.416112	-1.770487	-0.643958
7	1	0	0.070717	-0.455471	2.554158
8	7	0	-2.961243	-1.231432	1.287854
9	6	0	-4.330700	-1.405288	1.758237
10	1	0	-4.794674	-0.439201	1.984458
11	1	0	-4.919599	-1.904954	0.987566
12	1	0	-4.341345	-2.021192	2.661386
13	6	0	-1.003772	-1.656946	-1.792565
14	8	0	0.186566	-1.787061	-2.127485
15	6	0	-2.069493	-2.195844	-2.744613
16	1	0	-2.377130	-3.205033	-2.449179
17	1	0	-2.963224	-1.568164	-2.785772
18	1	0	-1.629130	-2.257827	-3.741316
19	6	0	-0.360719	-0.355655	0.363143
20	1	0	0.628323	-0.780297	0.175431
21	6	0	-0.406853	3.883352	-1.168020
22	6	0	0.920107	3.116114	-1.210046
23	6	0	-1.182924	3.565710	0.118724
24	1	0	1.451136	3.263339	-2.153550
25	1	0	1.575583	3.476052	-0.403385
26	1	0	-0.214465	4.957912	-1.231799
27	1	0	-1.016156	3.617120	-2.040237
28	1	0	-2.156326	4.061007	0.140112
29	1	0	-0.605568	3.911301	0.987485
30	6	0	-1.411481	2.069470	0.262008
31	6	0	0.753808	1.620403	-0.995828
32	8	0	1.397244	0.824895	-1.659664
33	8	0	-2.524343	1.617016	0.461718
34	6	0	-0.162539	1.185232	0.158043
35	1	0	0.470290	1.487441	1.015612
36	19	0	2.770384	-1.469933	-1.637902
37	6	0	3.066761	-0.357283	1.866894
38	8	0	2.441348	0.715410	1.970990
39	8	0	3.781251	-0.733616	3.013065
40	1	0	4.208883	-1.569532	2.775882
41	8	0	3.155975	-1.158174	0.894115

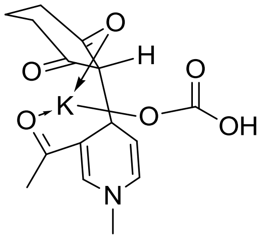

E(RB3LYP/6-31G(d,p)) = -1688.58078744 Ha

ΔG (298.15 K, 1 atm, B3LYP/6-31G(d,p)) = -1688.335562 Ha

18 (TS)

Center Number	Atomic Number	Atomic Type	Coordinates (Angstroms) X	Y	Z
1	6	0	-2.094613	-0.655241	-2.184949
2	6	0	-2.990692	0.409803	-0.265722
3	6	0	-1.750796	0.861664	0.117177
4	6	0	-0.842482	-0.330427	-1.841941
5	1	0	-2.357819	-1.093743	-3.141385
6	1	0	-3.902907	0.752428	0.212091
7	1	0	-0.043299	-0.492169	-2.557745
8	7	0	-3.183176	-0.394073	-1.340481
9	6	0	-4.480884	-0.989585	-1.630533
10	1	0	-4.511135	-2.040365	-1.321833
11	1	0	-5.260705	-0.444646	-1.095533
12	1	0	-4.687315	-0.932585	-2.702728
13	6	0	-1.657193	2.084042	0.899611
14	8	0	-0.614670	2.763336	0.933983
15	6	0	-2.872546	2.610879	1.658939
16	1	0	-3.504181	3.216884	0.999738
17	1	0	-3.490796	1.816144	2.083657
18	1	0	-2.514752	3.255380	2.464200
19	6	0	-0.494983	0.226704	-0.475887
20	1	0	0.243093	1.013954	-0.622713
21	6	0	0.859595	-2.510105	2.795516
22	6	0	1.783977	-1.356964	2.410347
23	6	0	0.273892	-3.143384	1.531439
24	1	0	2.137621	-0.800389	3.282711
25	1	0	2.675438	-1.755878	1.903609
26	1	0	1.403600	-3.254790	3.385775
27	1	0	0.044789	-2.136522	3.429267
28	1	0	-0.431461	-3.946686	1.759544
29	1	0	1.087292	-3.572704	0.928764
30	6	0	-0.461121	-2.106153	0.683293
31	6	0	1.161182	-0.344211	1.447392
32	8	0	1.563460	0.825783	1.515607
33	8	0	-1.578512	-2.381326	0.241208
34	6	0	0.237918	-0.829507	0.405475
35	1	0	1.287697	-1.170206	-0.447527
36	19	0	1.946101	2.909419	0.015499
37	6	0	2.875725	-0.421886	-1.681222
38	8	0	2.241198	-1.442248	-1.219292
39	8	0	3.948573	-0.796165	-2.440321
40	1	0	4.356671	0.027304	-2.749751
41	8	0	2.626527	0.781167	-1.523044

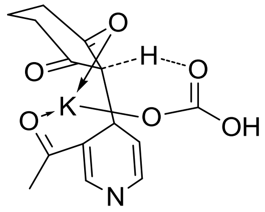

E(RB3LYP/6-31G(d,p)) = -1688.56797662 Ha

ΔG (298.15 K, 1 atm, B3LYP/6-31G(d,p)) = -1688.323139 Ha

19 (intermediate)

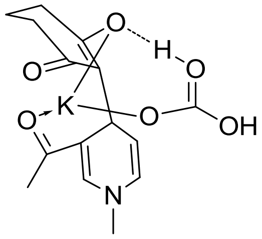

Center Number	Atomic Number	Atomic Type	Coordinates (Angstroms) X	Y	Z
1	6	0	2.109262	-1.740011	-1.630514
2	6	0	2.552886	-1.422477	0.681075
3	6	0	1.346066	-0.801946	0.886651
4	6	0	0.929800	-1.115376	-1.541329
5	1	0	2.455831	-2.235188	-2.530755
6	1	0	3.223183	-1.651584	1.502274
7	1	0	0.272016	-1.105084	-2.404715
8	7	0	2.978190	-1.848789	-0.530618
9	6	0	4.315377	-2.394490	-0.730163
10	1	0	4.960313	-1.679211	-1.252530
11	1	0	4.762036	-2.626455	0.237602
12	1	0	4.262388	-3.314108	-1.319964
13	6	0	0.857092	-0.582626	2.228192
14	8	0	-0.285526	-0.121643	2.434985
15	6	0	1.710010	-0.941695	3.441987
16	1	0	1.783500	-2.027855	3.563262
17	1	0	2.727422	-0.546622	3.368616
18	1	0	1.232254	-0.526898	4.330936
19	6	0	0.477178	-0.377928	-0.298218
20	1	0	-0.550396	-0.679379	-0.076419
21	6	0	0.390094	4.022015	-0.243127
22	6	0	-0.893133	3.309180	-0.661946
23	6	0	1.580708	3.383173	-0.953589
24	1	0	-1.751484	3.629426	-0.060879
25	1	0	-1.141878	3.561251	-1.704231
26	1	0	0.327373	5.092876	-0.465959
27	1	0	0.521065	3.928713	0.842926
28	1	0	2.535886	3.812394	-0.635859
29	1	0	1.501191	3.557107	-2.037471
30	6	0	1.657858	1.871006	-0.725989
31	6	0	-0.805459	1.794773	-0.559983
32	8	0	-1.965425	1.199976	-0.517185
33	8	0	2.775428	1.328678	-0.764895
34	6	0	0.423020	1.139046	-0.517453
35	1	0	-2.230700	0.066816	-1.228865
36	19	0	-2.816348	0.269619	1.875697
37	6	0	-3.082144	-1.709555	-1.136038
38	8	0	-2.524572	-0.775115	-1.849261
39	8	0	-3.441253	-2.760926	-1.905599
40	1	0	-3.846857	-3.411773	-1.311759
41	8	0	-3.282596	-1.698997	0.079771

E(RB3LYP/6-31G(d,p)) = -1688.60183195 Ha

ΔG (298.15 K, 1 atm, B3LYP/6-31G(d,p)) = -1688.355688 Ha

20 (TS)

Center Number	Atomic Number	Atomic Type	Coordinates (Angstroms) X	Y	Z
1	6	0	-1.175297	2.414428	-1.532992
2	6	0	-1.399814	2.336793	0.830568
3	6	0	-0.711520	1.153203	0.916250
4	6	0	-0.525868	1.243761	-1.557101
5	1	0	-1.332577	3.027783	-2.413200
6	1	0	-1.720049	2.876453	1.714927
7	1	0	-0.147663	0.880967	-2.509566
8	7	0	-1.677148	2.959558	-0.340633
9	6	0	-2.510387	4.154723	-0.398200
10	1	0	-3.504918	3.925518	-0.796875
11	1	0	-2.622779	4.568195	0.604982
12	1	0	-2.042587	4.909779	-1.036531
13	6	0	-0.240199	0.680081	2.198365
14	8	0	0.503247	-0.319454	2.290060
15	6	0	-0.600326	1.416994	3.486219
16	1	0	-0.047128	2.359157	3.566074
17	1	0	-1.666598	1.651445	3.551247
18	1	0	-0.324536	0.783860	4.331063
19	6	0	-0.387272	0.348036	-0.341133
20	1	0	0.652251	0.005499	-0.258378
21	6	0	-2.626114	-3.439444	-0.295507
22	6	0	-1.226347	-3.458847	-0.906655
23	6	0	-3.384220	-2.208549	-0.787502
24	1	0	-0.609958	-4.263244	-0.490043
25	1	0	-1.295966	-3.651582	-1.988346
26	1	0	-3.170499	-4.359298	-0.540590
27	1	0	-2.543091	-3.402268	0.799049
28	1	0	-4.360320	-2.104215	-0.301903
29	1	0	-3.582418	-2.304759	-1.866324
30	6	0	-2.618544	-0.896365	-0.567341
31	6	0	-0.464184	-2.146884	-0.709861
32	8	0	0.804205	-2.213033	-0.759493
33	8	0	-3.290981	0.154773	-0.474910
34	6	0	-1.186708	-0.943624	-0.512361
35	1	0	2.103696	0.809821	-1.435954
36	19	0	2.346859	-1.918600	1.311380
37	6	0	3.914156	0.750244	-0.937957
38	8	0	2.943037	1.202182	-1.730308
39	8	0	5.074987	1.282374	-1.322328
40	1	0	5.763524	0.936931	-0.731815
41	8	0	3.772654	-0.024448	-0.007816

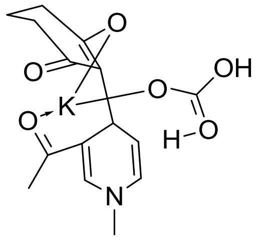

E(RB3LYP/6-31G(d,p)) = -1688.57869106 Ha

ΔG (298.15 K, 1 atm, B3LYP/6-31G(d,p)) = -1688.333400 Ha

21 (intermediate)

Center Number	Atomic Number	Atomic Type	Coordinates (Angstroms) X	Y	Z
1	6	0	0.469166	2.423444	-1.358264
2	6	0	0.324207	2.289997	1.006136
3	6	0	0.129802	0.934887	0.975816
4	6	0	0.245495	1.103024	-1.491299
5	1	0	0.705701	3.072905	-2.194355
6	1	0	0.456535	2.831545	1.936286
7	1	0	0.280039	0.668523	-2.487154
8	7	0	0.447965	3.054559	-0.112678
9	6	0	0.518390	4.509847	-0.040373
10	1	0	-0.446703	4.965967	-0.286792
11	1	0	0.800515	4.810107	0.969851
12	1	0	1.272866	4.882694	-0.737975
13	6	0	0.276687	0.153208	2.189494
14	8	0	0.266123	-1.093389	2.164770
15	6	0	0.493390	0.837589	3.536399
16	1	0	1.509393	1.240745	3.610046
17	1	0	-0.204536	1.662729	3.703834
18	1	0	0.360878	0.093809	4.323608
19	6	0	-0.199189	0.224933	-0.337696
20	1	0	0.381563	-0.695935	-0.384352
21	6	0	-4.366947	-1.169700	-0.172126
22	6	0	-3.351558	-2.076019	-0.865449
23	6	0	-4.152748	0.275802	-0.615232
24	1	0	-3.397655	-3.103755	-0.488994
25	1	0	-3.577127	-2.130500	-1.941724
26	1	0	-5.391158	-1.498015	-0.385990
27	1	0	-4.232264	-1.240081	0.915586
28	1	0	-4.798207	0.973709	-0.071379
29	1	0	-4.412595	0.377388	-1.680412
30	6	0	-2.705239	0.754570	-0.437528
31	6	0	-1.907072	-1.594969	-0.711825
32	8	0	-1.002309	-2.476592	-0.832053
33	8	0	-2.518191	1.984088	-0.292760
34	6	0	-1.653387	-0.217785	-0.477862
35	1	0	2.376408	0.781400	-1.338163
36	19	0	1.062056	-3.119823	0.617667
37	6	0	3.641665	-0.591051	-0.950127
38	8	0	3.351099	0.634213	-1.365659
39	8	0	4.967187	-0.767616	-0.994745
40	1	0	5.148476	-1.670335	-0.688244
41	8	0	2.845726	-1.440057	-0.582176

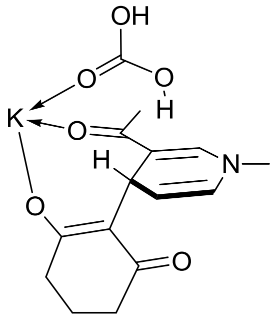

E(RB3LYP/6-31G(d,p)) = -1688.58314411 Ha

ΔG (298.15 K, 1 atm, B3LYP/6-31G(d,p)) = -1688.335901 Ha

22a (rate determining TS, conformation 1)

Center Number	Atomic Number	Atomic Type	Coordinates (Angstroms) X	Y	Z
1	6	0	0.043574	2.478303	-1.234249
2	6	0	0.035852	2.255238	1.114924
3	6	0	0.045664	0.901737	1.058399
4	6	0	0.315561	1.116346	-1.402049
5	1	0	-0.013512	3.160687	-2.076547
6	1	0	0.091754	2.812136	2.041943
7	1	0	0.110215	0.752232	-2.410492
8	7	0	-0.011893	3.055578	-0.023826
9	6	0	-0.240630	4.494316	0.135034
10	1	0	-1.271211	4.680190	0.449062
11	1	0	0.444272	4.896504	0.884605
12	1	0	-0.058343	4.994763	-0.815426
13	6	0	0.243049	0.120155	2.294235
14	8	0	0.467694	-1.094280	2.241796
15	6	0	0.211181	0.806403	3.650489
16	1	0	1.068290	1.478204	3.766677
17	1	0	-0.697429	1.400910	3.781993
18	1	0	0.260584	0.042507	4.427154
19	6	0	-0.116962	0.168150	-0.266750
20	1	0	0.571872	-0.671949	-0.273924
21	6	0	-4.155009	-1.556648	-0.306429
22	6	0	-3.017064	-2.427953	-0.835360
23	6	0	-4.012794	-0.137254	-0.852398
24	1	0	-3.019879	-3.425056	-0.382178
25	1	0	-3.138939	-2.579687	-1.918883
26	1	0	-5.127651	-1.983579	-0.576927
27	1	0	-4.114052	-1.531076	0.790615
28	1	0	-4.756662	0.544715	-0.427371
29	1	0	-4.180103	-0.141613	-1.940453
30	6	0	-2.629563	0.467923	-0.592393
31	6	0	-1.631936	-1.822148	-0.605610
32	8	0	-0.656476	-2.623792	-0.588636
33	8	0	-2.542210	1.718719	-0.546452
34	6	0	-1.505891	-0.408432	-0.455340
35	1	0	1.665348	1.078816	-1.384830
36	19	0	1.616569	-2.969165	0.640366
37	6	0	3.484041	-0.105828	-1.120824
38	8	0	2.951692	1.026184	-1.403564
39	8	0	4.847911	-0.066595	-1.216680
40	1	0	5.157514	-0.956533	-0.987856
41	8	0	2.926631	-1.163854	-0.791141

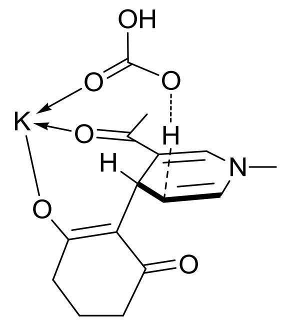

E(RB3LYP/6-31G(d,p)) = -1688.56564714 Ha

ΔG (298.15 K, 1 atm, B3LYP/6-31G(d,p)) = -1688.321654 Ha

E(RB3LYP/6-311++G(d,p)//B3LYP/6-31G(d,p)) = -1688.90648793 Ha

ΔG (298.15 K, 1 atm, B3LYP/6-311++G(d,p)//B3LYP/6-31G(d,p)) =-1688.662495 Ha

22b (rate determining TS, conformation 2)

Center Number	Atomic Number	Atomic Type	Coordinates (Angstroms) X	Y	Z
1	6	0	-0.059504	2.439726	-1.269577
2	6	0	0.057965	2.281387	1.081508
3	6	0	0.086306	0.927200	1.062192
4	6	0	0.216872	1.075499	-1.414328
5	1	0	-0.168855	3.097354	-2.126330
6	1	0	0.153270	2.864998	1.988531
7	1	0	-0.040432	0.681272	-2.399312
8	7	0	-0.059626	3.049054	-0.074769
9	6	0	-0.301001	4.488452	0.056917
10	1	0	-1.319938	4.668293	0.410036
11	1	0	0.409593	4.918289	0.766056
12	1	0	-0.166079	4.966594	-0.912751
13	6	0	0.357920	0.186886	2.309318
14	8	0	0.597548	-1.025376	2.282856
15	6	0	0.383476	0.914403	3.644286
16	1	0	1.228747	1.608934	3.693533
17	1	0	-0.530957	1.492069	3.807299
18	1	0	0.492945	0.176145	4.439352
19	6	0	-0.138377	0.153492	-0.231554
20	1	0	0.560958	-0.677943	-0.253730
21	6	0	-4.049356	-1.572872	-1.213480
22	6	0	-3.033037	-2.492313	-0.537945
23	6	0	-4.063343	-0.214748	-0.514224
24	1	0	-3.380545	-2.747926	0.475079
25	1	0	-2.917811	-3.439694	-1.074641
26	1	0	-3.770670	-1.436859	-2.266951
27	1	0	-5.047210	-2.026712	-1.206511
28	1	0	-4.470752	-0.327539	0.502224
29	1	0	-4.706438	0.507054	-1.028295
30	6	0	-2.670612	0.413043	-0.395558
31	6	0	-1.644186	-1.865763	-0.408497
32	8	0	-0.663653	-2.659632	-0.361774
33	8	0	-2.598816	1.664601	-0.346596
34	6	0	-1.527010	-0.445714	-0.326840
35	1	0	1.561391	1.055377	-1.474441
36	19	0	1.701845	-2.926996	0.691904
37	6	0	3.418268	-0.089455	-1.262714
38	8	0	2.850449	1.018547	-1.568580
39	8	0	4.774422	-0.033235	-1.434237
40	1	0	5.112042	-0.906373	-1.181636
41	8	0	2.899648	-1.139793	-0.853834

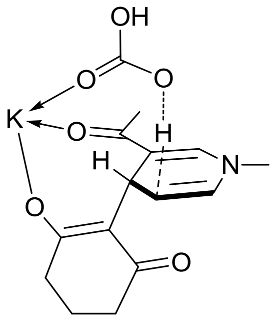

E(RB3LYP/6-31G(d,p)) = -1688.56534980 Ha

ΔG (298.15 K, 1 atm, B3LYP/6-31G(d,p)) = -1688.321112 Ha

E(RB3LYP/6-311++G(d,p)//B3LYP/6-31G(d,p)) = -1688.90619762 Ha

ΔG (298.15 K, 1 atm, B3LYP/6-311++G(d,p)//B3LYP/6-31G(d,p)) = -1688.661960 Ha

22c (rate determining TS, conformation 3)

Center Number	Atomic Number	Atomic Type	Coordinates (Angstroms) X	Y	Z
1	6	0	-0.493923	2.237816	-1.484748
2	6	0	-0.903652	2.466852	0.825343
3	6	0	-0.513877	1.203496	1.101060
4	6	0	0.157735	1.015372	-1.287227
5	1	0	-0.560953	2.694805	-2.467418
6	1	0	-1.196719	3.150795	1.612498
7	1	0	0.251090	0.416784	-2.195053
8	7	0	-0.937015	2.988474	-0.467789
9	6	0	-1.531729	4.310638	-0.676960
10	1	0	-2.606730	4.275593	-0.480869
11	1	0	-1.066953	5.037153	-0.006276
12	1	0	-1.364942	4.622235	-1.707526
13	6	0	-0.466226	0.825383	2.541398
14	8	0	-0.986314	1.529365	3.408906
15	6	0	0.311942	-0.413607	2.935267
16	1	0	0.083096	-1.279298	2.308153
17	1	0	1.383617	-0.211030	2.805191
18	1	0	0.124703	-0.633720	3.987705
19	6	0	-0.181607	0.215579	-0.012105
20	1	0	0.712376	-0.346209	0.264665
21	6	0	-3.116609	-2.804211	-1.269736
22	6	0	-2.053232	-3.242224	-0.264311
23	6	0	-3.672486	-1.438321	-0.870566
24	1	0	-2.527320	-3.468090	0.703218
25	1	0	-1.544891	-4.159459	-0.579846
26	1	0	-2.665970	-2.735730	-2.268799
27	1	0	-3.918258	-3.548957	-1.334527
28	1	0	-4.231720	-1.529932	0.073147
29	1	0	-4.374821	-1.045270	-1.612639
30	6	0	-2.579265	-0.384649	-0.667932
31	6	0	-0.979459	-2.184021	-0.016010
32	8	0	0.143071	-2.601553	0.401099
33	8	0	-2.876288	0.812246	-0.883207
34	6	0	-1.283205	-0.810580	-0.220373
35	1	0	1.440187	1.419780	-1.120861
36	19	0	2.685404	-2.808536	0.109809
37	6	0	3.470736	0.798894	-0.785491
38	8	0	2.654906	1.776429	-0.932706
39	8	0	4.764079	1.221024	-0.662726
40	1	0	5.300519	0.420035	-0.558393
41	8	0	3.214204	-0.414402	-0.746351

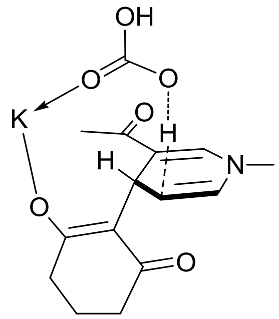

E(RB3LYP/6-31G(d,p)) = -1688.56088119 Ha

ΔG (298.15 K, 1 atm, B3LYP/6-31G(d,p)) = -1688.318450 Ha

E(RB3LYP/6-311++G(d,p)//B3LYP/6-31G(d,p)) = -1688.90271995 Ha

ΔG (298.15 K, 1 atm, B3LYP/6-311++G(d,p)//B3LYP/6-31G(d,p)) = -1688.660288 Ha

22d (rate determining TS, conformation 4)

Center Number	Atomic Number	Atomic Type	Coordinates (Angstroms) X	Y	Z
1	6	0	-0.347703	2.279484	-1.465600
2	6	0	-0.856444	2.467574	0.828383
3	6	0	-0.518560	1.185852	1.089443
4	6	0	0.265102	1.037869	-1.267412
5	1	0	-0.359335	2.760200	-2.439050
6	1	0	-1.166662	3.139927	1.619017
7	1	0	0.390695	0.459175	-2.184060
8	7	0	-0.814595	3.020422	-0.450780
9	6	0	-1.370118	4.359765	-0.657625
10	1	0	-2.456292	4.342065	-0.533310
11	1	0	-0.936553	5.056499	0.063483
12	1	0	-1.129728	4.698224	-1.664985
13	6	0	-0.553580	0.768485	2.518526
14	8	0	-1.077433	1.473136	3.383280
15	6	0	0.145447	-0.518309	2.910228
16	1	0	-0.082808	-1.350106	2.239743
17	1	0	1.230687	-0.359412	2.852675
18	1	0	-0.114838	-0.767482	3.940378
19	6	0	-0.161024	0.216910	-0.031929
20	1	0	0.700103	-0.381393	0.271948
21	6	0	-3.527047	-2.591803	-0.367217
22	6	0	-2.118509	-3.168982	-0.494049
23	6	0	-3.629145	-1.304214	-1.181875
24	1	0	-1.970538	-4.036048	0.158175
25	1	0	-1.952850	-3.521958	-1.523595
26	1	0	-4.276063	-3.321959	-0.694443
27	1	0	-3.735975	-2.373348	0.688264
28	1	0	-4.593653	-0.804252	-1.044966
29	1	0	-3.549412	-1.538117	-2.254587
30	6	0	-2.531951	-0.289270	-0.842721
31	6	0	-1.022693	-2.156128	-0.167788
32	8	0	0.089159	-2.624289	0.221044
33	8	0	-2.768949	0.918790	-1.071269
34	6	0	-1.282954	-0.766400	-0.321041
35	1	0	1.549194	1.407198	-1.020304
36	19	0	2.626150	-2.909437	-0.012321
37	6	0	3.541735	0.724175	-0.621962
38	8	0	2.753813	1.727550	-0.749641
39	8	0	4.832044	1.108212	-0.392376
40	1	0	5.346530	0.290186	-0.312008
41	8	0	3.261050	-0.482538	-0.683160

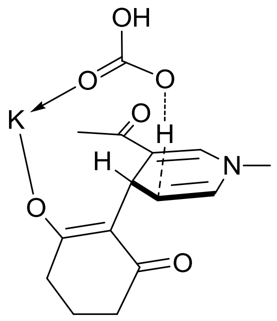

E(RB3LYP/6-31G(d,p)) = -1688.56117542 Ha

ΔG (298.15 K, 1 atm, B3LYP/6-31G(d,p)) = -1688.318877 Ha

E(RB3LYP/6-311++G(d,p)//B3LYP/6-31G(d,p)) = -1688.90303962 Ha

ΔG (298.15 K, 1 atm, B3LYP/6-311++G(d,p)//B3LYP/6-31G(d,p)) = -1688.660742 Ha

Hypothetical reaction leading to 10-acetyl-12-methyl-8-oxa-12-aza-tricyclo[7.3.1.0^{2,7}]trideca-2(7),10-dien-3-one (6aa)

Basing on TSs **22** we have constructed the TSs **24** which are supposed to be rate-determining TSs for formation of product **6aa** (Scheme below)**.** This product, a regioisomer of compound **6a**, could be formed in the case of initial nucleophilic attack of potassium enolate on the 6-C of the pyridinium salt **2a**. Thereafter, TSs **22** and **24** were recomputed at the b3lyp/6-311++g(d,p)//b3lyp/6-31g(d,p) level, and surprisingly **24c** (the lowest among TSs **24**) is 3.5 kcal/mol lower than **22a** (the lowest among TSs **22**).

In the plausible reaction mechanism (see main document) the lowest intermediate is enol **19**. Using the descent from TS **24** by IRC integration and subsequent relaxed scanning we have constructed similar enols **25**, which should be the lowest intermediates leading to product **6aa**. Thus, we can assess the activation energy for synthesizing compound **6a** to be 21.3 kcal/mol (energy gap between **22a** and **19**) and that for compound **6aa** to be 15.6 kcal/mol (energy gap between **24c** and **25c**).

In summary the formation of a solid amorphous phase has a crucial impact on the reaction: it determines the product regioselectivity, and for the case of reaction promoted by DIPEA/KI even causes the reaction running (see thermodynamic analysis).

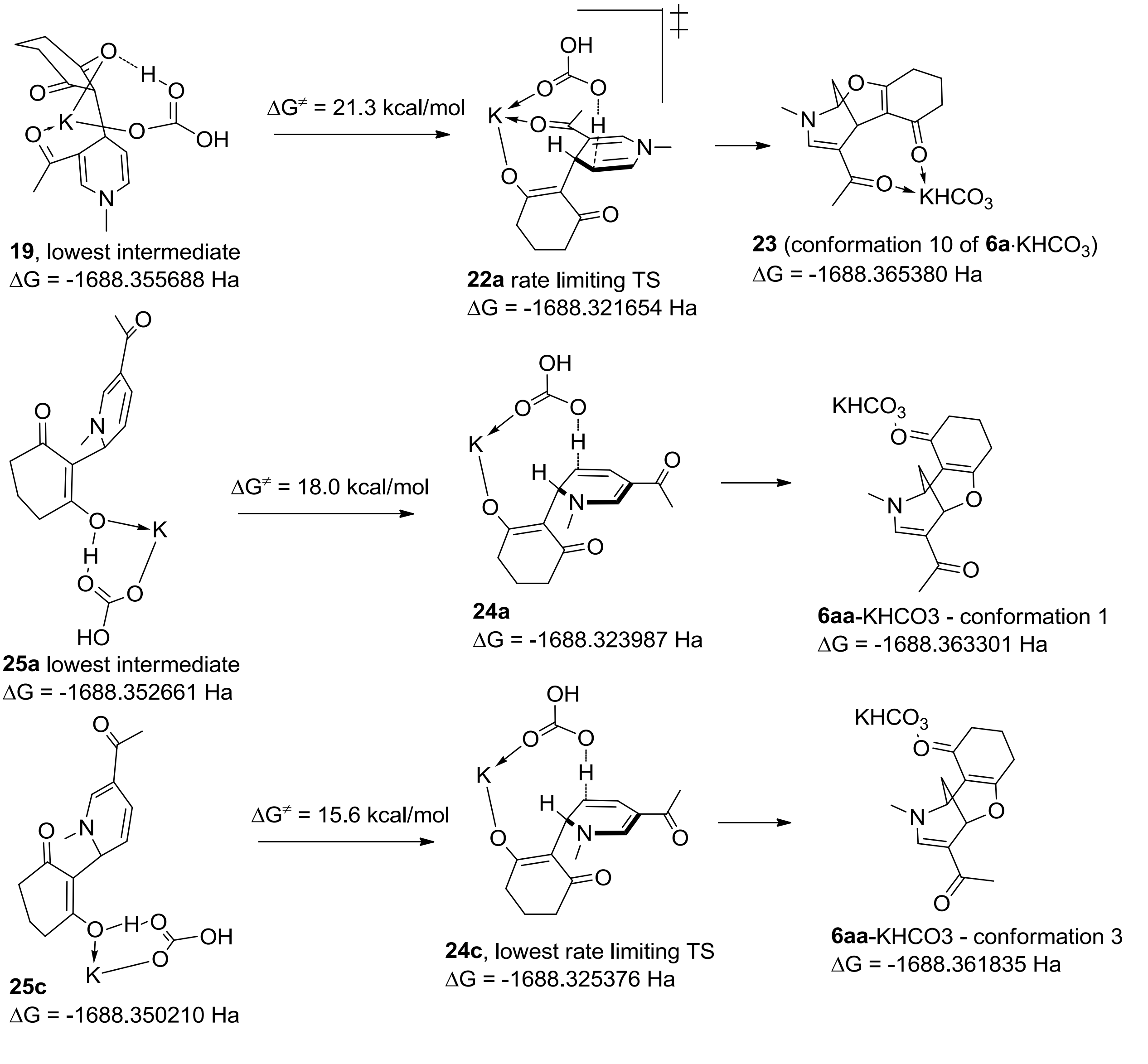

TS 24a (conformation 1)

Center Number	Atomic Number	Atomic Type	Coordinates (Angstroms) X	Y	Z
1	6	0	-0.029468	-0.158816	0.232134
2	6	0	2.259061	0.026285	1.189755
3	6	0	2.855527	0.342935	-0.048155
4	6	0	0.597703	0.524543	-0.993418
5	1	0	-0.865875	0.452749	0.580583
6	1	0	2.866503	-0.074775	2.083870
7	1	0	0.014508	0.376910	-1.903943
8	7	0	0.966074	-0.145052	1.365289
9	6	0	0.418313	-0.439053	2.689203
10	1	0	-0.294211	0.342412	2.970508
11	1	0	-0.102489	-1.398290	2.668250
12	1	0	1.222015	-0.475753	3.424715
13	6	0	4.305860	0.500202	-0.214930
14	8	0	4.792985	0.785327	-1.312369
15	6	0	5.215602	0.302694	0.988469
16	1	0	4.973584	1.009119	1.790051
17	1	0	5.116927	-0.707540	1.400118
18	1	0	6.248372	0.460630	0.676201
19	6	0	1.992414	0.511667	-1.138842
20	1	0	2.442252	0.752826	-2.098861
21	6	0	-1.818793	-3.909518	-1.102479
22	6	0	-0.338904	-3.972154	-0.727336
23	6	0	-2.587964	-3.111022	-0.050429
24	1	0	-0.214376	-4.571657	0.187243
25	1	0	0.263723	-4.458016	-1.501520
26	1	0	-1.926463	-3.420918	-2.079791
27	1	0	-2.238141	-4.916913	-1.204541
28	1	0	-2.611301	-3.670205	0.897274
29	1	0	-3.631637	-2.950470	-0.340661
30	6	0	-1.974492	-1.741497	0.237126
31	6	0	0.273180	-2.592402	-0.466636
32	8	0	1.503571	-2.451872	-0.632244
33	8	0	-2.742547	-0.853773	0.706929
34	6	0	-0.587112	-1.538450	-0.009250
35	1	0	0.408169	1.829168	-0.799952
36	19	0	-4.066852	1.301726	0.274783
37	6	0	-1.046985	3.409185	-0.503164
38	8	0	0.192652	3.093782	-0.545255
39	8	0	-1.235758	4.753303	-0.336743
40	1	0	-2.195424	4.890705	-0.320746
41	8	0	-2.032416	2.658857	-0.592690

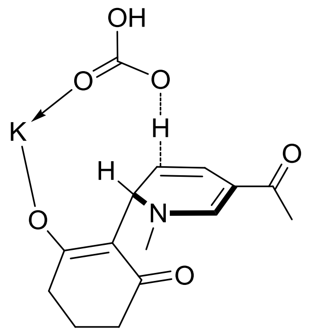

E(RB3LYP/6-31G(d,p)) = -1688.56529016 Ha

ΔG (298.15 K, 1 atm, B3LYP/6-31G(d,p)) = -1688.323987 Ha

E(RB3LYP/6-311++G(d,p)//B3LYP/6-31G(d,p)) = -1688.90821885 Ha

ΔG (298.15 K, 1 atm, B3LYP/6-311++G(d,p)//B3LYP/6-31G(d,p)) = -1688.666916 Ha

TS 24b (conformation 2)

Center Number	Atomic Number	Atomic Type	Coordinates (Angstroms) X	Y	Z
1	6	0	0.040885	0.160867	0.224540
2	6	0	-2.255051	-0.008213	1.165857
3	6	0	-2.834448	-0.381400	-0.063801
4	6	0	-0.563994	-0.592233	-0.970920
5	1	0	0.884901	-0.419011	0.607837
6	1	0	-2.872719	0.122674	2.048976
7	1	0	0.032495	-0.492553	-1.879186
8	7	0	-0.965537	0.185604	1.347002
9	6	0	-0.433567	0.535372	2.663733
10	1	0	0.266893	-0.239033	2.991928
11	1	0	0.096629	1.487665	2.604174
12	1	0	-1.246775	0.614653	3.385300
13	6	0	-4.281278	-0.556635	-0.240987
14	8	0	-4.752840	-0.892235	-1.330988
15	6	0	-5.207629	-0.314517	0.941456
16	1	0	-4.969732	-0.982196	1.776761
17	1	0	-5.123148	0.714014	1.308591
18	1	0	-6.234912	-0.495849	0.623755
19	6	0	-1.956416	-0.591733	-1.135924
20	1	0	-2.392888	-0.879338	-2.089318
21	6	0	1.534863	4.262948	-0.133125
22	6	0	0.309998	3.908371	-0.975056
23	6	0	2.558788	3.130787	-0.203021
24	1	0	-0.483842	4.656498	-0.883876
25	1	0	0.590020	3.876214	-2.039006
26	1	0	1.979086	5.206369	-0.470096
27	1	0	1.228729	4.412041	0.910701
28	1	0	3.400621	3.297303	0.477119
29	1	0	2.983973	3.075599	-1.216858
30	6	0	1.968994	1.759840	0.124107
31	6	0	-0.292268	2.548329	-0.610038
32	8	0	-1.511814	2.368418	-0.813316
33	8	0	2.769498	0.874769	0.541688
34	6	0	0.577107	1.537586	-0.077406
35	1	0	-0.377660	-1.883374	-0.697885
36	19	0	4.116273	-1.256646	0.063087
37	6	0	1.069231	-3.452697	-0.330794
38	8	0	-0.168502	-3.127728	-0.356101
39	8	0	1.252479	-4.781368	-0.064435
40	1	0	2.211076	-4.927022	-0.068715
41	8	0	2.056870	-2.722811	-0.513050

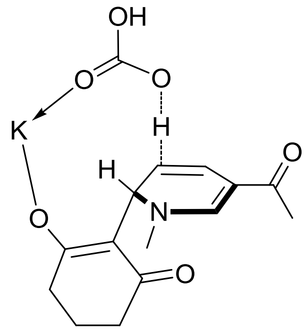

E(RB3LYP/6-31G(d,p)) = -1688.56541564 Ha

ΔG (298.15 K, 1 atm, B3LYP/6-31G(d,p)) = -1688.323778 Ha

E(RB3LYP/6-311++G(d,p)//B3LYP/6-31G(d,p)) = -1688.90823638 Ha

ΔG (298.15 K, 1 atm, B3LYP/6-311++G(d,p)//B3LYP/6-31G(d,p)) = -1688.666599 Ha

TS 24c (conformation 3)

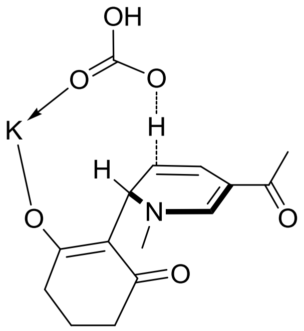

Center Number	Atomic Number	Atomic Type	Coordinates (Angstroms) X	Y	Z
1	6	0	-0.042147	-0.166575	0.252788
2	6	0	2.219262	-0.052495	1.269239
3	6	0	2.861701	0.247327	0.047242
4	6	0	0.638782	0.500236	-0.952462
5	1	0	-0.869823	0.468636	0.579514
6	1	0	2.831956	-0.167246	2.158666
7	1	0	0.075323	0.373186	-1.878137
8	7	0	0.922048	-0.184656	1.413723
9	6	0	0.333192	-0.466351	2.722883
10	1	0	-0.364729	0.333749	2.987561
11	1	0	-0.212643	-1.410989	2.685509
12	1	0	1.118124	-0.525998	3.476673
13	6	0	4.324866	0.337345	0.048631
14	8	0	4.981428	0.147441	1.078254
15	6	0	5.033981	0.670979	-1.253789
16	1	0	4.806473	-0.068820	-2.028554
17	1	0	4.721040	1.649046	-1.634591
18	1	0	6.109764	0.683847	-1.075613
19	6	0	2.037669	0.446813	-1.066139
20	1	0	2.485812	0.677909	-2.029294
21	6	0	-1.903007	-3.870287	-1.121801
22	6	0	-0.440071	-3.976009	-0.693668
23	6	0	-2.682892	-3.037285	-0.105100
24	1	0	-0.367301	-4.568284	0.231138
25	1	0	0.173080	-4.489967	-1.440931
26	1	0	-1.960139	-3.388324	-2.106666
27	1	0	-2.350668	-4.864795	-1.229902
28	1	0	-2.760604	-3.587241	0.845136
29	1	0	-3.709139	-2.844655	-0.435328
30	6	0	-2.035147	-1.686443	0.195345
31	6	0	0.206690	-2.613214	-0.426594
32	8	0	1.445173	-2.512284	-0.556716
33	8	0	-2.790542	-0.770938	0.631584
34	6	0	-0.634315	-1.529618	-0.003651
35	1	0	0.484597	1.825378	-0.757353
36	19	0	-4.043481	1.415823	0.148350
37	6	0	-0.903720	3.446407	-0.459160
38	8	0	0.323023	3.080419	-0.511882
39	8	0	-1.033678	4.795917	-0.290092
40	1	0	-1.986399	4.974316	-0.263586
41	8	0	-1.918151	2.736598	-0.541337

E(RB3LYP/6-31G(d,p)) = -1688.56669063 Ha

ΔG (298.15 K, 1 atm, B3LYP/6-31G(d,p)) = -1688.325376 Ha

E(RB3LYP/6-311++G(d,p)//B3LYP/6-31G(d,p)) = -1688.90939681 Ha

ΔG (298.15 K, 1 atm, B3LYP/6-311++G(d,p)//B3LYP/6-31G(d,p)) = -1688.668081 Ha

TS 24d (conformation 4)

Center Number	Atomic Number	Atomic Type	Coordinates (Angstroms) X	Y	Z
1	6	0	-0.044304	-0.173631	0.248595
2	6	0	2.227498	-0.093279	1.243985
3	6	0	2.852639	0.277960	0.032978
4	6	0	0.615596	0.570134	-0.923237
5	1	0	-0.876284	0.431943	0.618595
6	1	0	2.851422	-0.249002	2.119207
7	1	0	0.039966	0.496686	-1.847065
8	7	0	0.933170	-0.246666	1.395409
9	6	0	0.360819	-0.595173	2.695790
10	1	0	-0.318893	0.199388	3.019228
11	1	0	-0.201310	-1.526828	2.610538
12	1	0	1.156218	-0.712311	3.431638
13	6	0	4.314907	0.381245	0.023013
14	8	0	4.985382	0.134567	1.031436
15	6	0	5.005487	0.802553	-1.263777
16	1	0	4.776829	0.110206	-2.080897
17	1	0	4.678489	1.799138	-1.579265
18	1	0	6.083019	0.815943	-1.096669
19	6	0	2.013049	0.532557	-1.057747
20	1	0	2.447204	0.821071	-2.011765
21	6	0	-1.688883	-4.212458	-0.165915
22	6	0	-0.424067	-3.908002	-0.967683
23	6	0	-2.663156	-3.039821	-0.267819
24	1	0	0.336210	-4.686720	-0.849831
25	1	0	-0.667942	-3.867630	-2.040249
26	1	0	-2.159643	-5.137407	-0.517809
27	1	0	-1.422870	-4.373119	0.887132
28	1	0	-3.533388	-3.172861	0.383321
29	1	0	-3.051313	-2.966472	-1.295270
30	6	0	-2.028998	-1.694800	0.082847
31	6	0	0.219921	-2.571543	-0.585842
32	8	0	1.450622	-2.439620	-0.755366
33	8	0	-2.805775	-0.779693	0.480484
34	6	0	-0.624336	-1.527630	-0.077106
35	1	0	0.456590	1.878180	-0.642193
36	19	0	-4.072729	1.394524	-0.014168
37	6	0	-0.940423	3.480489	-0.307437
38	8	0	0.286236	3.110181	-0.301455
39	8	0	-1.080355	4.810722	-0.028539
40	1	0	-2.032105	4.993875	-0.057929
41	8	0	-1.946990	2.789473	-0.528217

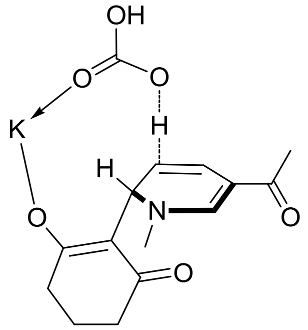

E(RB3LYP/6-31G(d,p)) = -1688.56679936 Ha

ΔG (298.15 K, 1 atm, B3LYP/6-31G(d,p)) = -1688.325304 Ha

E(RB3LYP/6-311++G(d,p)//B3LYP/6-31G(d,p)) = -1688.90936289 Ha

ΔG (298.15 K, 1 atm, B3LYP/6-311++G(d,p)//B3LYP/6-31G(d,p)) = -1688.667868 Ha

25a (conformation 1)

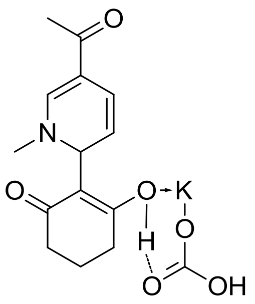

Center Number	Atomic Number	Atomic Type	Coordinates (Angstroms) X	Y	Z
1	6	0	-0.891480	-0.314222	0.189696
2	6	0	-3.200630	-0.307288	1.132870
3	6	0	-3.786438	-0.793234	-0.020291
4	6	0	-1.564966	-0.919002	-1.024320
5	1	0	-0.148781	-1.024117	0.580532
6	1	0	-3.796757	-0.064517	2.006796
7	1	0	-0.900111	-1.165795	-1.848285
8	7	0	-1.883028	-0.124751	1.288686
9	6	0	-1.334395	0.318434	2.561830
10	1	0	-0.548539	-0.372313	2.890307
11	1	0	-0.895173	1.316988	2.474909
12	1	0	-2.120959	0.338900	3.317368
13	6	0	-5.214778	-0.996007	-0.148961
14	8	0	-5.716085	-1.455350	-1.189792
15	6	0	-6.138273	-0.637120	1.014880
16	1	0	-5.874224	-1.182270	1.927222
17	1	0	-6.094297	0.432284	1.247183
18	1	0	-7.160826	-0.894336	0.734669
19	6	0	-2.886258	-1.124514	-1.117974
20	1	0	-3.326467	-1.548871	-2.014917
21	6	0	1.408823	3.141294	-1.263038
22	6	0	-0.001444	3.431856	-0.755326
23	6	0	2.114982	2.170137	-0.317208
24	1	0	0.053479	3.990354	0.191282
25	1	0	-0.573474	4.052189	-1.452049
26	1	0	1.352297	2.694895	-2.264167
27	1	0	1.987484	4.066259	-1.359454
28	1	0	2.333546	2.669851	0.638360
29	1	0	3.079761	1.849903	-0.722671
30	6	0	1.287818	0.929212	-0.017806
31	6	0	-0.814130	2.162254	-0.492036
32	8	0	-2.052303	2.225673	-0.560767
33	8	0	1.943252	-0.133228	0.349308
34	6	0	-0.103163	0.955115	-0.121159
35	1	0	3.258009	0.024861	0.722521
36	19	0	2.375077	-2.429310	-1.045141
37	6	0	5.083061	-0.714004	0.515139
38	8	0	4.279889	0.147116	1.067094
39	8	0	6.357806	-0.517306	0.916074
40	1	0	6.894388	-1.194107	0.474683
41	8	0	4.785315	-1.614088	-0.272566

E(RB3LYP/6-31G(d,p)) = -1688.59746464 Ha

ΔG (298.15 K, 1 atm, B3LYP/6-31G(d,p)) = -1688.352661 Ha

25b (conformation 2)

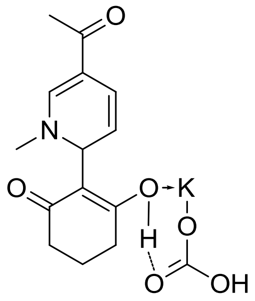

Center Number	Atomic Number	Atomic Type	X	Y	Z
1	6	0	-0.547476	-0.118927	0.114873
2	6	0	-2.801534	-0.303635	1.163654
3	6	0	-3.372755	-0.924261	0.070132
4	6	0	-1.183431	-0.907339	-1.009731
5	1	0	0.300019	-0.695543	0.511430
6	1	0	-3.386650	-0.066757	2.046668
7	1	0	-0.509755	-1.179369	-1.816764
8	7	0	-1.504560	0.023353	1.249603
9	6	0	-0.950162	0.570493	2.478682
10	1	0	-0.123634	-0.059788	2.830779
11	1	0	-0.563034	1.581014	2.317680
12	1	0	-1.719366	0.601368	3.251576
13	6	0	-4.777831	-1.271167	0.011976
14	8	0	-5.265345	-1.850965	-0.973765
15	6	0	-5.694511	-0.917573	1.183075
16	1	0	-5.347764	-1.364093	2.120978
17	1	0	-5.750697	0.165424	1.336231
18	1	0	-6.694611	-1.293999	0.962972
19	6	0	-2.477419	-1.255833	-1.031896
20	1	0	-2.895135	-1.812556	-1.864994
21	6	0	1.100467	3.908526	-0.499429
22	6	0	-0.220775	3.608079	-1.204305
23	6	0	2.055123	2.729227	-0.671171
24	1	0	-0.963646	4.396422	-1.049645
25	1	0	-0.053593	3.538294	-2.289761
26	1	0	1.551472	4.827592	-0.889106
27	1	0	0.913456	4.073375	0.569600
28	1	0	2.950879	2.842047	-0.050286
29	1	0	2.410720	2.678454	-1.711491
30	6	0	1.429017	1.385161	-0.334408
31	6	0	-0.844438	2.289140	-0.742491
32	8	0	-2.080143	2.174170	-0.770264
33	8	0	2.281896	0.437410	-0.084955
34	6	0	0.043099	1.221554	-0.320899
35	1	0	2.155982	-0.859117	-0.643516
36	19	0	4.446903	0.000736	1.398617
37	6	0	3.042651	-2.604171	-0.639938
38	8	0	2.109113	-1.816325	-1.099230
39	8	0	2.960004	-3.825137	-1.205327
40	1	0	3.677957	-4.356613	-0.827213
41	8	0	3.905477	-2.325312	0.191687

E(RB3LYP/6-31G(d,p)) = -1688.59552748 Ha

ΔG (298.15 K, 1 atm, B3LYP/6-31G(d,p)) = -1688.351293 Ha

25c (conformation 3)

Center Number	Atomic Number	Atomic Type	X	Y	Z
1	6	0	-0.576065	-0.111935	0.087178
2	6	0	-2.767811	-0.372863	1.235418
3	6	0	-3.421039	-0.857711	0.116830
4	6	0	-1.297876	-0.748195	-1.081131
5	1	0	0.269703	-0.753562	0.368804
6	1	0	-3.327717	-0.242628	2.156718
7	1	0	-0.682315	-0.929607	-1.957229
8	7	0	-1.466746	-0.076317	1.284688
9	6	0	-0.837158	0.337527	2.529669
10	1	0	0.004569	-0.326475	2.762637
11	1	0	-0.454182	1.359631	2.453303
12	1	0	-1.560375	0.286368	3.344474
13	6	0	-4.838285	-1.150158	0.222335
14	8	0	-5.495437	-0.954215	1.260884
15	6	0	-5.543602	-1.727433	-1.000554
16	1	0	-5.459556	-1.057209	-1.863228
17	1	0	-5.104481	-2.687104	-1.295923
18	1	0	-6.598486	-1.876116	-0.764575
19	6	0	-2.600629	-1.072018	-1.069190
20	1	0	-3.047285	-1.518350	-1.953021
21	6	0	1.175884	3.663831	-1.367427
22	6	0	-0.200422	3.756493	-0.713695
23	6	0	2.072291	2.729185	-0.556028
24	1	0	-0.115708	4.270038	0.256039
25	1	0	-0.907018	4.337930	-1.313914
26	1	0	1.071448	3.269775	-2.386497
27	1	0	1.634078	4.655028	-1.455278
28	1	0	2.319950	3.193517	0.410932
29	1	0	3.023754	2.541662	-1.064248
30	6	0	1.426464	1.380119	-0.276922
31	6	0	-0.835810	2.388302	-0.451538
32	8	0	-2.074083	2.315958	-0.393737
33	8	0	2.260742	0.409219	-0.066113
34	6	0	0.036301	1.250856	-0.232090
35	1	0	2.140455	-0.892252	-0.658491
36	19	0	4.459389	-0.002693	1.360047
37	6	0	3.053504	-2.614510	-0.683144
38	8	0	2.098776	-1.837355	-1.121482
39	8	0	2.978542	-3.831100	-1.257143
40	1	0	3.710258	-4.355790	-0.896216
41	8	0	3.924865	-2.327307	0.135558

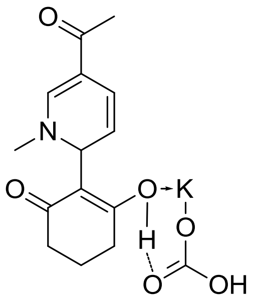

E(RB3LYP/6-31G(d,p)) = -1688.59462765 Ha

ΔG (298.15 K, 1 atm, B3LYP/6-31G(d,p)) = -1688.350210 Ha

25d (conformation 4)

Center Number	Atomic Number	Atomic Type	X	Y	Z
1	6	0	-0.537236	-0.089354	0.128927
2	6	0	-2.754474	-0.240234	1.246718
3	6	0	-3.381959	-0.833255	0.166531
4	6	0	-1.229233	-0.848386	-0.982534
5	1	0	0.308455	-0.690578	0.490633
6	1	0	-3.335025	-0.020770	2.137813
7	1	0	-0.589923	-1.125364	-1.815099
8	7	0	-1.453733	0.059821	1.297349
9	6	0	-0.848747	0.576173	2.516241
10	1	0	-0.029984	-0.080823	2.836001
11	1	0	-0.438906	1.577302	2.354235
12	1	0	-1.595297	0.618272	3.310209
13	6	0	-4.802567	-1.111859	0.265694
14	8	0	-5.485353	-0.806087	1.260123
15	6	0	-5.477901	-1.814804	-0.907202
16	1	0	-5.378517	-1.232962	-1.830348
17	1	0	-5.027026	-2.795541	-1.096305
18	1	0	-6.536889	-1.947216	-0.680123
19	6	0	-2.532424	-1.170066	-0.969495
20	1	0	-2.957144	-1.708226	-1.812144
21	6	0	1.174677	3.910407	-0.478409
22	6	0	-0.173475	3.648948	-1.146626
23	6	0	2.099194	2.715505	-0.699915
24	1	0	-0.895443	4.448308	-0.954082
25	1	0	-0.041775	3.596663	-2.237919
26	1	0	1.632278	4.826948	-0.866292
27	1	0	1.024301	4.060046	0.598602
28	1	0	3.016381	2.800755	-0.106607
29	1	0	2.420162	2.674902	-1.751846
30	6	0	1.457289	1.378713	-0.363687
31	6	0	-0.808699	2.333883	-0.689559
32	8	0	-2.046532	2.241281	-0.686928
33	8	0	2.299059	0.411298	-0.156420
34	6	0	0.069182	1.243441	-0.308027
35	1	0	2.121099	-0.887423	-0.690789
36	19	0	4.516630	-0.068349	1.234256
37	6	0	2.893747	-2.684265	-0.604609
38	8	0	2.039010	-1.850394	-1.131262
39	8	0	2.755512	-3.913934	-1.139556
40	1	0	3.418875	-4.479202	-0.713715
41	8	0	3.733340	-2.437701	0.260440

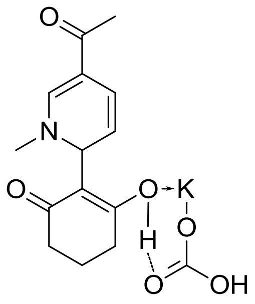

E(RB3LYP/6-31G(d,p)) = -1688.59526377 Ha

ΔG (298.15 K, 1 atm, B3LYP/6-31G(d,p)) = -1688.350633 Ha

Explicit inclusion of acetonitrile

Explicit inclusion of acetonitrile molecules in computation always led to their coordination on potassium cation. However, this coordination leads to free energy rising, if compared with free K-bounded product and free acetonitrile (acetonitrile was still included implicitly as solvent within IEFPCM model). The energy rising is illustrated in the following scheme: positive ΔG shows that rather MeCN dissociation proceeds.

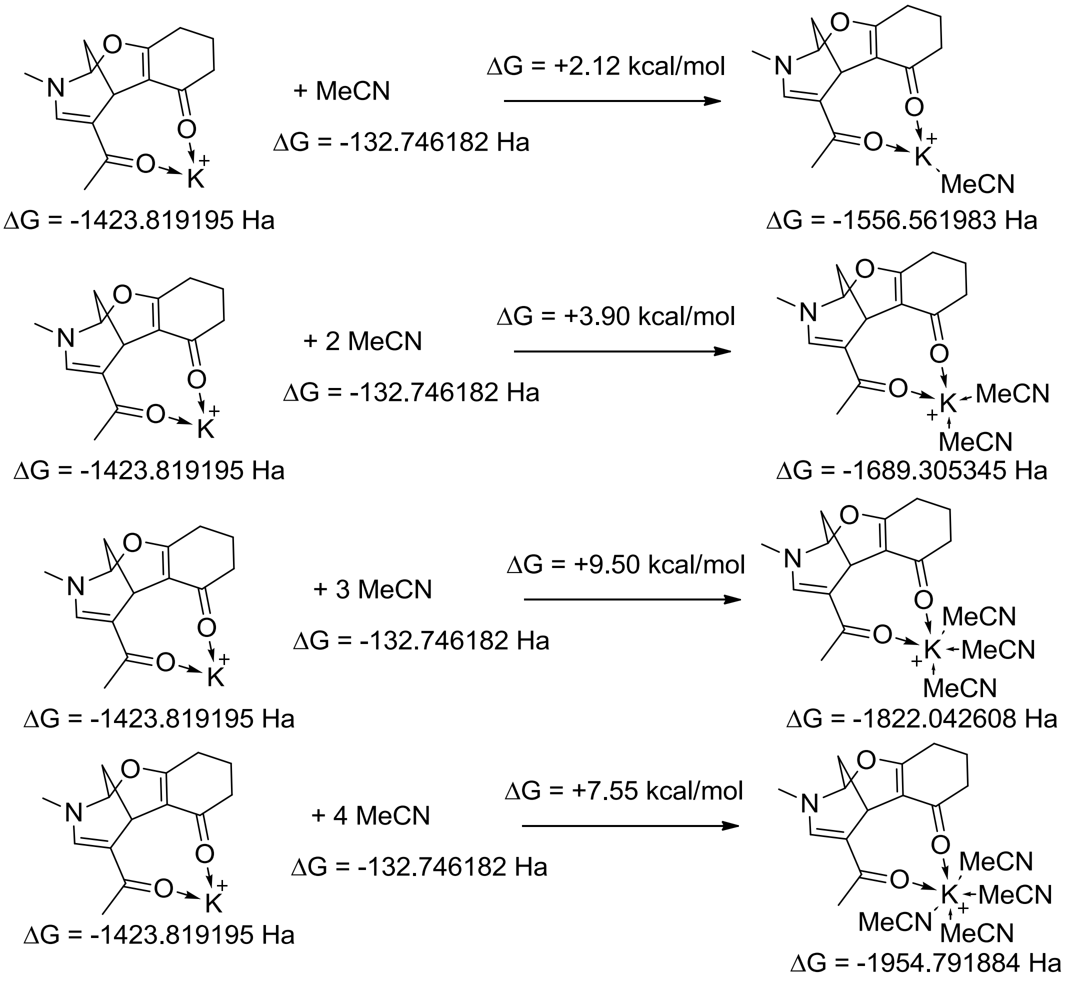

We tried also to compute whether MeCN can serve as base to induce enolization/enamine protonation; however, no reasonable TSs were found.

On the other hand, any attempt to do calculations for molecules in vacuum led to irrelevant results, so that acetonitrile is included implicitly in all computations reported in this article.

6a-K (conformation 2) + 1 MeCN

Center Number	Atomic Number	Atomic Type	Coordinates (Angstroms) X	Y	Z
1	6	0	-3.541907	-0.438882	1.130076
2	6	0	-2.439206	-1.953626	-0.417545
3	6	0	-1.189144	-1.524203	-0.059116
4	6	0	-2.256191	-0.540560	1.934975
5	1	0	-4.438486	-0.523126	1.744700
6	1	0	-2.584360	-2.757253	-1.130883
7	1	0	-2.250216	0.242507	2.697355
8	7	0	-3.592567	-1.450401	0.099079
9	6	0	-4.895458	-1.765936	-0.482149
10	1	0	-5.286677	-0.916645	-1.052374
11	1	0	-4.796682	-2.621868	-1.150739
12	1	0	-5.607849	-2.018623	0.308136
13	6	0	0.004170	-2.192514	-0.542674
14	8	0	1.135370	-1.870316	-0.139026
15	6	0	-0.108772	-3.332978	-1.546992
16	1	0	-0.555482	-4.218588	-1.082815
17	1	0	-0.724071	-3.063280	-2.410109
18	1	0	0.894326	-3.589292	-1.890116
19	6	0	-1.104839	-0.369695	0.933199
20	1	0	-0.134007	-0.396232	1.429333
21	6	0	-1.705409	3.343867	-1.357237
22	6	0	-2.836570	2.878271	-0.437274
23	6	0	-0.355353	3.200401	-0.653908
24	1	0	-2.975792	3.587500	0.391142
25	1	0	-3.792856	2.826860	-0.966725
26	1	0	-1.710288	2.736111	-2.269787
27	1	0	-1.870917	4.381457	-1.660880
28	1	0	-0.289510	3.913314	0.181393
29	1	0	0.482570	3.422637	-1.320867
30	6	0	-0.125934	1.815384	-0.064421
31	6	0	-2.538177	1.524702	0.140774
32	8	0	-3.665414	0.891140	0.531600
33	8	0	1.032156	1.432638	0.153225
34	6	0	-1.284350	1.001842	0.286772
35	1	0	-2.219648	-1.512605	2.434174
36	19	0	3.098449	-0.184619	0.270163
37	6	0	8.556753	0.108741	0.052208
38	1	0	8.932028	0.676818	0.906979
39	1	0	8.852237	0.614975	-0.870068
40	1	0	8.997081	-0.891277	0.067236
41	6	0	7.104183	0.011678	0.120693
42	7	0	5.948052	-0.065636	0.175368

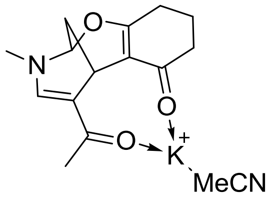

E(RB3LYP/6-31G(d,p)) = -1556.82693232 Ha

ΔG (298.15 K, 1 atm, B3LYP/6-31G(d,p)) = -1556.561983 Ha

6a-K (conformation 2) + 2 MeCN

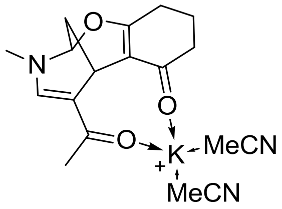

Center Number	Atomic Number	Atomic Type	Coordinates (Angstroms) X	Y	Z
1	6	0	-3.941016	-0.505410	-1.109835
2	6	0	-2.922213	1.699310	-1.006759
3	6	0	-1.651819	1.189849	-0.972716
4	6	0	-2.634577	-0.929271	-1.761900
5	1	0	-4.820805	-0.895976	-1.621916
6	1	0	-3.109258	2.766862	-1.035252
7	1	0	-2.586113	-2.020932	-1.785959
8	7	0	-4.047003	0.934404	-1.043560
9	6	0	-5.371828	1.511455	-0.826785
10	1	0	-5.743541	1.276422	0.176318
11	1	0	-5.317186	2.595217	-0.936229
12	1	0	-6.076737	1.119465	-1.565115
13	6	0	-0.491368	2.054784	-1.079784
14	8	0	0.658379	1.589279	-1.162468
15	6	0	-0.666924	3.568288	-1.116392
16	1	0	-1.158608	3.883383	-2.042681
17	1	0	-1.270851	3.930660	-0.279340
18	1	0	0.319757	4.030896	-1.070988
19	6	0	-1.509902	-0.326118	-0.907384
20	1	0	-0.525705	-0.608467	-1.282793
21	6	0	-2.087945	-1.491923	3.300705
22	6	0	-3.199251	-1.830298	2.303949
23	6	0	-0.718439	-1.807523	2.697772
24	1	0	-3.290594	-2.919307	2.183727
25	1	0	-4.174127	-1.472785	2.649449
26	1	0	-2.141345	-0.425767	3.551043
27	1	0	-2.234935	-2.048548	4.230629
28	1	0	-0.603841	-2.894785	2.575243
29	1	0	0.101495	-1.477483	3.342056
30	6	0	-0.507786	-1.188722	1.322790
31	6	0	-2.918885	-1.225425	0.958081
32	8	0	-4.049414	-1.077707	0.233104
33	8	0	0.645651	-1.000905	0.911486
34	6	0	-1.675521	-0.891226	0.501177
35	1	0	-2.607520	-0.557484	-2.789778
36	19	0	2.669357	0.087615	-0.372913
37	6	0	6.012574	3.734937	1.967079
38	1	0	6.833035	3.972079	1.285348
39	1	0	6.424865	3.327706	2.893555
40	1	0	5.461397	4.650781	2.194176
41	6	0	5.121736	2.760258	1.350296
42	7	0	4.412831	1.984347	0.859073
43	6	0	5.650949	-4.227601	-1.942236
44	1	0	6.689851	-4.085814	-1.634473
45	1	0	5.614960	-4.359919	-3.026327
46	1	0	5.255478	-5.124947	-1.460024
47	6	0	4.857096	-3.067508	-1.557366
48	7	0	4.225077	-2.144238	-1.250721

E(RB3LYP/6-31G(d,p)) = -1689.60159577 Ha

ΔG (298.15 K, 1 atm, B3LYP/6-31G(d,p)) = -1689.305345 Ha

6a-K (conformation 2) + 3 MeCN

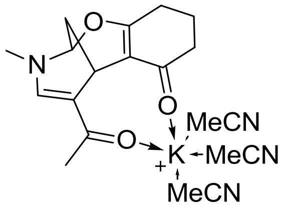

Center Number	Atomic Number	Atomic Type	Coordinates (Angstroms) X	Y	Z
1	6	0	-4.331822	-0.474558	1.122051
2	6	0	-3.309047	-1.967975	-0.498846
3	6	0	-2.048511	-1.501779	-0.239586
4	6	0	-2.986643	-0.537486	1.828088
5	1	0	-5.177287	-0.576136	1.803118
6	1	0	-3.486204	-2.778704	-1.196701
7	1	0	-2.943751	0.249769	2.585033
8	7	0	-4.433750	-1.495488	0.104956
9	6	0	-5.766772	-1.839959	-0.383923
10	1	0	-6.207652	-1.008104	-0.943806
11	1	0	-5.699218	-2.708242	-1.040481
12	1	0	-6.421776	-2.085560	0.456545
13	6	0	-0.876618	-2.134431	-0.817555
14	8	0	0.271704	-1.778708	-0.502953
15	6	0	-1.036504	-3.274447	-1.816554
16	1	0	-1.440860	-4.168150	-1.329804
17	1	0	-1.709345	-3.009519	-2.637394
18	1	0	-0.053798	-3.514538	-2.224261
19	6	0	-1.919007	-0.342073	0.741296
20	1	0	-0.912809	-0.342304	1.161770
21	6	0	-2.781500	3.343419	-1.506644
22	6	0	-3.832761	2.850642	-0.509262
23	6	0	-1.381825	3.240602	-0.899420
24	1	0	-3.936903	3.560042	0.324214
25	1	0	-4.820985	2.768299	-0.972145
26	1	0	-2.832712	2.731513	-2.415012
27	1	0	-2.996812	4.374570	-1.800989
28	1	0	-1.278014	3.957951	-0.071867
29	1	0	-0.599573	3.484259	-1.624014
30	6	0	-1.072470	1.864169	-0.325802
31	6	0	-3.456155	1.509279	0.051576
32	8	0	-4.534521	0.847667	0.526390
33	8	0	0.107337	1.513180	-0.189430
34	6	0	-2.181687	1.021278	0.107475
35	1	0	-2.887014	-1.505406	2.326836
36	19	0	2.251068	-0.053843	-0.127368
37	6	0	6.010314	-2.936244	-2.924102
38	1	0	6.764614	-3.306094	-2.225208
39	1	0	6.496833	-2.308171	-3.674409
40	1	0	5.538491	-3.786697	-3.422408
41	6	0	5.005649	-2.162475	-2.205450
42	7	0	4.206210	-1.546402	-1.633334
43	6	0	3.776818	-1.545159	4.932382
44	1	0	4.517327	-0.834251	5.307123
45	1	0	4.223164	-2.542124	4.901595
46	1	0	2.920303	-1.558719	5.610873
47	6	0	3.345243	-1.153568	3.596427
48	7	0	3.002172	-0.841772	2.532887
49	6	0	5.568906	4.342902	-0.189998
50	1	0	6.540620	4.095467	0.244365
51	1	0	5.117546	5.154721	0.385633
52	1	0	5.713259	4.674609	-1.221147
53	6	0	4.700490	3.172494	-0.162553
54	7	0	4.009584	2.240751	-0.141242

E(RB3LYP/6-31G(d,p)) = -1822.37522478 Ha

ΔG (298.15 K, 1 atm, B3LYP/6-31G(d,p)) = -1822.042608 Ha

6a-K (conformation 2) + 4 MeCN

Center Number	Atomic Number	Atomic Type	Coordinates (Angstroms) X	Y	Z
1	6	0	-4.467154	-0.942680	0.720470
2	6	0	-3.418509	-1.544903	-1.386587
3	6	0	-2.164987	-1.233742	-0.933673
4	6	0	-3.125440	-1.305118	1.336949
5	1	0	-5.315120	-1.344258	1.276074
6	1	0	-3.581856	-1.958916	-2.375263
7	1	0	-3.095815	-0.940622	2.366995
8	7	0	-4.552262	-1.402704	-0.646273
9	6	0	-5.878597	-1.499851	-1.251037
10	1	0	-6.325776	-0.508307	-1.380603
11	1	0	-5.798012	-1.978848	-2.227661
12	1	0	-6.535524	-2.102718	-0.617956
13	6	0	-0.982782	-1.535007	-1.721461
14	8	0	0.159564	-1.349487	-1.270100
15	6	0	-1.125565	-2.113856	-3.124384
16	1	0	-1.516157	-3.136304	-3.086918
17	1	0	-1.802559	-1.521780	-3.747116
18	1	0	-0.139344	-2.134824	-3.589777
19	6	0	-2.052998	-0.635778	0.464562
20	1	0	-1.050288	-0.819313	0.851848
21	6	0	-2.943695	3.659935	0.097439
22	6	0	-3.996351	2.764112	0.754774
23	6	0	-1.548334	3.307182	0.613912
24	1	0	-4.115500	3.024303	1.816336
25	1	0	-4.980007	2.890307	0.291947
26	1	0	-2.978851	3.518792	-0.989341
27	1	0	-3.170313	4.712018	0.292681
28	1	0	-1.462356	3.577571	1.676860
29	1	0	-0.762930	3.856636	0.087070
30	6	0	-1.225215	1.822215	0.513517
31	6	0	-3.607168	1.316593	0.659750
32	8	0	-4.681353	0.504525	0.775622
33	8	0	-0.042412	1.457044	0.495680
34	6	0	-2.327394	0.865202	0.506522
35	1	0	-3.018203	-2.393194	1.350008
36	19	0	2.108375	-0.054195	0.004440
37	6	0	3.390734	2.918105	-4.477340
38	1	0	4.283887	2.519361	-4.964524
39	1	0	3.570522	3.959989	-4.201068
40	1	0	2.553208	2.874679	-5.177989
41	6	0	3.082590	2.134961	-3.287061
42	7	0	2.836870	1.511870	-2.339750
43	6	0	1.067318	-3.022520	4.559734
44	1	0	1.984843	-3.245889	5.109675
45	1	0	0.587131	-3.960996	4.271853
46	1	0	0.391044	-2.460583	5.208600
47	6	0	1.379083	-2.239535	3.370234
48	7	0	1.627283	-1.616651	2.423304
49	6	0	5.951833	-3.736710	-1.491944
50	1	0	5.588979	-4.744397	-1.274953
51	1	0	6.889269	-3.572123	-0.955034
52	1	0	6.135498	-3.647547	-2.565461
53	6	0	4.961369	-2.752101	-1.074349
54	7	0	4.172981	-1.968389	-0.742232
55	6	0	5.535838	3.053810	3.034952
56	1	0	6.401798	2.461050	3.339727
57	1	0	5.018309	3.413826	3.927526
58	1	0	5.878797	3.911751	2.451518
59	6	0	4.632904	2.237233	2.233226
60	7	0	3.914407	1.587034	1.595200

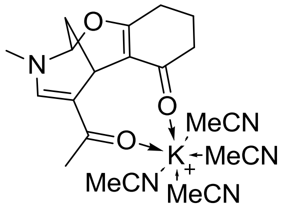

E(RB3LYP/6-31G(d,p)) = -1955.14854441 Ha

ΔG (298.15 K, 1 atm, B3LYP/6-31G(d,p)) = -1954.791884 Ha

Acetonitrile

Center Number	Atomic Number	Atomic Type	Coordinates (Angstroms) X	Y	Z
1	6	0	0.000000	0.000000	-1.180518
2	1	0	0.000000	1.026880	-1.554865
3	1	0	-0.889304	-0.513440	-1.554865
4	1	0	0.889304	-0.513440	-1.554865
5	6	0	0.000000	0.000000	0.278407
6	7	0	0.000000	0.000000	1.439609

E(RB3LYP/6-31G(d,p)) = -132.766833279 Ha

ΔG (298.15 K, 1 atm, B3LYP/6-31G(d,p)) = -132.746182 Ha
